# Radiotherapy with cetuximab or durvalumab for locoregionally advanced head and neck cancer in patients with a contraindication to cisplatin (NRG-HN004): an open-label, multicentre, parallel-group, randomised, phase 2/3 trial

**DOI:** 10.1016/S1470-2045(24)00507-2

**Published:** 2024-11-14

**Authors:** Loren K Mell, Pedro A Torres-Saavedra, Stuart J Wong, Julie A Kish, Steven S Chang, Richard C Jordan, Tian Liu, Minh Tam Truong, Eric W Winquist, Vinita Takiar, Trisha Wise-Draper, Jared R Robbins, Cristina P Rodriguez, Musaddiq J Awan, Beth M Beadle, Christina Henson, Samir Narayan, Sharon A Spencer, Steven Powell, Neal Dunlap, Assuntina G Sacco, Kenneth Shung Hu, Henry S Park, Julie E Bauman, Jonathan Harris, Sue S Yom, Quynh-Thu Le

**Affiliations:** Department of Radiation Medicine and Applied Sciences, University of California San Diego, La Jolla, CA, USA (Prof L K Mell MD); Biometric Research Program, Division of Cancer Treatment and Diagnosis, NCI, Bethesda, MD, USA (P A Torres-Saavedra PhD); Division of Hematology Oncology, Department of Medicine, Medical College of Wisconsin, Milwaukee, WI, USA (Prof S J Wong MD); Division of Hematology Oncology, Department of Medicine, Moffitt Cancer Center, Tampa, FL, USA (Prof J A Kish MD); Department of Otorhinolaryngology, Henry Ford Health System, Detroit, MI, USA (S S Chang MD); NRG Oncology Biospecimen Bank, University of California San Francisco, San Francisco, CA, USA (Prof R C Jordan DDS PhD); Department of Radiation Oncology, Emory University, Atlanta, GA, USA (T Liu PhD); Department of Radiation Oncology, Boston Medical Center, Boston, MA, USA (Prof M T Truong MD); Department of Oncology, London Regional Cancer Program, London, ON, Canada (Prof E W Winquist MD); Department of Radiation Oncology (V Takiar MD PhD) and Division of Hematology Oncology (T Wise-Draper MD PhD), University of Cincinnati, Cincinnati, OH, USA; Department of Radiation Oncology, University of Arizona College of Medicine, Tucson, AZ, USA (Prof J R Robbins MD); Division of Hematology Oncology, University of Washington, Seattle, WA, USA (Prof C P Rodriguez MD); Department of Radiation Oncology, Medical College of Wisconsin, Milwaukee, WI, USA (M J Awan MD); Department of Radiation Oncology, Stanford University School of Medicine, Stanford, CA, USA (B M Beadle MD PhD, Q-T Le MD); Department of Radiation Oncology, University of Oklahoma, Oklahoma City, OK, USA (C Henson MD); Department of Radiation Oncology, Trinity Health Ann Arbor, Ann Arbor, MI, USA (S Narayan MD); Department of Radiation Oncology, University of Alabama Birmingham, Birmingham, AL, USA (Prof S A Spencer MD); Sanford USD Medical Center, Sioux Falls, SD, USA (S Powell MD); Department of Radiation Oncology, University of Louisville, Louisville, KY, USA (Prof N Dunlap MD); Division of Hematology Oncology, Department of Medicine, University of California San Diego, La Jolla, CA, USA (A G Sacco MD); Department of Radiation Oncology, New York University Langone Medical Center, New York, NY, USA (Prof K S Hu MD); Department of Therapeutic Radiology, Yale University School of Medicine, New Haven, CT, USA (H S Park MD MPH); Division of Hematology/Oncology, Department of Medicine, George Washington University and George Washington Cancer Center, Washington, DC, USA (Prof J E Bauman MD MPH); NRG Oncology Statistics and Data Management Center, American College of Radiology, Philadelphia, PA, USA (J Harris MS); Department of Radiation Oncology, University of California San Francisco, San Francisco, CA, USA (Prof S S Yom MD PhD)

## Abstract

**Background:**

Management of patients with locoregionally advanced head and neck squamous cell carcinoma (HNSCC) when cisplatin is contraindicated is controversial. We aimed to assess whether radiotherapy with concurrent and adjuvant durvalumab would improve outcomes compared with radiotherapy with cetuximab.

**Methods:**

NRG-HN004 was designed as an open-label, multicentre, parallel-group, randomised, phase 2/3 trial with safety lead-in conducted at 89 academic and community medical centres in North America. Eligible patients were aged 18 years or older with American Joint Committee on Cancer 8th edition stage III–IVB p16-negative HNSCC or unfavourable stage I–III p16-positive oropharyngeal or unknown primary carcinoma, who had a contraindication to cisplatin (Eastern Cooperative Oncology Group [ECOG] performance status 2, renal or hearing impairment, peripheral neuropathy, aged at least 70 years with moderate or severe comorbidity, or aged younger than 70 years with severe comorbidity). Patients were randomly assigned (2:1) by permuted block randomisation (multiples of 6) to intravenous durvalumab 1500 mg starting 2 weeks before radiotherapy then every 4 weeks starting week 2 of radiotherapy (seven cycles) or intravenous cetuximab 400 mg/m2 1 week before radiotherapy then 250 mg/m2 weekly beginning week 1 of radiotherapy (eight cycles), with intensity-modulated radiotherapy (70 Gy in 35 fractions over 7 weeks). Stratification factors were tumour and nodal stage, ECOG performance status and comorbidity, and primary site and p16 status. The phase 2 primary endpoint was progression-free survival in the intention-to-treat population. There was one prespecified interim futility analysis at 50% of progression-free survival information. If the observed hazard ratio was 1·0 or more, favouring cetuximab, early stopping would be considered. Extended follow-up analysis was post hoc. This trial is registered with ClinicalTrials.gov, NCT03258554, and is closed to enrolment.

**Findings:**

Following a ten-patient safety lead-in, the phase 2 trial enrolled 190 patients from March 12, 2019, to July 30, 2021, 186 of whom were randomly assigned (123 to durvalumab and 63 to cetuximab). Median age was 72 years (IQR 64–77), 30 (16%) patients were women and 156 (84%) were men. Phase 2 accrual was suspended in July 30, 2021, following an interim futility analysis, and permanently closed in Sept 1, 2022. The phase 3 part of the trial was not conducted. At a median follow-up of 2·3 years (IQR 1·9–3·1) for the extended follow-up (data cutoff July 31, 2023; post-hoc analysis), 2-year progression-free survival was 50·6% (95% CI 41·5–59·8) in the durvalumab group versus 63·7% (51·3–76·1) in the cetuximab group (hazard ratio 1·33 [95% CI 0·84–2·12]; p=0·89). Adverse events were similar in both groups. The most common grade 3–4 adverse events were dysphagia (26 [22%] of 119 patients in the durvalumab group *vs* 18 [30%] of 61 patients in the cetuximab group), lymphopenia (33 [28%] *vs* 20 [33%]), and oral mucositis (13 [11%] *vs* 11 [18%]). Four (3%) patients in the durvalumab group and one (2%) in the cetuximab group died from treatment-related adverse events (death not otherwise specified, laryngeal oedema, lung infection, and respiratory failure in the durvalumab group and sudden death not otherwise specified in the cetuximab group).

**Interpretation:**

Our findings suggest that durvalumab did not improve outcomes compared with cetuximab in patients with HNSCC with contraindications to cisplatin. Further trials are needed to define the standard of care for this population.

**Funding:**

US National Cancer Institute and AstraZeneca.

## Introduction

Concurrent radiotherapy with cisplatin is standard definitive treatment for locoregionally advanced head and neck squamous cell carcinoma (HNSCC).^1^ However, cisplatin is contraindicated in more than a third of patients, due to older age, poor renal function, hearing loss, neuropathy, and other comorbidities, with correspondingly poor outcomes.^2–4^ Common alternatives to cisplatin for this population include concurrent cetuximab, docetaxel, and carboplatin (with or without paclitaxel or fluorouracil), with no consensus regarding the standard of care.

The management of patients considered unfit for cisplatin is controversial.^5^ In patterns of care studies, cetuximab has been the most commonly used radiosensitiser in lieu of cisplatin, and is therefore a standard drug in trials involving patients with HNSCC who are ineligible for cisplatin, despite the absence of direct randomised data among cisplatin alternatives.^2^ However, some studies have questioned the effectiveness of cetuximab in older, cisplatin-ineligible populations relative to carboplatin plus paclitaxel or radiotherapy alone.^6,7^ Thus, some centres have advocated cytotoxic radiosensitisers over cetuximab due to perceived loss of efficacy and cost considerations.^8,9^

Immune checkpoint inhibitors are effective for treating recurrent or metastatic HNSCC,^10,11^ and have been actively investigated in the concurrent and adjuvant setting in previously untreated populations undergoing radiotherapy,^12,13^ including cisplatin-ineligible populations.^14,15^ Durvalumab, a PD-L1 inhibitor, showed initial promise in patients with HNSCC,^16^ with low toxicity, and was thus an attractive strategy to augment outcomes for medically frail patients unfit for cisplatin. We thus aimed to test the hypothesis that concurrent and adjuvant durvalumab with radiotherapy could improve outcomes compared with cetuximab with radiotherapy for this population.

## Methods

### Study design and participants

NRG-HN004 was designed as an open-label, multicentre, parallel-group, randomised, phase 2/3 trial with safety lead-in conducted through the US National Cancer Institute’s Clinical Trial Network Group NRG Oncology, involving 89 academic and community medical centres in North America (appendix pp 33–34). Details of the lead-in study design and results are in the appendix (p 3). Phase 2 involved a go–no-go decision to assess whether the experimental group showed a promising efficacy signal (a statistically significant result at phase 2 analysis) to continue to the definitive phase 3. Details of the phase 3 design are provided in the trial protocol in the appendix. The study was approved by a central institutional review board or the institutional review board for each participating centre. All participants provided written, informed consent. This trial is registered with ClinicalTrials.gov, NCT03258554.

Eligible participants were aged 18 years or older with American Joint Committee on Cancer 8th edition stage III–IVB (T3–4b N0 M0 or T0–4b N1–3 M0) p16-negative squamous cell carcinoma of the larynx, hypopharynx, oropharynx, oral cavity, or unknown HNSCC, or unfavourable risk (ie, any T4 or N2–3 M0; or T0–2 N1 M0 or T3 N0–1 M0 with >10 pack-year smoking history) p16-positive squamous cell carcinoma of the oropharynx, with a contraindication to cisplatin. Contraindications to cisplatin were Eastern Cooperative Oncology Group (ECOG) performance status 2, renal impairment (creatinine clearance <60 mL/min), hearing impairment (defined as either existing need of a hearing aid or ≥25 decibel shift over two contiguous frequencies on a pretreatment hearing test), grade 1 or higher peripheral neuropathy, or vulnerability to cisplatin. Vulnerability to cisplatin was defined as either age 70 years or older with at least one, or younger than 70 years with at least two of the following conditions: modified (ie, excluding cancer diagnosis) Charlson Comorbidity Index (CCI) 1 or higher,^17^ Adult Comorbidity Evaluation (ACE)-27 index 1 or higher,^18^ Head and Neck Cancer Intergroup (HNCIG) omega score lower than 0·80,^19^ G-8 score 14 or lower,^20^ Cancer and Aging Research Group (CARG) toxicity score 30% or higher,^21^ or Cumulative Illness Rating Scale-Geriatric score 4 or higher.^22^ Exclusion criteria included any of the following: distant metastasis, ECOG performance status 3 or higher, bodyweight 30 kg or lower, pregnancy, previous invasive malignancy within 3 years (except for non-melanomatous skin cancer and early stage treated prostate cancer), previous immunotherapy or systemic therapy or radiotherapy to the head or neck region, and inadequate major organ function. Gender was self-reported by study participants (with options of male or female). Ethnicity was self-reported with predefined categories. Positive p16 status was defined as more than 70% strong nuclear or nuclear and cytoplasmic staining of tumour cells, confirmed by central pathology review. PD-L1 combined positive score (CPS) was assessed by masked central analysis of baseline tissue specimens with the 22c3 anti-PD-L1 antibody (Agilent Technologies, Santa Clara, CA, USA) following completion of the trial.

### Randomisation and masking

Eligible patients were randomly assigned (2:1) to radiotherapy with either durvalumab or cetuximab by permuted block randomisation (multiples of 6), stratified by stage (T0–3 and N0–2 *vs* T4, N3, or both), performance status and comorbidity (ECOG performance status 0 and modified CCI 0 *vs* ECOG performance status 1–2, modified CCI >0, or both), and primary site and p16 status (p16-positive oropharyngeal or unknown primary *vs* other). The rationale for the 2:1 randomisation was to incentivise participation, as many sites were using alternatives to cetuximab as their institutional standard. Investigators at each institution registered patients using an electronic system. Treatment assignment was centrally generated at the NRG Oncology Statistics and Data Management Center (Philadelphia, PA, USA) and provided to the institution when the patient was registered. No one was masked to treatment assignment.

### Procedures

Patients received intensity-modulated radiotherapy (70 Gy in 35 daily fractions over 7 weeks) with either durvalumab 1500 mg delivered intravenously beginning 2 weeks before radiotherapy then every 4 weeks beginning week 2 of radiotherapy for up to seven cycles (experimental group) or cetuximab 400 mg/m^2^ delivered intravenously 1 week before radiotherapy then 250 mg/m^2^ weekly beginning week 1 of radiotherapy for up to eight cycles (control group). All patients had pretreatment diagnostic head, neck, and chest evaluation with [^18^F]fluorodeoxyglucose ([^18^F]FDG)-PET–CT, CT, or MRI within 60 days of registration. Follow-up assessments occurred weekly during radiotherapy, every 4 weeks during adjuvant therapy, every 4 months for 1 year following radiotherapy, then every 6 months for 2 years, then annually. Whole-body PET–CT at 4 months following completion of radiotherapy for initial response evaluation was strongly encouraged. Laboratory and adverse event monitoring were conducted within 60 days of registration, within 7 days before any systemic therapy, weekly during radiotherapy, every 4 weeks during adjuvant therapy, 1 month after last dose of durvalumab, 2 and 3 months after last dose of cetuximab, every 4 months from the end of radiotherapy for 1 year, then every 6 months for 2 years, then annually. Adverse events were graded with Common Terminology Criteria for Adverse Events version 5. In the durvalumab group, adverse events were recorded regardless of relationship to protocol treatment during treatment and for 100 days from the last dose of durvalumab. In the cetuximab group, adverse events were recorded regardless of relationship to protocol treatment during treatment and for 30 days from the end of treatment. During follow-up, adverse events were recorded only if reasonably related to protocol treatment. Central review of treatment compliance was done on all patients with respect to cetuximab and durvalumab and on a random sample of patients for radiotherapy (appendix p 3). Protocol treatment was discontinued in the case of disease progression, unacceptable treatment delays and adverse events, intercurrent illness that prevented further administration of treatment, unacceptable non-compliance per investigator’s judgement, consent withdrawal, or any patient’s condition that rendered the patient unsuitable for further treatment per investigator’s judgement. If protocol treatment was discontinued, follow-up and data collection continued as specified in the protocol.

### Outcomes

The phase 2 primary endpoint was progression-free survival, defined as time from randomisation until first evidence of local, regional, or distant disease progression or recurrence, or death from any cause (assessed locally). Patients alive at the time of analysis were censored at the last follow-up (administrative censoring; all patients were potentially followed up for at least 2 years). Progressive disease was defined as clinical evidence of disease progression in a radiated field that was confirmed by cytology or histopathology or according to the Response Evaluation Criteria in Solid Tumours (RECIST) version 1.1.

Overall survival, defined as the time from randomisation to death due to any cause, was the intended phase 3 primary endpoint and is reported as a phase 2 secondary endpoint. Overall survival was not explicitly stated as a phase 2 secondary endpoint in the protocol. However, the decision to report overall survival if the progression-free survival endpoint was not met was implied as per the study protocol. The initial plan was to combine patients enrolled in phase 2 and 3 for the phase 3 primary endpoint analysis of overall survival. Protocol-specified secondary endpoints were locoregional failure, distant metastasis, competing mortality, response on [^18^F]FDG-PET–CT measured by RECIST version 1.1 at 4 months after the end of radiotherapy, acute (≤180 days after the end of radiotherapy) and late (>180 days after the end of radiotherapy) adverse events, feeding tube retention, quality-of-life endpoints including functional domain of the European Organisation for Research and Treatment of Cancer Quality of Life Questionnaire (EORTC QLQ) and swallowing quality of life using total composite MD Anderson Dysphagia Inventory (MDADI) score at 1 year from end of radiotherapy change from baseline, and translational biomarkers including PD-L1 and p16.

All clinical endpoints (locoregional failure, distant metastasis, and competing mortality) were defined from time of randomisation to failure. A locoregional failure was defined as a local or regional progression (with or without distant metastasis), death due to study cancer without documented progression, or death due to unknown causes without documented progression as first event. Of note, the protocol-specified locoregional failure definition was a combined endpoint because deaths due to unknown causes were considered failures. A distant metastasis failure was distant metastasis (without local or regional progression) as first event. A competing mortality failure was death due to second primary cancer, protocol treatment, or other cause as first event. The events of interest for each of these three endpoints were considered competing risks for the other endpoints. Prespecified secondary and exploratory endpoints of patient-reported and clinician-reported quality of life using the EORTC QLQ/HN35, EQ-5D, MDADI, and Performance Status Scale for Head and Neck Cancer Patients tools, and patient-reported toxicity measured by Patient-Reported Outcomes version of the Common Terminology Criteria for Adverse Events will be published in a separate manuscript according to the established publication plan.

### Statistical analysis

The phase 2 primary null hypothesis was that there would be no difference in progression-free survival between the experimental and control groups, and the alternative hypothesis was that there would be an improvement in progression-free survival in the experimental group compared with the control group. The phase 2 trial was designed to detect a hazard ratio (HR) of 0·65 for the experimental group relative to the control group. This HR would equate to an improvement in 2-year progression-free survival from 40·3% with cetuximab (yearly hazard rate 0·4544) to 55·4% with durvalumab (ie, median progression-free survival 1·53 years for cetuximab *vs* 2·35 years for durvalumab) under the assumption that progression-free survival follows an exponential distribution. With a one-sided α of 0·20, 80% statistical power, and using the log-rank test, a total of 69 progression-free survival events from 234 eligible randomly allocated patients was required. The α level of 0·20 was deemed adequate for a phase 2 go–no-go decision with an intermediate endpoint seeking an efficacy signal before proceeding to a definitive phase 3 trial with a primary endpoint of overall survival.^23^

Progression-free survival and overall survival were estimated using the Kaplan–Meier method, and the difference in outcomes between the groups was tested using a one-sided log-rank test including all randomly assigned patients (intention to treat). At the phase 2 final analysis, after 69 reported progression-free survival events, if the HR for the experimental versus the control group was 0·806 or less, the null hypothesis would be rejected, and the study would continue to phase 3; otherwise, the study would not continue to phase 3. HR and 95% CIs for treatment effect were estimated using Cox proportional hazard models. Locoregional failure, distant metastasis, and competing mortality were estimated by the cumulative incidence method, and groups were compared by two-sided cause-specific log-rank test at the 0·05 significance level. Cause-specific HRs and 95% CIs for locoregional failure, distant metastasis, and competing mortality were estimated using cause-specific Cox proportional hazards models. All analyses for locoregional failure, distant metastasis, and competing mortality were based on all randomly assigned patients (intention to treat). Adjusted treatment effect HRs and 95% CIs from multivariable Cox models after including the stratification factors (stage, ECOG performance status and CCI, and p16 status and tumour site) and age were also computed. The proportional hazards assumption for progression-free survival and overall survival in Cox models was tested using the Kolmogorov-type supremum test.^24^ 2-year progression-free survival and overall survival by group and a 95% CI for the difference using the Wald-type method and normal approximation were calculated, and median progression-free survival and overall survival by group using the Brookmeyer and Crowley method^25^ with 95% CIs were also estimated. Median follow-up was calculated in patients without an event.

Subgroup analyses were performed by key patient and tumour factors: age, gender, ECOG performance status, modified CCI, number of comorbidities, primary site and p16 status, T stage, N stage, smoking history, and PD-L1 CPS. All subgroup analyses were done post hoc except p16 status and PD-L1 CPS, which were prespecified (see protocol in the appendix). Treatment effect HRs and 95% CIs within subgroups were estimated using Cox models with treatment group, factor, and interaction. In addition, all clinical endpoints were analysed by primary site and p16 status due to the established prognostic relevance of this factor in patients with HNSCC. Given that deaths of unknown causes without documented progression were events in the protocol-specified locoregional failure definition, a post-hoc sensitivity analysis was performed to define an alternative endpoint (locoregional failure 2) that excluded these events. Consequently, a post-hoc sensitivity analysis was also performed to define an alternative competing mortality endpoint (competing mortality 2) that included these events.

Response on [^18^F]FDG-PET–CT was assessed by RECIST version 1.1 at 4 months after the end of radiotherapy and response rates (complete response or partial response) for all patients with [^18^F]FDG-PET–CT at both baseline and 4 months after radiotherapy were compared by two-sided Fisher’s exact test. Response at the same timepoint with any imaging technique was also performed in a post-hoc, non-protocol specified analysis. The distributions of maximum grade adverse events without regard to attribution (grade 5, 4, 3, and <3) by assigned treatment were compared by two-sided Fisher’s exact test at the 0·05 significance level. The maximum grade adverse event distributions were also compared by row mean score Cochran-Mantel-Haenszel test as a post-hoc analysis. In a post-hoc analysis, feeding tube rates at 1 year after the end of radiotherapy were compared by two-sided χ^2^ test. Adverse event analysis included only patients who started protocol therapy.

One prespecified interim futility analysis in phase 2 with 50% of information (35 progression-free survival events) was performed. If the observed hazard ratio was 1·0 or more, favouring cetuximab, then early stopping would be considered, concluding that durvalumab was not a candidate for further evaluation in phase 3. Considering the short follow-up at the time of the final prespecified phase 2 analysis, the study team decided to extend the follow-up until all randomly assigned patients completed potentially at least 2 years of follow-up to obtain stable 2-year progression-free survival estimates, which are clinically meaningful for this population.

The NRG Oncology data monitoring committee evaluated the trial biannually for accrual and safety, as well as at the protocol-specified interim analysis. All statistical analyses were done in SAS (version 9.4).

### Role of the funding source

The US National Cancer Institute (NCI) provided funding support to NRG Oncology to conduct the trial and had to approve the final protocol and every amendment. NCI had a role in study design, but not in data collection, data analysis, data interpretation, or writing of the report. AstraZeneca provided funding support to NRG Oncology to conduct the trial and contributed to the study design but had no role in data collection, data analysis, data interpretation, or writing of the report.

## Results

From Dec 12, 2017, to June 13, 2018, ten patients were enrolled in the lead-in phase (nine men and one woman; median age 67 years [IQR 65–70]; eight had oropharyngeal cancer [six p16-positive] and two had laryngeal cancer.) All ten patients had two or more contraindications to cisplatin. There were no dose-limiting toxic effects or grade 5 adverse events observed. Therefore, the experimental therapy at the starting dose met the primary safety endpoint to proceed to phase 2.

**Figure 1:**
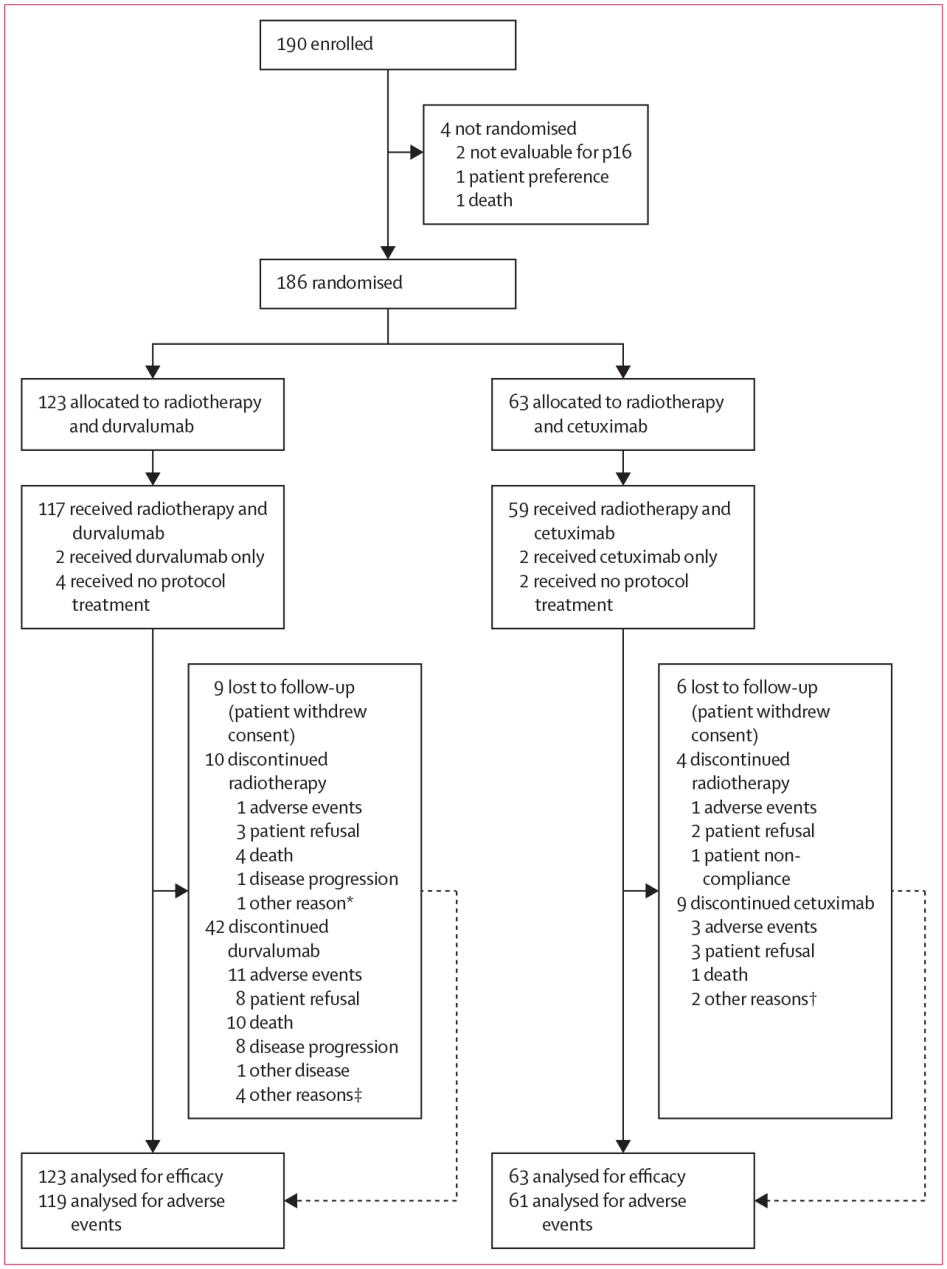
Trial profile *Discovery of liver metastasis likely to have been present before enrolment. †Missed doses could not be made up after radiotherapy per protocol. ‡One patient out of town, one physician decision, one persistent disease, and one likely metastasis before enrolment.

From March 12, 2019, to July 30, 2021, the phase 2 trial enrolled 190 patients, 186 of whom were randomly assigned (123 to durvalumab and 63 to cetuximab; figure 1). Four patients in the durvalumab group and two patients in the cetuximab group received no protocol treatment. Nine patients in the durvalumab group and six in the cetuximab group withdrew consent. Phase 2 accrual was permanently closed on Sept 1, 2022.

At a median follow-up of 6·4 months (IQR 2·2–14·2) at the interim futility analysis (data cutoff May 10, 2021), 37 patients had a progression-free survival event: 25 (22%) of 115 patients in the durvalumab group and 12 (21%) of 58 in the cetuximab group. The treatment effect HR (durvalumab *vs* cetuximab) was 1·05 (95% CI 0·53–2·09), which crossed the protocol-specified futility boundary (HR=1). As a result, given the equivocal HR estimate and the similar distribution of disease-specific events contributing to the progression-free survival endpoint, the NRG Oncology Data Monitoring Committee recommended a permanent accrual closure for this trial and additional follow-up to reach the 69 progression-free survival events required for the phase 2 primary endpoint analysis. As a result of this interim analysis, the protocol was amended to include a progression-free survival sensitivity analysis at the time of the phase 2 go–no-go decision. All patients randomly assigned to the durvalumab group with 8 or fewer weeks on study before notification to sites and their patients about the results (Sept 9, 2021) were censored at that time because they were allowed to switch to off-protocol treatment.

Median age was 72 years (IQR 64–77; table 1). Of 186 patients, 109 (59%) were aged 70 years or older. 156 (84%) of patients were men and 30 (16%) were women. The majority of patients were White (155 [83%]). 88 (47%) had p16-positive oropharyngeal or unknown primary disease, and 101 (58%) of 174 patients tested p16 positive. Of the 160 patients for whom PD-L1 CPS status was available, 127 (79%) had a score of 1 or higher, and 50 (31%) had a score of 20 or higher. Contraindications to cisplatin were hearing loss in 91 (49%) patients, renal insufficiency in 53 (28%) patients, grade 1 or higher peripheral neuropathy in 39 (21%) patients, and ECOG performance status 2 in 23 (12%) patients; 156 (84%) patients had at least one of these contraindications. 16 (9%) patients with no contraindications were aged 70 years or older with moderate or severe comorbidity, and 14 (8%) were younger than 70 years with severe comorbidity. The median number of comorbidities was 5 (IQR 4–6).

At the time of the protocol-specified analysis (data cutoff June 20, 2022), with a median follow-up of 1·2 years (IQR 0·9–2·1), 70 (38%) patients had had a progression-free survival event: 52 (42%) of 123 patients in the durvalumab group and 18 (29%) of 63 in the cetuximab group (table 2). Median progression-free survival was 2·2 years (95% CI 1·2–not reached) in the durvalumab group and 2·7 years (2·7–not reached) in the cetuximab group (HR 1·47 [95% CI 0·86–2·52]; one-sided log-rank test p=0·92). 2-year progression-free survival estimates were 51·0% (95% CI 40·7–61·2) for durvalumab and 66·4% (52·8–80·1) for cetuximab. The prespecified sensitivity analysis censoring five patients in the durvalumab group showed similar results with respect to HR and p value (table 2).

**Figure 2:**
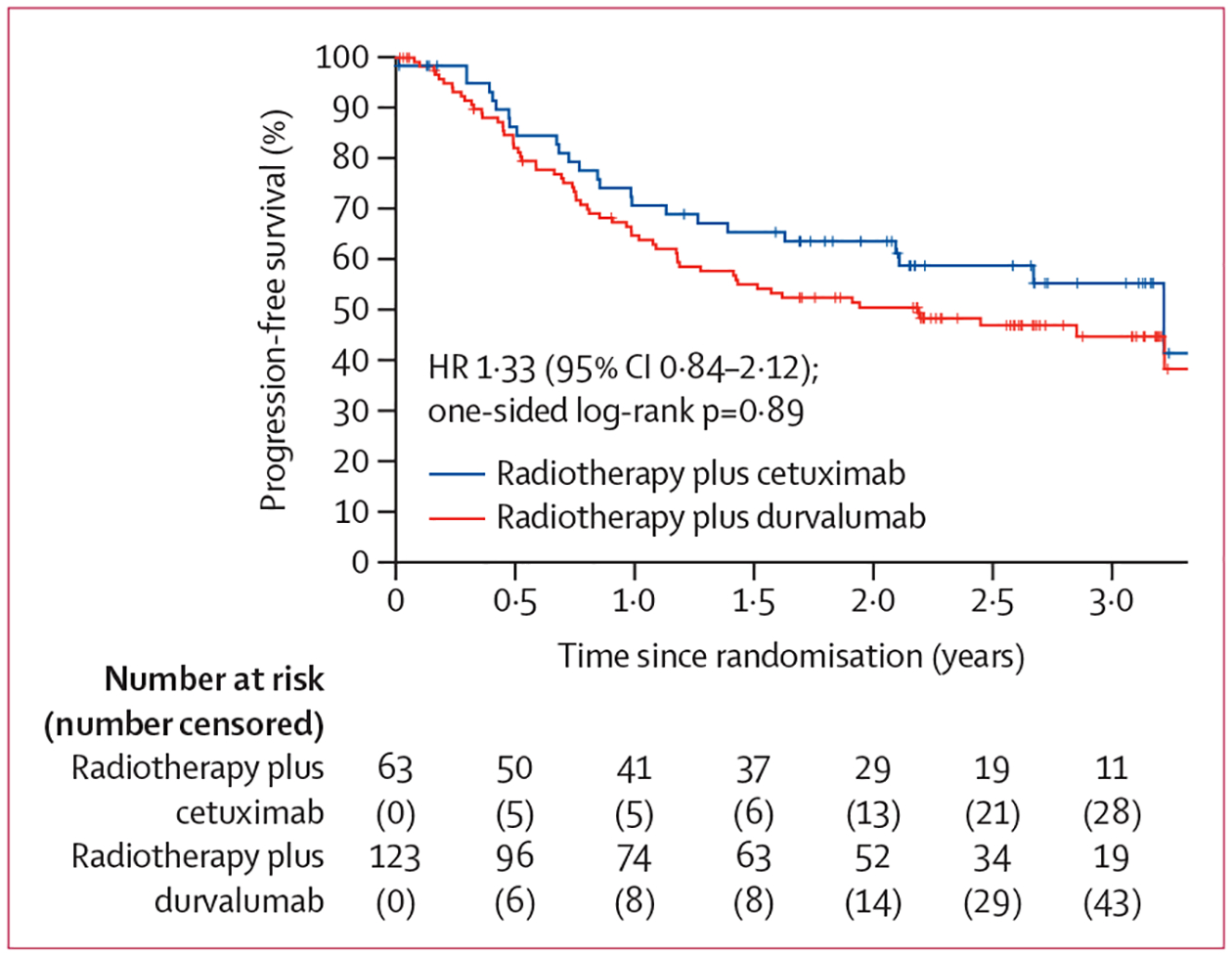
Kaplan–Meier estimates of progression-free survival in the post-hoc analysis Crosses denote censored patients.

**Figure 3:**
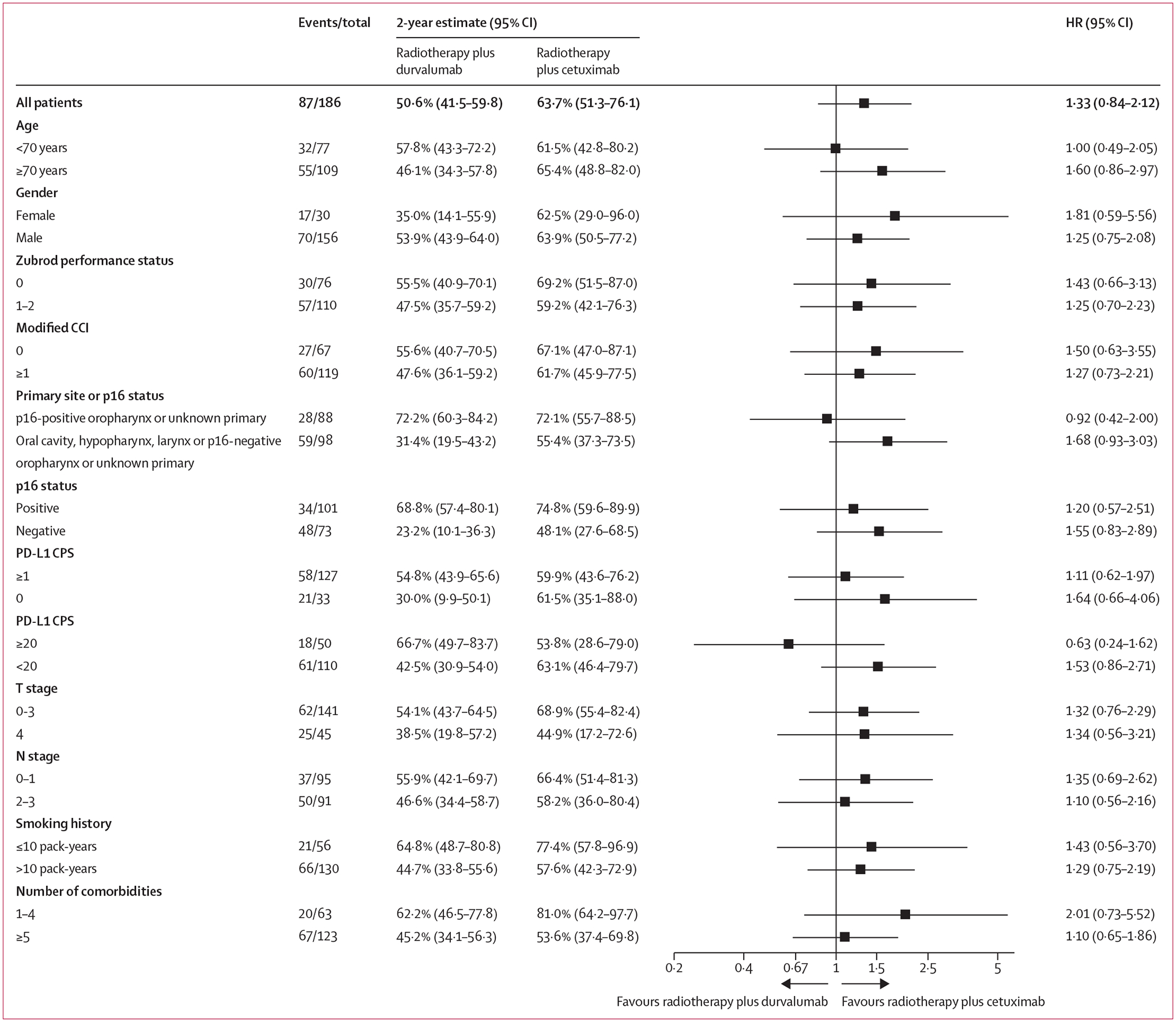
Prespecified and post-hoc subgroup analysis of progression-free survival CCI=Charlson Comorbidity Index. CPS=combined positive score.

Median follow-up at the post-hoc extended analysis (data cutoff July 31, 2023) was 2·3 years (IQR 1·9–3·1; table 2 and appendix p 3). Except for ECOG performance status and CCI (p=0·036; appendix p 4), the statistical tests to assess the proportionality assumption of progression-free survival hazards did not find serious violations. The non-proportionality of this factor was addressed by fitting a Cox model with it as a stratification factor, which resulted in similar HR estimates for the remaining covariates. 2-year progression-free survival was 50·6% (95% CI 41·5–59·8) in the durvalumab group versus 63·7% (51·3–76·1) in the cetuximab group (hazard ratio 1·33 [95% CI 0·84–2·12]; p=0·89; table 2, figure 2). The post-hoc, exploratory subgroup analysis results were consistent with the conclusion of no treatment effect of durvalumab across all subgroups (figure 3). Results of the progression-free survival analysis by p16 status and assigned treatment are shown in the appendix (p 18). The p16 status and treatment group interaction was not statistically significant (p=0·22). 2-year progression-free survival estimates in the p16-negative and p16-positive subgroups were 31·4% (95% CI 19·5–43·2) and 72·2% (60·3–84·2) in the durvalumab group and 55·4% (37·3–73·5) and 72·1% (55·7–88·5) in the cetuximab group. Similarly, PD-L1 CPS and treatment group interactions were not statistically significant (p=0·47 for CPS ≥1 and p=0·12 for CPS ≥20; appendix pp 19–20).

With extended follow-up, 58 patients had died: 41 (33%) in the durvalumab group and 17 (27%) in the cetuximab group. Post-hoc median overall survival was not reached (95% CI 2·9–not reached) in the durvalumab group and not reached (3·2–not reached) in the cetuximab group (HR 1·30 [95% CI 0·74–2·28]; one-sided log-rank p=0·82; appendix p 21). Results were similar after adjustment for stratification factors (appendix p 5). Except for primary site and p16 status (p=0·0092), the statistical tests to assess the proportionality assumption of hazards did not find serious violations (appendix p 5). Non-proportionality was addressed by fitting a Cox model with primary site and p16 status as stratification factors, which resulted in similar HR estimates for the remaining covariates (appendix p 5). Causes of death are shown in the appendix (p 6). Post-hoc 2-year overall survival estimates were 69·3% (95% CI 60·8–77·8) for durvalumab and 77·5% (66·7–88·3) for cetuximab.

Failure types for locoregional failure (locoregional failure 2), distant metastasis, and competing mortality (competing mortality 2) are in the appendix (p 6). 50 (27%) of 186 patients had locoregional failure events (38 [31%] of 123 in the durvalumab group and 12 [19%] of 63 in the cetuximab group), 22 (12%) patients had distant metastasis events (13 [11%] in the durvalumab group and nine [14%] in the cetuximab group), and 15 (8%) had competing mortality events (11 [9%] in the durvalumab group and four [6%] in the cetuximab group). At 2 years, locoregional failure estimates were 31·3% (95% CI 23·0–40·0) for durvalumab and 18·9% (10·0–29·9) for cetuximab (cause-specific HR 1·71 [95% CI 0·89–2·38], two-sided p=0·10; appendix p 22). 36 (19%) patients had locoregional failure 2 events (28 [23%] in the durvalumab group and eight [13%] in the cetuximab group) and 29 (16%) had competing mortality 2 events (21 [13%] in the durvalumab group and eight [13%] in the cetuximab group). At 2 years, locoregional failure 2 estimates were 23·5% (95% CI 16·2–31·6) for durvalumab and 13·9% (6·4–24·1) for cetuximab (cause-specific HR 1·91 [95% CI 0·87–4·19]; two-sided p=0·10; appendix p 23).

At 2 years, distant metastasis estimates were 9·5% (95% CI 5·0–15·7) for durvalumab and 12·1% (5·3–22·0) for cetuximab (cause-specific HR 0·76 [95% CI 0·32–1·77]; two-sided p=0·52; appendix p 24), and competing mortality estimates were 8·6% (95% CI 4·4–14·5) for durvalumab and 5·3% (1·4–13·4) for cetuximab (cause-specific HR 1·50 [95% CI 0·48–4·70]; two-sided p=0·49; appendix p 25). 2-year competing mortality 2 estimates were 16·4% (95% CI 10·3–23·7) for durvalumab and 10·4% (4·2–19·9) for cetuximab (cause-specific HR 1·41 [95% CI 0·62–3·18]; two-sided p=0·41; appendix p 26). Secondary outcomes by p16 status and assigned treatment are shown in the appendix (pp 27–32). Response rates at 4 months after radiotherapy assessed by [^18^F]FDG-PET–CT are in the appendix (p 7). Response rates assessed post hoc by any imaging were similar to [^18^F]FDG-PET–CT only (appendix p 8).

Adverse events are shown in table 3 and the appendix (pp 9–16). The most common grade 3–4 adverse events were dysphagia (26 [22%] of 119 patients in the durvalumab group *vs* 18 [30%] of 61 patients in the cetuximab group), lymphopenia (33 [28%] *vs* 20 [33%]), and oral mucositis (13 [11%] *vs* 11 [18%]). 11 (9%) patients in the durvalumab group and one (2%) patient in the cetuximab group died from adverse events regardless of relationship to treatment. The p value comparing the maximum grade distributions (grade 5, 4, 3, and <3) between treatment groups was 0·069. Results from the post-hoc row mean score Cochran-Mantel-Haenszel test were similar (p=0·11). Treatment-related serious adverse events were reported in 29 (24%) patients in the durvalumab group and 15 (25%) in the cetuximab group (appendix pp 15–16). The most common treatment-related serious adverse events were aspiration (three [3%] in the durvalumab group *vs* two [3%] in the cetuximab group), dehydration (five [4%] *vs* four [7%]), dysphagia (three [3%] *vs* four [7%]), dyspnoea (four [3%] *vs* one [2%]), laryngeal oedema (four [3%] *vs* none), and lung infection (one [1%] *vs* three [5%]). Four (3%) patients in the durvalumab group and one (2%) in the cetuximab group died from treatment-related adverse events (death not otherwise specified, laryngeal oedema, lung infection, and respiratory failure in the durvalumab group and sudden death not otherwise specified in the cetuximab group). One year after the end of radiotherapy, 19·0% (95% CI 12·1–28·7) of patients in the durvalumab group had a feeding tube, compared with 16·3% (8·1–30·0) in the cetuximab group (p=0·70).

Ten (5%) of 186 patients did not receive radiotherapy (six [5%] in the durvalumab group and four [6%] in the cetuximab group). 165 (89%) patients (108 [88%] in the durvalumab and 57 [90%] in the cetuximab group) received 68 Gy or more. In the durvalumab group, 84 (68%) patients received six or more doses (median 7 [IQR 5–7]). In the cetuximab group, 51 (81%) patients received seven or more doses (median 8 [7–8]). Radiotherapy compliance was scored per protocol or acceptable variation for 35 (88%) of 40 sampled patients in the durvalumab group and 26 (79%) of 33 sampled patients in the cetuximab group. Overall protocol drug compliance was per protocol or acceptable variation for 114 (93%) of 123 patients in the durvalumab group and 59 (94%) of 63 in the cetuximab group. Three (5%) of 61 patients who started durvalumab and 11 (9%) of 119 who started cetuximab discontinued due to adverse events (appendix p 17). 11 (73%) of 15 patients with disease progression in the cetuximab group and 21 (55%) of 38 in the durvalumab group had non-protocol treatment (appendix p 17).

## Discussion

The NRG-HN004 phase 2/3 trial was stopped after a planned interim futility analysis suggested that PD-L1 inhibition with durvalumab and radiotherapy was unlikely to improve outcomes over standard cetuximab with radiotherapy for patients with HNSCC and a contraindication to cisplatin. The final phase 2 results did not show a statistically significant signal of efficacy of durvalumab and radiotherapy. These findings are consistent with the results of other trials indicating a lack of benefit of immune checkpoint inhibitors compared with cetuximab in patients with HNSCC who are ineligible for cisplatin.^14,15^

Despite initial excitement for combining PD-1:PD-L1 checkpoint inhibitors with radiotherapy based on efficacy and a favourable toxicity profile in recurrent and metastatic HNSCC,^10,11^ and potential for synergy with radiotherapy,^26^ several studies have subsequently found no evidence of a benefit in patients with HNSCC with either PD-1 or PD-L1 inhibitors combined with fractionated radiotherapy as concurrent or adjuvant therapy in the definitive setting.^12–15^ Subsequent studies also have found no evidence that durvalumab is beneficial in patients with recurrent or metastatic HNSCC,^27^ despite promising early data.^16^ These results have renewed the controversy about the optimal regimen for previously untreated locoregionally advanced HNSCC when cisplatin is contraindicated. Currently, there is no clear explanation for the failure of checkpoint inhibitors to improve outcomes in randomised trials. There is some evidence of increased efficacy in patients with higher PD-L1 expression or higher CPS.^12,28^ Our findings in subgroups with CPS 20 or greater versus CPS less than 20 seem to support this; however results from analyses of small subgroup should be interpreted with caution. Sequencing of immunotherapy to follow definitive radiotherapy has not been found to improve efficacy,^29^ although many other trials are ongoing. Concurrent doublet regimens could be an efficacious strategy in patients who are unfit to receive cisplatin, particularly for regimens with known activity in HNSCC.^30^

Cetuximab, which targets EGFR, is an effective therapy for patients with HNSCC.^31^ When combined with radiotherapy in these patients, cetuximab has been found to improve overall survival over radiotherapy alone,^31^ but to be less efficacious than cisplatin for p16-positive oropharyngeal cancers.^32,33^ Cetuximab has been used frequently as an alternative radiosensitiser when cisplatin is contraindicated, despite a lack of direct evidence from randomised trials of its benefit over radiotherapy alone for those with cisplatin contraindications. Before this study, there was concern that cetuximab could even be worse than radiotherapy alone in older adults, based on an observed trend toward worse outcomes in patients older than 65 years.^31^ This, coupled with the presumption that immunotherapy would likely improve outcomes, prompted the use of a 2:1 randomisation, ostensibly to limit exposure of patients in the control group to potentially less effective therapy. Thus, although our findings leading to early study closure were surprising, they should also allay concerns about the effectiveness of cetuximab as a standard option when cisplatin is contraindicated. Interestingly, the exploratory results by p16 and CPS status suggest that the difference in the overall treatment effect is primarily driven by the p16-negative or low PD-L1 expression subgroups, which are known to be correlated.^34^ Thus, further investigation of checkpoint inhibition in the p16-positive population could be warranted, especially if this strategy could be shown to be less toxic and at least as efficacious compared with standard therapy in patients ineligible for cisplatin.

The 2-year progression-free survival estimate for radiotherapy with cetuximab in this trial (63·7%) is the highest reported to date from a multicentre controlled trial in patients ineligible for cisplatin to our knowledge. The corresponding 2-year progression-free survival from radiotherapy with cetuximab in the PembroRad trial was 39·9% and in the REACH trial was 31·0%. However, there were important differences in the populations of these trials. Although NRG-HN004 included a higher proportion of patients with p16-positive oropharyngeal squamous cell carcinoma (44%) than PembroRad (27·5%) or REACH (21·3%), it also included patients with a higher median age (72 years) than PembroRad (65 years) or REACH (67 years), and included patients with ECOG performance status 2 (13%) and excluded patients older than 70 years with a higher relative risk for cancer events. Classic contraindications to cisplatin include renal insufficiency, hearing loss, neuropathy, poor performance status, comorbidities, and older age.^3^ A novel aspect of our study was the use of validated instruments (eg, Charlson comorbidity index, ACE-27, HNCIG omega score, and CARG score) to refine the population for whom cisplatin is contraindicated. In particular, although older age (>70 years) has been associated with a lack of benefit of intensive therapy in meta-analyses,^35^ studies have found a benefit to concurrent chemotherapy in those with a higher relative risk for cancer events (ie, greater omega score).^19^

A limitation of this study, in part related to its early closure, is that we were unable to obtain robust estimates of treatment effects within subgroups due to small subsample sizes. Thus, we cannot rule out that durvalumab with radiotherapy is superior to radiotherapy alone in patients with high CPS or PD-L1 expression. Additionally, we could not determine whether p16 status influences the effectiveness of checkpoint inhibitors in this population.

The choice of the best control group for trials of cisplatin-ineligible patients with HNSCC has been actively debated. Our trial did not test radiotherapy alone, due to concerns about its strength as a control group. To the extent that durvalumab appears to be inactive for HNSCC, our findings lend credence to these concerns, since it is unlikely that radiotherapy alone would be superior to radiotherapy with durvalumab, which yielded worse outcomes compared with radiotherapy with cetuximab. In light of a randomised trial showing a survival benefit to radiotherapy with docetaxel over radiotherapy alone in patients ineligible for cisplatin,^9^ either docetaxel or cetuximab could be considered a viable treatment option for this population. Other randomised trials have found a benefit to non-cisplatin chemoradiotherapy (eg, carboplatin and fluorouracil) over radiotherapy alone,^35,36^ although these trials were conducted in populations eligible for cisplatin. Although some retrospective studies have reported superior outcomes with carboplatin-based regimens, especially carboplatin plus paclitaxel, compared with cetuximab,^6,7^ selection bias complicates estimation of treatment effects in such studies. Given our results, radiotherapy with cetuximab appears to be a good comparator and control group for new randomised trials, which are needed to help define the optimal strategy for cisplatin-ineligible patients with HNSCC.

## Supplementary Material

Appendix

## Figures and Tables

**Table 1:** Baseline characteristics of the intention-to-treat population

	Durvalumab (n=123)	Cetuximab (n=63)
**Age, years**		
Median	72 (64–77)	71 (63–77)
Range	48–88	48–90
≤49	2 (2%)	1 (2%)
50–59	10 (8%)	9 (14%)
60–69	35 (28%)	20 (32%)
70–79	60 (49%)	25 (40%)
≥80	16 (13%)	8 (13%)
**Gender**		
Men	102 (83%)	54 (86%)
Women	21 (17%)	9 (14%)
**Race**		
American Indian or Alaska Native	1 (1%)	1 (2%)
Asian	2 (2%)	1 (2%)
Black or African American	11 (9%)	6 (10%)
Native Hawaiian or other Pacific Islander	0	1 (2%)
White	105 (85%)	50 (79%)
More than one race	1 (1%)	0
Unknown or not reported	3 (2%)	4 (6%)
**Ethnicity**		
Hispanic or Latino	1 (1%)	3 (5%)
Not Hispanic or Latino	117 (95%)	56 (89%)
Unknown	5 (4%)	4 (6%)
**ECOG performance status**		
0	48 (39%)	28 (44%)
1	60 (49%)	27 (43%)
2	15 (12%)	8 (13%)
**Modified CCI** *		
0	43 (35%)	24 (38%)
≥1	80 (65%)	39 (62%)
**Stratification factor: ECOG and modified CCI**		
ECOG 0 and modified CCI 0	20 (16%)	13 (21%)
ECOG 1–2, modified CCI >0, or both	103 (84%)	50 (79%)
**Primary site**		
Oropharynx, pl6-positive	55 (45%)	27 (43%)
Unknown, pl6-positive	3 (2%)	3 (5%)
Oropharynx, p16-negative	15 (12%)	3 (5%)
Unknown, p16-negative	1 (1%)	0
Oral cavity	6 (5%)	4 (6%)
Hypopharynx	13 (11%)	8 (13%)
Larynx	30 (24%)	18 (29%)
**Stratification factor: primary site and p16 status**		
p16-positive oropharynx or unknown primary	58 (47%)	30 (48%)
pl6-negative oropharynx or unknown primary, larynx, hypopharynx, or oral cavity	65 (53%)	33 (52%)
**pl6 status** †		
pl6-negative	47/115 (41%)	26/59 (44%)
p16-positive	68/115 (59%)	33/59 (56%)
**PD-L1 combined positive score**		
Median	7·0 (1·0–32·5)	4·0 (0·5–31·0)
Range	0–100	0–100
0	20/108 (19%)	13/52 (25%)
≤1	88/108 (81%)	39/52 (75%)
1–9	41/108 (38%)	19/52 (37%)
10–19	14/108 (13%)	3/52 (6%)
20–29	5/108 (5%)	2/52 (4%)
30–39	4/108 (4%)	5/52 (10%)
40–49	1/108 (1%)	1/52 (2%)
≥50	23/108 (21%)	9/52 (17%)
**T stage (AJCC 8th edition)**		
T0	4 (3%)	3 (5%)
T1	11 (9%)	7 (11%)
T2	38 (31%)	16 (25%)
T3	42 (34%)	20 (32%)
T4	15 (12%)	11 (17%)
T4a	11 (9%)	6 (10%)
T4b	2 (2%)	0
**N stage (AJCC 8th edition)**		
N0	17 (14%)	14 (22%)
N1	36 (29%)	28 (44%)
N2	21 (17%)	8 (13%)
N2a	2 (2%)	0
N2b	21 (17%)	3 (5%)
N2c	17 (14%)	9 (14%)
N3	4 (3%)	0
N3a	2 (2%)	0
N3b	3 (2%)	1 (2%)
**Stratification factor: T and N stage**		
T0–3 and N0–2	88 (72%)	45 (71%)
T4, N3, or both	35 (28%)	18 (29%)
**Smoking history, pack-years**		
Median	29 (7–45)	20 (2–45)
Range	0–183	0–98
≤10	35 (28%)	21 (33%)
>10	88 (72%)	42 (67%)
**Comorbidity group**		
Absolute or relative contraindication to cisplatin	104 (85%)	52 (83%)
Age ≥70 years with moderate to severe comorbidity or vulnerability to cisplatin	12 (10%)	4 (6%)
Age <70 years with severe comorbidity or vulnerability to cisplatin	7 (6%)	7 (11%)
**Number of comorbidities**		
Median	5 (4–6)	5 (4–6)
Range	1–8	2–8
1	1 (1%)	0
2	5 (4%)	3 (5%)
3	10 (8%)	7 (11%)
4	23 (19%)	14 (22%)
5	31 (25%)	17 (27%)
≥6	53 (43%)	22 (35%)

Data are median (IQR), range, n (%), or n/N (%). AJCC=American Joint Committee on Cancer. CCI=Charlson Comorbidity Index. ECOG=Eastern Cooperative Oncology Group.

* Modified CCI is calculated excluding age, albumin, and cancer diagnosis.

† p16 status was assessed centrally before randomisation for patients with oropharynx or unknown primary site and at the end of the trial for other sites.

**Table 2:** Progression-free survival in the intention-to-treat population

	Durvalumab (n=123)	Cetuximab (n=63)	Difference (95% CI)*	Unadjusted HR (95% CI)	One-sided log-rank p value	Adjusted HR (95% CI)
Prespecified analysis (data cutoff June 20, 2022)						
Event	52 (42%)	18 (29%)	··	··	··	··
Censored	71 (58%)	45 (71%)	··	··	··	··
Median progression-free survival (95% CI)†, years	2·2 (1·2 to NR)	2·7 (2·7 to NR)	··	1·47 (0·86–2·52)‡	0·92	1·43 (0·84–2·45)
2-year progression-free survival (95% CI)*	51·0% (40·7 to 61·2)	66·4% (52·8 to 80·1)	−15·4% (−32·5 to 1·6)	··	··	··
Prespecified sensitivity analysis (data cutoffJune 20, 2022)	··	··	··	1·49 (0·87–2·56)	0·93	1·46 (0·85–2·50)
Post-hoc extended follow-up analysis (data cutoff July 31, 2023)						
Event	62 (50%)	25 (40%)	··	··	··	··
Censored	61 (50%)	38 (60%)	··	··	··	··
Median progression-free survival (95% CI)†, years	2·2 (1·2 to NR)	3·2 (2·1 to NR)	··	1·33 (0·84–2·12)§	0·89	1·33 (0·83–2·11)
2-year progression-free survival (95% CI)*	50·6% (41·5 to 59·8)	63·7% (51·3 to 76·1)	−13·1% (−28·5 to 2·4)	··	··	··

Differences in 2-year progression-free survival are durvalumab minus cetuximab. Hazard ratios are durvalumab versus cetuximab and were estimated by Cox proportional hazards model; exact method was used to handle ties. Adjusted analyses were done using the Cox model including stratification factors as covariates. HR=hazard ratio. NR=not reached.

* Wald-type method with Greenwood’s formula for variance.

† Brookmeyer and Crowley method.

‡ 80% lower confidence bound of 1·17.

§ 80% lower confidence bound 1·09.

**Table 3:** Adverse events by treatment group, without regard to attribution

	Durvalumab group (n=119)				Cetuximab group (n=61)			
	Grade 1–2	Grade 3	Grade 4	Grade 5	Grade 1–2	Grade 3	Grade 4	Grade 5
Overall highest grade	35 (29%)	57 (48%)	15 (13%)	11 (9%)	12 (20%)	37 (61%)	11 (18%)	1 (2%)
Acidosis	0	1 (1%)	1 (1%)	0	0	0	0	0
Alkalosis	0	0	1 (1%)	0	0	0	0	0
Anaemia	35 (29%)	8 (7%)	1 (1%)	0	15 (25%)	5 (8%)	0	0
Anorexia	51 (43%)	16 (13%)	0	0	24 (39%)	8 (13%)	0	0
Aspiration	4 (3%)	7 (6%)	0	0	2 (3%)	2 (3%)	0	0
Chronic kidney disease	3 (3%)	1 (1%)	1 (1%)	0	2 (3%)	0	0	0
Death, not otherwise specified	0	0	0	3 (3%)	0	0	0	0
Dehydration	22 (18%)	5 (4%)	0	0	10 (16%)	3 (5%)	1 (2%)	0
Dermatitis radiation	77 (65%)	6 (5%)	0	0	38 (62%)	8 (13%)	0	0
Dysphagia	72 (61%)	26 (22%)	0	0	29 (48%)	18 (30%)	0	0
Dyspnoea	35 (29%)	6 (5%)	3 (3%)	0	15 (25%)	3 (5%)	0	0
Fatigue	90 (76%)	8 (7%)	0	0	50 (82%)	2 (3%)	0	0
Heart failure	0	1 (1%)	2 (2%)	0	0	3 (5%)	0	0
Hyperglycaemia	24 (20%)	7 (6%)	0	0	13 (21%)	1 (2%)	0	0
Hypoglycaemia	3 (3%)	0	1 (1%)	0	3 (5%)	0	1 (2%)	0
Hypokalaemia	13 (11%)	5 (4%)	1 (1%)	0	6 (10%)	1 (2%)	0	0
Hyponatraemia	27 (23%)	2 (2%)	0	0	8 (13%)	0	1 (2%)	0
Hypoxia	2 (2%)	2 (2%)	1 (1%)	0	0	1 (2%)	0	0
Laryngeal oedema	5 (4%)	2 (2%)	0	1 (1%)	2 (3%)	0	0	0
Laryngeal obstruction	0	0	1 (1%)	0	0	0	0	0
Lipase increased	7 (6%)	1 (1%)	1 (1%)	0	0	0	0	0
Lung infection	5 (4%)	3 (3%)	0	2 (2%)	0	4 (7%)	0	0
Lymphopenia	18 (15%)	26 (22%)	7 (6%)	0	9 (15%)	14 (23%)	6 (10%)	0
Mucositis oral	72 (61%)	13 (11%)	0	0	36 (59%)	11 (18%)	0	0
Myocardial infarction	1 (1%)	3 (3%)	1 (1%)	0	0	1 (2%)	0	0
Neutrophil count decreased	4 (3%)	1 (1%)	1 (1%)	0	2 (3%)	0	0	0
Pericardial effusion	0	0	0	0	0	0	1 (2%)	0
Pneumonitis	2 (2%)	1 (1%)	1 (1%)	0	0	0	0	0
Pulmonary oedema	1 (1%)	1 (1%)	1 (1%)	0	0	1 (2%)	0	0
Pulmonary fistula	0	0	1 (1%)	0	0	0	0	0
Respiratory failure	0	0	4 (3%)	1 (1%)	0	0	1 (2%)	0
Sepsis	0	1 (1%)	1 (1%)	0	0	1 (2%)	0	0
Sudden death, not otherwise specified	0	0	0	4 (3%)	0	0	0	1 (2%)
Weight loss	53 (45%)	14 (12%)	0	0	27 (44%)	5 (8%)	0	0
White blood cell decreased	14 (12%)	1 (1%)	1 (1%)	0	8 (13%)	1 (2%)	0	0

Data shown are grade 3 adverse events occurring in at least 5% in either group and all grade 4–5 adverse events.

## Data Availability

Within 6 months of publication, the data from this article will be available for data sharing proposals at the NCI National Clinical Trials Network/NCI Community Oncology Research Program data archive: https://nctn-data-archive.nci.nih.gov/.

## Research in context

### Evidence before this study

We searched PubMed from Jan 1, 1998, to March 31, 2017, for randomised trials of immunotherapy, cetuximab, or chemotherapy for patients with head and neck cancer with contraindication to cisplatin, or who were unfit or ineligible for cisplatin. The search was limited to randomised trials with the search terms “cisplatin”, “unfit”, “ineligible”, “contraindication”, “contraindicated”, “cetuximab”, “immunotherapy”, “checkpoint inhibitor”, “carboplatin”, “paclitaxel”, or “docetaxel”. No language restrictions were applied. There were zero published studies at that time. Many patients with locoregionally advanced head and neck squamous cell carcinoma (HNSCC) are unfit for standard concurrent radiotherapy and cisplatin due to organ dysfunction, older age, or comorbidities. The optimal treatment strategy for this population is unknown, with poor outcomes and conflicting data for and against several alternatives to cisplatin, including cetuximab, docetaxel, and carboplatin plus paclitaxel, in combination with radiotherapy. Previous studies suggested that PD-1:PD-L1 checkpoint inhibitors are effective against HNSCC with low toxicity and can exhibit synergy with radiotherapy, making immunotherapy an attractive option to improve outcomes in older HNSCC patients with comorbidities.

### Added value of this study

This study is consistent with other trials showing no benefit of PD-1:PD-L1 checkpoint inhibitors compared with cetuximab when combined with fractionated definitive-intent radiotherapy for cisplatin-ineligible patients with HNSCC. To the best of our knowledge, we report the highest progression-free survival rate with cetuximab reported to date from a multicentre, controlled trial in cisplatin-ineligible patients with HNSCC, supporting the utility of this regimen as a standard. Novel criteria based on validated instruments were introduced to help define cisplatin ineligible patients with HNSCC.

### Implications of all the available evidence

Despite initial promise, immune checkpoint inhibition is not an effective therapeutic strategy for this overall population. Cetuximab with radiotherapy is standard treatment for patients with locoregionally advanced HNSCC who have a contraindication to cisplatin. Alternative radiosensitising strategies are needed and being actively investigated.
