# Supplementary material for: Radiotherapy with cetuximab or durvalumab for locoregionally advanced head and neck cancer in patients with a contraindication to cisplatin (NRG-HN004): an open-label, multicentre, parallel-group, randomised, phase 2/3 trial
Source: Lancet Oncol. Author manuscript; Available in PMC 2025 Jan 13. (PMC11726348; doi:10.1016/S1470-2045(24)00507-2)
Supplement: Appendix [file NIHMS2046514-supplement-Appendix.zip › HN004 Appendix 10-29-24.pdf]

APPENDIX

TABLE OF CONTENTS

**SUPPLEMENTARY METHODS & RESULTS**	**3**
**SUPPLEMENTARY TABLES**	**4-17**
**TABLE A1**	**4**
**TABLE A2**	**4**
**TABLE A3**	**5**
**TABLE A4**	**5**
**TABLE A5**	**6**
**TABLE A6**	**6**
**TABLE A7**	**7**
**TABLE A8**	**8**
**TABLE A9**	**9-14**
**TABLE A10**	**15-16**
**TABLE A11**	**17**
**TABLE A12**	**17**
**SUPPLEMENTARY FIGURES**	**18-32**
**FIGURE A1**	**18**
**FIGURE A2**	**19-20**
**FIGURE A3**	**21**
**FIGURE A4**	**22**
**FIGURE A5**	**23**
**FIGURE A6**	**24**
**FIGURE A7**	**25**
**FIGURE A8**	**26**
**FIGURE A9**	**27**
**FIGURE A10**	**28**
**FIGURE A11**	**29**
**FIGURE A12**	**30**
**FIGURE A13**	**31**
**FIGURE A14**	**32**
**SITES, INVESTIGATORS, AND NUMBER OF PATIENTS RECRUITED**	**33-34**
**NRG-HN004 PROTOCOL**	**35-159**

SUPPLEMENTARY METHODS & RESULTS

Lead-in study design

The lead-in study included 3 dose levels to de-escalate durvalumab in the concurrent and maintenance cycles. The primary endpoint of the safety run-in was dose-limiting toxicity (DLT) of RT with durvalumab. A DLT was defined as any grade ≥ 3 adverse event (AE), or delay in RT > 2 weeks or inability to complete RT, definitely or probably related to durvalumab, occurring from week -2 up to 4 weeks following RT, with exclusions for expected grade 3-4 toxicities. Toxicity was assessed using Common Terminology Criteria for Adverse Events (CTCAE) version 4.0. A dose level was declared safe if 0-2 DLTs were observed in 8 evaluable patients. All patients who receive at least one dose of durvalumab are considered evaluable for DLT, should a DLT occur. In the absence of DLT, patients must have received at least one dose of durvalumab and one fraction of radiation therapy and have completed the DLT observation period to be evaluable for DLT. With 8 patients, if the true DLT rate was ≤ 20%, the probability of the therapy being deemed safe would be ≥ 80%.

Lead-in study results

Ten patients were enrolled from December 12, 2017 to June 13, 2018 (9 men and 1 woman; median age 67 [IQR: 65-70] years; 8 had oropharyngeal cancer (6 p16-positive), 2 had laryngeal cancer.) All 10 patients had ≥ 2 contraindications to cisplatin. All 10 patients completed RT, and 9 of 10 patients received all 7 doses of durvalumab. One patient received 2 doses then discontinued due to diarrhea possibly related to durvalumab. This patient underwent a steroid taper followed by colonoscopy with biopsy that did not show evidence of immune colitis. There were no DLTs or grade 5 AEs observed. One grade 4 AE was reported (lymphocyte count decreased, unlikely related to durvalumab). One grade 3 AE was rated definitely related to durvalumab: lymphocyte count decreased. Grade 3 AEs possibly related to durvalumab were: diarrhea (n=1), nausea (1), vomiting (1), lymphocyte count decreased (1). The experimental therapy at the starting dose level met its primary safety endpoint to proceed to phase II.

Protocol-Specified Phase II Sensitivity Analysis

When phase II accrual was suspended, some patients still under RT+durvalumab treatment were allowed to switch to off-protocol treatment after releasing the Dear Investigator Letter/Dear Patient Letter. Therefore, a key sensitivity analysis to the protocol-specified phase II primary analysis was performed. Patients randomized to the RT+durvalumab arm eight weeks or less before the date of release of the DP/DI letters (8/9/2021) were censored at this time. All the remaining patients receiving RT+durvalumab at the letter release time were included in the phase II final analysis without this censoring type.

Treatment compliance reviews

Since the radiation therapy was not investigational in this trial, reviews were done on a random sample of randomized patients. Approximately 31% of all randomized patients were randomly chosen for review by study chairs. The sample selection followed a systematic random sample within treatment arm throughout the entire accrual period. Reviews of cetuximab and durvalumab were done by study chairs on all patients since durvalumab was investigational.

SUPPLEMENTARY TABLES

Table A1: Cox proportional hazards models for progression-free survival (n=186; 87 events)

Variable	Univariate models HR (95% CI)	Univariate models p-value[1]	Multivariable model #1 HR (95% CI)	Multivariable model #1 p-value[1]	Multivariable model #2 HR (95% CI)	Multivariable model #2 p-value[1]
RT+Durvalumab vs. RT+Cetuximab	1·33 (0·84-2·12)	0·2263	1·33 (0·83-2·11)	0·2331	1·30 (0·81-2·08)	0·2724
T4 and/or N3 vs. T0-3 and N0-2	1·76 (1·13-2·75)	0·0121	1·92 (1·23-3·01)	0·0043	1·88 (1·20-2·95)	0·0062
Zubrod PS 1-2 and/or CCI > 0 vs. Zubrod PS 0 and CCI 0	2·26 (1·13-4·50)	0·0210	2·12 (1·06-4·23)	0·0338	2·15 (1·07-4·30)	0·0307
p16-negative OPC/CUP or LC/HPC/OCC vs. p16-positive OPC/CUP	2·66 (1·70-4·19)	<·0001	2·81 (1·78-4·44)	<·0001	2·85 (1·80-4·50)	<·0001
Age ≥ 70 years vs. < 70 years	1·24 (0·80-1·92)	0·3323	--	--	1·19 (0·76-1·86)	0·4396

HR, hazard ratio; CI, confidence interval; PS, performance status; OPC, oropharyngeal cancer;
CUP, cancer of unknown primary; LC, laryngeal cancer; HPC, hypopharyngeal cancer; OCC, oral cavity cancer;
CCI, modified Charlson Comorbidity Index calculated by excluding age, albumin, and cancer diagnosis.
[1] Chi-square test.

Table A2: P values for Kolmogorov-type supremum test with 10,000 simulated paths of cumulative sums of martingale residuals over follow-up times to test proportional hazard assumption of Cox models for progression-free survival (n=186; 87 events)

Predictor	Univariate models	Multivariable model #1	Multivariable model #2
**Assigned treatment**	0·9082	0·9436	0·9452
**T and N stage**	0·5865	0·6177	0·6208
**Zubrod PS and CCI**	0·0497	0·0355	0·0356
**Primary site and p16 status**	0·1815	0·2369	0·2328
**Age**	0·6972	--	0·6899

PS, performance status; CCI, modified Charlson Comorbidity Index calculated by excluding age, albumin, and cancer diagnosis.

Table A3: Cox proportional hazards models for overall survival (n=186; 58 events)

Variable	Univariate models HR (95% CI)	Univariate models p-value[1]	Multivariable model #1 HR (95% CI)	Multivariable model #1 p-value[1]	Multivariable model #2 HR (95% CI)	Multivariable model #2 p-value[1]
RT+Durvalumab vs. RT+Cetuximab	1·30 (0·74-2·28)	0·3670	1·28 (0·73-2·26)	0·3930	1·22 (0·69-2·16)	0·5016
T4 and/or N3 vs. T0-3 and N0-2	1·87 (1·09-3·19)	0·0231	1·97 (1·15-3·39)	0·0140	1·92 (1·12-3·31)	0·0186
Zubrod PS 1-2 and/or CCI > 0 vs. Zubrod PS 0 and CCI 0	2·59 (1·04-6·49)	0·0416	2·37 (0·95-5·95)	0·0649	2·46 (0·98-6·17)	0·0552
p16-negative OPC/CUP or LC/HPC/OCC vs. p16-positive OPC/CUP	2·31 (1·34-4·01)	0·0028	2·39 (1·37-4·15)	0·0020	2·47 (1·42-4·31)	0·0014
Age ≥ 70 years vs. < 70 years	1·41 (0·82-2·43)	0·2122	--	--	1·43 (0·82-2·48)	0·2036

HR, hazard ratio; CI, confidence interval; PS, performance status; OPC, oropharyngeal cancer;
CUP, cancer of unknown primary; LC, laryngeal cancer; HPC, hypopharyngeal cancer; OCC, oral cavity cancer;
CCI, modified Charlson Comorbidity Index calculated by excluding age, albumin, and cancer diagnosis.
[1] Chi-square test.

Table A4: P values for Kolmogorov-type supremum test with 10,000 simulated paths of cumulative sums of martingale residuals over follow-up times to test proportional hazard assumption of Cox models for overall survival (n=186; 58 events)

Predictor	Univariate models	Multivariable model #1	Multivariable model #2
**Assigned treatment**	0·8404	0·8935	0·8874
**T and N stage**	0·2630	0·2380	0·2433
**Zubrod PS and CCI**	0·4418	0·4151	0·4212
**Primary site and p16 status**	0·0092	0·0105	0·0105
**Age**	0·6544	--	0·6426

PS, performance status; CCI, modified Charlson Comorbidity Index calculated by excluding age, albumin, and cancer diagnosis.

Table A5: Causes of death by treatment arm

	RT+Cetuximab (n=17)	RT+Durvalumab (n=41)	Total (n=58)
Due to this disease	8 (47%)	15 (37%)	23 (40%)
Due to second primary or other malignancy	1 (6%)	0 (0%)	1 (2%)
Due to protocol treatment	0 (0%)	2 (5%)	2 (3%)
Due to other cause	3 (18%)	11 (27%)	14 (24%)
Unknown	5 (29%)	13 (32%)	18 (31%)

Table A6: Failure types (first event) by treatment arm

No.	Event Type	Cetuximab (n=25)	Durvalumab (n=62)	Total (n=87)
I	Locoregional progression/recurrence	5 (20%)	24 (39%)	29 (33%)
II	Distant metastasis	9 (36%)	13 (21%)	22 (25%)
III	Locoregional and distant	1 (4%)	1 (2%)	2 (2%)
IV	Death from this disease, failure site unspecified	2 (8%)	3 (5%)	5 (6%)
V	Death from second primary malignancy	1 (4%)	0 (0%)	1 (1%)
VI	Death from protocol treatment	0 (0%)	2 (3%)	2 (2%)
VII	Death from other cause	3 (12%)	9 (15%)	12 (14%)
VIII	Death from unknown cause	4 (16%)	10 (16%)	14 (16%)

Event types I, III, IV, and VIII were considered failures for the locoregional failure (LRF) endpoint. Event type II was considered a failure for the distant metastasis (DM) endpoint. Event types V, VI, and VII were considered failures for the competing mortality (CM) endpoint. For the alternative (non-protocol specified) definitions of LRF and CM, namely, LRF2 and CM2, event types I, III, and IV were considered failures for LRF2, and V, VI, VII, and VIII for CM2.

Table A7: Response by FDG-PET/CT at 4 Months from End of RT

	Cetuximab (n=22)	Durvalumab (n=29)	p-value [1]
Response - evaluable patients			
CR	12 (55%)	14 (48%)	
PR	4 (18%)	8 (28%)	
SD	3 (14%)	3 (10%)	
PD	3 (14%)	4 (14%)	
CR+PR / total evaluable	16 / 22	22 / 29	1·0000
Response rate	72·7%	75·9%	
95% confidence interval (Wilson score)	51·8-86·8%	57·9-87·8%	
Timing of assessment (months from end of RT) - evaluable patients			
Median	3·8	3·9	
Min - Max	2·5 – 4·8	3·0 – 4·7	
Q1 - Q3	3·4 – 4·1	3·2 – 4·1	

Response was measured with RECIST version 1.1.
CR, complete response; PR, partial response; SD, stable disease; PD, progressive disease;
Q1, first quartile; Q3, third quartile.
[1] Fisher's exact test.

Table A8: Response by Any Imaging at 4 Months from End of RT

	Cetuximab (n=63)	Durvalumab (n=123)	p-value [1]
Response - all patients			
CR	17 (27%)	32 (26%)	
PR	12 (19%)	22 (18%)	
SD	6 (10%)	8 (7%)	
PD	4 (6%)	13 (11%)	
NE, died prior to time point	4 (6%)	12 (10%)	
NE, censored prior to time point	5 (8%)	8 (7%)	
NE, no assessment at this time point	15 (24%)	28 (23%)	
Response - evaluable patients			
CR	17/39 (44%)	32/75 (43%)	
PR	12/39 (31%)	22/75 (29%)	
SD	6/39 (15%)	8/75 (11%)	
PD	4/39 (10%)	13/75 (17%)	
CR+PR / total evaluable	29 / 39	54 / 75	0·83
Response rate	74·4%	72·0%	
95% confidence interval (Wilson score)	58·9-85·4%	61·0-80·9%	
Method of assessment - evaluable patients			
CT scan	16/39 (41%)	41/75 (55%)	
MRI scan	1/39 (3%)	4/75 (5%)	
PET/CT scan	22/39 (56%)	29/75 (39%)	
Unknown	0/39 (0%)	1/75 (1%)	
Timing of assessment (months from end of RT) - evaluable patients	(n=39)	(n=75)	
Median	3·8	3·9	
Min - Max	2·5 – 5·9	2·1 – 4·9	
Q1 - Q3	3·4 – 4·0	3·4 – 4·2	

Response was measured with RECIST version 1.1.
CR, complete response; PR, partial response; SD, stable disease; PD, progressive disease; NE, not evaluable;
Q1, first quartile; Q3, third quartile.
[1] Fisher's exact test.

Table A9: Adverse events by treatment arm, without regard to attribution (Complete)

Term	Cetuximab (n=61) n (%) by grade				Durvalumab (n=119) n (%) by grade			
	1-2	3	4	5	1-2	3	4	5
Overall highest grade	12 (20)	37 (61)	11 (18)	1 (2)	35 (29)	57 (48)	15 (13)	11 (9)
Abdominal pain	12 (20)	0 (0)	0 (0)	0 (0)	17 (14)	1 (1)	0 (0)	0 (0)
Acidosis	0 (0)	0 (0)	0 (0)	0 (0)	0 (0)	1 (1)	1 (1)	0 (0)
Acute kidney injury	0 (0)	1 (2)	0 (0)	0 (0)	0 (0)	4 (3)	0 (0)	0 (0)
Agitation	0 (0)	0 (0)	0 (0)	0 (0)	1 (1)	1 (1)	0 (0)	0 (0)
Alanine aminotransferase increased	6 (10)	0 (0)	0 (0)	0 (0)	12 (10)	3 (3)	0 (0)	0 (0)
Alkaline phosphatase increased	4 (7)	0 (0)	0 (0)	0 (0)	11 (9)	1 (1)	0 (0)	0 (0)
Alkalosis	0 (0)	0 (0)	0 (0)	0 (0)	0 (0)	0 (0)	1 (1)	0 (0)
Allergic reaction	1 (2)	1 (2)	0 (0)	0 (0)	1 (1)	0 (0)	0 (0)	0 (0)
Anal pain	1 (2)	0 (0)	0 (0)	0 (0)	0 (0)	1 (1)	0 (0)	0 (0)
Anaphylaxis	0 (0)	1 (2)	0 (0)	0 (0)	0 (0)	0 (0)	0 (0)	0 (0)
Anemia	15 (25)	5 (8)	0 (0)	0 (0)	35 (29)	8 (7)	1 (1)	0 (0)
Anorexia	24 (39)	8 (13)	0 (0)	0 (0)	51 (43)	16 (13)	0 (0)	0 (0)
Anxiety	5 (8)	0 (0)	0 (0)	0 (0)	9 (8)	1 (1)	0 (0)	0 (0)
Aphonia	0 (0)	0 (0)	0 (0)	0 (0)	0 (0)	1 (1)	0 (0)	0 (0)
Arthralgia	4 (7)	0 (0)	0 (0)	0 (0)	13 (11)	0 (0)	0 (0)	0 (0)
Arthritis	3 (5)	1 (2)	0 (0)	0 (0)	5 (4)	0 (0)	0 (0)	0 (0)
Aspartate aminotransferase increased	9 (15)	0 (0)	0 (0)	0 (0)	17 (14)	4 (3)	0 (0)	0 (0)
Aspiration	2 (3)	2 (3)	0 (0)	0 (0)	4 (3)	7 (6)	0 (0)	0 (0)
Atrial fibrillation	2 (3)	2 (3)	0 (0)	0 (0)	1 (1)	1 (1)	0 (0)	0 (0)
Atrial flutter	0 (0)	1 (2)	0 (0)	0 (0)	0 (0)	0 (0)	0 (0)	0 (0)
Blood bilirubin increased	3 (5)	0 (0)	0 (0)	0 (0)	6 (5)	1 (1)	0 (0)	0 (0)
Bone infection	0 (0)	1 (2)	0 (0)	0 (0)	0 (0)	0 (0)	0 (0)	0 (0)
CPK increased	0 (0)	0 (0)	0 (0)	0 (0)	0 (0)	1 (1)	0 (0)	0 (0)
Cardiac disorders - Other	1 (2)	1 (2)	0 (0)	0 (0)	1 (1)	0 (0)	0 (0)	0 (0)
Cardiac troponin I increased	2 (3)	0 (0)	0 (0)	0 (0)	4 (3)	1 (1)	0 (0)	0 (0)
Catheter related infection	0 (0)	1 (2)	0 (0)	0 (0)	0 (0)	0 (0)	0 (0)	0 (0)
Cheilitis	0 (0)	1 (2)	0 (0)	0 (0)	0 (0)	0 (0)	0 (0)	0 (0)
Chest pain - cardiac	0 (0)	1 (2)	0 (0)	0 (0)	1 (1)	1 (1)	0 (0)	0 (0)
Chronic kidney disease	2 (3)	0 (0)	0 (0)	0 (0)	3 (3)	1 (1)	1 (1)	0 (0)
Cognitive disturbance	0	0	0	0	1	1	0	0

Table A9: Adverse events by treatment arm, without regard to attribution (Complete)

Term	Cetuximab (n=61)				Durvalumab (n=119)			
	n (%) by grade				n (%) by grade			
	1-2	3	4	5	1-2	3	4	5
	(0)	(0)	(0)	(0)	(1)	(1)	(0)	(0)
Constipation	25 (41)	0 (0)	0 (0)	0 (0)	37 (31)	3 (3)	0 (0)	0 (0)
Cough	28 (46)	0 (0)	0 (0)	0 (0)	54 (45)	0 (0)	0 (0)	0 (0)
Creatinine increased	11 (18)	0 (0)	0 (0)	0 (0)	23 (19)	2 (2)	0 (0)	0 (0)
Death NOS	0 (0)	0 (0)	0 (0)	0 (0)	0 (0)	0 (0)	0 (0)	3 (3)
Dehydration	10 (16)	3 (5)	1 (2)	0 (0)	22 (18)	5 (4)	0 (0)	0 (0)
Delirium	0 (0)	1 (2)	0 (0)	0 (0)	0 (0)	1 (1)	0 (0)	0 (0)
Depressed level of consciousness	0 (0)	0 (0)	0 (0)	0 (0)	0 (0)	3 (3)	0 (0)	0 (0)
Dermatitis radiation	38 (62)	8 (13)	0 (0)	0 (0)	77 (65)	6 (5)	0 (0)	0 (0)
Diarrhea	17 (28)	0 (0)	0 (0)	0 (0)	29 (24)	3 (3)	0 (0)	0 (0)
Dizziness	4 (7)	0 (0)	0 (0)	0 (0)	22 (18)	1 (1)	0 (0)	0 (0)
Dry mouth	35 (57)	2 (3)	0 (0)	0 (0)	82 (69)	2 (2)	0 (0)	0 (0)
Dry skin	8 (13)	0 (0)	0 (0)	0 (0)	13 (11)	0 (0)	0 (0)	0 (0)
Dysgeusia	35 (57)	0 (0)	0 (0)	0 (0)	73 (61)	0 (0)	0 (0)	0 (0)
Dysphagia	29 (48)	18 (30)	0 (0)	0 (0)	72 (61)	26 (22)	0 (0)	0 (0)
Dyspnea	15 (25)	3 (5)	0 (0)	0 (0)	35 (29)	6 (5)	3 (3)	0 (0)
Ear pain	7 (11)	0 (0)	0 (0)	0 (0)	12 (10)	1 (1)	0 (0)	0 (0)
Edema limbs	14 (23)	0 (0)	0 (0)	0 (0)	28 (24)	1 (1)	0 (0)	0 (0)
Ejection fraction decreased	0 (0)	0 (0)	0 (0)	0 (0)	0 (0)	1 (1)	0 (0)	0 (0)
Electrocardiogram QT corrected interval prolonged	0 (0)	0 (0)	0 (0)	0 (0)	0 (0)	1 (1)	0 (0)	0 (0)
Enterocolitis infectious	0 (0)	0 (0)	0 (0)	0 (0)	0 (0)	1 (1)	0 (0)	0 (0)
Epistaxis	3 (5)	1 (2)	0 (0)	0 (0)	1 (1)	0 (0)	0 (0)	0 (0)
Esophageal pain	2 (3)	0 (0)	0 (0)	0 (0)	1 (1)	1 (1)	0 (0)	0 (0)
Esophagitis	0 (0)	0 (0)	0 (0)	0 (0)	6 (5)	1 (1)	0 (0)	0 (0)
Fall	2 (3)	0 (0)	0 (0)	0 (0)	8 (7)	2 (2)	0 (0)	0 (0)
Fatigue	50 (82)	2 (3)	0 (0)	0 (0)	90 (76)	8 (7)	0 (0)	0 (0)
Febrile neutropenia	0 (0)	0 (0)	0 (0)	0 (0)	0 (0)	1 (1)	0 (0)	0 (0)
Fever	4 (7)	0 (0)	0 (0)	0 (0)	6 (5)	1 (1)	0 (0)	0 (0)
Fracture	1 (2)	2 (3)	0 (0)	0 (0)	2 (2)	0 (0)	0 (0)	0 (0)
Gastroesophageal reflux disease	7 (11)	0 (0)	0 (0)	0 (0)	6 (5)	0 (0)	0 (0)	0 (0)
Gastrointestinal disorders - Other	7 (11)	1 (2)	0 (0)	0 (0)	14 (12)	1 (1)	0 (0)	0 (0)
General disorders and administration site conditions - Other	6 (10)	0 (0)	0 (0)	0 (0)	1 (1)	1 (1)	0 (0)	0 (0)

Table A9: Adverse events by treatment arm, without regard to attribution (Complete)

Term	Cetuximab (n=61) n (%) by grade				Durvalumab (n=119) n (%) by grade			
	1-2	3	4	5	1-2	3	4	5
Generalized muscle weakness	3 (5)	2 (3)	0 (0)	0 (0)	5 (4)	1 (1)	0 (0)	0 (0)
Headache	13 (21)	0 (0)	0 (0)	0 (0)	11 (9)	1 (1)	0 (0)	0 (0)
Hearing impaired	3 (5)	1 (2)	0 (0)	0 (0)	6 (5)	0 (0)	0 (0)	0 (0)
Heart failure	0 (0)	3 (5)	0 (0)	0 (0)	0 (0)	1 (1)	2 (2)	0 (0)
Hematoma	1 (2)	1 (2)	0 (0)	0 (0)	0 (0)	0 (0)	0 (0)	0 (0)
Hepatic failure	0 (0)	0 (0)	0 (0)	0 (0)	0 (0)	1 (1)	0 (0)	0 (0)
Hip fracture	0 (0)	0 (0)	0 (0)	0 (0)	0 (0)	1 (1)	0 (0)	0 (0)
Hoarseness	14 (23)	0 (0)	0 (0)	0 (0)	18 (15)	1 (1)	0 (0)	0 (0)
Hyperglycemia	13 (21)	1 (2)	0 (0)	0 (0)	24 (20)	7 (6)	0 (0)	0 (0)
Hyperkalemia	3 (5)	1 (2)	0 (0)	0 (0)	6 (5)	0 (0)	0 (0)	0 (0)
Hypermagnesemia	0 (0)	0 (0)	0 (0)	0 (0)	4 (3)	1 (1)	0 (0)	0 (0)
Hypernatremia	4 (7)	0 (0)	0 (0)	0 (0)	1 (1)	1 (1)	0 (0)	0 (0)
Hyperphosphatemia	0 (0)	0 (0)	0 (0)	0 (0)	0 (0)	1 (1)	0 (0)	0 (0)
Hypertension	11 (18)	3 (5)	0 (0)	0 (0)	14 (12)	4 (3)	0 (0)	0 (0)
Hypoalbuminemia	16 (26)	0 (0)	0 (0)	0 (0)	21 (18)	2 (2)	0 (0)	0 (0)
Hypocalcemia	7 (11)	0 (0)	0 (0)	0 (0)	15 (13)	0 (0)	0 (0)	0 (0)
Hypoglycemia	3 (5)	0 (0)	1 (2)	0 (0)	3 (3)	0 (0)	1 (1)	0 (0)
Hypokalemia	6 (10)	1 (2)	0 (0)	0 (0)	13 (11)	5 (4)	1 (1)	0 (0)
Hypomagnesemia	20 (33)	0 (0)	0 (0)	0 (0)	21 (18)	0 (0)	0 (0)	0 (0)
Hyponatremia	8 (13)	0 (0)	1 (2)	0 (0)	27 (23)	2 (2)	0 (0)	0 (0)
Hypotension	8 (13)	0 (0)	0 (0)	0 (0)	12 (10)	2 (2)	0 (0)	0 (0)
Hypothyroidism	7 (11)	0 (0)	0 (0)	0 (0)	25 (21)	0 (0)	0 (0)	0 (0)
Hypoxia	0 (0)	1 (2)	0 (0)	0 (0)	2 (2)	2 (2)	1 (1)	0 (0)
INR increased	0 (0)	0 (0)	0 (0)	0 (0)	5 (4)	1 (1)	0 (0)	0 (0)
Ileal obstruction	0 (0)	1 (2)	0 (0)	0 (0)	0 (0)	0 (0)	0 (0)	0 (0)
Infections and infestations - Other	2 (3)	1 (2)	0 (0)	0 (0)	6 (5)	1 (1)	0 (0)	0 (0)
Injury to carotid artery	0 (0)	0 (0)	0 (0)	0 (0)	0 (0)	1 (1)	0 (0)	0 (0)
Injury, poisoning and procedural complications - Other	1 (2)	0 (0)	0 (0)	0 (0)	2 (2)	3 (3)	0 (0)	0 (0)
Insomnia	9 (15)	0 (0)	0 (0)	0 (0)	10 (8)	1 (1)	0 (0)	0 (0)
Laryngeal edema	2 (3)	0 (0)	0 (0)	0 (0)	5 (4)	2 (2)	0 (0)	1 (1)
Laryngeal mucositis	1 (2)	0 (0)	0 (0)	0 (0)	0 (0)	1 (1)	0 (0)	0 (0)
Laryngeal obstruction	0	0	0	0	0	0	1	0

Table A9: Adverse events by treatment arm, without regard to attribution (Complete)

Term	Cetuximab (n=61)				Durvalumab (n=119)			
	n (%) by grade				n (%) by grade			
	1-2	3	4	5	1-2	3	4	5
	(0)	(0)	(0)	(0)	(0)	(0)	(1)	(0)
Laryngeal stenosis	0 (0)	0 (0)	0 (0)	0 (0)	0 (0)	1 (1)	0 (0)	0 (0)
Leukocytosis	0 (0)	0 (0)	0 (0)	0 (0)	0 (0)	1 (1)	0 (0)	0 (0)
Lipase increased	0 (0)	0 (0)	0 (0)	0 (0)	7 (6)	1 (1)	1 (1)	0 (0)
Localized edema	1 (2)	0 (0)	0 (0)	0 (0)	4 (3)	1 (1)	0 (0)	0 (0)
Lung infection	0 (0)	4 (7)	0 (0)	0 (0)	5 (4)	3 (3)	0 (0)	2 (2)
Lymphedema	13 (21)	0 (0)	0 (0)	0 (0)	17 (14)	0 (0)	0 (0)	0 (0)
Lymphocyte count decreased	9 (15)	14 (23)	6 (10)	0 (0)	18 (15)	26 (22)	7 (6)	0 (0)
Mucositis oral	36 (59)	11 (18)	0 (0)	0 (0)	72 (61)	13 (11)	0 (0)	0 (0)
Musculoskeletal and connective tissue disorder - Other	1 (2)	0 (0)	0 (0)	0 (0)	5 (4)	1 (1)	0 (0)	0 (0)
Myalgia	9 (15)	0 (0)	0 (0)	0 (0)	24 (20)	1 (1)	0 (0)	0 (0)
Myasthenia gravis	0 (0)	0 (0)	0 (0)	0 (0)	0 (0)	1 (1)	0 (0)	0 (0)
Myocardial infarction	0 (0)	1 (2)	0 (0)	0 (0)	1 (1)	3 (3)	1 (1)	0 (0)
Nausea	34 (56)	1 (2)	0 (0)	0 (0)	49 (41)	4 (3)	0 (0)	0 (0)
Neck edema	2 (3)	0 (0)	0 (0)	0 (0)	7 (6)	1 (1)	0 (0)	0 (0)
Neck pain	7 (11)	1 (2)	0 (0)	0 (0)	13 (11)	3 (3)	0 (0)	0 (0)
Nervous system disorders - Other	0 (0)	0 (0)	0 (0)	0 (0)	1 (1)	1 (1)	0 (0)	0 (0)
Neutrophil count decreased	2 (3)	0 (0)	0 (0)	0 (0)	4 (3)	1 (1)	1 (1)	0 (0)
Obesity	0 (0)	1 (2)	0 (0)	0 (0)	0 (0)	0 (0)	0 (0)	0 (0)
Oral hemorrhage	0 (0)	2 (3)	0 (0)	0 (0)	1 (1)	2 (2)	0 (0)	0 (0)
Oral pain	8 (13)	2 (3)	0 (0)	0 (0)	6 (5)	0 (0)	0 (0)	0 (0)
Oropharyngeal pain	4 (7)	0 (0)	0 (0)	0 (0)	6 (5)	1 (1)	0 (0)	0 (0)
Pain	8 (13)	0 (0)	0 (0)	0 (0)	19 (16)	1 (1)	0 (0)	0 (0)
Pericardial effusion	0 (0)	0 (0)	1 (2)	0 (0)	0 (0)	0 (0)	0 (0)	0 (0)
Peripheral sensory neuropathy	5 (8)	0 (0)	0 (0)	0 (0)	12 (10)	0 (0)	0 (0)	0 (0)
Pharyngeal hemorrhage	1 (2)	1 (2)	0 (0)	0 (0)	0 (0)	0 (0)	0 (0)	0 (0)
Platelet count decreased	5 (8)	0 (0)	0 (0)	0 (0)	16 (13)	0 (0)	0 (0)	0 (0)
Pleural effusion	2 (3)	1 (2)	0 (0)	0 (0)	3 (3)	0 (0)	0 (0)	0 (0)
Pneumonitis	0 (0)	0 (0)	0 (0)	0 (0)	2 (2)	1 (1)	1 (1)	0 (0)
Pneumothorax	0 (0)	0 (0)	0 (0)	0 (0)	0 (0)	2 (2)	0 (0)	0 (0)
Pruritus	23 (38)	0 (0)	0 (0)	0 (0)	31 (26)	0 (0)	0 (0)	0 (0)
Pulmonary edema	0 (0)	1 (2)	0 (0)	0 (0)	1 (1)	1 (1)	1 (1)	0 (0)

Table A9: Adverse events by treatment arm, without regard to attribution (Complete)

Term	Cetuximab (n=61) n (%) by grade				Durvalumab (n=119) n (%) by grade			
	1-2	3	4	5	1-2	3	4	5
Pulmonary fistula	0 (0)	0 (0)	0 (0)	0 (0)	0 (0)	0 (0)	1 (1)	0 (0)
Rash acneiform	38 (62)	3 (5)	0 (0)	0 (0)	6 (5)	0 (0)	0 (0)	0 (0)
Rash maculo-papular	13 (21)	1 (2)	0 (0)	0 (0)	22 (18)	2 (2)	0 (0)	0 (0)
Renal calculi	0 (0)	0 (0)	0 (0)	0 (0)	0 (0)	1 (1)	0 (0)	0 (0)
Respiratory failure	0 (0)	0 (0)	1 (2)	0 (0)	0 (0)	0 (0)	4 (3)	1 (1)
Respiratory, thoracic and mediastinal disorders - Other	3 (5)	1 (2)	0 (0)	0 (0)	2 (2)	1 (1)	0 (0)	0 (0)
Restlessness	0 (0)	0 (0)	0 (0)	0 (0)	0 (0)	1 (1)	0 (0)	0 (0)
Salivary duct inflammation	4 (7)	1 (2)	0 (0)	0 (0)	5 (4)	1 (1)	0 (0)	0 (0)
Sepsis	0 (0)	1 (2)	0 (0)	0 (0)	0 (0)	1 (1)	1 (1)	0 (0)
Serum amylase increased	0 (0)	0 (0)	0 (0)	0 (0)	6 (5)	2 (2)	0 (0)	0 (0)
Sinus bradycardia	2 (3)	0 (0)	0 (0)	0 (0)	7 (6)	1 (1)	0 (0)	0 (0)
Skin and subcutaneous tissue disorders - Other	7 (11)	0 (0)	0 (0)	0 (0)	7 (6)	0 (0)	0 (0)	0 (0)
Skin infection	2 (3)	1 (2)	0 (0)	0 (0)	5 (4)	3 (3)	0 (0)	0 (0)
Somnolence	1 (2)	0 (0)	0 (0)	0 (0)	2 (2)	1 (1)	0 (0)	0 (0)
Sore throat	22 (36)	1 (2)	0 (0)	0 (0)	33 (28)	5 (4)	0 (0)	0 (0)
Stoma site infection	0 (0)	1 (2)	0 (0)	0 (0)	0 (0)	0 (0)	0 (0)	0 (0)
Stridor	0 (0)	0 (0)	0 (0)	0 (0)	0 (0)	1 (1)	0 (0)	0 (0)
Sudden death NOS	0 (0)	0 (0)	0 (0)	1 (2)	0 (0)	0 (0)	0 (0)	4 (3)
Surgical and medical procedures - Other	0 (0)	0 (0)	0 (0)	0 (0)	1 (1)	1 (1)	0 (0)	0 (0)
Syncope	0 (0)	1 (2)	0 (0)	0 (0)	0 (0)	2 (2)	0 (0)	0 (0)
Thromboembolic event	2 (3)	0 (0)	0 (0)	0 (0)	1 (1)	2 (2)	0 (0)	0 (0)
Thrush	6 (10)	0 (0)	0 (0)	0 (0)	14 (12)	0 (0)	0 (0)	0 (0)
Tinnitus	4 (7)	0 (0)	0 (0)	0 (0)	13 (11)	0 (0)	0 (0)	0 (0)
Tracheal stenosis	0 (0)	0 (0)	0 (0)	0 (0)	0 (0)	1 (1)	0 (0)	0 (0)
Trismus	1 (2)	1 (2)	0 (0)	0 (0)	5 (4)	0 (0)	0 (0)	0 (0)
Tumor hemorrhage	0 (0)	1 (2)	0 (0)	0 (0)	1 (1)	0 (0)	0 (0)	0 (0)
Tumor pain	0 (0)	0 (0)	0 (0)	0 (0)	0 (0)	1 (1)	0 (0)	0 (0)
Urinary tract infection	1 (2)	1 (2)	0 (0)	0 (0)	2 (2)	0 (0)	0 (0)	0 (0)
Vasovagal reaction	0 (0)	0 (0)	0 (0)	0 (0)	0 (0)	1 (1)	0 (0)	0 (0)
Vision decreased	0 (0)	0 (0)	0 (0)	0 (0)	0 (0)	1 (1)	0 (0)	0 (0)
Voice alteration	1 (2)	0 (0)	0 (0)	0 (0)	13 (11)	0 (0)	0 (0)	0 (0)
Vomiting	17	2	0	0	22	2	0	0

Table A9: Adverse events by treatment arm, without regard to attribution (Complete)

Term	Cetuximab (n=61)				Durvalumab (n=119)			
	n (%) by grade				n (%) by grade			
	1-2	3	4	5	1-2	3	4	5
	(28)	(3)	(0)	(0)	(18)	(2)	(0)	(0)
Weight loss	27 (44)	5 (8)	0 (0)	0 (0)	53 (45)	14 (12)	0 (0)	0 (0)
Wheezing	4 (7)	0 (0)	0 (0)	0 (0)	11 (9)	1 (1)	0 (0)	0 (0)
White blood cell decreased	8 (13)	1 (2)	0 (0)	0 (0)	14 (12)	1 (1)	1 (1)	0 (0)

Adverse events were graded with CTCAE version 5.
Limited to grade 1-2 incidence of at least 10% on either arm and all grade 3-5.

Table A10: Treatment-related* serious adverse events by treatment arm

Term	Cetuximab (n=61)				Durvalumab (n=119)			
	n (%) by grade				n (%) by grade			
	1-2	3	4	5	1-2	3	4	5
Overall highest grade	1 (2)	12 (20)	1 (2)	1 (2)	1 (1)	22 (18)	2 (2)	4 (3)
Acute kidney injury	0 (0)	0 (0)	0 (0)	0 (0)	0 (0)	2 (2)	0 (0)	0 (0)
Alanine aminotransferase increased	0 (0)	0 (0)	0 (0)	0 (0)	0 (0)	1 (1)	0 (0)	0 (0)
Allergic reaction	0 (0)	1 (2)	0 (0)	0 (0)	0 (0)	0 (0)	0 (0)	0 (0)
Anaphylaxis	0 (0)	1 (2)	0 (0)	0 (0)	0 (0)	0 (0)	0 (0)	0 (0)
Anorexia	0 (0)	0 (0)	0 (0)	0 (0)	0 (0)	2 (2)	0 (0)	0 (0)
Aspartate aminotransferase increased	0 (0)	0 (0)	0 (0)	0 (0)	0 (0)	2 (2)	0 (0)	0 (0)
Aspiration	0 (0)	2 (3)	0 (0)	0 (0)	0 (0)	3 (3)	0 (0)	0 (0)
Atrial fibrillation	1 (2)	1 (2)	0 (0)	0 (0)	0 (0)	0 (0)	0 (0)	0 (0)
Bone infection	0 (0)	1 (2)	0 (0)	0 (0)	0 (0)	0 (0)	0 (0)	0 (0)
Chills	1 (2)	0 (0)	0 (0)	0 (0)	0 (0)	0 (0)	0 (0)	0 (0)
Cognitive disturbance	0 (0)	0 (0)	0 (0)	0 (0)	1 (1)	0 (0)	0 (0)	0 (0)
Creatinine increased	0 (0)	0 (0)	0 (0)	0 (0)	0 (0)	1 (1)	0 (0)	0 (0)
Death NOS	0 (0)	0 (0)	0 (0)	0 (0)	0 (0)	0 (0)	0 (0)	1 (1)
Dehydration	0 (0)	4 (7)	0 (0)	0 (0)	0 (0)	5 (4)	0 (0)	0 (0)
Dermatitis radiation	0 (0)	2 (3)	0 (0)	0 (0)	0 (0)	1 (1)	0 (0)	0 (0)
Diarrhea	0 (0)	0 (0)	0 (0)	0 (0)	0 (0)	1 (1)	0 (0)	0 (0)
Dizziness	0 (0)	0 (0)	0 (0)	0 (0)	0 (0)	1 (1)	0 (0)	0 (0)
Dysphagia	0 (0)	4 (7)	0 (0)	0 (0)	0 (0)	3 (3)	0 (0)	0 (0)
Dyspnea	1 (2)	0 (0)	0 (0)	0 (0)	2 (2)	2 (2)	0 (0)	0 (0)
Esophageal pain	0 (0)	0 (0)	0 (0)	0 (0)	0 (0)	1 (1)	0 (0)	0 (0)
Fall	0 (0)	0 (0)	0 (0)	0 (0)	1 (1)	1 (1)	0 (0)	0 (0)
Fatigue	0 (0)	0 (0)	0 (0)	0 (0)	1 (1)	0 (0)	0 (0)	0 (0)
Fever	1 (2)	0 (0)	0 (0)	0 (0)	0 (0)	0 (0)	0 (0)	0 (0)
Gastrointestinal disorders - Other	0 (0)	0 (0)	0 (0)	0 (0)	0 (0)	1 (1)	0 (0)	0 (0)
Hepatic failure	0 (0)	0 (0)	0 (0)	0 (0)	0 (0)	1 (1)	0 (0)	0 (0)
Hyperglycemia	0 (0)	1 (2)	0 (0)	0 (0)	1 (1)	1 (1)	0 (0)	0 (0)
Hypoglycemia	0 (0)	0 (0)	0 (0)	0 (0)	0 (0)	0 (0)	1 (1)	0 (0)
Hyponatremia	0 (0)	0 (0)	1 (2)	0 (0)	0 (0)	0 (0)	0 (0)	0 (0)
Hypotension	0 (0)	0 (0)	0 (0)	0 (0)	0 (0)	2 (2)	0 (0)	0 (0)
Hypoxia	0 (0)	0 (0)	0 (0)	0 (0)	0 (0)	1 (1)	0 (0)	0 (0)
Injury to carotid artery	0	0	0	0	0	1	0	0

Table A10: Treatment-related* serious adverse events by treatment arm

Term	Cetuximab (n=61) n (%) by grade				Durvalumab (n=119) n (%) by grade			
	1-2	3	4	5	1-2	3	4	5
	(0)	(0)	(0)	(0)	(0)	(1)	(0)	(0)
Laryngeal edema	0 (0)	0 (0)	0 (0)	0 (0)	1 (1)	2 (2)	0 (0)	1 (1)
Lethargy	0 (0)	0 (0)	0 (0)	0 (0)	1 (1)	0 (0)	0 (0)	0 (0)
Localized edema	0 (0)	0 (0)	0 (0)	0 (0)	0 (0)	1 (1)	0 (0)	0 (0)
Lung infection	0 (0)	3 (5)	0 (0)	0 (0)	0 (0)	0 (0)	0 (0)	1 (1)
Lymphocyte count decreased	1 (2)	0 (0)	0 (0)	0 (0)	0 (0)	0 (0)	0 (0)	0 (0)
Mucositis oral	0 (0)	1 (2)	0 (0)	0 (0)	1 (1)	1 (1)	0 (0)	0 (0)
Myasthenia gravis	0 (0)	0 (0)	0 (0)	0 (0)	0 (0)	1 (1)	0 (0)	0 (0)
Myocardial infarction	0 (0)	0 (0)	0 (0)	0 (0)	0 (0)	1 (1)	0 (0)	0 (0)
Nausea	0 (0)	0 (0)	0 (0)	0 (0)	0 (0)	1 (1)	0 (0)	0 (0)
Pneumonitis	0 (0)	0 (0)	0 (0)	0 (0)	1 (1)	1 (1)	1 (1)	0 (0)
Rash maculo-papular	0 (0)	0 (0)	0 (0)	0 (0)	0 (0)	1 (1)	0 (0)	0 (0)
Respiratory failure	0 (0)	0 (0)	0 (0)	0 (0)	0 (0)	0 (0)	1 (1)	1 (1)
Sepsis	0 (0)	1 (2)	0 (0)	0 (0)	0 (0)	0 (0)	0 (0)	0 (0)
Sinus bradycardia	0 (0)	0 (0)	0 (0)	0 (0)	1 (1)	0 (0)	0 (0)	0 (0)
Skin infection	0 (0)	0 (0)	0 (0)	0 (0)	0 (0)	1 (1)	0 (0)	0 (0)
Sudden death NOS	0 (0)	0 (0)	0 (0)	1 (2)	0 (0)	0 (0)	0 (0)	0 (0)
Tracheal mucositis	0 (0)	0 (0)	0 (0)	0 (0)	1 (1)	0 (0)	0 (0)	0 (0)
Urinary tract infection	0 (0)	1 (2)	0 (0)	0 (0)	0 (0)	0 (0)	0 (0)	0 (0)
Vomiting	0 (0)	1 (2)	0 (0)	0 (0)	0 (0)	1 (1)	0 (0)	0 (0)
Weight loss	0 (0)	0 (0)	0 (0)	0 (0)	0 (0)	2 (2)	0 (0)	0 (0)

Adverse events were graded with CTCAE version 5.
* Definitely, probably, or possibly related to protocol treatment.

Table A11: Cetuximab and durvalumab discontinuations due to adverse events

Cetuximab (n=3)

1. Anaphylaxis
2. G-tube leak
3. Dermatitis radiation

Durvalumab (n=11)

1. Aspartate aminotransferase increased; creatinine increased
2. Weight loss
3. Arthritis; joint effusion
4. Diarrhea
5. Colitis
6. Hepatic failure
7. Localized edema
8. Acute kidney injury; creatinine increased; serum amylase increased
9. Pneumonitis
10. Not specified
11. Myasthenia gravis

Table A12: Non-protocol treatment in patients with disease progression

	**Cetuximab (n=15)**	**Durvalumab (n=38)**
Any	11 (73%)	21 (55%)
Radiation therapy	2 (13%)	4 (11%)
Chemotherapy	4 (27%)	13 (34%)
Biologic/targeted therapy	0	2 (5%)
Immunotherapy	8 (53%)	7 (18%)
Surgery	2 (13%)	8 (21%)

SUPPLEMENTARY FIGURES

Figure A1: Kaplan-Meier estimates of progression-free survival by primary site and p16 status (p16-positive oropharyngeal/unknown primary vs. all others) and treatment arm.

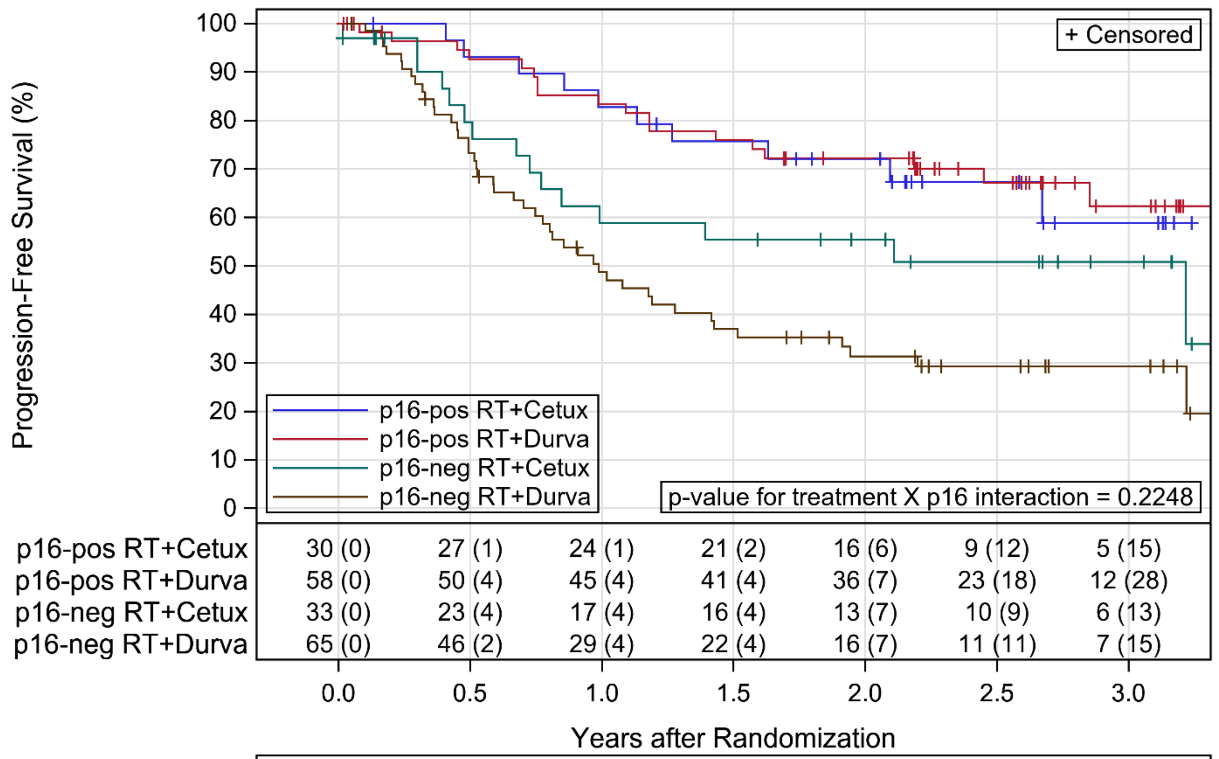

	No. of Patients	Failed	Censored	HR (95% CI)
p16-pos RT+Cetux	30	10	20	Reference
p16-pos RT+Durva	58	18	40	0.92 (0.42-2.00)
p16-neg RT+Cetux	33	15	18	Reference
p16-neg RT+Durva	65	44	21	1.68 (0.93-3.03)

Figure A2: Progression-free survival by PD-L1 CPS (0 vs. ≥1) and assigned treatment (Panel A) and progression-free survival by PD-L1 CPS (<20 vs. ≥20) and assigned treatment (Panel B)

A

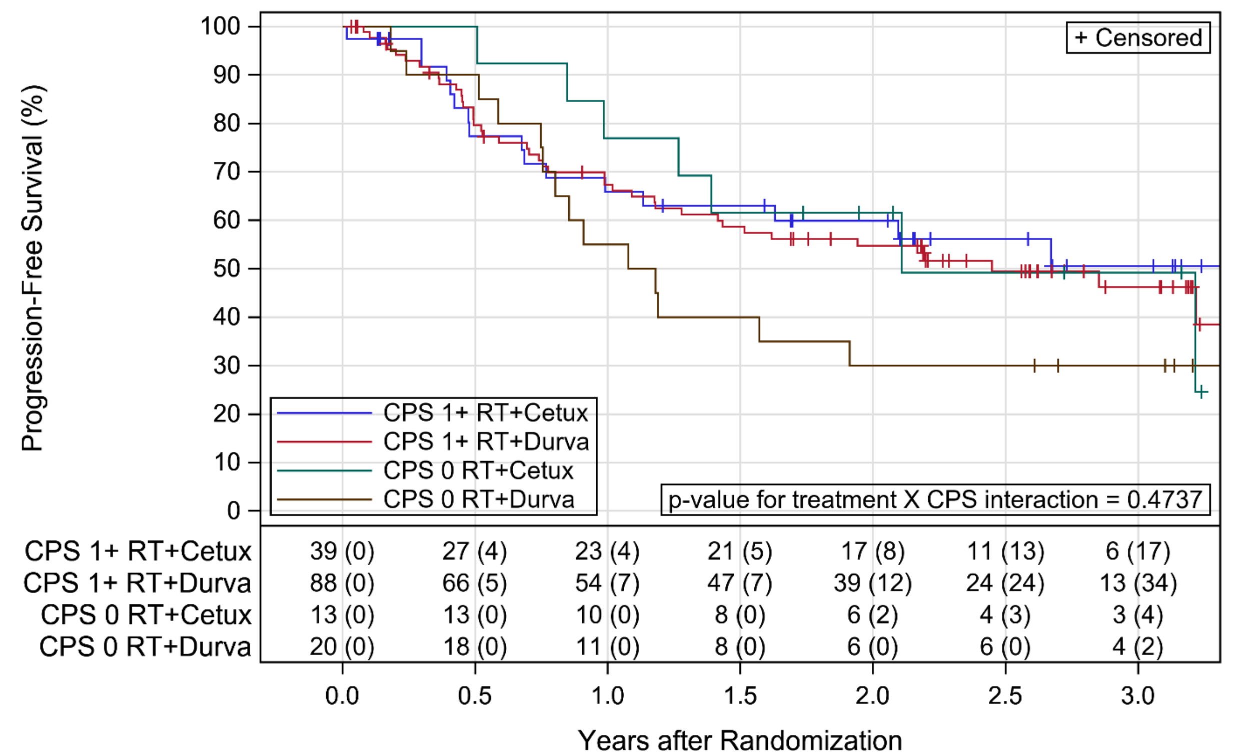

	No. of Patients	Failed	Censored	HR (95% CI)
CPS 1+ RT+Cetux	39	16	23	Reference
CPS 1+ RT+Durva	88	42	46	1.11 (0.62-1.97)
CPS 0 RT+Cetux	13	7	6	Reference
CPS 0 RT+Durva	20	14	6	1.64 (0.66-4.06)

B

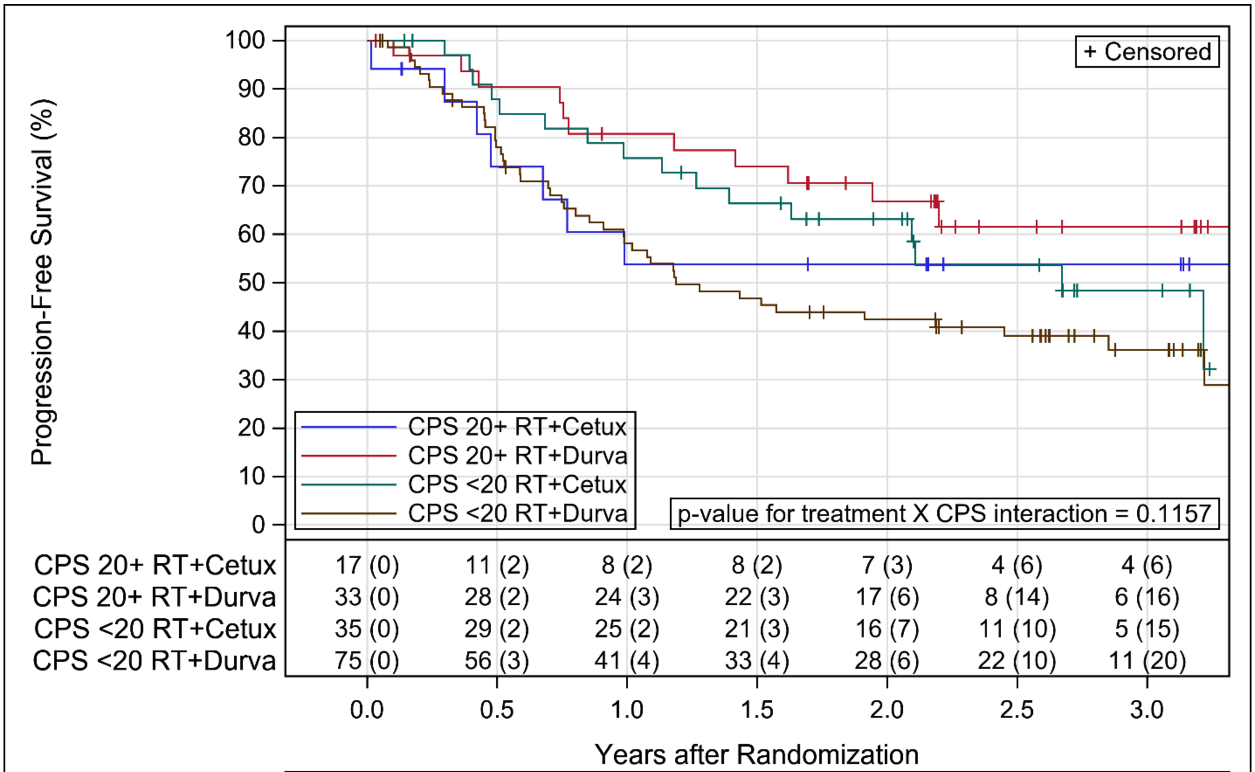

	No. of Patients	Failed	Censored	HR (95% CI)
CPS 20+ RT+Cetux	17	7	10	Reference
CPS 20+ RT+Durva	33	11	22	0.63 (0.24-1.62)
CPS <20 RT+Cetux	35	16	19	Reference
CPS <20 RT+Durva	75	45	30	1.53 (0.86-2.71)

Figure A3: Kaplan-Meier estimates of overall survival by treatment arm

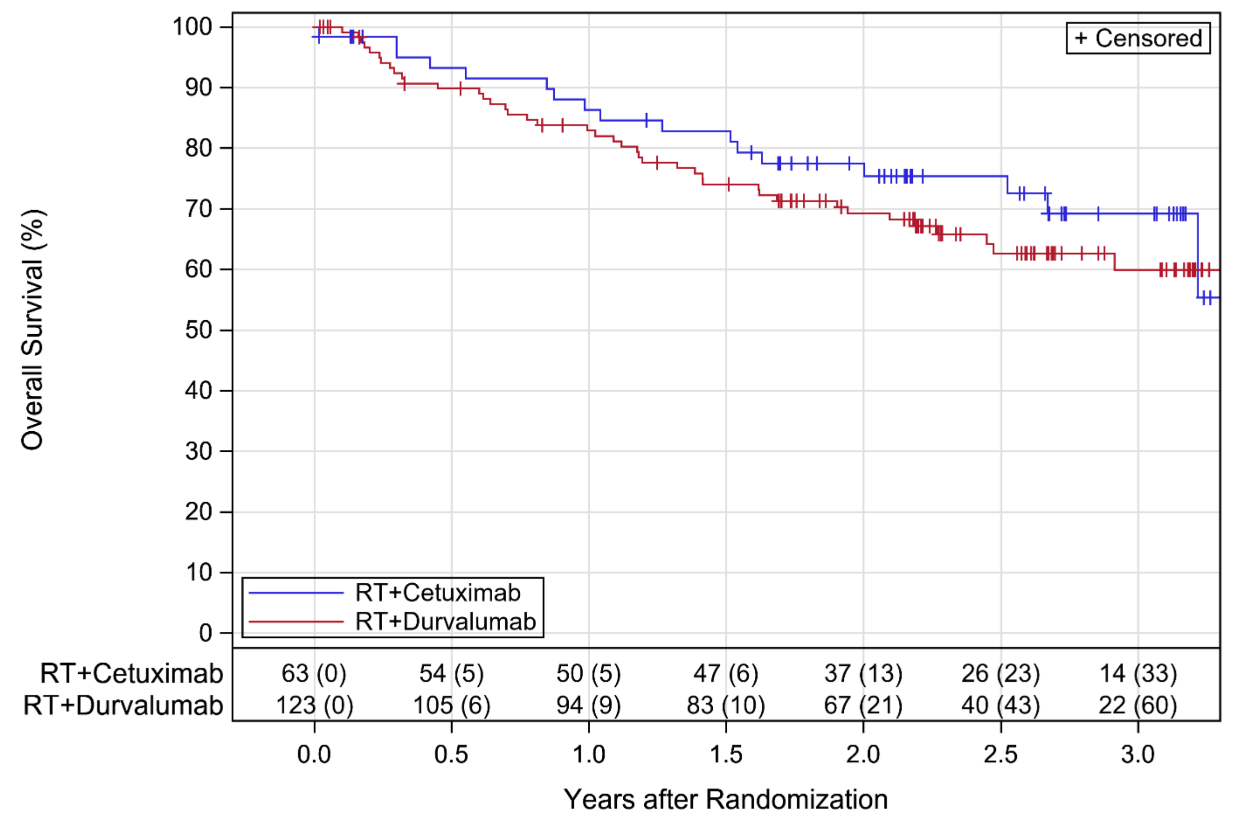

	No. of Patients	Failed	Censored	HR (95% CI)
RT+Cetuximab	63	17	46	Reference
RT+Durvalumab	123	41	82	1.30 (0.74-2.28)

Figure A4: Cumulative incidence estimates of locoregional failure (LRF) by treatment arm (death from unknown causes is a failure for this endpoint; see definition of failure types in Table A6).

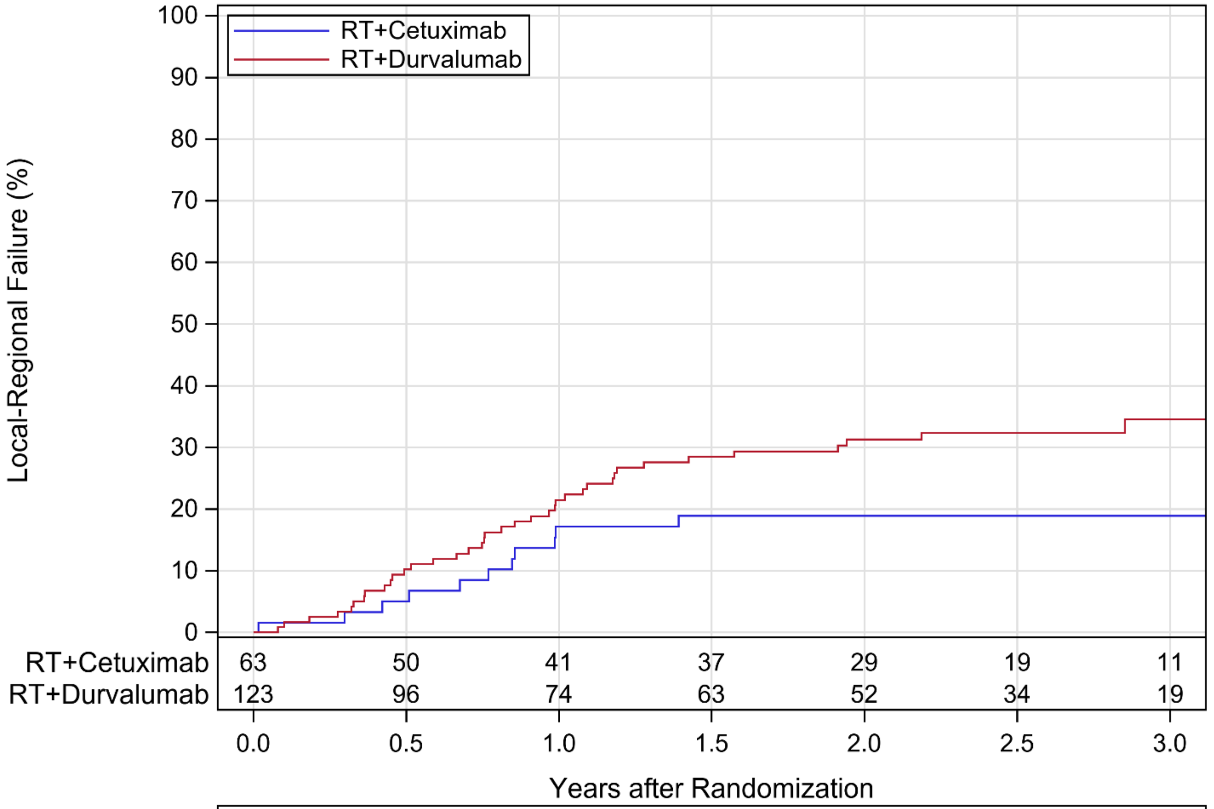

	No. of Patients	Failed	Censored	Competing Risk	HR (95% CI)
RT+Cetuximab	63	12	38	13	Reference
RT+Durvalumab	123	38	61	24	1.71 (0.89-3.28)

Figure A5: Cumulative incidence estimates of locoregional failure (LRF2) by treatment arm (death from unknown causes is not a failure for this endpoint; see definition of failure types in Table A6).

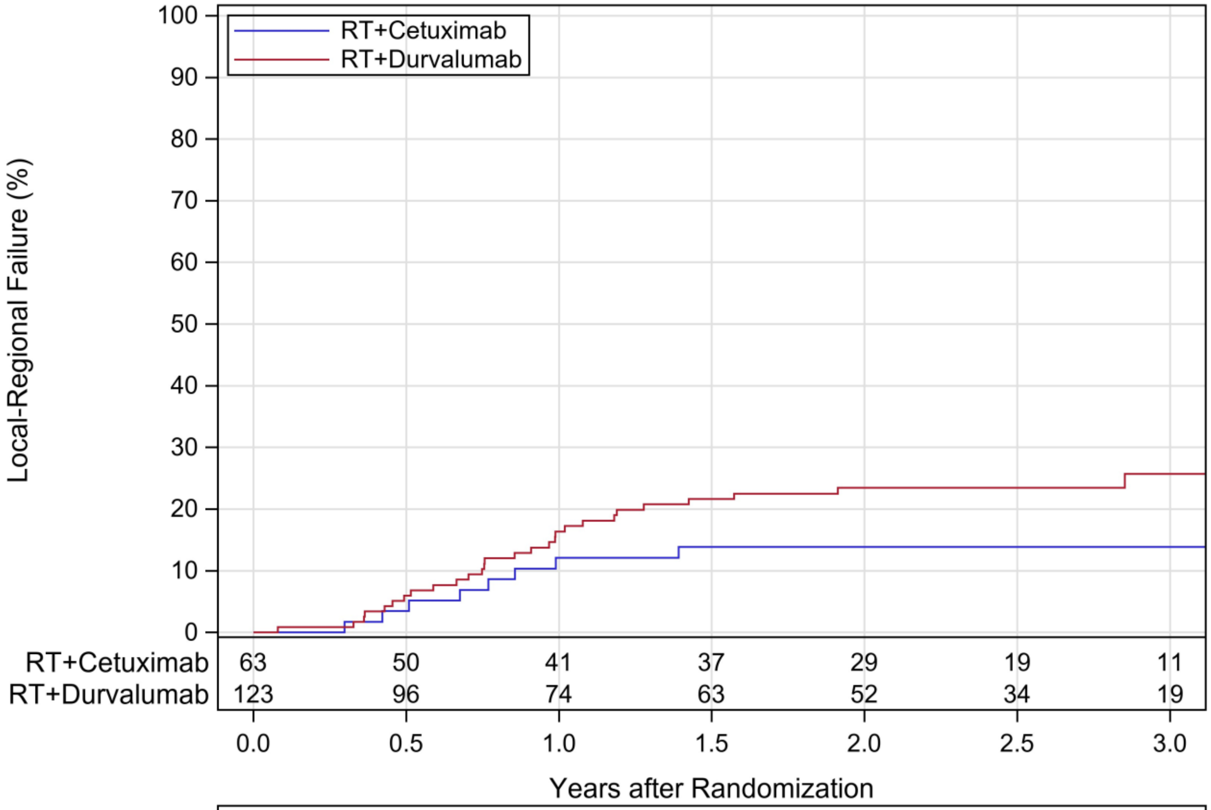

	No. of Patients	Failed	Censored	Competing Risk	HR (95% CI)
RT+Cetuximab	63	8	38	17	Reference
RT+Durvalumab	123	28	61	34	1.91 (0.87-4.19)

Figure A6: Cumulative incidence estimates of distant metastasis by treatment arm

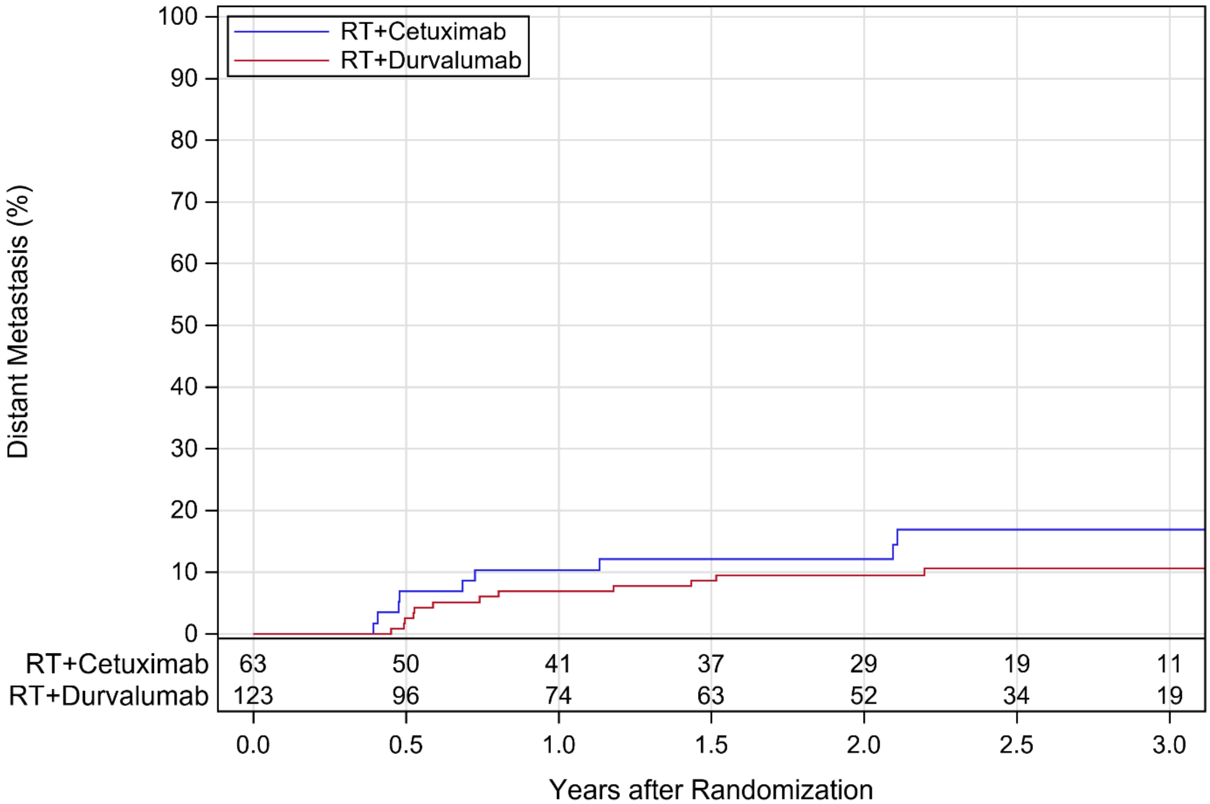

	No. of Patients	Failed	Censored	Competing Risk	HR (95% CI)
RT+Cetuximab	63	9	38	16	Reference
RT+Durvalumab	123	13	61	49	0.76 (0.32-1.77)

Figure A7: Cumulative incidence estimates of competing mortality (CM) by treatment arm (death with unknown causes is not a failure for this endpoint; see CM definition in Table A6).

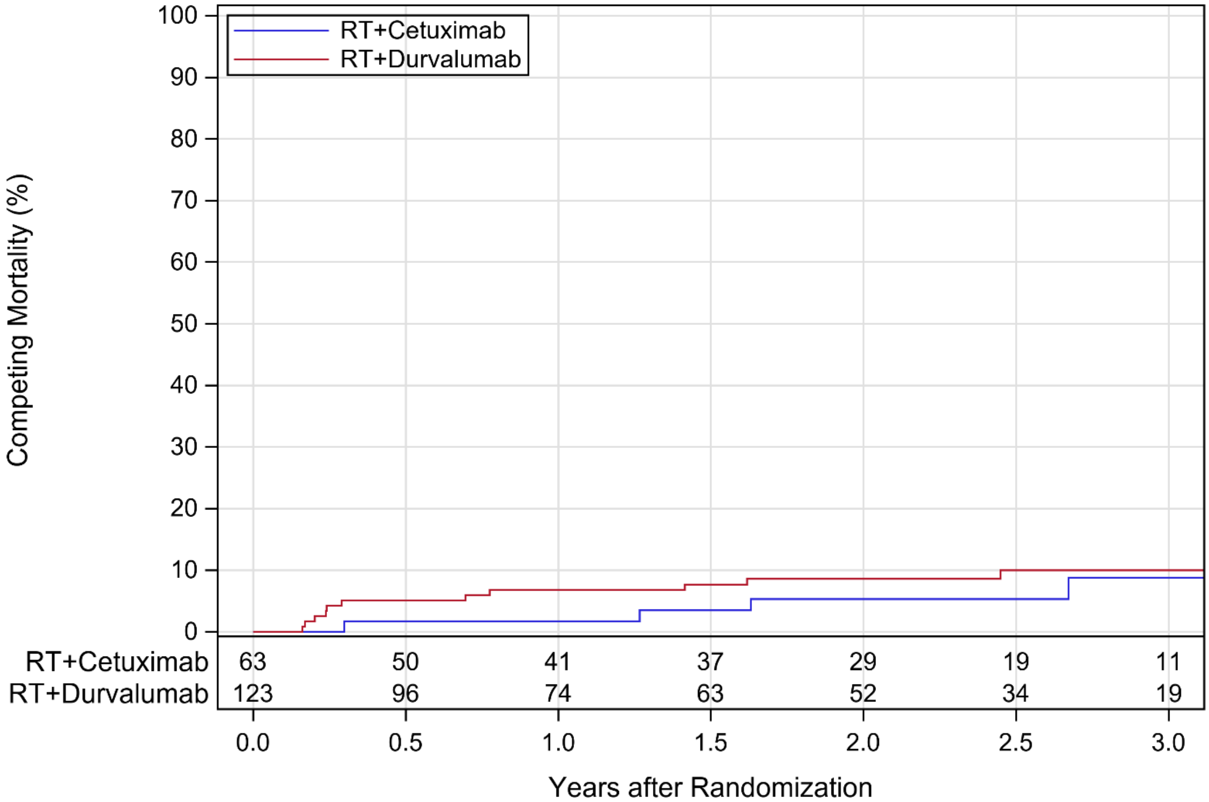

	No. of Patients	Failed	Censored	Competing Risk	HR (95% CI)
RT+Cetuximab	63	4	38	21	Reference
RT+Durvalumab	123	11	61	51	1.50 (0.48-4.70)

Figure A8: Cumulative incidence estimates of competing mortality (CM2) by treatment arm (death with unknown causes is a failure for this endpoint; see CM definition in Table A6).

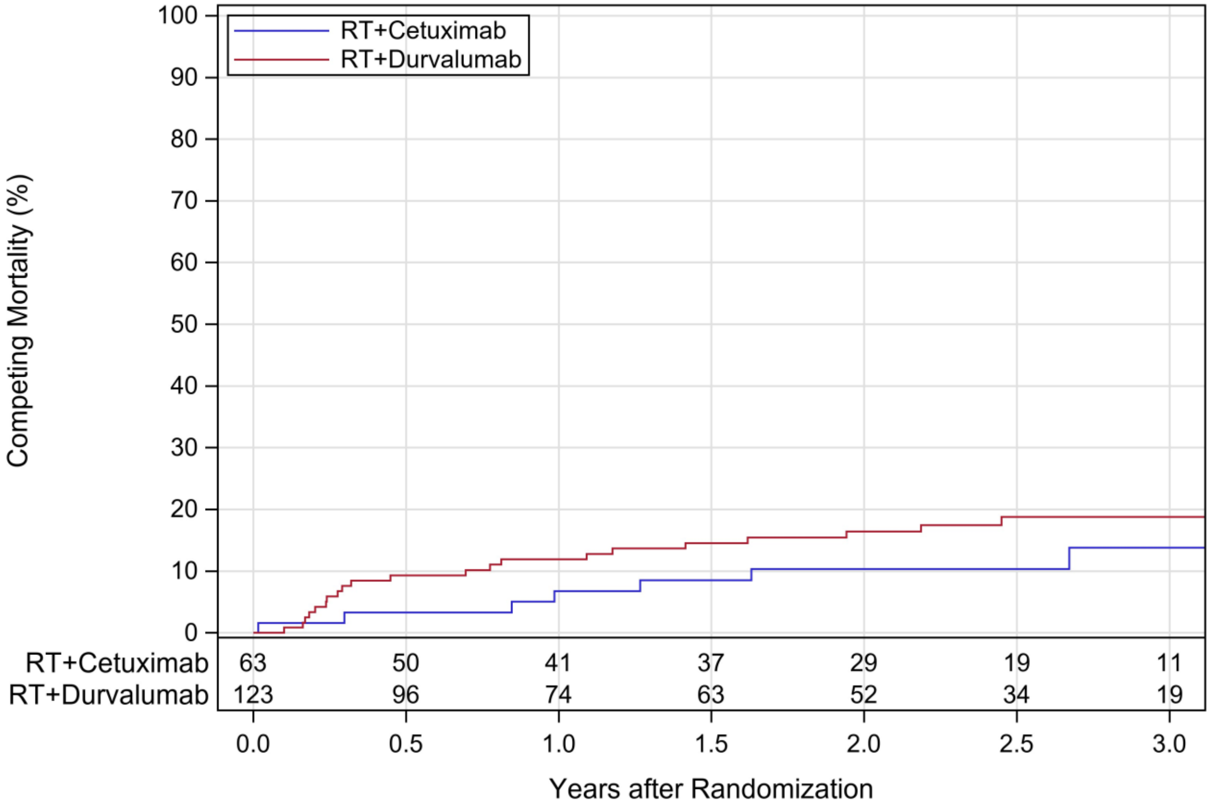

	No. of Patients	Failed	Censored	Competing Risk	HR (95% CI)
RT+Cetuximab	63	8	38	17	Reference
RT+Durvalumab	123	21	61	41	1.41 (0.62-3.18)

Figure A9: Kaplan-Meier estimates of overall survival by primary site and p16 status (p16-positive oropharyngeal/unknown primary vs. all others) and treatment arm.

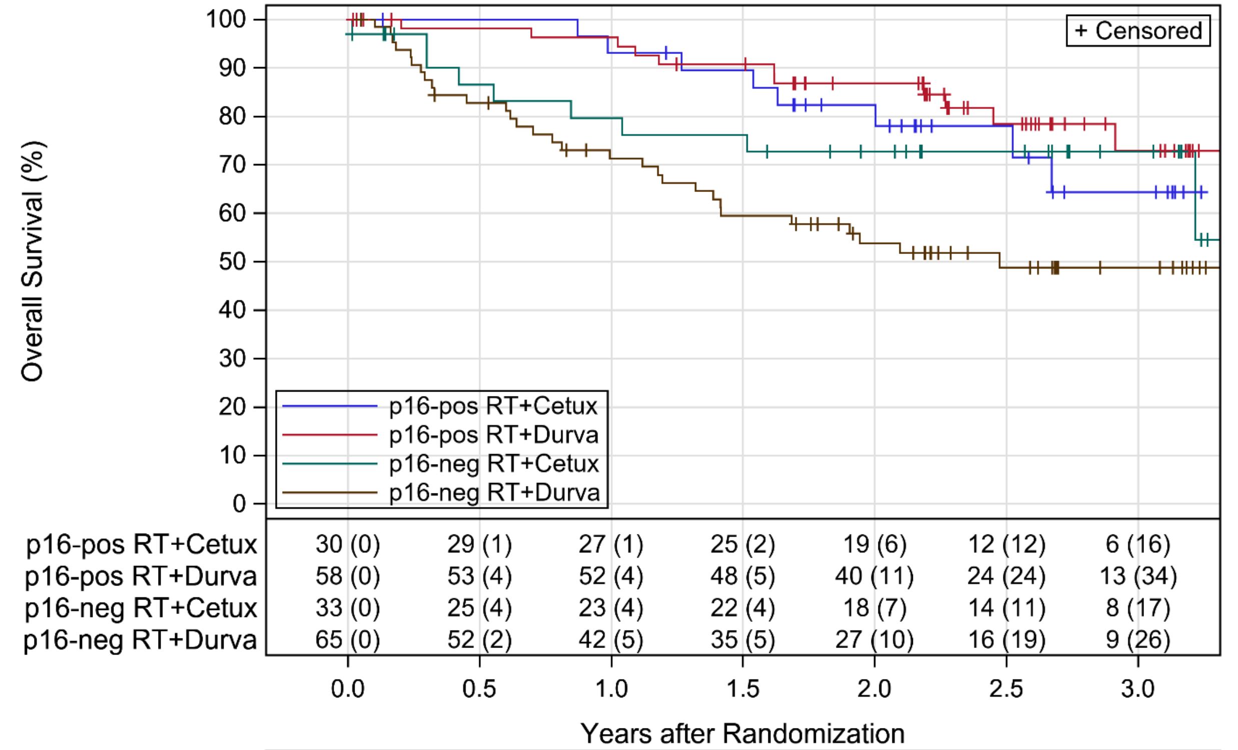

	No. of Patients	Failed	Censored
p16-pos RT+Cetux	30	8	22
p16-pos RT+Durva	58	11	47
p16-neg RT+Cetux	33	9	24
p16-neg RT+Durva	65	30	35

Figure A10: Cumulative incidence estimates of locoregional failure by primary site and p16 status (p16-positive oropharyngeal/unknown primary vs. all others) and treatment arm (note this is a combined endpoint; see definition of failure types in Table A6).

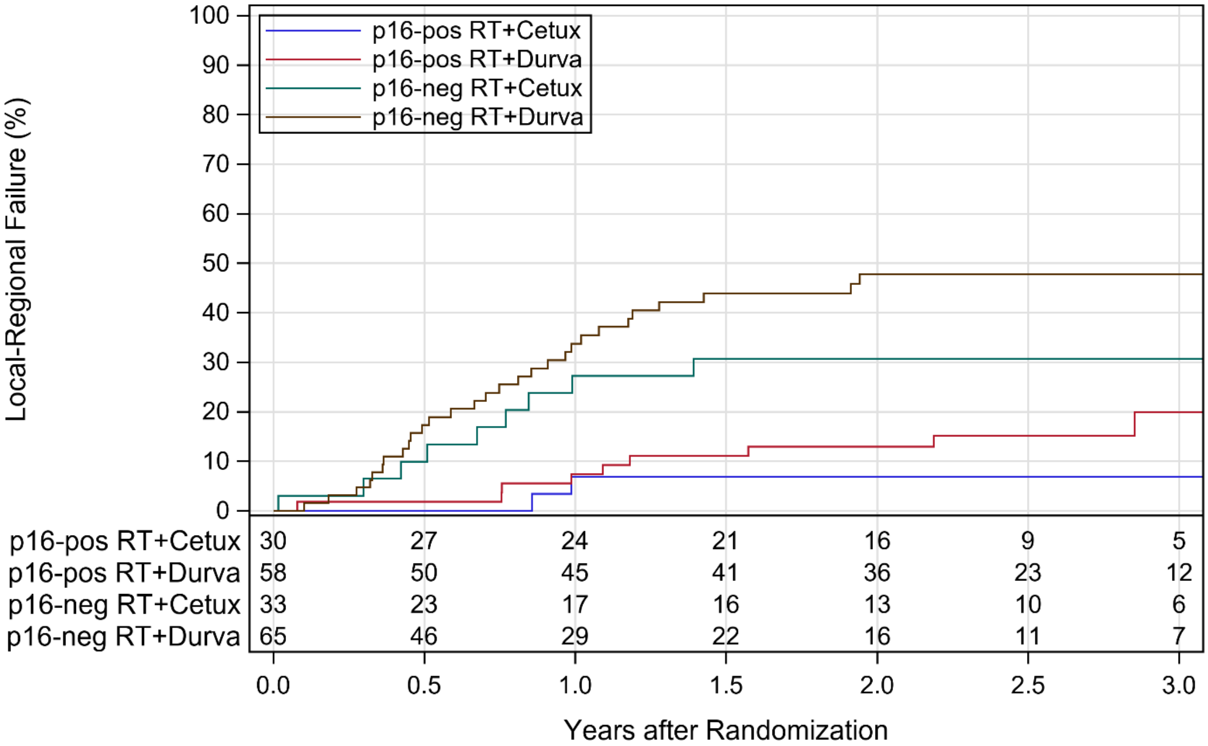

	No. of Patients	Failed	Censored	Competing Risk
p16-pos RT+Cetux	30	2	20	8
p16-pos RT+Durva	58	9	40	9
p16-neg RT+Cetux	33	10	18	5
p16-neg RT+Durva	65	29	21	15

Figure A11: Cumulative incidence estimates of locoregional failure (LRF2) by primary site and p16 status (p16-positive oropharyngeal/unknown primary vs. all others) and treatment arm (death from unknown causes is not a failure for this endpoint; see definition of failure types in Table A6).

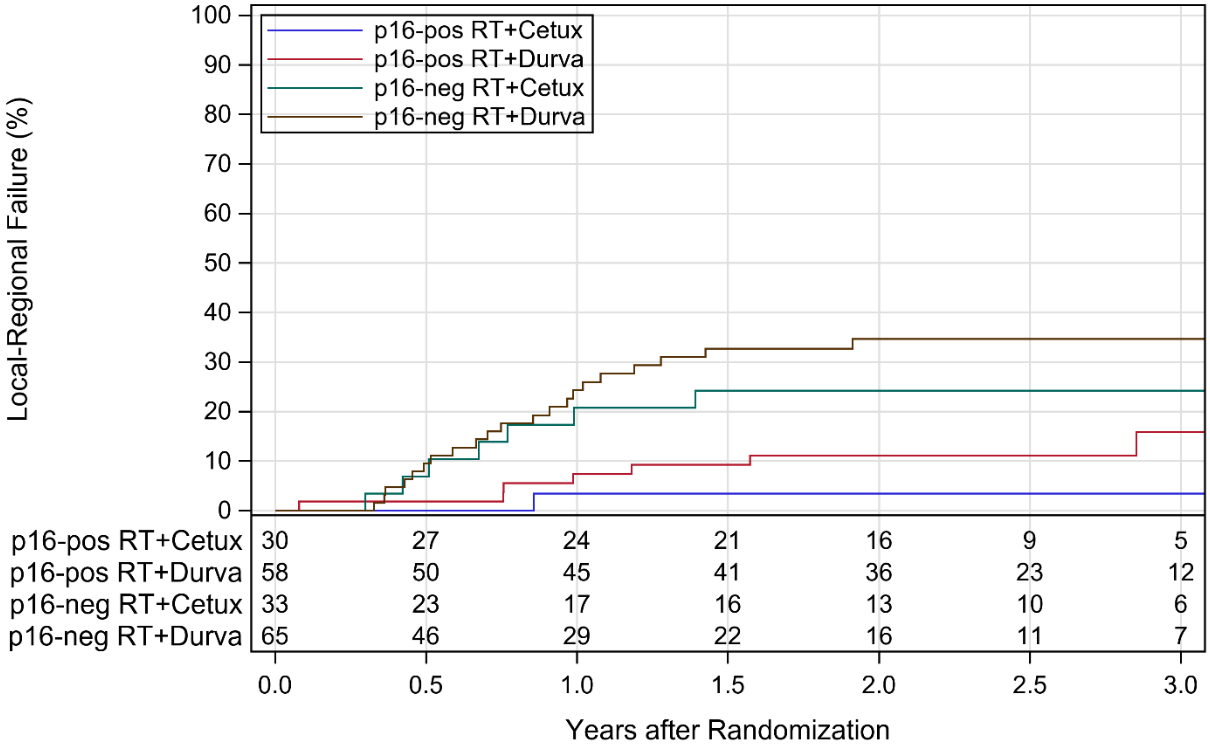

	No. of Patients	Failed	Censored	Competing Risk
p16-pos RT+Cetux	30	1	20	9
p16-pos RT+Durva	58	7	40	11
p16-neg RT+Cetux	33	7	18	8
p16-neg RT+Durva	65	21	21	23

Figure A12: Cumulative incidence estimates of distant metastasis by primary site and p16 status (p16-positive oropharyngeal/unknown primary vs. all others) and treatment arm.

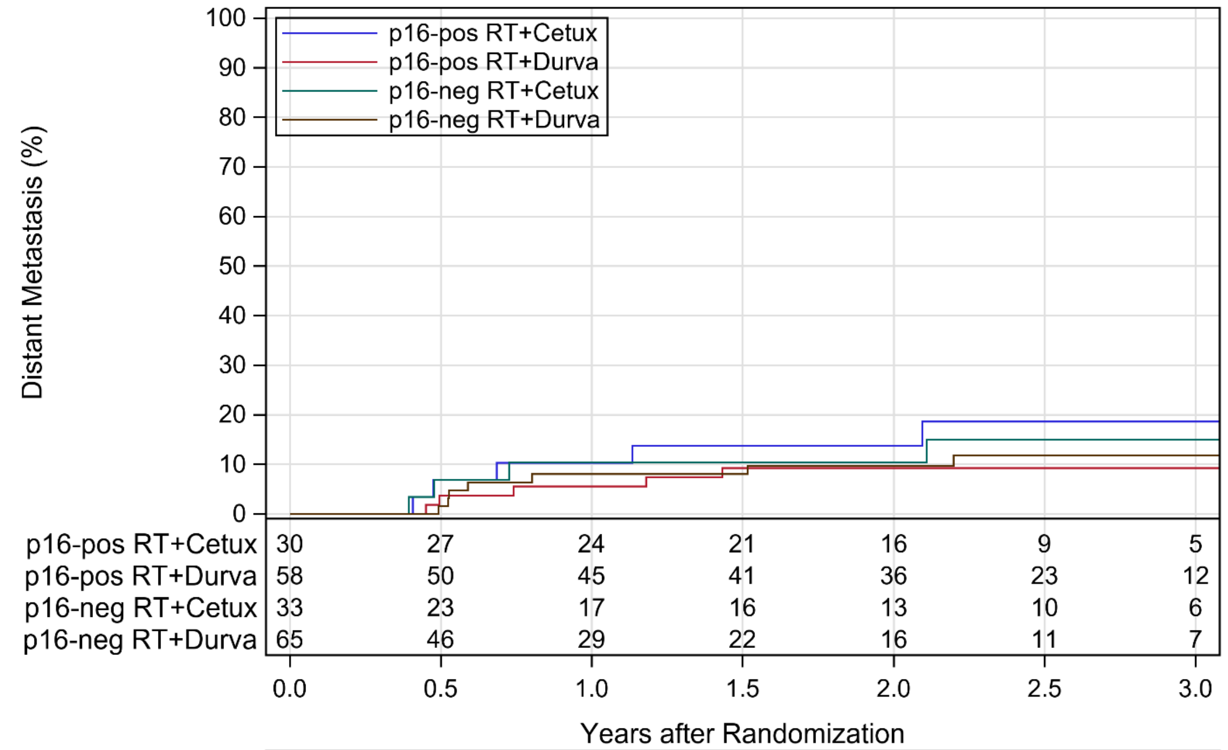

	No. of Patients	Failed	Censored	Competing Risk
p16-pos RT+Cetux	30	5	20	5
p16-pos RT+Durva	58	5	40	13
p16-neg RT+Cetux	33	4	18	11
p16-neg RT+Durva	65	8	21	36

Figure A13: Cumulative incidence estimates of competing mortality by primary site and p16 status (p16-positive oropharyngeal/unknown primary vs. all others) and treatment arm.

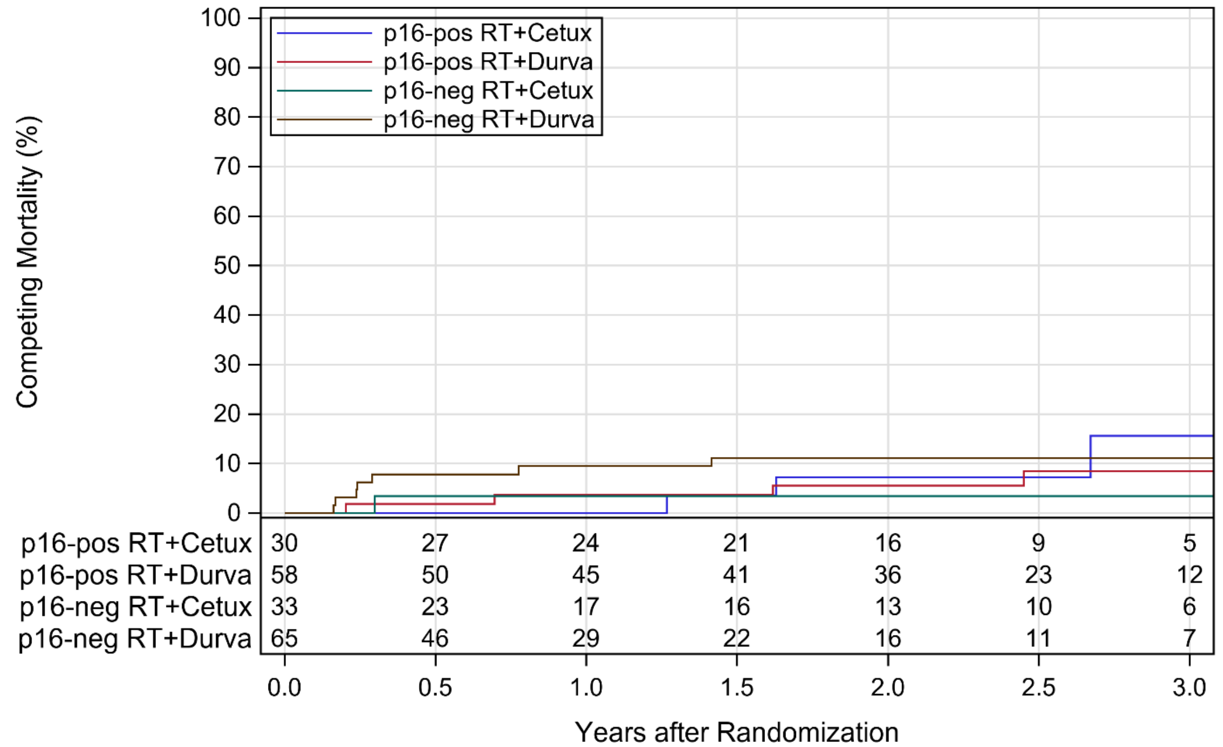

	No. of Patients	Failed	Censored	Competing Risk
p16-pos RT+Cetux	30	3	20	7
p16-pos RT+Durva	58	4	40	14
p16-neg RT+Cetux	33	1	18	14
p16-neg RT+Durva	65	7	21	37

Figure A14: Cumulative incidence estimates of competing mortality (CM2) by primary site and p16 status (p16-positive oropharyngeal/unknown primary vs. all others) and treatment arm (death with unknown causes is a failure for this endpoint; see CM definition in Table A6).

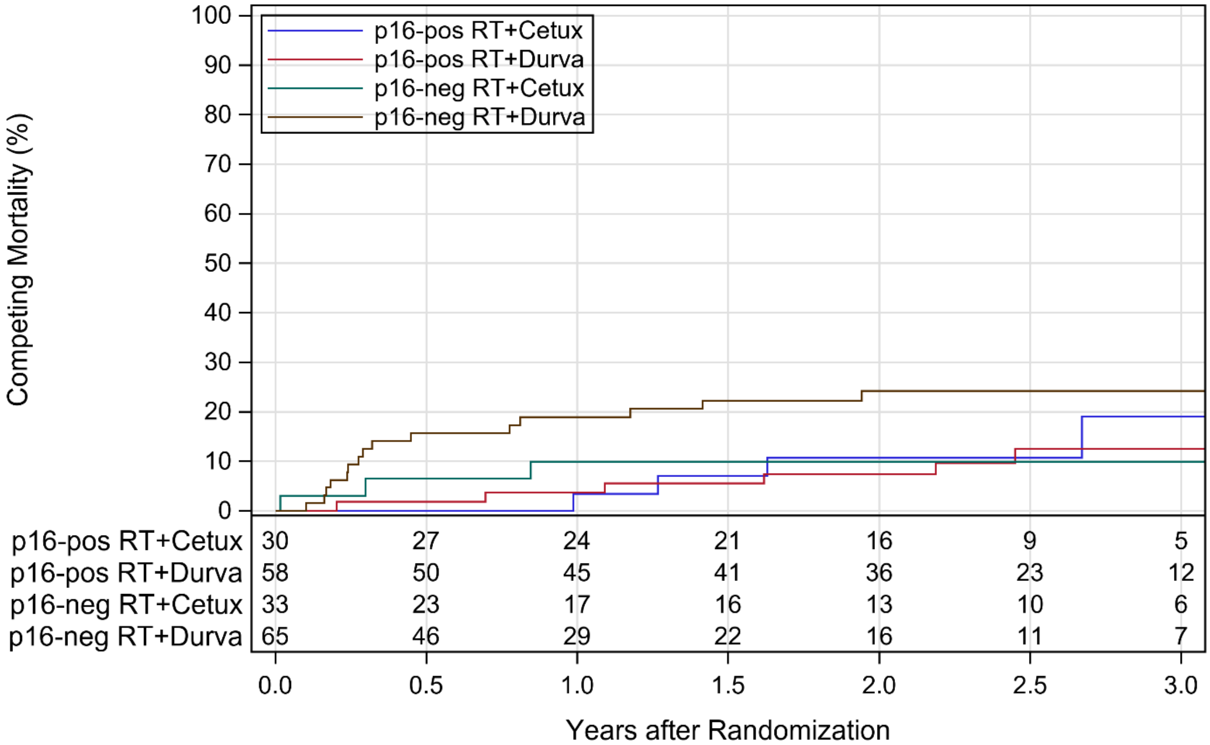

	No. of Patients	Failed	Censored	Competing Risk
p16-pos RT+Cetux	30	4	20	6
p16-pos RT+Durva	58	6	40	12
p16-neg RT+Cetux	33	4	18	11
p16-neg RT+Durva	65	15	21	29

SITES, INVESTIGATORS, AND NUMBER OF PATIENTS RECRUITED

Site	Number of Patients	Principal Investigator
University of Cincinnati Cancer Center-UC Medical Center	9	Jordan Kharofa, MD
University of Washington Medical Center - Montlake	9	Heidi Gray, MD
Stanford Cancer Institute Palo Alto	8	Elizabeth Kidd, MD
University of Oklahoma Health Sciences Center	8	Debra Richardson, MD
Sanford USD Medical Center - Sioux Falls	7	Daniel Almquist, MD
University of Alabama at Birmingham Cancer Center	7	Andrew McDonald, MD
The James Graham Brown Cancer Center at University of Louisville	6	Jason Chesney, MD, PhD
UC San Diego Moores Cancer Center	6	Loren K Mell, MD
University of Arizona Cancer Center-North Campus	6	Baldassarre Stea, MD, PhD
Banner University Medical Center - Tucson	4	Baldassarre Stea, MD, PhD
Cedars-Sinai Medical Center	4	Zachary Zumsteg, MD
Emory University Hospital Midtown	4	Ashesh Jani, MD
Helen F Graham Cancer Center	4	Adam Raben, MD
Henry Ford Hospital	4	Eleanor Walker, MD
Medical College of Wisconsin	4	Elizabeth Gore, MD
Penn State Milton S Hershey Medical Center	4	Mitchell Machtay, MD
Trinity Health Saint Joseph Mercy Hospital Ann Arbor	4	Philip Stella, MD
University of Cincinnati Cancer Center-West Chester	4	Thomas Herzog, MD
University of Virginia Cancer Center	4	Linda Duska, MD, MPH
University of Wisconsin Carbone Cancer Center	4	Andrew Baschnagel, MD
Yale University	4	Henry Park, MD
Medical University of South Carolina	3	David Marshall, MD
State University of New York Upstate Medical University	3	Mary Cunningham, MD
UCSF Medical Center-Mission Bay	3	Sue Yom, MD, PhD
Allegheny General Hospital	2	Thomas Julian, MD
Atrium Health Cabarrus/LCI-Concord	2	Antoinette Tan, MD
Aurora Saint Luke's Medical Center	2	Peter Polewski, MD
Chelsea Hospital	2	Philip Stella, MD
Indiana University/Melvin and Bren Simon Cancer Center	2	Naoyuki Saito, MD, PhD
Laura and Isaac Perlmutter Cancer Center at NYU Langone	2	Bhavana Pothuri, MD
Memorial Sloan Kettering Commack	2	Narek Shaverdian, MD
Moffitt Cancer Center	2	Jimmy J Caudell, MD, PhD
Spartanburg Medical Center	2	James Bearden, MD
VA Palo Alto Health Care System	2	Elizabeth Kidd, MD
Zablocki Veterans Administration Medical Center	2	Elizabeth Gore, MD
Alta Bates Summit Medical Center-Herrick Campus	1	Christopher U Jones, MD
Ascension Via Christi Hospitals Wichita	1	Pavan Reddy, MD
Aspirus Regional Cancer Center	1	Andrew Huang, MD
Asplundh Cancer Pavilion	1	Maria Werner-Wasik,MD
Atrium Health Stanly/LCI-Albemarle	1	Antoinette Tan, MD
Boston Medical Center	1	Minh Truong, MD
Carle Cancer Center	1	Kendrith Rowland, MD
Case Western Reserve University	1	Steven Waggoner, MD

SITES, INVESTIGATORS, AND NUMBER OF PATIENTS RECRUITED (CONTINUED)

Site	Number of Patients	Principal Investigator
City of Hope Comprehensive Cancer Center	1	Terence Williams, MD, PhD
Coborn Cancer Center at Saint Cloud Hospital	1	Donald Jurgens, MD
Community Cancer Center North	1	Daniel Weed, MD
Fox Chase Cancer Center	1	Thomas Galloway, MD
Froedtert Menomonee Falls Hospital	1	Elizabeth Gore, MD
Froedtert West Bend Hospital/Kraemer Cancer Center	1	Elizabeth Gore, MD
Geisinger Medical Center	1	Fiori Alite, MD
Guthrie Medical Group PC-Robert Packer Hospital	1	Michael Haddock, MD
Hennepin County Medical Center	1	Yan Ji, MD, PhD
Iowa Methodist Medical Center	1	Robert Behrens, MD
John H Stroger Jr Hospital of Cook County	1	Thomas Lad, MD
Kaiser Permanente Northwest	1	Abdul Hai Mansoor, MD
Kaiser Permanente-Walnut Creek	1	Samantha Seaward, MD
McLaren Cancer Institute-Lapeer Region	1	Sai Bikkina, MD
Memorial Sloan Kettering Monmouth	1	Carol Aghajanian, MD
Memorial Sloan Kettering Nassau	1	Carol Aghajanian, MD
MetroHealth Medical Center	1	Tithi Biswas, MD
Montefiore Medical Center - Moses Campus	1	Nitin Ohri, MD
Nebraska Cancer Specialists/Oncology Hematology West PC	1	Richard Deming, MD
Norton Brownsboro Hospital and Medical Campus	1	John Hamm, MD
Regions Hospital	1	Yan Ji, MD, PhD
Rush University Medical Center	1	Ruta Rao, MD
Saint Alphonsus Cancer Care Center-Caldwell	1	John Schallenkamp, MD
Saint Alphonsus Cancer Care Center-Nampa	1	John Schallenkamp, MD
Saint Vincent Hospital Cancer Center at Saint Mary's	1	Matthew Ryan, MD
Sentara Norfolk General Hospital	1	Scott Williams, MD, PhD
Smilow Cancer Hospital Care Center - Waterford	1	Henry Park, MD
Smilow Cancer Hospital Care Center-Trumbull	1	Farzana Pashankar, MD
Sparrow Hospital	1	Gordan Srkalovic, MD, PhD
Stony Brook University Medical Center	1	Michael Pearl, MD
Sutter Cancer Centers Radiation Oncology Services-Roseville	1	Christopher U Jones, MD
The Cancer Center of Hawaii-Liliha	1	Richard Lee, MD, PhD
UH Seidman Cancer Center at Lake Health Mentor Campus	1	Amy Armstrong, MD
UHHS-Westlake Medical Center	1	Amy Armstrong, MD
Unity Hospital	1	Yan Ji, MD, PhD
University Health Network-Princess Margaret Hospital	1	Alexander Sun, MD
University of Illinois	1	Kent Hoskins, MD
University of Miami Miller School of Medicine-Sylvester Cancer Center	1	Alan Libardi Dal Pra, MD
University of Rochester	1	Yuhchyau Chen, MD, PhD
Vince Lombardi Cancer Clinic-Sheboygan	1	Peter Polewski, MD
Virginia Commonwealth University/Massey Cancer Center	1	Timothy Harris, MD PhD
Washington University School of Medicine	1	Premal Thaker, MD
Wayne State University/Karmanos Cancer Institute	1	Matthew Dominello, DO

NRG ONCOLOGY
NRG-HN004 PROTOCOL

(ClinicalTrials.gov NCT # 03258554) (22-MAR-2018)

Randomized Phase II/III Trial of Radiotherapy with Concurrent MEDI4736 (Durvalumab) vs. Radiotherapy with Concurrent Cetuximab in Patients with Locoregionally Advanced Head and Neck Cancer with a Contraindication to Cisplatin (15-JAN-2019)

This trial is part of the National Clinical Trials Network (NCTN) program, which is sponsored by the National Cancer Institute (NCI). The trial will be led by NRG Oncology with the participation of the network of NCTN organizations: the Alliance for Clinical Trials in Oncology; ECOG-ACRIN Medical Group; SWOG; and the Canadian Cancer Trials Group (CCTG).

Coordinating Center:
NRG Oncology, Four Penn Center; 1600 JFK Blvd; Suite 1020; Philadelphia, PA 19103

Study Team (13-JAN-2021)

**Principal Investigator** / Radiation Oncology<br>Loren K. Mell, MD<br>University of California San Diego<br>3855 Health Sciences Drive, MC0843<br>La Jolla, CA 92093<br>858-246-0471 / FAX 858-822-5568<br>lmell@ucsd.edu	Senior Adult Oncology Co-Chair<br>Julie A. Kish, MD<br>Moffitt Cancer Center<br>12902 Magnolia Drive, Tampa, FL 33612<br>813-745-3822 / FAX 813-745-1908<br>julie.kish@moffitt.org
Medical Oncology Co-Chair<br>Stuart Wong, MD<br>Medical College of Wisconsin<br>9200 W. Wisconsin Avenue<br>Milwaukee, WI 53226<br>414-805-4603 / FAX 414-805-4606<br>swong@mcw.edu	Translational Science Co-Chair<br>Andy Minn, MD, PhD<br>University of Pennsylvania<br>421 Curie Blvd, Room 510<br>Philadelphia, PA 19104<br>215-746-5515 / FAX 215-746-5511<br>andyminn@upenn.edu
Surgical Oncology Co-Chair<br>Steven S. Chang MD FACS<br>Henry Ford Cancer Institute<br>Henry Ford Health System<br>2799 West Grand Blvd K-8<br>Detroit MI 48202<br>313-916-5741 / FAX 313-916-7263<br>schang1@hfhs.org	Pathology Co-Chair<br>Richard Jordan, DDS, PhD<br>NRG Oncology Biospecimen Bank<br>2340 Sutter Street, Rm S341<br>San Francisco, CA 94115<br>415-476-7864 / FAX 415-476-5271<br>richard.jordan@ucsf.edu

Study Team Continued

<u>Physics Co-Chair</u><br>Tian Liu, PhD, DABR<br>Emory University<br>Department of Radiation Oncology<br>1365 Clifton Road, Clinic A<br>Atlanta, GA 30322<br>404-778-1848 / FAX 404-778-4139<br>tliu34@emory.edu	<u>Senior Statistician</u><br>Pedro Torres-Saavedra, PhD<br>NRG Oncology<br>50 South 16th St, Suite 2800<br>Philadelphia, PA 19102<br>215-717-0851 / FAX 215-928-0153<br>torresp@nrgoncology.org
<u>Quality of Life Co-Chair</u><br>Minh Tam Truong, MD<br>Department of Radiation Oncology<br>Boston Medical Center<br>Boston University School of Medicine<br>Moakley Building, LL237<br>830 Harrison Ave<br>Boston, MA 02118<br>617-638-7070 / FAX 617-638-7037<br>minh-tam.truong@bmc.org	<u>Canadian Cancer Trials Group (CCTG) Medical Oncology Co-Chair</u><br>Eric W. Winquist, MD<br>London Regional Cancer Program<br>790 Commissioners Road East<br>London, ON N6A 4L6<br>Canada<br>519-685-8640 / FAX 519-685-8624<br>eric.winquist@lhsc.on.ca

NRG Oncology Contact Information (09-MAR-2022)

**Data Management**<br>For questions concerning eligibility or data submission	**Vanita Patel, MS**<br>**Lisa Abate**<br>NRG Oncology<br>50 South 16th St, Suite 2800<br>Philadelphia, PA 19102<br>215-574-3170 (Patel)<br>215-574-3178 (Abate)<br>FAX 215-940-8830<br>patelv@nrgoncology.org<br>abatel@nrgoncology.org
**RTQA**:<br>For questions concerning RT data submission	<u>**Dosimetrist**</u><br>**Marsha Radden, BS, R.T.(R)(T)**<br>NRG Oncology<br>50 South 16th St, Suite 2800<br>Philadelphia, PA 19102<br>215-717-2757<br>mradden@acr.org

NRG Oncology Contact Information Continued

**RTQA**:<br>For questions concerning RT Credentialing	http://irochouston.mdanderson.org<br>OR<br>IROC-Credentialing@mdanderson.org
**RTQA**:<br>For questions concerning data submission to TRIAD	Triad-Support@acr.org
**Protocol Development**:<br>For questions concerning protocol and informed consent versions & amendments	**Protocol Administrator**<br>Thien Nu Do<br>NRG Oncology<br>50 South 16th St, Suite 2800<br>Philadelphia, PA 19102<br>215-574-3174<br>dot@nrgoncology.org
**Head and Neck Cancer Committee Chair:**	Sue S. Yom, MD, PhD<br>University of California, San Francisco<br>San Francisco, CA<br>415-353-7175<br>sue.yom@ucsf.edu

Protocol Agent

**Agent**	**Supply**	**NSC #**	**IND #**	**IND Sponsor**
Durvalumab (MEDI4736)	CTEP/PMB	778709	134416	DCTD
Cetuximab	Commercial	714692		

Participating Sites (15-JAN-2019)

☒ U.S.
☒ Canada through CCTG
☐ Approved International Member Sites

Document History

	Version Date
**Amendment 7**	**March 09, 2022**

Amendment 6	January 13, 2021
Amendment 5	October 4, 2019
Amendment 4	August 21, 2019
Amendment 3	January 15, 2019
Amendment 2	July 5, 2018
Amendment 1	March 22, 2018
Initial	December 5, 2017

This protocol was designed and developed by NRG Oncology. It is intended to be used only in conjunction with institution-specific IRB approval for study entry. No other use or reproduction is authorized by NRG Oncology nor does NRG Oncology assume any responsibility for unauthorized use of this protocol.

NRG ONCOLOGY

NRG-HN004

Randomized Phase II/III Trial of Radiotherapy with Concurrent MEDI4736 (Durvalumab) vs. Radiotherapy with Concurrent Cetuximab in Patients with Locoregionally Advanced Head and Neck Cancer with a Contraindication to Cisplatin

**CONTACT INFORMATION** (09-MAR-2022)		
**For regulatory requirements:**	**For patient enrollments:**	**For data submission:**

Regulatory documentation must be submitted to the Cancer Trials Support Unit (CTSU) via the Regulatory Submission Portal.<br><br>(Sign in at https://www.ctsu.org, and select Regulatory > Regulatory Submission.)<br><br>Institutions with patients waiting that are unable to use the Portal should alert the CTSU Regulatory Office immediately by phone or email: 1-866-651-CTSU (2878), or CTSURegHelp@coccg.org to receive further instruction and support.<br><br>Contact the CTSU Regulatory Help Desk at 1-866-651-CTSU (2878) for regulatory assistance.	Refer to the patient enrollment section of the protocol for instructions on using the Oncology Patient Enrollment Network (OPEN). OPEN is accessed at https://www.ctsu.org/OPEN_SYSTEM/ or https://OPEN.ctsu.org.<br><br>Contact the CTSU Help Desk with any OPEN related questions by phone or email : 1-888-823-5923, or ctsucontact@westat.com.	Data collection for this study will be done exclusively through Medidata Rave. Refer to the data submission section of the protocol for further instructions.
The most current version of the **study protocol and all supporting documents** must be downloaded from the protocol-specific page located on the CTSU member's website (https://www.ctsu.org). Access to the CTSU members' website is managed through the Cancer Therapy and Evaluation Program - Identity and Access Management (CTEP-IAM) registration system and requires user log in with a CTEP-IAM username and password. Permission to view and download this protocol and its supporting documents is restricted and is based on person and site roster assignment housed in the CTSU Regulatory Support System (RSS).		
**For clinical questions (i.e. patient eligibility or treatment-related)** Contact the Study PI of the Lead Protocol Organization.		
**For non-clinical questions (i.e. unrelated to patient eligibility, treatment, or clinical data submission)** Contact the CTSU Help Desk by phone or email:<br>CTSU General Information Line – 1-888-823-5923, or ctsucontact@westat.com. All calls and correspondence will be triaged to the appropriate CTSU representative.		

TABLE OF CONTENTS

PHASE II/III SCHEMA 42
1. OBJECTIVES 44
1.1 Primary Objective 44
1.2 Secondary Objectives 44
1.3 Exploratory Objectives 44
2. BACKGROUND 44
2.1 Background of Head and Neck Cancer 44
2.2 Epidemiology, Treatment, and Outcomes for Medically Unfit HNC Patients 45
2.3 Comparative Effectiveness of Radiosensitizers in Elderly/Medically Unfit HNC Patients 45
2.4 Aging and the Immune System 46
2.5 Preclinical Data Supporting RT+ Anti-PD-L1 Therapy in HNC 46
2.6 Clinical Data Supporting RT and Anti-PD-1 Therapy in HNC 47
2.7 Clinical Data for Anti-PD-L1 Therapy [MEDI4736 (Durvalumab)] 47
2.8 Pharmacokinetic/Pharmacodynamic Data on MEDI4736 (durvalumab) and Anti-MEDI4736 Antibodies 48
2.9 Evidence-Based Standard of Care for HNC Patients Medically Unfit for Cisplatin 49
2.10 Correlative Science 49
3. PATIENT SELECTION, ELIGIBILITY, AND INELIGIBILITY CRITERIA 53

3.1 Patient Selection Guidelines 53
3.2 Eligibility Criteria 53
3.3 Ineligibility Criteria 56
4. REQUIREMENTS FOR STUDY ENTRY, TREATMENT, AND FOLLOW-UP 57
5. TREATMENT PLAN/REGIMEN DESCRIPTION 66
5.1 Systemic Therapy 66
5.2 Radiation Therapy 68
5.3 General Concomitant Medication and Supportive Care Guidelines 75
5.4 Duration of Therapy 76
6. TREATMENT MODIFICATIONS/MANAGEMENT 76
6.1 Dose Modifications and Toxicity Management Guidelines for Immune-Mediated, Infusion-Related, and Non-Immune Mediated Reactions for MEDI4736 (Durvalumab) 76
6.2 Dose Modification for Cetuximab 102
7. ADVERSE EVENTS REPORTING REQUIREMENTS 107
7.1 Protocol Agents 107
7.2 Adverse Events and Serious Adverse Events 107
7.3 Comprehensive Adverse Events and Potential Risks (CAEPR) List for 109
MEDI4736 (Durvalumab, NSC 778709) 109
7.4 Adverse Events for Commercial Study Agents: Cetuximab 113
7.5 Expedited Reporting of Adverse Events 113
7.6 Routine Reporting Requirements for Adverse Events 115
7.7 Pregnancy 115
8. REGISTRATION AND STUDY ENTRY PROCEDURES 115
8.1 CTEP Registration Procedures and Access requirements for OPEN, Medidata Rave, and TRIAD 115
8.2 CTSU Registration Procedures 117
8.3 RT-Specific Pre-Registration Requirements 119
8.4 Patient Enrollment 120
8.5 Medidata Patient Cloud ePRO Registration 121
9. DRUG INFORMATION 122
9.1 Investigational Study Agent: MEDI4736 (Durvalumab) 122
9.2 Commercial Agent: Cetuximab NSC# 714692 124
10. PATHOLOGY/BIOSPECIMEN 124
10.1 Biospecimen Submissions 124
10.2 Pre-hoc Central Review of Oropharyngeal and Unknown Primaries 125
10.3 Tissue Blocks for Central Review of PD-L1 and p16 status 126
10.4 Optional Specimen Submissions 127
11. SPECIAL STUDIES (NON-TISSUE) 130
11.1 Quality of Life Instruments Description 130
12. MODALITY REVIEWS 132
12.1 Radiation Therapy Quality Assurance Reviews 132
12.2 Medical Oncology Modality Quality Assurance Reviews 133
13. DATA AND RECORDS 133
13.1 Data Management/Collection 133
13.2 Data Quality Portal 134
13.3 Summary of Data Submission 135
13.4 Global Reporting/Monitoring 136
14. STATISTICAL CONSIDERATIONS 136
14.1 Study Design 136
14.2 Study Endpoints 136
14.3 Primary Objectives Study Design 137
14.4 Study Monitoring of Primary Objectives 139
14.5 Accrual/Study Duration Considerations 141
14.6 Secondary Endpoints 141
14.7 Exploratory Analyses 144
14.8 Gender/Ethnicity/Race Distribution 146

REFERENCES 148
APPENDIX I: CTEP Collaborative Agreements Language 153
APPENDIX II: PREPARATION, PROCESSING, AND SHIPPING OF PERIPERHAL BLOOD 155
APPENDIX III Medidata Patient Cloud Operational Instructions 156

NRG-HN004

Randomized Phase II/III Trial of Radiotherapy with Concurrent MEDI4736 (Durvalumab) vs. Radiotherapy with Concurrent Cetuximab in Patients with Locoregionally Advanced Head and Neck Cancer with a Contraindication to Cisplatin

PHASE II/III SCHEMA (15-JAN-2019)

STEP 1: REGISTRATION

For patients with oropharyngeal or unknown primaries:
p16 determination by immunohistochemistry confirmed by central pathology review prior to Step 2 Registration.

Note: For patients with oral cavity, laryngeal, and hypopharyngeal primaries,
[illegible]

STEP 2: REGISTRATION

STRATIFICATION

- AJCC 8th Edition stage (T0-3 and N0-2 vs. T4 and/or N3)
- Performance status/comorbidity (PS=0 and modified CCI* =0 vs. PS=1–2 and/or modified CCI > 0)
- Primary site (p16+ OPX/CUP vs. Other [Larynx, HPX, Oral Cavity, or p16- OPX/CUP])

*Modified CCI (Charlson Comorbidity Index) will be calculated excluding age, albumin, and cancer diagnosis (see Section 3).

RANDOMIZATION 1:2

ARM 1

RT + Cetuximab **

ARM 2

RT
+ MEDI4736 (durvalumab) **

**See Section 5.0 for radiation therapy and systemic therapy details.

**See Section 5.0 for radiation therapy and systemic therapy details.

NRG-HN004

Randomized Phase II/III Trial of Radiotherapy with Concurrent MEDI4736 (Durvalumab) vs. Radiotherapy with Concurrent Cetuximab in Patients with Locoregionally Advanced Head and Neck Cancer with a Contraindication to Cisplatin

LEAD-IN SCHEMA (15-JAN-2019)

Note: Please note that the lead-in phase has been completed and all relevant schema and dosing have been grayed out throughout the protocol.

STEP 1: REGISTRATION

For patients with oropharyngeal or unknown primaries:
p16 determination by immunohistochemistry confirmed by
central pathology review prior to Step 2 Registration.

Note: For patients with oral cavity, laryngeal, and hypopharyngeal primaries,
analysis of p16 status prior to Step 2 Registration is not required.

↓

STEP 2: REGISTRATION

INVESTIGATIONAL THERAPY

Two Weeks Prior to Radiation Therapy (RT): MEDI4736 (durvalumab) 1500 mg

Concurrent with RT (70 Gy over 7 weeks): MEDI4736 (durvalumab) 1500 mg on Weeks 2 and 6

Following RT: MEDI4736 (durvalumab) 1500 mg every 4 Weeks x 4 cycles

1. OBJECTIVES

1.1 Primary Objective

1.1.1 **Lead-In**: To determine the safety of radiotherapy (RT) with concurrent and adjuvant anti-PD-L1 therapy [MEDI4736 (durvalumab)] is safe in patients with locoregionally advanced head and neck cancer (HNC) who have a contraindication to cisplatin.

1.1.2 **Phase II:** To test the hypothesis that concurrent RT and anti-PD-L1 therapy improves PFS compared to standard therapy (RT with concurrent cetuximab) in patients with locoregionally advanced HNC who have a contraindication to cisplatin.

1.1.3 **Phase III:** To test the hypothesis that concurrent RT and anti-PD-L1 therapy improves overall survival compared to standard therapy (RT with concurrent cetuximab) in patients with locoregionally advanced HNC who have a contraindication to cisplatin.

1.2 Secondary Objectives

1.2.1 To compare toxicity using CTCAE and PRO-CTCAE between patients treated with RT + anti-PD-L1 therapy versus RT/cetuximab.

1.2.2 To test the effect of anti-PD-L1 therapy in the subpopulation of patients with tumors that overexpress PD-L1.

1.2.3 To compare overall survival, response (at 4-month FDG-PET-CT), locoregional failure, distant metastasis, and competing mortality in the two arms by known risk factors, including p16 status and ω score.

1.2.4 To test the hypothesis that MEDI4736 (durvalumab) therapy arm will have less decline in the physical function domain of European Organization for Research and Treatment of Cancer Core Questionnaire (EORTC QLQ-C30 Version 3.0) based on the change in score from baseline to 12 months from end of RT, compared to the cetuximab-RT arm in patients with locoregionally advanced HNC who have a contraindication to cisplatin.

1.2.5 To test the hypothesis that MEDI4736 (durvalumab) therapy arm at 1 year (from end of RT) will have less decline in swallowing related QOL using the MDADI total composite score, based on the change in score from baseline to 12 months from end of RT, compared to the cetuximab-RT arm in patients who are medically unfit for cisplatin.

1.2.6 To compare swallowing related performance and function short and long term using the PSS-HN.

1.2.7 To evaluate gastrostomy tube retention rates between arms.

1.3 Exploratory Objectives

1.3.1 To test the hypothesis that radiation combined with MEDI4736 (durvalumab) enhances the adaptive immune response using three types of immunophenotyping compared to radiation combined with cetuximab.

1.3.2 To compare overall QOL short term (end RT-8 months) and long term (12-24 months from end of RT) between arms using the EORTC QLQ-C30 Version 3.0/HN35.

1.3.3 To evaluate swallowing related QOL short term (end RT-8 months) and long term (12-24 months from end of RT) using the EORTC HN35 swallowing domain and MDADI (subscales) between arms in patients with locoregionally advanced HNC who have a contraindication to cisplatin.

1.3.4 To evaluate patient reported fatigue using the fatigue items in the EORTC QLQ and PRO-CTCAE.

1.3.5 To compare clinician and patient reported toxicity using CTCAE and PRO CTCAE.

1.3.6 To explore health utilities between cetuximab and MEDI4736 (durvalumab) RT using the EQ5D-5L.

2. BACKGROUND

2.1 Background of Head and Neck Cancer (15-JAN-2019)

Head and neck cancer (HNC) is among the most common malignancies in the United States, with globally more than 500,000 new cases and 350,500 deaths annually (WHO 2015). The majority of patients present with locoregionally advanced HNC, which is generally treated with multi-modality therapy consisting of radiation therapy (RT) and chemotherapy, with or without surgery. Despite advances in the therapy of HNC and the understanding of its causes (including human papillomavirus [HPV]), outcomes remain poor, with 5-year progression-free survival (PFS) generally less than 50%, excepting HPV-associated oropharyngeal cancer (Adelstein 2000, Ang 2010, Bernier 2004, Brizel 1998, Cooper 2004).

2.2 Epidemiology, Treatment, and Outcomes for Medically Unfit HNC Patients

Concurrent RT+cisplatin is considered the standard of care for many locoregionally advanced head/neck cancers (LRA-HNC). However, many LRA-HNC patients cannot receive cisplatin due to contraindications such as advanced age or comorbidity. More than 30% of the LRA-HNC population in the United States is older than age 65, approximately 32% of the population has at least moderate comorbidity, and 8% has severe comorbidity (Dansky-Ullmann 2012, Rose 2011). Dansky-Ullmann et al. (2012) reported that 38.7% of oral cavity, oropharynx, larynx, and hypopharynx cancer patients potentially eligible for chemotherapy and radiation receive either no chemotherapy or receive alternatives to cisplatin. An analysis of the LORHAN prospective HNC registry also showed that cisplatin was the concurrent agent in only 51% of patients. Thus, there is evidence that much of the HNC population cannot receive cisplatin, even if otherwise indicated.

Cisplatin is generally considered to be contraindicated due to toxicity and/or lack of efficacy in patients with poor organ function, performance status, pre-existing comorbidities, or advanced age (Ahn 2016). There is considerable evidence that patients older than 70 do not benefit from cisplatin (Pignon 2009, Kish 2015). However, alternative radiosensitizers have proven to be well-tolerated in such patients, albeit with uncertain efficacy. Outcomes with RT alone are poor. Newer therapies with promising efficacy and toxicity profiles are likely to be well-tolerated and could improve survival in this population. Patients with severe dysfunction are excluded because of their low likelihood of tolerating systemic therapy and high risk of competing mortality and treatment-related morbidity and mortality. Never-smokers with p16+ oropharynx primaries/localizing carcinoma of unknown primary (CUP) have a low probability of recurrence and may not benefit from radiosensitizers. The rationale for comorbidity measures come from several validated predictive models (Carmona 2016, Hurria 2005, Kallogjeri 2014, Pottel 2015).

Treatment options in medically unfit patients vary extensively from RT alone, to RT+carboplatin or RT+cetuximab. However, RT/cetuximab is the most widely accepted approach for treatment intensification in patients who are unfit for cisplatin (Ang 2012, Baxi 2014, Wong 2011). Cetuximab is a monoclonal antibody against the epidermal growth factor receptor (EGFR). Support for RT+cetuximab is based on a randomized trial indicating a survival advantage to RT+cetuximab over RT alone (Bonner 2006, 2010). In the LORHAN study, among patients who were not prescribed concurrent cisplatin, cetuximab was the next most commonly used regimen (21%), followed by carboplatin + paclitaxel (7%) (Wong 2011).

Outcomes for patients unable to receive cisplatin due to age or comorbidity are particularly poor (Siddiqui 2012). These patients frequently have organ dysfunction that alters their tolerance to aggressive chemotherapy, and they are at high risk for both cancer recurrence and competing mortality. Elderly and less healthy patients have been clearly under-represented in clinical trials; as such, clinical trial data frequently do not adequately convey the effectiveness of new therapies in these patients (Carmona 2016, Mell 2010, 2014, Rose 2011, Sarris 2014). Given the aging of the U.S. population and the increasing prevalence of HNC patients with comorbidities, it is widely recognized in the oncology community that there is an increased need for clinical trials representing and addressing the specific needs of this population (Wildiers 2013).

2.3 Comparative Effectiveness of Radiosensitizers in Elderly/Medically Unfit HNC Patients

Cetuximab is the only non-platinum radiosensitizing regimen with level I evidence supporting its use in HNC (Pfister 2015). The phase III randomized trial by Bonner et al. found that RT/cetuximab improved overall survival (hazard ratio [HR] 0.73, 95% CI 0.56-0.95; p=0.018) compared to RT alone in patients with locoregionally advanced HNC. Five-year overall survival was 45.6% in the RT + cetuximab group and 36.4% in the RT-alone group. Toxicity was tolerable in the RT + cetuximab arm.

Nonetheless, the effectiveness of cetuximab in the elderly/medically unfit population is controversial. The median age of patients receiving RT+cetuximab on the Bonner trial was 56 years. Of these patients, 110 (26%) were older than 65, and 135 (32%) patients had KPS < 90. On post-hoc analysis of both of these subpopulations, the effect of cetuximab was not statistically significant, in contrast to younger and healthier patients (Bonner 2010), but these findings are hypothesis-generating. Also, many patients with medical comorbidities were ineligible. In addition, RTOG 0522 did not find a benefit to the combination of RT with concurrent cisplatin and cetuximab compared to RT and cisplatin (Ang 2014). An Italian trial (Magrini

2016) also closed early with only 70 patients due to a higher number of treatment interruptions in the RT/cetuximab arm compared to RT with weekly cisplatin. However, subsequent studies—including a phase II trial—specifically support the safety and efficacy of RT/cetuximab in the elderly and/or medically unfit population (Alongi 2012, Jensen 2010). Unpublished data from RTOG 0920 and RTOG 1016 also have shown tolerability of RT/cetuximab in the elderly population. Therefore, RT+cetuximab is safe in this population, but its efficacy is unclear.

Given the paucity of clinical trials tailored toward the elderly/medically unfit population, it is prudent to further investigate the use of concurrent cetuximab in this population. Retrospective studies suggest that RT+cetuximab may be as effective as bolus cisplatin (Strom 2015) and that bolus cisplatin may be more effective than weekly cisplatin (Rades 2016). Among elderly patients in the LORHAN database, only 43% received RT with concurrent systemic therapy, with cetuximab the most common regimen (21%), followed by cisplatin (18%) and other (4%). Therefore, some elderly patients do receive cisplatin. However, there is considerable evidence from meta-analyses and population studies that even elderly patients healthy enough to receive cisplatin do not benefit from it (Pignon 2009, VanderWalde 2014, Kish 2015). A possible explanation is that small gains in efficacy with concurrent radiosensitizers are outweighed by adverse effects in patients who are more susceptible to treatment-related morbidity and mortality. This provides a strong rationale to investigate alternative regimens with the potential for heightened efficacy and reduced toxicity, such as immune checkpoint blockade.

Presently, there is no known subgroup of elderly patients who benefit from cytotoxic chemotherapy. Multivariable models incorporating complementary comorbidity indices such as the Charlson Comorbidity Index (CCI) and ACE-27 are known to be an effective approach for risk-stratifying this population according to survival (Kallogjeri 2014, Rose 2011). Additionally, nomograms exist to help predict patient's likelihood for high-grade toxicity from chemotherapy (Firat 2002, Hurria 2005, Pottel 2015, Miller 1992). Furthermore, recent evidence suggests that generalized competing event (GCE)-based risk-stratification methods could be used to identify groups most likely to benefit from treatment intensification in variety of malignancies, including head/neck cancer (Carmona 2016). These validated instruments can be useful in identifying subgroups of patients who are more/less likely to benefit from treatment intensification, but have been rarely implemented in clinical trial designs.

2.4 Aging and the Immune System

Combining standard RT with immune modulation is an attractive strategy in elderly patients, given their poorer anti-tumor T cell function and the favorable toxicity profile of emerging immunotherapy strategies, particularly PD1 and PD-L1 inhibitors. The importance of intact immune surveillance in controlling outgrowth of neoplastic transformation has been known for decades, and abundant evidence is available indicating that aging-associated dysfunction of host immunity plays an important role in the development of cancer. Multiple mechanisms related to aging are thought to contribute to the heightened risk for cancer. Functional variations of cellular T cell response have been shown to be age-related and predisposing to development of cancer (Haynes 2005). Other mechanisms, demonstrated in mouse models and humans, include decreases in dendritic cell number and function associated with aging (Sprecher 1990; Zavala 2006). In addition, there is considerable evidence to indicate that aging affects the immune system and anti-tumor response through distortions in T-cell maturation, function, proliferation, and receptor repertoire (Gottesman 1984, Haynes 2009, Lustgarten 2004, Mosley 1998, Thoman 1993).

Accumulating evidence shows a correlation between tumor-infiltrating lymphocytes (TILs) in cancer tissue and favorable prognosis in various malignancies. In particular, the presence of CD8+ T-cells and the ratio of CD8+ effector T-cells / FoxP3+ regulatory T-cells correlates with improved prognosis and long-term survival in many solid tumors.

2.5 Preclinical Data Supporting RT+ Anti-PD-L1 Therapy in HNC

The PD-1 receptor-ligand interaction is a major pathway hijacked by tumors to suppress immune control. The normal function of PD-1, expressed on the cell surface of activated T-cells under healthy conditions, is to down-modulate unwanted or excessive immune responses, including autoimmune reactions. PD-1 (encoded by the gene Pdcd1) is an Ig superfamily member related to CD28 and CTLA-4 which has been shown to negatively regulate antigen receptor signaling upon engagement of its ligands (PD-L1 and/or PD-

L2). PD-1 and family members are type I transmembrane glycoproteins containing an Ig Variable-type (V-type) domain responsible for ligand binding and a cytoplasmic tail which is responsible for the binding of signaling molecules. The cytoplasmic tail of PD-1 contains 2 tyrosine-based signaling motifs, an immunoreceptor tyrosine-based inhibition motif (ITIM) and an immunoreceptor tyrosine-based switch motif (ITSM). Following T-cell stimulation, PD-1 recruits the tyrosine phosphatases SHP-1 and SHP-2 to the ITSM motif within its cytoplasmic tail, leading to the dephosphorylation of effector molecules such as CD3ζ, PKCθ and ZAP70 which are involved in the CD3 T-cell signaling cascade.

The mechanism by which PD-1 down modulates T-cell responses is similar to, but distinct from that of CTLA-4 as both molecules regulate an overlapping set of signaling proteins. PD-1 is expressed on activated lymphocytes including peripheral CD4+ and CD8+ T-cells, B-cells, T regs and natural killer cells (MacFarlane 2014). Expression also has been shown during thymic development on CD4-CD8- (double negative) T-cells as well as subsets of macrophages and dendritic cells. The ligands for PD-1 (PD-L1 and PD-L2) are constitutively expressed or can be induced in a variety of cell types, including non-hematopoietic tissues as well as in various tumors. Both ligands are type I transmembrane receptors containing both IgV- and IgC-like domains in the extracellular region and contain short cytoplasmic regions with no known signaling motifs. Binding of either PD-1 ligand to PD-1 inhibits T-cell activation triggered through the T-cell receptor. PD-L1 is expressed at low levels on various non-hematopoietic tissues, most notably on vascular endothelium, whereas PD-L2 protein is only detectably expressed on antigen-presenting cells found in lymphoid tissue or chronic inflammatory environments. PD-L2 is thought to control immune T-cell activation in lymphoid organs, whereas PD-L1 serves to dampen unwarranted T-cell function in peripheral tissues. Although healthy organs express little (if any) PD-L1, a variety of cancers were demonstrated to express abundant levels of this T-cell inhibitor. This suggests that the PD-1/PD-L1 pathway plays a critical role in tumor immune evasion and should be considered as an attractive target for therapeutic intervention.

It has been observed in pre-clinical studies that HNC can arise in immune-privileged sites (Lyford-Pike 2013) and that PD-L1 expression is characteristically associated with virus-associated malignancies (Chen 2013). Multiple studies further indicate that immunotherapy, particularly PD-1:PD-L1 checkpoint blockade, acts synergistically with radiotherapy (Liang 2013, Sharabi, Lim 2015, Sharabi, Nirschl 2015, Tyman-Saint 2015, Zandberg 2014).

2.6 Clinical Data Supporting RT and Anti-PD-1 Therapy in HNC

There is considerable clinical evidence for the safety and effectiveness of PD-1:PD-L1 checkpoint inhibitors in patients with solid tumors, including elderly patients. Recently, a meta-analysis found that immune checkpoint inhibitors benefited both older and younger patients in improving overall survival across a spectrum of cancers (Nishijima 2016). The Checkmate-141 trial found a survival advantage with nivolumab compared to standard therapy in patients with recurrent/metastatic head/neck cancer (Ferris 2106). In this study, 68 patients over age 65 were treated with nivolumab and with no increased toxicity. The Keynote trial (Seiwert 2014) reported an 18% response rate with pembrolizumab in patients with recurrent head/neck cancer.

2.7 Clinical Data for Anti-PD-L1 Therapy [MEDI4736 (Durvalumab)]

MEDI4736 (durvalumab) is a human mAb of the immunoglobulin G1 kappa subclass that inhibits binding of PD-L1 with PD-1 on antigen-presenting cells and T-cells resulting in stimulation of the patient’s anti-tumor immune response. A fixed-dosing approach is preferred by the prescribing community and has been selected for the present trial due to ease of use and reduction of potential dosing errors. Given expectation of similar PK exposure and variability, it is considered feasible to utilize a fixed-dosing regimen. Based on an average body weight of 75 kg, a fixed dose of 750 mg q2 weeks of MEDI4736 (durvalumab) is equivalent to 10 mg/kg q2 weeks and 1500 mg q4 weeks of MEDI4736 (durvalumab) is equivalent to 20 mg/kg q4 weeks. MEDI4736 (durvalumab) is being studied in humans, as a single drug or in combination with other drugs, as part of ongoing clinical trials including patients with recurrent or metastatic HNC (D4190C00011, D4193C00001/HAWK, D4193C0003/CONDOR, D4193C0002/EAGLE).

The majority of the safety data currently available for MEDI4736 (durvalumab) are based on the first-time-

in-human, single-agent study (Study CD-ON-MEDI4736-1108) in patients with advanced solid tumors. In patients receiving MEDI4736 (durvalumab) at 10 mg/kg every 2 weeks, 71.8%) had at least 1 treatment-emergent adverse event (TEAE). The most frequently reported (10% of patients) TEAEs (all National Cancer Institute [NCI] Common Terminology Criteria for Adverse Event [CTCAE] grades) were fatigue, dyspnea, nausea, constipation, and decreased appetite. The important potential risks, based on the mechanism of action of MEDI4736 (durvalumab), as well as data from studies of relevant or similar therapies, include immune-mediated reactions, such as enterocolitis, dermatitis, hepatitis/hepatotoxicity, endocrinopathy, pneumonitis, neuropathy, and other events such as serious infections, infusion-related reactions, anaphylaxis or serious allergic reactions, and immune complex disease. The development of a serious infection is a theoretical risk based on findings from non-clinical safety studies. Thus far, no patients have experienced immune complex disease following exposure to MEDI4736 (durvalumab). Antidrug antibodies (ADAs) have been identified in 3 of the 31 patients tested.

Preliminary data from ongoing MEDI4736 HNC studies have indicated that MEDI4736 (durvalumab) is safe. Segal et al. (2015) presented on 62 patients in 2015, with mean age 58 (range: 24-96) treated with MEDI4736 (durvalumab). 86% were male, 64% were prior smokers, 60% were HPV negative or unknown HPV status. Grade ≥ 3 related AEs were reported in 7% of pts: rash (2 pts), and increased GGT, fatigue, and tumor inflammation (1 patient each). No drug-related AEs led to discontinuation or death. No colitis or grade ≥ 3 pneumonitis was observed. ORR was 12% (25% in PD-L1+ pts), and DCR at 24 weeks was 16% (25% in PD-L1+ pts). Median duration of response was not reached. The safety profile in SCCHN was manageable and responses were durable, with higher responses in PD-L1+ pts. The ongoing KESTREL trial is testing MEDI4736 (durvalumab) in the recurrent/metastatic population and no untoward toxicity has been observed to date (Seiwert 2016).

The current trial will have a lead-in component to verify the safety of RT with MEDI4736 (durvalumab) in this population. Based on the favorable toxicity profile of PD-1:PD-L1 checkpoint inhibitors in a wide variety of populations, the safety profile of RT/ MEDI4736 (durvalumab) is hypothesized to be as good or better than RT/cetuximab, which is already known to be safe in this population.

2.8 Pharmacokinetic/Pharmacodynamic Data on MEDI4736 (durvalumab) and Anti-MEDI4736 Antibodies (05-JUL-2018)

Based on available PK/pharmacodynamic data from ongoing Study 1108 with doses ranging from 0.1 to 10 mg/kg Q2W or 15 mg/kg Q3W, MEDI4736 (durvalumab) exhibited non-linear (dose-dependent) PK consistent with target-mediated drug disposition. The PK approached linearity at ≥3 mg/kg Q2W, suggesting near complete target saturation (membrane-bound and sPD-L1), and further shows that the MEDI4736 (durvalumab) dosing frequency can be adapted to a particular regimen given the linearity seen at doses higher than 3 mg/kg. The expected half-life with doses ≥3 mg/kg Q2W is approximately 21 days. A dose-dependent suppression in peripheral sPD-L1 was observed over the dose range studied, consistent with engagement of MEDI4736 (durvalumab) with PD-L1. A low level of immunogenicity has been observed. No patients have experienced immune-complex disease following exposure to MEDI4736 (durvalumab) (For further information on immunogenicity, please see the current IB).

Data from Study D4190C00006 [Phase I trial in NSCLC patients using the combination of MEDI4736 (durvalumab) and tremelimumab] also show an approximately dose-proportional increase in PK exposure for MEDI4736 (durvalumab) over the dose range of 3 to 20 mg/kg MEDI4736 (durvalumab) Q4W or Q2W. (For further information on PK observations in Study 006, please see the current IB).

The observed MEDI4736 (durvalumab) PK data from the combination study were well in line with the predicted monotherapy PK data (5th median and 95th percentiles) for a Q4W regimen.

A population PK model was developed using the data from Study 1108 (doses=0.1 to 10 mg/kg Q2W or 15 mg/kg Q3W (**Error! Reference source not found.**). Multiple simulations indicate that a similar overall exposure is expected following both 10 mg/kg Q2W and 20 mg/kg Q4W regimens, as represented by AUC_{ss} (4 weeks). Median $C_{max,ss}$ is expected to be higher with 20 mg/kg Q4W (~1.5-fold) and median $C_{trough,ss}$ is expected to be higher with 10 mg/kg Q2W (~1.25-fold). Clinical activity with the 20 mg/kg Q4W dosing regimen is anticipated to be consistent with 10 mg/kg Q2W with the proposed similar dose of 20 mg/kg Q4W

expected to (a) achieve complete target saturation in majority of patients; (b) account for anticipated variability in PK, pharmacodynamics, and clinical activity in diverse cancer populations; (c) maintain sufficient PK exposure in case of anti-drug antibody (ADA) impact; and (d) achieve PK exposure that yielded maximal antitumor activity in animal models.

Given the similar area under the plasma drug concentration-time curve (AUC) and modest differences in median peak and trough levels at steady state, the observation that both regimens maintain complete sPD-L1 suppression at trough, and the available clinical data, the 20 mg/kg Q4W and 10 mg/kg Q2W regimens are expected to have similar efficacy and safety profiles, supporting further development with a dose of 20 mg/kg Q4W. In summary, the dose and schedule of durvalumab in NRG-HN004 (1500 mg flat dosing, 3-week interval between dose 1 and 2, with an every-4-week interval after cycle 2) is supported by the above PK data.

2.9 Evidence-Based Standard of Care for HNC Patients Medically Unfit for Cisplatin

Outcomes for HNC patients who are medically unfit for cisplatin remain poor, and these patients have been historically under-represented in randomized trials. Furthermore, the aging of the U.S. population and rising prevalence (Zumsteg 2016) makes this an increasingly relevant problem. Evidence from clinical trials to support current community practice patterns is lacking. This would be the first trial to investigate PD-L1 inhibition in conjunction with radiotherapy in this population in an effort to define a standard of care, so the proposal is novel in this regard. This trial would also be the first in the U.S. to test the comparative effectiveness of immunotherapy versus EGFR therapy in this population.

A fundamental question this study will address is whether treatment intensification is a potential strategy to improve outcomes in head/neck cancer patients with a contraindication to cisplatin, or whether, regardless of efficacy, treatment intensification is unlikely to be successful. It is possible that existing treatment intensification strategies are effective but too toxic to be beneficial to this population in the net, but less toxic strategies such as immunotherapy could be. On the other hand, it is possible that any treatment, regardless of its effectiveness and toxicity profile, would be unable to augment survival due to the mortality risk posed by competing comorbidities. These answers are not knowable outside of conducting a clinical trial that specifically addresses this population.

2.10 Correlative Science (21-AUG-2019)

Biology

This study aims to incorporate correlative translational science to identify subpopulations that may selectively benefit from RT + immunotherapy. The study will provide novel insights into the effects of immunotherapy on both tumor and the immune system in this unique population, and provide an initial evaluation of the comparative impact of immunotherapy versus EGF-R inhibition on toxicity. PFS is known to be a good surrogate endpoint for overall survival in HNC (Michiels 2009), and the high rate of events in this population leads to a fast readout of trial results. Heterogeneity in this population is an issue, as variation in medical comorbidities, p16 status, smoking history, and other known prognostic factors differentially affect treatment outcomes. The trial results may help identify subgroups that derive a selective benefit (e.g., patients with PD-L1 overexpressing tumors, p16-negative tumors), for which a phase III study could be specifically tailored. Collection of blood samples will allow us to examine the effects of tumor biomarkers such as T-cell infiltration and CD8 expression on the therapeutic effect in an elderly population.

Multiple studies, including RTOG studies, have established that p16 is a prognostic biomarker in non-oropharyngeal HNSCC (Chung 2014, Alsidawi 2017, Wookey 2017). However, other studies have not found HPV status to be correlated with outcomes in HNSCC (Fakhry 2017). Therefore, the prognostic role of p16/HPV status in non-oropharyngeal cancer is unclear. This study will be among the first NRG Oncology head and neck cancer trials to collect p16 status systematically on patients with both oropharyngeal and nonoropharyngeal tumors and will lend further insight into the prognostic role of p16 status in this population.

Competing Risks

Competing events such as cancer recurrence and mortality, treatment-related morbidity and mortality, and

intercurrent non-cancer mortality are well-known to complicate the interpretation of treatment effects. Carmona et al. (2016) showed that GCE models significantly improve the ability to risk-stratify elderly patients at risk for competing events and better identify sub-populations who are likely to selectively benefit from treatment intensification. Essentially, the GCE approach identifies subsets of patients who are more likely to experience a cancer event (e.g. recurrence or mortality) than a competing event (e.g., non-cancer death). By definition, a patient with a ω ratio < 0.5 has a greater hazard for non-cancer mortality than for cancer mortality. A review of RTOG trials 9003, 0522, and 0129 shows that GCE is effective in stratifying patients age 65 years or older into competing event strata (Figure 1). In particular, studies have found that patients with a predicted ω ratio (i.e., ω score) ≥ 0.80 selectively benefit from intensive treatment (Mell 2019, Zakeri 2018). Various modeling approaches and nomograms have been used to develop ω scores. The model used in this trial is based on a nomogram developed from the updated MARCH meta-analysis (Lacas 2017). Data from the current trial will provide the first opportunity to test the GCE approach prospectively within a cooperative group trial.

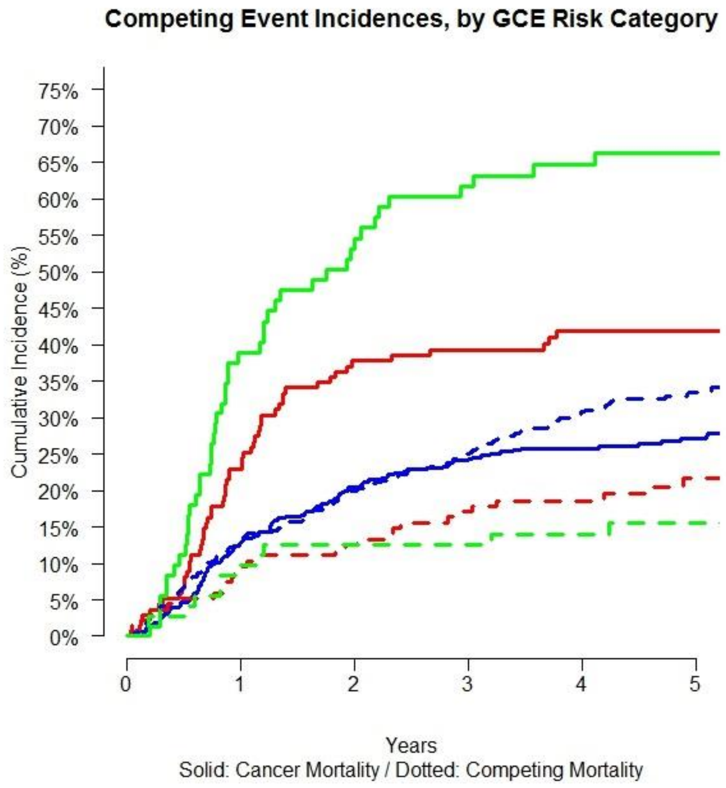

Figure 1. Stratification of patients older than 65 from RTOG 9003, 0522, and 0129 using a Generalized Competing Event (GCE) model. Solid lines represent cancer mortality, and dotted lines represent competing causes of mortality. "Low-risk" patients (blue) have comparatively lower incidence of cancer mortality relative to competing mortality versus that for the "intermediate-risk" (red) and "high-risk" (green) patients.

Supportive Care
The trial will also collect further evidence to support the use of nomograms to predict toxicity, and could validate the use of such tools in the community.

Quality of Life
Health-related quality of life (HR-QOL) is a complex multidimensional concept describing the patient's perception of the effects of disease and treatment on their physical, functional, psychological, emotional, and social well-being, in context of their culture and environment. It is an important endpoint on clinical trials to compare treatment arms

and understand its effects from the patient's perspective. While physician-reported toxicity may detect and grade adverse clinical events, it fails to provide the patient's perspective on how a treatment affects the patient. When comparing different treatments with potentially contrasting toxicity profiles, patient reported outcomes (PROs) may be invaluable in determining the best treatment arm combined with disease control, and survival endpoints.

Evaluation of HR-QOL in Elderly Patients with Multiple Co-Morbidities

Evaluation of HR-QOL in locally advanced HNSCC patients who are not candidates for cisplatin therapy represents a unique opportunity to study patients with multiple co-morbidities and/or elderly vulnerable patients. Since the effects of MEDI4736 (durvalumab) immunotherapy combined RT in patients with HNSCC who have a contraindication to cisplatin on HR-QOL are currently unknown, this study will provide a unique and critical opportunity to characterize the effects of this novel combination treatment on HR-QOL.

The primary QOL endpoint, the null hypothesis (H_0) states that there is no difference in mean change in QOL physical function domain of EORTC QLQ from baseline to 1 year (from end of RT) between MEDI4736-RT and cetuximab-RT arms. Alternative hypothesis (H_A) states that there is a clinically meaningful difference in QOL physical function domain of EORTC QLQ (change from baseline) between MEDI4736-RT and cetuximab-RT arm, whereby MEDI4736 will have a less decline in QOL compared to the cetuximab arm at one year (from end of RT). A greater than 10-point difference in mean change in score between arms is considered a minimum important change. A 10-point change on a 0-100 points scale in physical functioning QOL domain of the EORTC QLQ 30 is considered clinically meaningful. Exploratory analyses also will evaluate other domains of the EORTC QLQ/HN35 in the short term (end of RT-8 months) and long term (12-24 months) between arms.

Specific to HNSCC, patients undergoing chemoradiation experience significant distress from a constellation of symptoms including acute nausea, alterations in taste and smell, mucositis leading to significant oral pain, odynophagia, and malnutrition and in the long term, consequential late sequelae, such as xerostomia and dysphagia, may have functional consequences for dental health, normalcy of diet and nutrition, and ability to socialize and eat in public (List et al., 1997; List et al., 1999). It is important to recognize that symptoms from cancer therapy are often found in clusters, and measuring a symptom in isolation may not provide the full picture of the burden of treatment or disease on the patient. Studies have shown that CRT for patients with HNC have been found to be associated with two stable symptom clusters: head and neck-specific symptoms (radiodermatitis, dysphagia, radiomucositis, dry mouth, pain, taste disturbance, and fatigue), and a second cluster including gastrointestinal side effects (nausea, vomiting, and dehydration) (Xiao 2013). In a National Cancer Institute Symptom Management and QOL Clinical Trials Planning Meeting, investigators identified ten HNC-specific core symptoms and two HR-QOL domains, including swallowing, oral pain, skin changes, dry mouth, dental health, opening mouth/trismus, taste, excess/thick mucus/saliva, shoulder disability/motion, voice/hoarseness, social domain, and functional domain which were deemed important for evaluation on future clinical HNC trials (Chera 2014). Based on these recommendations we will evaluate the HNC specific core symptoms using the HN specific items in the EORTC H&N35 module, as well as evaluating symptom specific instruments, including the MDADI (MD Anderson Dysphagia Inventory), to evaluate swallowing-related QOL, which are important core symptoms in HNC treatment.

Fatigue in HNSCC Related to IMRT and MEDI4736 (durvalumab)

In head and neck cancer, fatigue is one of the most common and distressing symptoms affecting up to 75% of patients, where the majority of patients' experience fatigue usually peaking during the first and second weeks after completion of CRT and that can remain higher than baseline in approximately half of these patients long term (Spratt 2012). The underlying etiology of fatigue in head and neck cancer patients is thought to be multifactorial. Dosimetric factors have been implicated in patients undergoing IMRT due to increase mean and maximum doses to central nervous system structures. The PARSPORT (CRUK/03/005) trial demonstrated a statistically significant increase in acute clinician-graded fatigue in patients who were treated with IMRT as compared to 3DCRT. The investigators attributed this finding to dosimetric central nervous system (CNS) factors. Doses to the posterior fossa, brainstem, and cerebellum have been correlated to grade 2 or higher acute fatigue (Gulliford, 2012. Another study identified dosimetric variables to the brainstem, cerebellum, pituitary gland, pineal gland, hypothalamus, hippocampus, and basal ganglia being associated with increased grade 2 or higher fatigue, hypothesizing that the possible radiation-induced fatigue may be caused by pituitary-related hormonal function or disruption of connections between these critical CNS structures (Powell, 2014). Patient reported fatigue has been demonstrated in up to 75% of patients receiving radiation for HNC (Niska et al., 2017). More recent datasets have shown that using PROs for measuring fatigue may have greater predictive accuracy than clinician scores alone (Quinten et al., 2011). Furthermore, fatigue is one of the frequently reported (10% of patients) treatment-emergent adverse event (TEAE)s from MEDI4736 (durvalumab). Hence, the combined effect of IMRT and MEDI4736 (durvalumab) on patient-

reported fatigue warrants study. Hence an exploratory objective is to evaluate patient reported fatigue using the fatigue items in the EORTC QLQ and fatigue item in the PRO-CTCAE in patients receiving MEDI4736 (durvalumab)+ RT using IMRT for HNSCC. The hypothesis is that short term fatigue up to 4 months in HNSCC patients receiving MEDI4736 (durvalumab) + RT is caused by immune mediated effects combined with RT dose volume factors to the CNS organs at risk (OARs). A reduction of incidence/severity of fatigue is achieved by optimal dosing of MEDI4736 (durvalumab) with optimal IMRT by reduced dose/volume to CNS OARs.

Swallowing-Related QOL with MEDI4736 (Durvalumab) + RT

Oropharyngeal dysphagia after HNC radiotherapy is a major treatment related morbidity, which may have devastating consequences on function and QOL in HNC survivors. Furthermore, the functional morbidity of dysphagia after multimodality treatment may contribute to non-cancer deaths, such as complications from malnutrition and aspiration, which may offset any survival gains from treatment intensification (Cooper 2012; Forastiere 2013). Multiple factors are thought to contribute to the severity of oropharyngeal dysphagia including radiation dosimetric factors (such as the mean dose and volume to the larynx and pharyngeal constrictors), and the addition of concurrent chemotherapy or biologic therapy. Poor swallowing outcomes after HNC radiotherapy have been attributed to higher mean radiation dose to the swallowing muscles including the pharyngeal constrictors, glottis, supraglottic larynx, and esophagus (Feng et al., 2007). Swallowing outcomes have been assessed by patient reported measures, clinician graded toxicity, percutaneous endoscopic gastrostomy tube rates, and objective measures such as videofluoroscopy (Eisbruch et al., 2011; Vainshtein et al., 2015). Using IMRT to spare the pharyngeal constrictors, larynx, cervical esophagus, and parotid glands have resulted in improved objective and patient reported swallowing in single institutional series (Vainshtein et al., 2015). HR-QOL after IMRT is generally stable or improved at two years, and new late toxicities beyond two years are uncommon. Eleven percent and 14% of patients reported ‘moderate’ or ‘severe’ long-term worsening in HNQOL pain and overall bother domains, respectively, which were shown to be associated with higher mean radiation dose to the cervical esophagus, larynx, and pharyngeal constrictors (Vainshtein et al., 2015). Bhide et al. retrospectively evaluated superior, middle, and inferior pharyngeal constrictor, and supra-hyoid muscle radiation dose in 50 patients with HNC treated with induction chemotherapy followed by CRT (Bhide 2009). One-year clinician graded swallowing toxicity and swallowing PROs using the MDADI was available for 37 patients. There was a significant correlation between observer-rated RTOG graded dysphagia and all MDADI parameters. Levendag et al. examined the relationship between pharyngeal constrictor radiation dose, observer reported dysphagia, and patient reported swallowing using the EORTC-QLQ/H&N35 and MDADI, in 81 patients with OPC treated with CRT using both IMRT and 3DCRT (Levendag et al., 2007). A dose-effect relationship was found with an increase in the probability of clinician graded dysphagia of 19% for every 10 Gy increase in mean radiation dose above 55 Gy to the superior and middle pharyngeal constrictors. At a mean follow-up of 18 months, RTOG Grade 3 and 4 dysphagia (determined from chart review) was found in 23% of patients, and poor swallowing outcomes reflected by a total MDADI score of ≤ 50 was found in 26% of patients. A significant correlation was also noted between observer-rated grade 3 and 4 dysphagia and MDADI total scores, such that MDADI scores of ≤ 50 were considered equivalent to clinician graded 3 or 4 dysphagia. In addition, the scores of several EORTC-QLQ/H&N35 items (question 35=swallowing liquids, 36 =pureed, 37=solids) and the general MDADI was found to be associated with the superior and middle constrictor radiation dose, and the scores of EORTC-QLQ/H&N35 questions 37=swallowing solids and 38=aspiration were associated with radiation dose to the inferior constrictor. The most commonly used validated PRO swallowing instrument is the MD Anderson Dysphagia Inventory (MDADI), a 20-item self-administered patient reported dysphagia-specific instrument consisting of global, emotional, functional, and physical subscales. The MDADI has been validated in patients with HNC in single institution series (Chen 2001), and is currently being used on several prospective multicenter randomized clinical trials. The MDADI total score for clinically acceptable swallowing is defined as mean total MDADI score ≥ 60. The secondary QOL objective is to compare swallowing related QOL between arms and it is hypothesized that the MEDI4736-RT will have less swallowing QOL decline at 1-year (from end of RT) swallowing QOL (mean change from baseline) compared to the cetuximab arm. The Null Hypothesis states that is that there is no difference in swallowing related QOL decline between arms. The swallowing QOL endpoint will measure the mean individual change in total composite MDADI score at 1 year (from end of RT) from baseline in each arm. One of the arms will be deemed inferior to the other if there is a mean change score of ≥ 10 points from baseline for that arm and there is a ≥ 10 point difference between the 2 arms that is statistically significant. The minimum important difference (MID) is defined as the smallest difference in score in the domain of interest that patients perceive as important, that would lead to consider a change in the patient’s management (King 2011). The MID should be within 5 to 10% of the instrument range (Ringash 2007). Since the MDADI score ranges from 0-100, a change of 5- 10% (ie. 5-10 points) in the score may be considered meaningful. Hutcheson recently

published proposed MID for the MDADI to be 10 points (Hutcheson 2016). Exploratory analyses will evaluate the percentage of patients in each arm with poor swallowing QOL, defined as an individual patient MDADI score of < 60 at 1 year from the end of RT and will also evaluate the subscale scores of the MDADI inventory, particularly the physical and functional subscales at 1 year (end of RT) and the EORTC HN35 swallowing domain.

The effects of MEDI4736 (durvalumab) immunotherapy combined with IMRT in HNSCC on swallowing-related QOL are unknown. Particularly in this trial which includes laryngeal and hypopharyngeal HNSCC patients, patients with multiple co-morbidities who are at greater risk for dysphagia, understanding the impact dysphagia on long term swallowing-related QOL and survival in this patient population will be important understand on this trial.

QOL and PRO-CTCAE will be measured during the phase II and III portion of the study.

3. PATIENT SELECTION, ELIGIBILITY, AND INELIGIBILITY CRITERIA

Note: Per NCI guidelines, exceptions to inclusion and exclusion criteria are not permitted.

For questions concerning eligibility, please contact the Biostatistical/Data Management Center (see protocol cover page). For radiation therapy-related eligibility questions, please contact RTQA (see protocol cover page).

3.1 Patient Selection Guidelines

3.1.1 Patients must have the psychological ability and general health that permits completion of the study requirements and required follow up.

3.1.2 Women of childbearing potential and men who are sexually active should be willing and able to use medically acceptable forms of contraception during therapy and for 6 months following the last dose of cetuximab or MEDI14736 (durvalumab).

3.1.3 Submission of H&E stained slides and formalin-fixed and paraffin-embedded (FFPE) tissue block (or punch biopsy of FFPE block) to the Biospecimen Bank at UCSF for central review for oropharyngeal and unknown primaries and for p16 analysis for all other non-oropharyngeal primaries is mandatory for all patients. *Investigators should check with their pathology department regarding release of biospecimens before approaching patients about participation in the trial. (See details in Sections 3.2 and 10)*

3.2 Eligibility Criteria (04-OCT-2019)

A patient cannot be considered eligible for this study unless ALL of the following conditions are met.

<u>Prior to Step 1 Registration</u>

3.2.1 Patients must have pathologically confirmed, previously untreated, unresected squamous cell carcinoma of the larynx, hypopharynx, oropharynx, oral cavity, or carcinoma of unknown head/neck primary prior to Step 1 registration. Submission of H&E stained slides and formalin-fixed and paraffin-embedded (FFPE) tissue block (or punch biopsy of FFPE block) to the Biospecimen Bank at UCSF for central review for oropharyngeal and unknown primaries and for p16 analysis for all other non- oropharyngeal primaries is mandatory for all patients. For oropharyngeal and unknown primaries, submission of H&E and p16 stained slides (with the required block for PD-L1) to the Biospecimen Bank at UCSF for central review is also required ***prior to Step 2 registration*** (See Section 10 for further criteria for central pathology review).

Note: Fine needle aspirates (FNA) samples are not acceptable since they do not provide enough material for PD-L1 and p16 testing. However, if a cell block derived from the FNA is available, it is allowable if there are sufficient cells present in the block for PD-L1 testing. Dr. Jordan will determine this upon receipt. For sites submitting FNA cell blocks for ALL patients they must do so within 7-10 business days from registering the patient. Sites must confirm with their cytology/pathology labs to make sure they can provide the required material as the bank must be able to retain these samples for the mandatory testing.

3.2.2 Patients must have locoregionally advanced HNSCC (see table below)

- for p16-positive oropharyngeal/unknown primaries, AJCC 8th edition stage III and selected stage I-II based on smoking status in pack-years

- for laryngeal, hypopharyngeal, and oral cavity primaries and p16-negative oropharyngeal/unknown primaries, AJCC 8th edition stage III-IVB

Based on the following minimum diagnostic workup within 60 days prior to Step 1 registration:

- General history and physical examination by a Radiation Oncologist or Medical Oncologist or ENT Physician or Head & Neck Surgeon
- For larynx, hypopharynx, and base of tongue primaries, a laryngopharyngoscopy (mirror or fiberoptic or direct procedure) is required, unless the patient cannot tolerate or refuses
- Imaging of the head and neck with a neck CT or MRI (with contrast, unless contraindicated) or PET/CT. Note that the CT portion of the PET/CT must be of diagnostic quality, including contrast administration unless contraindicated. If the CT portion of the PET/CT study is low-dose (non-diagnostic), then an additional CT or MRI study with contrast (unless contraindicated) is required.
- Chest imaging: Chest CT with or without contrast (unless contraindicated) or PET/CT

**p16-Positive Oropharyngeal or Unknown Primaries**		
**AJCC 8th Edition TNM**	**AJCC 8th Edition Stage**	**Pack-Years***
T0-2 N1 M0	I	> 10
T0-2 N2 M0	II	Any
T3 N0-1 M0	II	> 10
T3 N2 M0	II	Any
T0-3 N3 M0	III	Any
T4 N0-3 M0	III	Any
**Laryngeal, Hypopharyngeal, Oral Cavity, and p16-Negative Oropharyngeal or Unknown Primaries**		
**AJCC 8th Edition TNM**	**AJCC 8th Edition Stage**	
T3-4b N0 M0	III-IVB	
T0-4b N1-3 M0	III-IVB	

*Note: Twenty cigarettes is considered equivalent to one pack.

3.2.3 Patients must have a contraindication to cisplatin as defined in the following bullet points. Sites must complete the online tool at comogram.org prior to Step 1 registration to determine if the patient is eligible. The scores must be recorded on a CRF. (Refer to data submission table on the NRG-HN004 protocol page on the CTSU website).

- Age ≥ 70 with moderate to severe comorbidity or vulnerability to cisplatin, defined as having **one or more** of the following conditions within 30 days prior to Step 1 registration:
  - Modified Charlson Comorbidity Index ≥ 1
  - ACE-27 Index ≥ 1
  - ω score < 0.80
  - G-8 score ≤ 14
  - CARG Toxicity Score ≥ 30%
  - CIRS-G Score ≥ 4

—OR—

- Age < 70 with severe comorbidity or vulnerability to cisplatin, defined as having **two or more** of the following conditions within 30 days prior to Step 1 registration
  - Modified Charlson Comorbidity Index ≥ 1
  - ACE-27 Index ≥ 1
  - ω score < 0.80

- G-8 score ≤ 14
- CARG Toxicity Score ≥ 30%
- CIRS-G Score ≥ 4

—OR—

- Age ≥ 18 with an absolute or relative contraindication to cisplatin, defined as **one or more** of the following within 30 days prior to Step 1 registration:
  - Creatinine clearance (CC) > 30 and < 60 cc/min
    For this calculation, use the Cockroft-Gault formula:
    CC = 0.85 (if female)* ((140–Age) / (Serum Creatinine)) * (Weight in kg / 72)
    Patient must be greater than 18 years old
  - Zubrod performance status 2 prior to Step 1 registration
  - Pre-existing peripheral neuropathy grade ≥ 1
  - History of hearing loss, defined as either:
    - Existing need of a hearing aid OR
    - ≥ 25 decibel shift over 2 contiguous frequencies on a pretreatment hearing test as clinically indicated

3.2.4 Adequate hematologic function within 14 days prior to Step 1 registration defined as follows:
- Absolute neutrophil count (ANC) ≥ 1,000 cells/mm^3
- Platelets ≥ 100,000 cells/mm^3
- Hemoglobin ≥ 9.0 g/dl (Note: The use of transfusion or other intervention to achieve Hgb ≥ 9.0 g/dl is acceptable).

3.2.5 Adequate hepatic and renal function within 14 days prior to Step 1 registration defined as follows:
- AST or ALT ≤ 2.5 times institutional upper limit of normal
- Serum bilirubin ≤ 1.5 x institutional upper limit of normal
- Measured creatinine clearance (CL) > 30 mL/min or Calculated creatinine CL > 30 mL/min by the Cockcroft-Gault formula (Cockcroft and Gault 1976) or by 24-hour urine collection for determination of creatinine clearance
  Cockcroft-Gault formula:
  CC = 0.85 (if female)* ((140–Age) / (Serum Creatinine)) * (Weight in kg / 72)

3.2.6 For women of childbearing potential, a negative serum or urine pregnancy test within 14 days prior to Step 1 registration.
Note: Women will be considered post-menopausal if they have been amenorrheic for 12 months without an alternative medical cause. The following age-specific requirements apply:
- Women < 50 years of age would be considered post-menopausal if they have been amenorrheic for 12 months or more following cessation of exogenous hormonal treatments and if they have luteinizing hormone and follicle-stimulating hormone levels in the post-menopausal range for the institution or underwent surgical sterilization (bilateral oophorectomy or hysterectomy).
- Women ≥ 50 years of age would be considered post-menopausal if they have been amenorrheic for 12 months or more following cessation of all exogenous hormonal treatments, had radiation-induced menopause with last menses >1 year ago, had chemotherapy-induced menopause with last menses >1 year ago, or underwent surgical sterilization (bilateral oophorectomy, bilateral salpingectomy or hysterectomy).

3.2.7 The patient or a legally authorized representative must provide study-specific informed consent prior to Step 1 registration.

Prior to Step 2 Registration (for patients with oropharyngeal and unknown primaries)

3.2.8 For patients with oropharyngeal or unknown primaries: p16 determination by immunohistochemistry (defined as greater than 70% strong nuclear or nuclear and cytoplasmic staining of tumor cells), confirmed by central pathology review; (see Section 10.1 for details).
Note: For patients with oral cavity, laryngeal, and hypopharyngeal primaries, analysis of p16 status prior to Step 2 registration/randomization is not required (p16 status will be analyzed centrally post-hoc). Step 2 registration for these patients can be completed after Step 1 registration.

3.3 Ineligibility Criteria (13-JAN-2021)

Patients with any of the following conditions are NOT eligible for this study.

<u>Prior to Step 1 Registration</u>

3.3.1 Prior invasive malignancy within the past 3 years (except for non-melanomatous skin cancer, and early stage treated prostate cancer); synchronous head and neck primaries are ineligible

3.3.2 Prior radiotherapy to the region of the study cancer that would result in overlap of radiation therapy fields
Note: Prior external beam radiotherapy is excluded, but Iodine 131 is allowed.

3.3.3 Prior immunotherapy

3.3.4 Prior systemic therapy, including cytotoxic chemotherapy, biologic/targeted therapy, or immune therapy for the study cancer

3.3.5 Major surgery within 28 days prior to Step 1 registration

3.3.6 Proven evidence of distant metastases

3.3.7 If both of the following conditions are present, the patient is ineligible:

- ≤ 10 pack-year smoking history
- p16-positive carcinoma of the oropharynx or unknown primary that are T0-3, N0-1 (AJCC 8th Edition)

Note: In the event that a registered patient with ≤ 10 pack-years has a p16-positive result on central review with the tumor and nodal stage T0-3, N0-1 (AJCC 8th Edition), then the site will be notified that the patient is ineligible.

3.3.8 Age < 18 years

3.3.9 Zubrod performance status ≥ 3

3.3.10 Body weight ≤ 30 kg

3.3.11 Patients with oral cavity cancer are excluded from participation if the patient is medically operable and resection of the primary tumor is considered technically feasible by an oral or head and neck cancers surgical subspecialist. *(Please consult the Surgical Oncology Co-PI, Steven Chang, MD, if clarification is needed on an individual case.)*

3.3.12 Any of the following severe laboratory abnormalities within 14 days of Step 1 registration, unless corrected prior to Step 1 registration:

- Sodium < 130 mmol/L or > 155 mmol/L
- Potassium < 3.5 mmol/L or > 6 mmol/L
- Fasting glucose < 40 mg/dl or > 400 mg/dl
- Serum calcium (ionized or adjusted for albumin) < 7 mg/dl or > 12.5 mg/dl
- Magnesium < 0.9 mg/dl or > 3 mg/dl

3.3.13 Unstable angina and/or congestive heart failure requiring hospitalization within 3 months prior to Step 1 registration

3.3.14 Transmural myocardial infarction within 3 months prior to Step 1 registration

3.3.15 Respiratory illness requiring hospitalization at the time of Step 1 registration
Note: If the respiratory illness is resolved and the patient meets the eligibility status above, then the patient can be considered for the trial.

3.3.16 Idiopathic pulmonary fibrosis or other severe interstitial lung disease that requires oxygen therapy or is thought to require oxygen therapy within 1 year prior to Step 1 registration

3.3.17 History of (non-infectious) pneumonitis that required steroids or current pneumonitis

3.3.18 Clinically apparent jaundice and/or known coagulation defects

3.3.19 Active or prior documented autoimmune or inflammatory disorders (including inflammatory bowel disease [e.g., colitis or Crohn's disease], diverticulitis [with the exception of diverticulosis], systemic lupus erythematosus, Sarcoidosis syndrome, or Wegener syndrome [granulomatosis with polyangiitis], Graves'

disease, rheumatoid arthritis, hypophysitis, uveitis, etc.).

The following are exceptions to this criterion:

- Patients with vitiligo or alopecia;
- Patients with hypothyroidism (e.g., following Hashimoto syndrome) stable on hormone replacement;
- Any chronic skin condition that does not require systemic therapy;
- Patients without active disease in the last 5 years may be included but only after consultation with the medical oncology study chair;
- Patients with celiac disease controlled by diet alone.

3.3.20 History of active primary immunodeficiency including, but not limited to Acquired Immune Deficiency Syndrome (AIDS) based upon current CDC definition; Note: HIV testing is not required for entry into this protocol. The need to exclude patients with AIDS from this protocol is necessary because the treatment involved in this protocol may be immunosuppressive. Patients with known HIV, CD4 counts ≥ 200/μL, and undetectable viral loads who are stable on an antiretroviral regimen may be included.

3.3.21 Current or prior use of immunosuppressive medication within 14 days before Step 1 registration, with the exceptions of intranasal and inhaled corticosteroids or systemic corticosteroids at physiological doses, which are not to exceed 10 mg/day of prednisone, or an equivalent corticosteroid

3.3.22 Receipt of live attenuated vaccination within 30 days prior to Step 1 registration

3.3.23 Medical or psychiatric illness which would compromise the patient's ability to tolerate treatment or limit compliance with study requirements

3.3.24 Pregnancy or women of childbearing potential and men who are sexually active and not willing/able to use medically acceptable forms of contraception during treatment and for 6 months after the last dose of cetuximab or MEDI14736 (durvalumab); this exclusion is necessary because the treatment involved in this study may be significantly teratogenic. Women who are breastfeeding are also excluded.

3.3.25 Prior allergic reaction or hypersensitivity to cetuximab or MEDI4736 (durvalumab) or any of study drug excipients.

3.3.26 History of allogenic organ transplantation

3.3.27 Uncontrolled hypertension

3.3.28 Uncontrolled cardiac arrhythmia

3.3.29 Uncontrolled serious chronic gastrointestinal condition associated with diarrhea

3.3.30 Active infection including tuberculosis (clinical evaluation that includes clinical history, physical examination and radiographic findings, and TB testing in line with local practice), hepatitis B (known positive HBV surface antigen (HBsAg) result), hepatitis C. Patients with a past or resolved HBV infection (defined as the presence of hepatitis B core antibody [anti-HBc] and absence of HBsAg) are eligible. Patients positive for hepatitis C (HCV) antibody are eligible only if polymerase chain reaction is negative for HCV RNA.

4. REQUIREMENTS FOR STUDY ENTRY, TREATMENT, AND FOLLOW-UP

(13-JAN-2021)

PRE-TREATMENT ASSESSMENTS

**Assessments**	**Calendar Days Prior to Step 1 Registration**				**Calendar Days Prior to Treatment, inclusive of Treatment Day 1**
		**≤60**	**≤30**	**≤14**	
Pathologic confirmation of previously untreated, unresected squamous cell carcinoma of larynx, hypopharynx, oropharynx, oral cavity, or carcinoma of unknown head/neck primary	X				
General history and physical examination by a Radiation Oncologist or Medical Oncologist or ENT Physician or Head &		X			≤14

Neck Surgeon; evaluation must include a listing of concomitant medications.					
For larynx, hypopharynx, and base of tongue primaries, a laryngopharyngoscopy (mirror or fiberoptic or direct procedure)		X*			
Imaging of the head and neck with a neck CT or MRI (with contrast, unless contraindicated) or PET/CT (CT portion of the PET/CT must be of diagnostic quality with contrast, unless contraindicated. See section 3.2.2 for details.)		X			
Chest imaging with CT (with or without contrast, unless contraindicated) or PET/CT		X			
Charlson Comorbidity Index			X		
ACE-27 Index			X		
ω-Score			X		
G-8 Score (QoL)			X		
CARG Toxicity Score			X		
CIRS-G Score			X		
Zubrod performance status			X		
Pre-existing peripheral neuropathy grade ≥ 1			X		
History of hearing loss (use of hearing aid or ≥ 25 decibel shift over 2 contiguous frequencies on a pretreatment hearing test as clinically indicated)			X		
Tissue sample for correlative research (baseline) (mandatory)					≤90
Complete Blood Count with Differential (CBCD), including Absolute Neutrophil Count (ANC)				X	
Complete Metabolic Panel (fasting), Magnesium, AST or ALT, Total bilirubin				X	
EKG, troponin					≤72 hours of 1st MEDI14736 (durvalumab) infusion
Pregnancy test, serum or urine (for women of childbearing potential)				X	
Informed consent		X			
PSS-HN (Phase II/III patients only; clinician graded performance/function)					≤28
PRO-CTCAE Assessment (Phase II/III patients only)**					≤28

Quality of Life Assessments***<br>(Phase II/III patients only)<br>• EORTC QLQ-C30 and H&N35<br>• MDADI<br>• EQ-5D-5L					≤28
Blood specimen collection for Banking (for patients who consent to biobanking)					≤14
Blood specimen collection for Peripheral Blood Analysis (for phase II/III patients who consent to blood laboratory study)					≤14
Mandatory pre-hoc central confirmation of p16 status (oropharyngeal and unknown primary cases only)		After Step 1 registration and before Step 2 registration			

*Unless patient refuses or cannot tolerate.
**The specific PRO-CTCAE items for this protocol can be found on the forms section of the CTSU protocol webpage and is titled “NRG-HN004 NCI PRO-CTCAE Item Library”. Baseline PRO-CTCAE will be completed on paper.
***Baseline Quality of Life questionnaires will be completed on paper.

ASSESSMENTS DURING RADIATION THERAPY
(Day -7 [7 days prior to any treatment] to 4 weeks after RT + Systemic Therapy)

**Assessments**	**Within 7 Days Prior to Any Systemic Therapy**	**Weekly (± 2 days) during RT, or as specified**	**End of RT (± 2 days)**	**1 mo. (± 7 days) after end of RT**
Complete Blood Count with Differential (CBCD), including Absolute Neutrophil Count (ANC)	X	X		
Creatinine Clearance	X	X		
Complete Metabolic Panel, Magnesium	X	X		
TSH*<br><br>Note: Thyroid function tests are not needed for patients randomized to the cetuximab arm.	X	●Week 2 of RT<br>●Week 6 of RT		
Physical examination by Radiation Oncologist or Medical Oncologist or ENT Physician or Head & Neck Surgeon; evaluation must include a listing of concomitant medications		X		
Troponin**	X [for MEDI4736 (durvalumab) arm only]			
Adverse event evaluation	X	X	X	
PSS-HN (Phase II/III patients only; clinician graded performance/function)			X	
PRO-CTCAE<br>(Phase II/III patients only)***		●After the first dose of drug and prior to starting RT<br>●Week 2 of RT<br>●Week 6 of RT	X	
Quality of Life<br>(Phase II/III patients only)<br>• EORTC QLQ-C30 and H&N35<br>• EQ5D-5L<br>• MDADI			X	
Blood specimen collection for Banking (for patients who consent to biobanking)		≤ 7 days prior to starting RT		X
Blood specimen collection for Peripheral Blood Analysis (for phase II/III patients who consent to blood laboratory study)		≤ 7 days prior to starting RT (and after starting systemic		X

		therapy)		

*With reflex to free T4; T3 only if clinically indicated afterwards.
**For all patients in safety run-in component, troponin must be tested prior to each dose of MEDI14736 (durvalumab) and any additional cardiac testing should be performed as clinically indicated. During the phase II/III component, EKG and troponin (and other cardiac tests such as CK and echocardiogram) should be performed as clinically indicated.
***The specific PRO-CTCAE items for this protocol can be found on the forms section of the CTSU protocol webpage and is titled "NRG-HN004 NCI PRO-CTCAE Item Library". A 7-day window will be allowed for collection of PRO-CTCAE. This study uses Medidata Patient Cloud ePRO. Remember to register the patient to the Patient Cloud ePRO. For instructions on registering the patients, please refer to Appendix III.

ASSESSMENTS DURING ADJUVANT THERAPY
4-16 weeks after RT+ MEDI4736 (Durvalumab), for patients on investigational therapy only

**Assessments**	**Within 3 Days Prior to Beginning Adjuvant Therapy**	**Q 4 weeks (± 7 days) During Adjuvant Therapy**
Complete Blood Count with Differential (CBCD), including Absolute Neutrophil Count (ANC)	X	X
Creatinine Clearance	X	X
Complete Metabolic Panel, Magnesium	X	X
TSH*, amylase, lipase	X	X
Troponin**		X
Physical examination by Radiation Oncologist or Medical Oncologist or ENT Physician or Head & Neck Surgeon; evaluation must include a listing of concomitant medications		X
Adverse Event evaluation	X	X

*With reflex to free T4; T3 only if clinically indicated afterwards
**For all patients in safety run-in component, troponin must be tested prior to each dose of MEDI14736 (durvalumab) and any additional cardiac testing should be performed as clinically indicated. During the phase II/III component, EKG and troponin (and other cardiac tests such as CK and echocardiogram) should be performed as clinically indicated.

ASSESSMENTS IN FOLLOW UP

Assessments	1 mo. (± 7 days) after last dose of systemic therapy	For cetuximab arm only: 2 and 3 mos. (± 7 days) after last dose of systemic therapy	Q 4 mos. (± 28 days) from end of RT for 1 year, then Q 6 mos. (± 28 days) for 2 years, then annually******
Complete Blood Count with Differential (CBCD), including Absolute Neutrophil Count (ANC)	X		
Creatinine Clearance	X		
TSH, amylase, lipase*	X		
Complete Metabolic Panel, Magnesium	X		
Physical examination by Radiation Oncologist or Medical Oncologist or ENT Physician or Head & Neck Surgeon; evaluation must include a listing of medications	X		X
Performance Status evaluation , including PSS-HN (Phase II/III patients only)			X<br>(4, 8, 12, 18, 24 months only)
Evaluation for presence of a feeding tube	X		X
Adverse event evaluation	X	X†	X
Whole body 18-F-FDG-PET/CT			4 months, then as clinically indicated**
Chest CT (with or without contrast)			As clinically indicated
Diagnostic CT or MRI of neck, with contrast			4 months, then as clinically indicated***
Troponin****	X [for MEDI4736 (durvalumab) arm only]		
Quality of Life Assessments<br>(Phase II/III patients only)<br>• EORTC QLQ-C30 and H&N35<br>• MDADI<br>• EQ5D-5L			X<br>(4, 8, 12, 18, 24 months from end of RT only)
PRO-CTCAE Assessment (Phase II/III patients only)*****			X<br>(4 months from end of RT only)
Blood specimen collection for banking (for patients who consent to biobanking)	X (for durvalumab arm only)		X<br>(4 months from end of RT for cetuximab arm only)
Blood specimen collection for Peripheral Blood Analysis (for phase	X (for durvalumab arm		X<br>(4 months from end

II/III patients who consent to blood laboratory study)	only)		of RT for cetuximab arm only)

*TSH, amylase, lipase for patients in the MEDI4736 (durvalumab) arm. For TSH, with reflex to free T4; T3 only if clinically indicated afterwards.
**Both whole body 18-F-FDG-PET/CT and diagnostic CT or MRI of the neck are strongly encouraged 4 months following completion of RT +/- 2 weeks, but are not mandatory. For patients in the MEDI4736 (durvalumab) arm, this imaging should be done after the last dose of adjuvant MEDI4736 (durvalumab). If a PET/CT is acquired at any other time in follow-up then additional CT or MRI of the neck and chest CT are not required at the same time point.
***CT (with contrast) and/or MRI (with contrast) of the head and neck is highly recommended at the indicated time points, particularly in the presence of persistent symptoms or abnormal/equivocal clinical examination findings.
****For all patients in safety run-in component, troponin must be tested at 1 month after last MEDI14736 (durvalumab) infusion and any additional cardiac testing should be performed as clinically indicated. During the phase II/III component, EKG and troponin (and other cardiac tests such as CK and echocardiogram) should be performed only as clinically indicated
*****The specific PRO-CTCAE items for this protocol can be found on the forms section of the CTSU protocol webpage and is titled "NRG-HN004 NCI PRO-CTCAE Item Library". A 7-day window will be allowed for collection of PRO-CTCAE. This study uses Medidata Patient Cloud ePRO. Remember to register the patient to the Patient Cloud ePRO. For instructions on registering the patients, please refer to Appendix III.
****** Patients enrolled to the safety-lead in portion are followed for a total of 2 years.
† Patients randomized to Cetuximab: Adverse event evaluation is required at Month 2 and 3 after the last dose of systemic therapy. The patient can be seen by the Radiation or Medical Oncologist or the adverse event evaluation can be done over the phone by a physician or designee (i.e. physician assistant, nurse or nurse practitioner).

Note: For patients with local or regional neck failure, post-treatment imaging showing the failure at any time post-treatment must be submitted in DICOM format via TRIAD. The report is to be uploaded into Rave.

<u>Definition of Disease Assessments</u>
Progression of head and neck cancer will be assessed at defined time points by radiological imaging as indicated in the Assessment Tables above. Additionally, any clinical evidence of disease progression as determined by the treating physician outside of these defined time points during treatment and in follow up should trigger confirmation by radiological imaging and biopsy, if clinically feasible and associated with acceptable risk to the patient. The window for confirmation is at least 4 weeks after the scan showing progressive disease.

The protocol will utilize RECIST, v. 1.1 for the purposes of evaluating response and progression.

As described in RECIST, v. 1.1, it is strongly recommended that the imaging studies performed at baseline be performed in follow up on the same equipment with the same techniques as noted above in Recommendations for Imaging. CT neck and chest with contrast of diagnostic quality (slice thickness no greater than 3 mm in the head/neck and 5 mm in the chest) is preferred, with CT head, abdomen/pelvis added if clinically indicated. 18-F-FDG-PET/CT (inclusive of the chest) may be used as radiographic evaluation for overall cancer status.

Progressive disease will be defined as follows:

1. Clinical evidence of disease progression in a radiated field that is confirmed by cytology or histopathology (**see Note**)

   —OR—

2. Progressive disease as defined by RECIST, v. 1.1 criteria (Eisenhauer 2009)
   a) At least a 20% increase in the sum of the diameters of target lesions; the sum also must demonstrate an

absolute increase of at least 5 mm OR

b) unequivocal progression of non-target lesions OR
c) the appearance of one or more new lesions

Note: A positive biopsy at the primary site or involved nodes that occurs < 4 months from the end of RT will be categorized as disease persistence and ≥ 4 months as progression.

Immediate adjuvant neck dissection is not indicated in this population and should not be performed. Pathologic confirmation of persistent disease at the time of a salvage neck dissection (performed, say, for persistent adenopathy on imaging ≥ 4 months post-treatment, or for clinical/radiographic evidence of progression of adenopathy < 4 months post-treatment) would be considered a progression event. An adjuvant neck dissection performed in deviation of the protocol that indicates pathologic persistent disease will not be considered a progression event. Pathologic confirmation of disease is required to consider a neck dissection as a progression event. If a progression occurs at a site of previous gross disease, this is considered a local failure; if progression occurs in the elective neck, this is considered a regional failure.

It is anticipated that measurable, target lesions by RECIST, v. 1.1 for head and neck cancer would include the primary tumor and up to 2 measurable cervical lymph nodes with a short axis of ≥ 15 mm. Non-target lesions would include cervical lymph nodes > 10 mm and < 15 mm. Lymph nodes will be considered a single "organ" for the purpose of analyzing response by RECIST v. 1.1.

Local or regional progression during primary treatment for head and neck cancer is unusual; however, it may occur and would be defined by meeting RECIST, v. 1.1 criteria in comparison to the imaging studies performed at baseline/enrollment. Per RECIST, v. 1.1. progressive disease is defined by at least a 20% increase in the sum of the diameters of target lesions, taking as a reference the smallest sum on study. It is anticipated that the first required imaging time point after completion of radiotherapy (4 months after RT) will be the reference scan for defining progression. However, further decline in measurement of target lesions may occur beyond the 4-month post RT evaluation time point. In that instance, the reference scan containing the smallest sum may occur beyond the 4-month post-RT time point.

RECIST, v. 1.1 recognizes that it can be difficult to determine progressive disease in a previously irradiated or previously treated area (e.g. after neck dissection). In the event of clinical suspicion of progressive disease within an irradiated field in the absence of meeting formal RECIST definition, investigators are strongly encouraged to perform further investigation, including:

a) Repeat CT imaging at a short interval (e.g. six weeks). If short interval imaging results in confirmation of progressive disease by RECIST, v. 1.1 definition, the date of progression will be defined by the first scan.
b) 18-F-FDG-PET/CT: Per RECIST, progressive disease would be indicated on 18-F-FDG-PET/CT if a previously negative 18-F-FDG-PET/CT becomes positive at a new lesion. For this protocol, a previously negative measurable lesion that becomes positive by FDG-PET also would be considered progression. Biopsy confirmation, however, is still strongly recommended.
c) Biopsy

In addition to formal evaluation of progressive disease by RECIST, v. 1.1, investigators will be asked at the time of first progression to report whether progressive disease was local (at the primary tumor), regional (within the head and neck, including cervical lymph nodes) or distant (inclusive of brain and below the clavicles). Sites of distant disease progression should be noted. This will accommodate analysis of patterns of failure.

All images should be reviewed and reported by the local study investigators by RECIST, version 1.1.

Case-report forms for investigator interpretation of progressive disease by RECIST, v. 1.1 are available in Medidata Rave. All imaging studies performed at baseline and 4 months post-RT or in response to clinical concern for progression are required to be submitted for a potential centralized review by RECIST, version 1.1.

5. TREATMENT PLAN/REGIMEN DESCRIPTION

5.1 **Systemic Therapy** (15-JAN-2019)

Protocol treatment must begin within 10 days after Step 2 Registration.

**Arm 1**		Week of IMRT						
	-1	1	2	3	4	5	6	7
IMRT (2 Gy/fx, 5 days/week)		X	X	X	X	X	X	X
Cetuximab<br>400 mg/m^2 loading dose	X							
Cetuximab*<br>250 mg/m^2		X	X	X	X	X	X	X
*Refer to section 5.1.2 for acceptable variance +/- 1 day for weekly cetuximab.								

**Arm 2**		Week of IMRT										
	-2*	1**	2*	3	4	5	6	7	10	14	18	22
IMRT (70 Gy- 2 Gy/fx)		X	X	X	X	X	X	X				
MEDI4736<br>(Durvalumab)<br>1500 mg IV	X		X				X		X	X	X	X
*NOTE: Time between the first and second doses of MEDI4736 (durvalumab) is 3 weeks. All remaining doses are every 4 weeks. Refer to section 5.1.1 for acceptable variance +/- 2 days.<br>**RT will start 2 weeks (+/- 1 day) after the first dose of MEDI4736 (durvalumab).												

5.1.1 MEDI4736 (Durvalumab)

Cycle 1 of MEDI4736 (durvalumab) will be administered as a 1500 mg fixed dose starting 2 weeks prior to RT. A variance of 2 days is acceptable for scheduling conflicts such as vacation or holidays. Please refer to the dosing table in 5.1 for further schedule details. The infusion rate for MEDI4736 (durvalumab) will be approximately 60 minutes.

TABLE 1
Lead-In Dose Levels

Dose Level	MEDI4736 (Durvalumab)	IMRT
Dose level 1	Initial Dose: 1500 mg IV Week -2<br><br>Concurrent: 1500 mg IV Weeks 2, 6 of IMRT<br><br>Maintenance: 1500 mg IV, q4 weeks for 4 doses (Weeks 10, 14, 18, 22)	70 Gy in 7 weeks (conventional fractionation)
Dose level -1; if necessary	Initial Dose: 1500 mg IV Week -2<br><br>Concurrent: 1500 mg IV<br>Week 6 of IMRT<br><br>Maintenance: 1500 mg IV, q4 weeks for 5 doses (Weeks 10, 14, 18, 22, 26)	70 Gy in 7 weeks (conventional fractionation)

Dose level -2;<br>if necessary	Initial Dose: 1500 mg IV Week -2<br><br>Concurrent: None<br><br>Maintenance: 1500 mg IV, q4 weeks for 6 doses<br>(Weeks 10, 14, 18, 22, 26, 30)	70 Gy in 7 weeks<br>(conventional<br>fractionation)

TABLE 2
Lead-In Treatment Plan by Dose Level

Dose Level 1		Week of IMRT										
	-2*	1**	2*	3	4	5	6	7	10	14	18	22
IMRT (2 Gy/fx)		X	X	X	X	X	X	X				
MEDI4736<br>(Durvalumab)<br>1500 mg IV	X		X				X		X	X	X	X
*NOTE: Time between the first and second doses of durvalumab is 3 weeks.<br>**RT will start 2 weeks (+/- 1 day) after the first dose of durvalumab.												

(Dose Level -1;<br>if necessary		Week of IMRT											
	-2*	1	2	3	4	5	6*	7	10	14	18	22	26
IMRT (2 Gy/fx)		X	X	X	X	X	X	X					
MEDI4736<br>(Durvalumab)<br>1500 mg IV	X						X		X	X	X	X	X
*NOTE: Time between the first and second doses of durvalumab is 7 weeks.													

Dose Level -2;<br>if necessary		Week of IMRT												
	-2*	1	2	3	4	5	6	7	10*	14	18	22	26	30
IMRT (2 Gy/Fx)		X	X	X	X	X	X	X						
MEDI4736<br>(Durvalumab)<br>1500 mg IV	X								X	X	X	X	X	X
*NOTE: Time between the first and second doses of durvalumab is 11 weeks.														

[Dose Level -1 and Dose Level -2 were not used for the Lead-In Treatment Plan]

5.1.2 Cetuximab

For the phase II/III regimen in Arm 1, cetuximab will be administered as 400 mg/m^2 loading dose prior to RT then 250 mg/m^2 weekly x 7 cycles concurrent with RT.

Note: Actual body weight should be use for all calculations of BSA.

Cetuximab Loading Dose: Patients on Arm 1 will receive an initial dose of cetuximab, 400 mg/m^2, intravenously (IV) over 120 minutes (maximum infusion rate 10 mg/min). No radiation will be given this day.

The loading dose of cetuximab will precede the first 250 mg/m^2 dose of concurrent cetuximab and the first radiation treatment by at least 5, but no more than 7, days (the day of the loading dose is not included in these 5 days).

Cetuximab Concurrent Dose: Subsequent doses will be given at 250 mg/m^2 intravenously (IV) over 60 minutes (maximum infusion rate 10 mg/min). A variance of 1 day is acceptable for scheduling conflicts such as vacation or holidays.

Note: Patients receive a total of 8 doses of cetuximab over 8 weeks, including the initial loading dose, 7 doses concurrent with radiation therapy. If a dose of cetuximab is omitted, it will not be made up or added to the end of treatment. The omitted dose and the reason for the omission should be recorded in the site's source documentation.

Hypersensitivity Prophylaxis: Infusion reactions may occur during or following cetuximab administration. Most infusion reactions occur with the first infusion of cetuximab, but some patients' first infusion reactions have been reported following subsequent doses (a severe reaction occurred in one patient following the 8th dose). The infusion reaction may occur during the infusion or be delayed until any time after the infusion.

- Loading Dose Prophylaxis: All patients will be premedicated with diphenhydramine hydrochloride, 50 mg, (or an equivalent antihistamine) by IV 30-60 minutes prior to the first dose of cetuximab in an effort to prevent an infusion reaction. At the discretion of the treating physician, dexamethasone, 20 mg, and an H2 blocker also may be administered IV.
- Subsequent Dose Prophylaxis: Premedications are recommended prior to subsequent doses, but at the Investigator's discretion, the dose of diphenhydramine or dexamethasone may be reduced.
- Monitoring Loading Dose: The medical staff should closely observe patients for treatment-related adverse events, especially infusion reactions (see Section 6.2 for management) during the cetuximab infusion and during a post-infusion observation period, per their institutional guidelines. For the initial cetuximab infusion, vital signs (blood pressure, heart rate, respiratory rate, and temperature) should be monitored before, during, and after administration of cetuximab according to institutional guidelines, preferably in an area with resuscitation equipment and other agents (epinephrine, prednisone equivalents, etc.) available, and with a nurse and physician present in close proximity to the treatment area throughout the infusion and observation period. In the event that a patient experiences an infusion reaction, the site should follow institutional guidelines for proper management.
- Monitoring Subsequent Doses: For subsequent infusions, vital signs should be taken pre- and post-infusion; however, it is recommended that the patient be observed for 1 hour post-infusion. For the duration that patients are on study therapy, adverse event monitoring will be done continuously. Patients will be evaluated for adverse events at each visit and are to be instructed to call their physician to report any clinically significant adverse events between visits. Patients should be instructed to report any delayed reactions to the investigator immediately.

5.2 Radiation Therapy (13-JAN-2021)

Protocol treatment must begin within 10 days after Step 2 Registration.

Note: All participating institutions must be credentialed for head and neck IMRT and IGRT prior to registering patients to the study (see Section 8.3 for details).

Intensity Modulated Radiation Therapy (IMRT) and Image-Guided Radiation Therapy (IGRT) are mandatory for this study. Proton therapy is not permitted.

IMRT (70 Gy) will be given in 35 fractions over 7 weeks, 5 fractions per week. Missed treatments due to holidays or logistical reasons can be compensated by delivering additional BID fractions, with a minimum inter-fraction interval of 6 hours, or by treating on Saturday or Sunday.

5.2.1 Treatment Technology

Megavoltage energy photon beam irradiation with a photon beam of ≥ 4 MV is required.

Note:

- VMAT is allowed.
- Matched conventional anterior low neck field is allowed.
- Cyberknife is not allowed.

5.2.2 Immobilization and Simulation (04-OCT-2019)

Immobilization

Immobilization with a thermoplastic mask is mandatory for head and neck IMRT. Patients will be planned in the supine position with their arms at their sides, ensuring the shoulders are situated as far inferior as possible to avoid entry and exit through the shoulders. Additional immobilization devices such as a bite block are permitted. It is strongly encouraged that the participating centers also include the shoulders in the immobilization to further ensure accurate patient set-up on a daily basis.

Simulation Imaging

The treatment planning CT scan is mandatory for defining target volumes and normal organ at risk. The planning CT scan should be done with intravenous contrast (unless contraindicated). CT scan thickness should be a maximum of 0.3 cm, and the CT scan should be acquired with the patient in the same position and using the same immobilization device as for treatment. All tissues receiving irradiation should be included in the CT scan limits. The scanning limits should at least encompass the orbits superiorly, and extend at least 1 cm below the suprasternal notch inferiorly.

5.2.3 Imaging for Structure Definition, Image Registration/Fusion and Follow up

A diagnostic CT or MRI for structure delineation is recommended. These may be fused to the planning CT scans to facilitate target and structure definition. When available
FDG PET/CT images may also be fused to the planning CT data set. All image sets used for structure delineation must be submitted with the RT digital data.

5.2.4 Definition of Dose Prescriptions, Target Volumes, and Margins

Note: All structures must be named for digital RT data submission as listed in the table below. The structures marked as “Required” in the table must be contoured and submitted with the treatment plan. Structures marked as “Required when applicable” must be contoured and submitted when applicable.

Resubmission of data may be required if labeling of structures does not conform to the standard DICOM name listed. Capital letters, spacing and use of underscores must be applied exactly as indicated.

Standard Name	Description	Validation Required/Required When Applicable/Optional
GTV_7000	Primary tumor and involved nodes	**Required**
CTV_7000	GTV + 5-10mm margin, excluding anatomic boundaries to tumor spread	**Required**
PTV_7000	CTV-to-PTV 5mm margin that can be reduced to 3mm in some situations	**Required**
PTV_Eval_7000	PTV minus OARs, subtract 8 mm from the skin when needed	**Required when applicable**
CTV_5600	CTV_7000 + 1cm margin (excluding anatomic boundaries to tumor spread), plus elective nodal regions	**Required**
PTV_5600	CTV-to-PTV 3mm margin	**Required**
CTV_6125	CTV_7000 plus regions at high-risk for subclinical disease	**Required when applicable**
PTV_6125	CTV-to-PTV 3mm margin	**Required when applicable**

General

The primary tumor and involved nodes will be encompassed by PTV_7000 and will receive 2 Gy per fraction x 35 fractions, while subclinical disease sites will be encompassed by PTV_5600 which will

receive 1.6 Gy per fraction x 35 fractions. Treatment of both volumes will occur at the same time using a simultaneous integrated boost (SIB) technique delivered at 5 fractions per week over 7 weeks. For regions considered to be at high risk for microscopic disease, PTV_6125 may be treated to a dose of 61.25 Gy at 1.75 Gy per fraction x 35 fractions. The treatment plan used for each patient will be based on an analysis of the volumetric dose, including dose-volume histogram (DVH) analyses of the PTVs and critical normal structures. Inverse planning with computerized optimization should be used. The density corrected dose distributions shall be calculated, and the dose prescription is to be based on a dose distribution corrected for heterogeneities.

Gross Tumor Volume (GTV) represents the region judged to contain gross primary tumor or grossly involved node(s) based on clinical and endoscopic examinations, CT scan, and, when applicable, other imaging techniques. Grossly positive lymph nodes are defined as any lymph nodes > 1 cm or nodes with a necrotic center.

Clinical Target Volume (CTV) is defined as the GTV plus areas considered at risk for containing microscopic disease delineated by the treating physician. CTV_7000 represents GTV plus a margin of generally 5-10 mm, and CTV_5600 represents CTV_7000 with a margin of 10 mm, plus nodal regions to receive elective irradiation. When the tumor is infiltrative (endophytic) or when the border is ill defined, it might be desirable to deliver an intermediate dose to a volume (CTV_6125) that is slightly larger than CTV_7000. The CTV margins can be narrower when GTV is in the proximity of the spinal cord or critical normal tissues. CTV should be cropped to exclude anatomical barriers to tumor spread such as air cavities, uninvolved bone, and external body contours.

Guidelines for CT based delineation of lymph node levels for node negative patients can be found on the NRG Oncology website: https://www.nrgoncology.org/ciro-head-and-neck

For patients with positive neck nodes, consult Gregoire et al. (2014) for the delineation of the nodal CTV.

Planning Target Volume (PTV) represents an additional margin around CTV to compensate for the variability of treatment set-up and internal organ motion. A minimum margin of 3mm around the CTV is required in all directions to define each respective PTV, except for situations in which the CTV is adjacent to spinal cord or other critical normal tissues. In such situations, the margin can be reduced judiciously at the discretion of the treating physician.

PTV_Eval_7000: PTV volume minus impinging high priority OARs for dosimetric evaluation. For some patients, a PTV will overlap critical organs, such as the brainstem, spinal cord, optic structures, and brachial plexus. When a PTV overlaps a critical OAR (spinal cord and/or brainstem) and its associated PRV, the PTV should be modified to exclude the OAR so as to limit the dose delivered to the OAR. 8 mm can be subtracted from the skin when needed.

When expansion of a CTV results in a PTV that extends beyond the patient's body surface, the PTV should be constrained to at least 3 mm from within the external contour, while still including the CTV. The use of tissue equivalent bolus material is indicated in situations where the disease is at or just under the skin surface. The PTV should align with the skin surface when bolus is used. Other volumes, such as tuning or avoidance or optimization structures, can be employed to drive the IMRT treatment planning process. Such volumes should be considered to be treatment-planning tools that are not reported or sent forward for review.

Note. ***PTV _7000*** and ***PTV_Eval_7000*** (Required When Applicable) must be sent for review.

5.2.5 Definition of Critical Structures and Margins

Note: All structures must be named for digital RT data submission as listed in the table below. The structures marked as "Required" in the table must be contoured and submitted with the treatment plan. Structures marked as "Required when applicable" must be contoured and submitted when applicable. Resubmission of data may be required if labeling of structures does not conform to the standard DICOM name listed. Capital letters, spacing and use of underscores must be applied exactly as indicated.

Standard Name	Description	Validation (Required/Required when applicable/Optional)
SpinalCord	Spinal Cord	**Required**
SpinalCord_PRV05	Planning Risk Volume of 5mm margin around Spinal Cord	**Required**
BrainStem	Brain Stem	**Required**
BrainStem_PRV05	Planning Risk Volume of 5mm margin around Brain Stem	**Required**
Lobe_Temporal_L	Left Temporal Lobe	**Required**
Lobe_Temporal_R	Right Temporal Lobe	**Required**
OpticNrv_L	Left Optic Nerve	**Required**
OpticNrv_R	Right Optic Nerve	**Required**
OpticChiasm	Optic Chiasm	**Required**
Parotid_L	Left Parotid	**Required**
Parotid_R	Right Parotid	**Required**
Cavity_Oral	Oral Cavity	**Required**
Lips	Lips	**Required**
Bone_Mandible	Mandible	**Required**
Joint_TM_L	Left Temporomandibular Joint	**Required**
Joint_TM_R	Right Temporomandibular Joint	**Required**
Pharynx	Uninvolved posterior pharyngeal wall plus adjacent constrictor muscles	**Required**
Esophagus_S	Upper Cervical Esophagus	**Required**
Larynx_SG	Supra Glottic Larynx	**Required**
Glnd_Submand_L	Left Submandibular Salivary Gland	**Required**
Glnd_Submand_R	Right Submandibular Salivary Gland	**Required**
External	Patient contour encompassing all patient anatomy with a single contour on each slice	**Required**
E-PTV	All tissue excluding the PTVs	**Required**

Detailed Specifications

Spinal Cord: The spinal cord begins at the cranial-cervical junction (i.e. the top of the C1 vertebral body). The last slice of the contouring of the spinal cord should be at T3-T4. The spinal cord shall be defined based on the treatment planning CT scan. In addition, however, a Planning Risk Volume (PRV) spinal cord shall be defined. The PRV cord = spinal cord + 5 mm in each dimension. This is irrespective of the use of IGRT for margin reduction.
Brainstem: The inferior most portion of the brainstem is at the cranial-cervical junction where it meets the spinal cord. For the purposes of this study, the superior most portion of the brainstem is approximately at the level of the top of the posterior clinoid process. The brainstem shall be defined based on the treatment planning CT scan. In addition, however, a Planning Risk Volume (PRV) brainstem shall be defined. The PRV brainstem = brainstem + 5 mm in each dimension. This is irrespective of the use IGRT for margin reduction.

Lips and Oral Cavity: These should be contoured as 2 separate structures as the goal is to keep the lip dose much lower than the oral cavity dose. The definition of lips is self-explanatory. The oral cavity will be defined as a composite structure consisting of the anterior one half to two thirds of the oral tongue/floor of mouth, buccal mucosa, and palate.

Parotid Glands: Parotid glands are defined based on the treatment planning CT scan.

Pharynx: This is defined as the “uninvolved” posterior pharyngeal wall plus adjacent constrictor muscles. This extends from the superior constrictor region (the inferior pterygoid plates level) to the cricopharyngeal inlet (posterior cricoid cartilage level).

Cervical Esophagus (Esophagus_S): This is defined as a tubular structure that starts at the bottom of pharynx and extends to the thoracic inlet.

Larynx (GSL) (Larynx_SG): The GSL begins just inferior to the hyoid bone and extends to the cricoid cartilage inferiorly and extends from the anterior commissure to include the arytenoids. This includes the infrahyoid but not suprahyoid epiglottis.

Mandible (Bone_Mandible): The mandible includes the entire bony structure from TMJ through the symphysis. It is recognized that for oral cavity cancers, this may overlap with CTVs and PTVs.

Unspecified Tissue outside of the Targets: This will be defined as all tissue located between the skull base and thoracic inlet excluding all PTVs and defined normal structures within the external contour of the patient.

5.2.6 Dose Prescription

Note: The information provided in this section can be used for adjusting the dose constraints for treatment planning purposes. This table together with the planning priority table should be used during dose optimization. It is important to remember that ideal plans might not be achievable in all cases. Thus, the Compliance Criteria table could be different than the information given here. Cases will be scored using the Compliance Criteria table.

**Target Standard Name**	**Dose (Gy)**	**Fraction Size** (Gy)	**# of fractions**	**Frequency**	**Dose specification technique**
PTV_7000	70	2.0	35	Daily	Covering 95% of PTV

5.2.7 Compliance Criteria

The compliance criteria listed here will be used to score each case. Given the limitations inherent in the treatment planning process, the numbers given in this section can be different than the prescription table. The Per Protocol and Variation Acceptable categories are both considered to be acceptable. The Per Protocol cases can be viewed as ideal plans, and the Variation Acceptable category can include more challenging plans that do not fall at or near the ideal results. A final category, called Deviation Unacceptable, results when cases do not meet the requirements for either Per Protocol or Variation Acceptable. Plans falling in this category are considered to be suboptimal and additional treatment planning optimization is recommended.

VxGy [cc], VxGy [%], Vx%[cc], Vx%[%]: Volume [cc or %] receiving Dose [Gy, or %]
Dxcc[Gy], Dxcc[%], Dx%[Gy], Dx%[%]: Dose [Gy or %] to Volume [cc or % of total volume]
Minimum dose is defined to D99%[Gy] or D99%[%]
Maximum dose is defined as D0.03cc[Gy] or D0.03cc[%]
Mean[Gy] or Mean[%]: Mean dose in Gy or %

Normalization of Dose: The plan is normalized such that 95% of the PTV_7000 volume receives prescription dose of 70 Gy.

Note: Deviation Unacceptable occurs when dose limits for Variation Acceptable are not met

Target Volume and OAR Constraints

Name of Structure	Dosimetric Parameter	Per Protocol	Variation Acceptable
PTV_7000	D95%[Gy]	>=70	>=69
	V95%[%]	>99	>=90
	D99%[Gy]	>=66.5	>=63

	D0.03cc[Gy]	<=77	<= 82
PTV_Eval_7000	D99%[Gy]	>=66.5	> 63
PTV_6125	D95%[Gy]	>=61.25	>=58
PTV_5600	D95%[Gy]	>=56	>=50.4
SpinalCord_PRV05	D0.03cc[Gy]	<= 50	<= 52
BrainStem_PRV05	D0.03cc[Gy]	<= 54	<=56
OpticNrv_L or OpticNrv_R	D0.03cc[Gy]	<= 54	<=56
OpticChiasm	D0.03cc[Gy]	<=54	<=56
Bone_Mandible	D0.03cc[Gy]	<=70	<=75
Joint_TM_L or Joint_TM_R	D0.03cc[Gy]	<=70	<=75
Lobe_Temporal_L or Lobe_Temporal_R	D0.03cc[Gy]	<=70	<=72

Per Protocol range is excluded from Variation Acceptable range.

It is recognized that portions of PTVs close to the skin or critical PRVs (spinal cord and brainstem) may receive significantly less than the prescription doses. This is acceptable in these regions as long as cold spots within these PTVs do not exist within the GTV. In cases of PTVs close to skin, tissue equivalent bolus must be utilized to ensure adequate dose.

It is also recognized that PTVs abutting or enclosing higher dose PTVs will have regions of maximum dose that may exceed their prescribed dose in order to achieve acceptable minimal doses to the higher dose PTVs which are considered a higher priority target.

Recommended dose acceptance criteria for other normal tissue but not to be used for plan score

**Name of Structure**	**Recommended dose acceptance criteria**
Parotid_L or Parotid_R	Mean[Gy] <26 (for at least one parotid)
Lips	Mean[Gy] <20
Larynx_SG	Mean[Gy] <20
Pharynx	Mean[Gy] <45
Glnd_Submand_L or Glnd_Submand_R (contralateral)	Mean[Gy] <39
Cavity_Oral (non-involved oral cavity)	Mean[Gy] <30
	D0.03cc[Gy] <60
Esophagus_S	Mean[Gy] <30
	D0.03cc[Gy] <60
E-PTV	D1cc[Gy] <74

Delivery Compliance Criteria

	Per Protocol	Variation Acceptable	Deviation Unacceptable
Overall Treatment time	< 50 days	50-54 days (without a medically appropriate indication for delay	> 54 days (without a medically appropriate indication for delay)
Interruptions	0-2 days	3-4 days	> 4 days

5.2.8 Treatment Planning Priorities and Instructions

Prioritization of IMRT Planning

1. SpinalCord
2. BrainStem
3. PTV_7000
4. PTV_6125 (if applicable)
5. PTV_5600
6. a. Pharynx
   b. Parotid gland contralateral to primary tumor site
7. a. Larynx_SG
   b. Esophagus_S
8. a. Lips
   b. Cavity_Oral
9. a. Parotid gland ipsilateral to primary tumor site
   b. Bone_Mandible
10. Unspecified tissue outside the targets

Acceptable choices of algorithm are listed at:
http://rpc.mdanderson.org/rpc/Services/Anthropomorphic_%20Phantoms/TPS%20-%20algorithm%20list%20updated.pdf. Any algorithm used for this study must be credentialed by IROC Houston.

All doses should be reported in terms of dose-to-water and not in terms of dose-to-medium.

An algorithm that is not included in this list must be credentialed by IROC Houston. For Convolution/Superposition type algorithms, dose should be reported as computed inherently by the given algorithm. For Monte Carlo or Grid Based Boltzmann Solver algorithms, conversion of Dm (dose-to-medium) to Dw (dose-to-water) should be avoided. Dm, computed inherently by these algorithms, should be reported. These principles hold for Pencil Beam type algorithms and for homogeneous dose calculations when allowed for a clinical trial.

Primary dataset for dose calculation

In the case in which contrast is present during the treatment planning CT, whether the density of the contrast should be overridden to a representative background electron density should be tested to demonstrate such density overridden is negligible to dose calculation. In addition, image artifacts such as streaks near metal, dental implants, fillings, clips or other high density objects should be overridden with appropriate HUs. In some instances plastic surgical shuts may appear radio-opaque and require appropriate HU override.

Dose matrix resolution

Dose grid size should be ≤ 3 mm in all directions.

Adaptive Planning

Patients may experience weight change or the target size may change during treatment. Due to the sensitive nature of the dose distribution to anatomical change, adaptive planning may be required to maintain protocol dose coverage to the PTV and OARs. All adaptive plans and final plan sum dose statistics should be submitted at the end of treatment.

5.2.9 Patient-Specific QA

For IMRT/VMAT plans, patient specific QA is highly recommended. Any patient-specific QA performed should follow your institutional and AAPM guidelines. The recommended patient specific QA criteria is for 90% of the comparison points to pass a ±3%/3mm Gamma Index analysis.

5.2.10 Treatment Localization/IGRT

Image-guided radiation therapy (IGRT) is radiation therapy using imaging to facilitate accuracy and precision throughout its entire process. In this section we use the terminology IGRT to focus on image-

guidance at the time of radiation delivery to ensure its adherence to the planned treatment, with computer assisted process, i.e. image handling together with calculation of shifts and rotations (if available) must be determined with computer assistance.

IGRT Instructions

Bony anatomy IGRT credentialing is required (see Section 8.3). Daily image guidance of IMRT may be achieved using any one or more of the following techniques:

- Orthogonal kilovoltage (KV) images;
- Linear-accelerator mounted kV and MV cone beam CT images;
- Linear-accelerator mounted MV CT images.

The institution's procedure for registering daily treatment imaging datasets with a reference dataset should comply with the following recommendations:

- Region-of-Interest (ROI) or "clip box" for fusion should be set to encompass the high dose PTV and adjacent spinal cord; if the supraclavicular region is a part of the target volume the ROI should extend to the C6 level;
- If the fusion software allows the user to create an irregular ROI, treatment room objects seen on in-room x-rays should be excluded from the registration;
- Both manual (e.g. drag-and-drop system based on bony anatomy matching) and automatic (e.g. based on mutual information) types of registration can be used; the result of the fusion must be visually checked for the alignment of the bony anatomy, such as vertebral bodies and applicable soft tissue structures (e.g. optic nerves and/or optic chiasm).
- Following the registration, the translational and (if the appropriate technology is available) rotational corrections should be applied to the treatment couch. If all the variances are < 2.5 mm (this typically corresponds to one half of the usual PRV margin), the treatment can proceed without correction (however, the physician/team may elect to perform adjustments even for a variance < 2.5 mm). If one or more corrections are 2.5 to 5 mm, adjustment is necessary prior to treatment; however, reimaging is not mandatory. If one or more of the corrections are > 5 mm, the imaging must be repeated in addition to performing table/positioning adjustments. However, the use of numerous repeat IGRT studies should be avoided;
- Imaging dose to the patient may become significant if repeated studies are performed for patients with severe set up problems (e.g., requiring frequent corrections of more than 5 mm). It is recommended that patients demonstrating severe set up problems during the first 7 calendar days of treatment be re-simulated or moved to a treatment with larger margins.

5.3 General Concomitant Medication and Supportive Care Guidelines

5.3.1 Permitted Supportive/Ancillary Care and Concomitant Medications

All supportive therapy for optimal medical care will be given during the study period at the discretion of the attending physician(s) within the parameters of the protocol and documented on each site's source documents as concomitant medication. Please see Section 6.1/Supportive Care Guidelines for details.

5.3.2 Prohibited Therapies

- The use of amifostine or palifermin as a radioprotectant is not allowed. The use of granulocyte colony stimulating factor or erythropoietin is not allowed. Transfusion is to be performed at the discretion of the treating physician.
- Any exceptions must be approved by the Principal Investigator, Dr. Mell. An e-mail must be sent to Dr. Mell for the approval and NRG Oncology Headquarters Data Managers must be copied on that e-mail.
- Immunosuppressive medications, including, but not limited to, systemic corticosteroids at doses exceeding 10 mg/day or prednisone or equivalent, methotrexate, azathioprine and tumor necrosis factor-a blockers. These should not be given concomitantly or used for premedication prior to the MEDI4736 (durvalumab) infusions. Exceptions include: the use of immunosuppressive medications for the management of IP-related AEs, use in patients with contrast allergies. A temporary period of steroids will be allowed if clinically indicated and considered to be essential for the management of non-immunotherapy related events experienced by the patients (e.g. chronic obstructive pulmonary

disease, radiation, nausea, etc.)

5.3.3 Participation in Other Trials

Patients may not participate in other clinical trials that are intended to treat the diagnosed head and neck cancer or intended to reduce toxicity of therapy.

5.3.4 Blood Donation and Vaccination

- Patients should not donate blood while participating in this study, or for at least 90 days after receipt of the final dose of MEDI4736 (durvalumab).
- Live attenuated vaccines should not be administered whilst receiving MEDI4736 (durvalumab) and for 30 days after the last dose of MEDI4736 (durvalumab).

5.4 Duration of Therapy (15-JAN-2019)

Patients assigned to MEDI4736 (durvalumab) plus RT will receive 7 doses of MEDI4736 (durvalumab). Patients assigned to cetuximab plus RT will receive 8 doses of cetuximab.

In the absence of treatment delays due to adverse event(s), treatment will continue as specified in the above treatment modality sections or until one of the following criteria applies:

- Disease progression;
- Intercurrent illness that prevents further administration of treatment;
- Unacceptable adverse event(s);
- Patient decides to withdraw consent for participation in the study;
- General or specific changes in the patient's condition render the patient unacceptable for further treatment in the judgment of the investigator;
- New data related to the experimental agent which would suggest that continuing treatment on protocol would impose unwarranted potential risks beyond what was known at the time of treatment initiation.

6. TREATMENT MODIFICATIONS/MANAGEMENT

NOTE: PRO-CTCAE data should not be used for determining dose delays or dose modifications or any other protocol directed action.

6.1 Dose Modifications and Toxicity Management Guidelines for Immune-Mediated, Infusion-Related, and Non-Immune Mediated Reactions for MEDI4736 (Durvalumab) (15-JAN-2019)

Dose modifications, per the tables below, are based upon CTCAE v5.0 adverse event assessment.

6.1.1 Dose Modifications

There are no dose reductions for MEDI4736 (durvalumab). If the patient meets retreatment criteria, the full dose of 1500 mg will be administered. If the patient does not meet retreatment criteria before the next scheduled dose, if/when the toxicity resolves to the point where treatment is possible, the doses of MEDI4736 (durvalumab) are applied off schedule q 4 weeks.

Adverse events (both non-serious and serious) associated with MEDI4736 (durvalumab) exposure may represent an immunologic etiology. These adverse events may occur shortly after the first dose or several months after the last dose of treatment. MEDI4736 (durvalumab) must be withheld for drug-related toxicities and severe or life-threatening AEs as per the table below.

Note: If in the judgment of the investigator an event is considered ***clinically unrelated or unlikely to be related to*** MEDI4736 (durvalumab), and an alternate clinical explanation is likely, probable, or definite (for example, attributable to prerenal azotemia in the setting of nausea, and responding to hydration), then corticosteroids may be omitted and MEDI4736 (durvalumab) may be administered. However, even the unrelated event should be treated appropriately and resolved prior to continuing treatment.

Dose Modifications	Toxicity Management
Drug administration modifications of study drug/study regimen will be made to manage potential immune-related AEs based on severity of treatment-emergent toxicities graded per NCI CTCAE v5.0.<br>In addition to the criteria for permanent discontinuation of study drug/study regimen based on CTC grade/severity (table below), permanently discontinue study drug/study regimen for the following conditions:<br>• Inability to reduce corticosteroid to a dose of ≤10 mg of prednisone per day (or equivalent) **within 12 weeks** after last dose of study drug/study regimen<br>• Recurrence of a previously experienced Grade 3 treatment-related AE following resumption of dosing<br>**Grade 1** No dose modification<br>**Grade 2** Hold study drug/study regimen dose until Grade 2 resolution to Grade ≤1.<br>If toxicity worsens, then treat as Grade 3 or Grade 4.<br>Study drug/study regimen can be resumed once event stabilizes to Grade ≤1 after completion of steroid taper.<br>Patients with endocrinopathies who may require prolonged or continued steroid replacement can be retreated with study drug/study regimen on the following conditions:<br>1. The event stabilizes and is controlled.<br>2. The patient is clinically stable as per Investigator or treating physician's clinical judgement.<br>3. Doses of prednisone are at ≤10 mg/day or equivalent.<br>**Grade 3** Depending on the individual toxicity, study drug/study regimen may be permanently discontinued. Please refer to guidelines below.<br>**Grade 4** Permanently discontinue study drug/study regimen.<br>Note: There are some exceptions to permanent discontinuation of study drug for Grade 4 events (i.e., hyperthyroidism, hypothyroidism, Type 1 diabetes mellitus).	It is recommended that management of immune-mediated adverse events (imAEs) follows the guidelines presented in this table:<br>– Patients should be thoroughly evaluated to rule out any alternative etiology (e.g., disease progression, concomitant medications, and infections).<br>– In the absence of a clear alternative etiology, all events should be considered potentially immune related.<br>– Symptomatic and topical therapy should be considered for low-grade (Grade 1 or 2, unless otherwise specified) events.<br>– For persistent (>3 to 5 days) low-grade (Grade 2) or severe (Grade ≥3) events, promptly start prednisone 1 to 2 mg/kg/day PO or IV equivalent.<br>– If symptoms recur or worsen during corticosteroid tapering (28 days of taper), increase the corticosteroid dose (prednisone dose [e.g., up to 2 to 4 mg/kg/day PO or IV equivalent]) until stabilization or improvement of symptoms, then resume corticosteroid tapering at a slower rate (>28 days of taper).<br>– More potent immunosuppressives such as TNF inhibitors (e.g., infliximab) (also refer to the individual sections of the imAE for specific type of immunosuppressive) should be considered for events not responding to systemic steroids.<br>– Discontinuation of study drug/study regimen is not mandated for Grade 3/Grade 4 inflammatory reactions attributed to local tumor response (e.g., inflammatory reaction at sites of metastatic disease and lymph nodes). Continuation of study drug/study regimen in this situation should be based upon a benefit-risk analysis for that patient.

AE Adverse event; CTC Common Toxicity Criteria; CTCAE Common Terminology Criteria for Adverse Events; imAE Immune-mediated adverse event; IV intravenous; NCI National Cancer Institute; PO By mouth.

Specific Immune-Mediated Reactions

Adverse Events	Severity Grade of the Event (NCI CTCAE version v5.0)	Dose Modifications	Toxicity Management
**Pneumonitis/Interstitial Lung Disease (ILD)**	**Any Grade**	**General Guidance**	**For Any Grade:**<br>– Monitor patients for signs and symptoms of pneumonitis or ILD (new onset or worsening shortness of breath or cough). Patients should be evaluated with imaging and pulmonary function tests, including other diagnostic procedures as described below.<br>– Initial work-up may include clinical evaluation, monitoring of oxygenation via pulse oximetry (resting and exertion), laboratory work-up, and high- resolution CT scan.
	**Grade 1**	No dose modifications required. However, consider holding study drug/study regimen dose as clinically appropriate and during diagnostic work-up for other etiologies.	**For Grade 1 (radiographic changes only):**<br>– Monitor and closely follow up in 2 to 4 days for clinical symptoms, pulse oximetry (resting and exertion), and laboratory work-up and then as clinically indicated.<br>– Consider Pulmonary and Infectious disease consult.
	**Grade 2**	Hold study drug/study regimen dose until Grade 2 resolution to Grade ≤1.<br>• If toxicity worsens, then treat as Grade 3 or Grade 4.<br>• If toxicity improves to Grade ≤1, then the decision to reinitiate study drug/study regimen will be based upon treating physician's clinical judgment and after completion of steroid taper.	**For Grade 2 (mild to moderate new symptoms):**<br>– Monitor symptoms daily and consider hospitalization.<br>– Promptly start systemic steroids (e.g., prednisone 1 to 2 mg/kg/day PO or IV equivalent).<br>– Reimage as clinically indicated.<br>– If no improvement within 3 to 5 days, additional workup should be considered and prompt treatment with IV methylprednisolone 2 to 4 mg/kg/day started<br>– If still no improvement within 3 to 5 days despite IV methylprednisolone at 2 to 4 mg/kg/day, promptly start immunosuppressive therapy such as TNF inhibitors (e.g., infliximab at 5 mg/kg every 2 weeks). Caution: It is

			important to rule out sepsis and refer to infliximab label for general guidance before using infliximab.<br>− Once the patient is improving, gradually taper steroids over ≥28 days and consider prophylactic antibiotics, antifungals, or anti-PCP treatment (refer to current NCCN guidelines for treatment of cancer-related infections (Category 2B recommendation)[a]<br>− Consider Pulmonary and Infectious disease consult.<br>− Consider, as necessary, discussing with study physician.
	**Grade 3 or 4**	Permanently discontinue study drug/study regimen.	**For Grade 3 or 4 (severe or new symptoms, new/worsening hypoxia, life-threatening):**<br>− Promptly initiate empiric IV methylprednisolone 1 to 4 mg/kg/day or equivalent.<br>− Obtain Pulmonary and Infectious disease consult; consider, as necessary, discussing with study physician.<br>− Hospitalize the patient.<br>− Supportive care (e.g., oxygen).<br>− If no improvement within 3 to 5 days, additional workup should be considered and prompt treatment with additional immunosuppressive therapy such as TNF inhibitors (e.g., infliximab at 5 mg/kg every 2 weeks' dose) started. Caution: rule out sepsis and refer to infliximab label for general guidance before using infliximab.<br>− Once the patient is improving, gradually taper steroids over ≥28 days and consider prophylactic antibiotics, antifungals, and, in particular, anti-PCP treatment (refer to current NCCN guidelines for treatment of cancer-related infections (Category 2B recommendation).[a]
**Diarrhea/Colitis**	**Any Grade**	**General Guidance**	**For Any Grade:**

		– Monitor for symptoms that may be related to diarrhea/enterocolitis (abdominal pain, cramping, or changes in bowel habits such as increased frequency over baseline or blood in stool) or related to bowel perforation (such as sepsis, peritoneal signs, and ileus).<br>– Patients should be thoroughly evaluated to rule out any alternative etiology (e.g., disease progression, other medications, or infections), including testing for clostridium difficile toxin, etc.<br>– Steroids should be considered in the absence of clear alternative etiology, even for low-grade events, in order to prevent potential progression to higher grade event.<br>– Use analgesics carefully; they can mask symptoms of perforation and peritonitis.
**Grade 1**	No dose modifications.	**For Grade 1:**<br>– Monitor closely for worsening symptoms.<br>– Consider symptomatic treatment, including hydration, electrolyte replacement, dietary changes (e.g., American Dietetic Association colitis diet), and loperamide. Use probiotics as per treating physician's clinical judgment.
**Grade 2**	Hold study drug/study regimen until resolution to Grade ≤1<br>• If toxicity worsens, then treat as Grade 3 or Grade 4.<br>• If toxicity improves to Grade ≤1, then study drug/study regimen can be resumed after completion of steroid taper.	**For Grade 2:**<br>– Consider symptomatic treatment, including hydration, electrolyte replacement, dietary changes (e.g., American Dietetic Association colitis diet), and loperamide and/or budesonide.<br>– Promptly start prednisone 1 to 2 mg/kg/day PO or IV equivalent.<br>– If event is not responsive within 3 to 5 days or worsens despite prednisone at 1 to 2 mg/kg/day PO or IV equivalent, GI consult should be obtained for consideration of further workup, such as imaging and/or colonoscopy,

		to confirm colitis and rule out perforation, and prompt treatment with IV methylprednisolone 2 to 4 mg/kg/day started.<br>− If still no improvement within 3 to 5 days despite 2 to 4 mg/kg IV methylprednisolone, promptly start immunosuppressives such as infliximab at 5 mg/kg once every 2 weeks[a]. **Caution:** it is important to rule out bowel perforation and refer to infliximab label for general guidance before using infliximab.<br>− Consider, as necessary, discussing with study physician if no resolution to Grade ≤1 in 3 to 4 days.<br>− Once the patient is improving, gradually taper steroids over ≥28 days and consider prophylactic antibiotics, antifungals, and anti-PCP treatment (refer to current NCCN guidelines for treatment of cancer-related infections [Category 2B recommendation]).[a]
**Grade 3 or 4**	**Grade 3**<br>Permanently discontinue study drug/study regimen for Grade 3 if toxicity does not improve to Grade ≤1 within 14 days; study drug/study regimen can be resumed after completion of steroid taper.<br><br>**Grade 4**<br>Permanently discontinue study drug/study regimen.	**For Grade 3 or 4:**<br>− Promptly initiate empiric IV methylprednisolone 2 to 4 mg/kg/day or equivalent.<br>− Monitor stool frequency and volume and maintain hydration.<br>− Urgent GI consult and imaging and/or colonoscopy as appropriate.<br>− If still no improvement within 3 to 5 days of IV methylprednisolone 2 to 4 mg/kg/day or equivalent, promptly start further immunosuppressives (e.g., infliximab at 5 mg/kg once every 2 weeks). **Caution**: Ensure GI consult to rule out bowel perforation and refer to infliximab label for general guidance before using infliximab.<br>− Once the patient is improving, gradually taper steroids over

			≥28 days and consider prophylactic antibiotics, antifungals, and anti-PCP treatment (refer to current NCCN guidelines for treatment of cancer-related infections [Category 2B recommendation]).[a]
**Hepatitis (elevated LFTs)**<br>Infliximab should not be used for management of immune-related hepatitis.	**Any Grade**	**General Guidance**	**For Any Grade:**<br>– Monitor and evaluate liver function test: AST, ALT, ALP, and TB.<br>– Evaluate for alternative etiologies (e.g., viral hepatitis, disease progression, concomitant medications).
	**Grade 1 (Based on ULN regardless of baseline LFT)**	No dose modifications.<br>• If it worsens, then treat as Grade 2 event.	**For Grade 1:**<br>– Continue LFT monitoring per protocol.
	**Grade 2 (Based on ULN regardless of baseline LFT)**	Hold study drug/study regimen dose until Grade 2 resolution to Grade ≤1.<br>• If toxicity worsens, then treat as Grade 3 or Grade 4.<br>• If toxicity improves to Grade ≤1 or baseline, resume study drug/study regimen after completion of steroid taper.	**For Grade 2:**<br>– Regular and frequent checking of LFTs (e.g., every 1 to 2 days) until elevations of these are improving or resolved.<br>– If no resolution to Grade ≤1 in 1 to 2 days, consider, as necessary, discussing with study physician.<br>– If event is persistent (>3 to 5 days) or worsens, promptly start prednisone 1 to 2 mg/kg/day PO or IV equivalent.<br>– If still no improvement within 3 to 5 days despite 1 to 2 mg/kg/day of prednisone PO or IV equivalent, consider additional work up and start prompt treatment with IV methylprednisolone 2 to 4 mg/kg/day.<br>– If still no improvement within 3 to 5 days despite 2 to 4 mg/kg/day of IV methylprednisolone, promptly start immunosuppressives (mycophenolate mofetil).[a] Discuss with study physician if mycophenolate mofetil is not available. **Infliximab should NOT be used.**<br>– Once the patient is improving, gradually taper steroids over

			≥28 days and consider prophylactic antibiotics, antifungals, and anti-PCP treatment (refer to current NCCN guidelines for treatment of cancer-related infections [Category 2B recommendation]).[a]
	**Grade 3 or 4 (Based on ULN regardless of baseline LFT)**	**For Grade 3:**<br>For elevations in transaminases ≤8 × ULN, or elevations in bilirubin ≤5 × ULN:<br>• Hold study drug/study regimen dose until resolution to Grade ≤1 or baseline<br>• Resume study drug/study regimen if elevations downgrade to Grade ≤1 or baseline within 14 days and after completion of steroid taper.<br>• Permanently discontinue study drug/study regimen if the elevations do not downgrade to Grade ≤1 or baseline within 14 days<br>For elevations in transaminases >8 × ULN or elevations in bilirubin >5 × ULN, discontinue study drug/study regimen.<br>Permanently discontinue study drug/study regimen for any case meeting Hy's law criteria (AST and/or ALT >3 × ULN + bilirubin >2 × ULN without initial findings of cholestasis (i.e., elevated alkaline P04) and in the absence of any alternative cause.[b]	**For Grade 3 or 4:**<br>– Promptly initiate empiric IV methylprednisolone at 1 to 4 mg/kg/day or equivalent.<br>– If still no improvement within 3 to 5 days despite 1 to 4 mg/kg/day methylprednisolone IV or equivalent, promptly start treatment with immunosuppressive therapy (i.e., mycophenolate mofetil). Discuss with study physician if mycophenolate is not available. **Infliximab should NOT be used.**<br>– Perform hepatology consult, abdominal workup, and imaging as appropriate.<br>– Once the patient is improving, gradually taper steroids over ≥28 days and consider prophylactic antibiotics, antifungals, and anti-PCP treatment (refer to current NCCN guidelines for treatment of cancer-related infections [Category 2B recommendation]).[a]

		**For Grade 4:**<br>Permanently discontinue study drug/study regimen.	
**Nephritis or renal dysfunction**<br>(elevated serum creatinine)	**Any Grade**	**General Guidance**	**For Any Grade:**<br>– Consult with nephrologist.<br>– Monitor for signs and symptoms that may be related to changes in renal function (e.g., routine urinalysis, elevated serum BUN and creatinine, decreased creatinine clearance, electrolyte imbalance, decrease in urine output, or proteinuria).<br>– Patients should be thoroughly evaluated to rule out any alternative etiology (e.g., disease progression or infections).<br>– Steroids should be considered in the absence of clear alternative etiology even for low-grade events (Grade 2), in order to prevent potential progression to higher grade event.
	**Grade 1**	No dose modifications.	**For Grade 1:**<br>– Monitor serum creatinine weekly and any accompanying symptoms.<br>• If creatinine returns to baseline, resume its regular monitoring per study protocol.<br>• If creatinine worsens, depending on the severity, treat as Grade 2, 3, or 4.<br>– Consider symptomatic treatment, including hydration, electrolyte replacement, and diuretics.
	**Grade 2**	Hold study drug/study regimen until resolution to Grade ≤1 or baseline.<br>• If toxicity worsens, then treat as Grade 3 or 4.<br>• If toxicity improves to Grade ≤1 or baseline, then resume study drug/study regimen	**For Grade 2:**<br>– Consider symptomatic treatment, including hydration, electrolyte replacement, and diuretics.<br>– Carefully monitor serum creatinine every 2 to 3 days and as clinically warranted.<br>– Consult nephrologist and consider renal biopsy if clinically indicated.

		after completion of steroid taper.	– If event is persistent (>3 to 5 days) or worsens, promptly start prednisone 1 to 2 mg/kg/day PO or IV equivalent.<br>– If event is not responsive within 3 to 5 days or worsens despite prednisone at 1 to 2 mg/kg/day PO or IV equivalent, additional workup should be considered and prompt treatment with IV methylprednisolone at 2 to 4 mg/kg/day started.<br>– Once the patient is improving, gradually taper steroids over ≥28 days and consider prophylactic antibiotics, antifungals, and anti-PCP treatment (refer to current NCCN guidelines for treatment of cancer-related infections [Category 2B recommendation]).[a]<br>– When event returns to baseline, resume study drug/study regimen and routine serum creatinine monitoring per study protocol.
	**Grade 3 or 4**	Permanently discontinue study drug/study regimen.	**For Grade 3 or 4:**<br>– Carefully monitor serum creatinine on daily basis.<br>– Consult nephrologist and consider renal biopsy if clinically indicated.<br>– Promptly start prednisone 1 to 2 mg/kg/day PO or IV equivalent.<br>– If event is not responsive within 3 to 5 days or worsens despite prednisone at 1 to 2 mg/kg/day PO or IV equivalent, additional workup should be considered and prompt treatment with IV methylprednisolone 2 to 4 mg/kg/day started.<br>– Once the patient is improving, gradually taper steroids over ≥28 days and consider prophylactic antibiotics, antifungals, and anti-PCP treatment (refer to current NCCN guidelines for treatment of cancer-related infections

			[Category 2B recommendation]).[a]
**Rash** (excluding bullous skin formations)	**Any Grade** (refer to NCI CTCAE v5.0 for definition of severity/grade depending on type of skin rash)	**General Guidance**	**For Any Grade:**<br>– Monitor for signs and symptoms of dermatitis (rash and pruritus).<br>– IF THERE IS ANY BULLOUS FORMATION, THE STUDY PHYSICIAN SHOULD BE CONTACTED **AND STUDY DRUG DISCONTINUED.**
	**Grade 1**	No dose modifications.	**For Grade 1:**<br>– Consider symptomatic treatment, including oral antipruritics (e.g., diphenhydramine or hydroxyzine) and topical therapy (e.g., urea cream).
	**Grade 2**	For persistent (>1 to 2 weeks) Grade 2 events, hold scheduled study drug/study regimen until resolution to Grade ≤1 or baseline.<br>• If toxicity worsens, then treat as Grade 3.<br>• If toxicity improves to Grade ≤1 or baseline, then resume drug/study regimen after completion of steroid taper.	**For Grade 2:**<br>– Obtain dermatology consult.<br>– Consider symptomatic treatment, including oral antipruritics (e.g., diphenhydramine or hydroxyzine) and topical therapy (e.g., urea cream).<br>– Consider moderate-strength topical steroid.<br>– If no improvement of rash/skin lesions occurs within 3 to 5 days or is worsening despite symptomatic treatment and/or use of moderate strength topical steroid, consider, as necessary, discussing with study physician and promptly start systemic steroids such as prednisone 1 to 2 mg/kg/day PO or IV equivalent.<br>– Consider skin biopsy if the event is persistent for >1 to 2 weeks or recurs.
	**Grade 3 or 4**	**For Grade 3:**<br>Hold study drug/study regimen until resolution to Grade ≤1 or baseline.<br>If temporarily holding the study drug/study regimen does not provide improvement of the Grade 3 skin rash to Grade ≤1 or baseline	**For Grade 3 or 4:**<br>– Consult dermatology.<br>– Promptly initiate empiric IV methylprednisolone 1 to 4 mg/kg/day or equivalent.<br>– Consider hospitalization.<br>– Monitor extent of rash [Rule of Nines].

		within 30 days, then permanently discontinue study drug/study regimen.<br><br>**For Grade 4:**<br>Permanently discontinue study drug/study regimen.	− Consider skin biopsy (preferably more than 1) as clinically feasible.<br>− Once the patient is improving, gradually taper steroids over ≥28 days and consider prophylactic antibiotics, antifungals, and anti-PCP treatment (refer to current NCCN guidelines for treatment of cancer-related infections [Category 2B recommendation]).[a]<br>− consider, as necessary, discussing with study physician.
**Endocrinopathy** (e.g., hyperthyroidism, hypothyroidism, Type 1 diabetes mellitus, hypophysitis, hypopituitarism, and adrenal insufficiency; exocrine event of amylase/lipase increased also included in this section)	**Any Grade** (depending on the type of endocrinopathy, refer to NCI CTCAE v5.0 for defining the CTC grade/severity)	General Guidance	**For Any Grade:**<br>− Consider consulting an endocrinologist for endocrine events.<br>− Consider, as necessary, discussing with study physician.<br>− Monitor patients for signs and symptoms of endocrinopathies. Non-specific symptoms include headache, fatigue, behavior changes, changed mental status, vertigo, abdominal pain, unusual bowel habits, polydipsia, polyuria, hypotension, and weakness.<br>− Patients should be thoroughly evaluated to rule out any alternative etiology (e.g., disease progression including brain metastases, or infections).<br>− Depending on the suspected endocrinopathy, monitor and evaluate thyroid function tests: TSH, free T3 and free T4 and other relevant endocrine and related labs (e.g., blood glucose and ketone levels, HgA1c).<br>− For modest asymptomatic elevations in serum amylase and lipase, corticosteroid treatment is not indicated as long as there are no other signs or symptoms of pancreatic inflammation.<br>− If a patient experiences an AE that is thought to be possibly of autoimmune nature (e.g.,

			thyroiditis, pancreatitis, hypophysitis, or diabetes insipidus), the investigator should send a blood sample for appropriate autoimmune antibody testing.
	**Grade 1**	No dose modifications.	**For Grade 1 (including those with asymptomatic TSH elevation):**<br>− Monitor patient with appropriate endocrine function tests.<br>− For suspected hypophysitis/hypopituitarism, consider consultation of an endocrinologist to guide assessment of early-morning ACTH, cortisol, TSH and free T4; also consider gonadotropins, sex hormones, and prolactin levels, as well as cosyntropin stimulation test (though it may not be useful in diagnosing early secondary adrenal insufficiency).<br>− If TSH < 0.5 × LLN, or TSH >2 × ULN or consistently out of range in 2 subsequent measurements, include free T4 at subsequent cycles as clinically indicated and consider consultation of an endocrinologist .
	**Grade 2**	For Grade 2 endocrinopathy and Type 1 diabetes mellitus other than hypothyroidism, hold study drug/study regimen dose until patient is clinically stable.<br>• If toxicity worsens, then treat as Grade 3 or Grade 4.<br>Study drug/study regimen can be resumed once event stabilizes and after completion of steroid taper.<br>Patients with endocrinopathies who may require prolonged or continued steroid	**For Grade 2 (including those with symptomatic endocrinopathy):**<br>− Consult endocrinologist to guide evaluation of endocrine function and, as indicated by suspected endocrinopathy and as clinically indicated, consider pituitary scan.<br>− For all patients with abnormal endocrine work up, except those with isolated hypothyroidism or Type 1 DM, and as guided by an endocrinologist, consider short-term corticosteroids (e.g., 1 to 2 mg/kg/day methylprednisolone or IV equivalent) and prompt initiation of treatment with relevant hormone replacement (e.g., hydrocortisone, sex hormones).

	replacement (e.g., adrenal insufficiency) can be retreated with study drug/study regimen on the following conditions:<br>1. The event stabilizes and is controlled.<br>2. The patient is clinically stable as per investigator or treating physician's clinical judgement.<br>3. Doses of prednisone are ≤10 mg/day or equivalent.	– Isolated hypothyroidism may be treated with replacement therapy, without study drug/study regimen interruption, and without corticosteroids.<br>– Isolated Type 1 diabetes mellitus (DM) may be treated with appropriate diabetic therapy, without study drug/study regimen interruption, and without corticosteroids.<br>– Once patients on steroids are improving, gradually taper immunosuppressive steroids (as appropriate and with guidance of endocrinologist) over ≥28 days and consider prophylactic antibiotics, antifungals, and anti-PCP treatment (refer to current NCCN guidelines for treatment of cancer-related infections [Category 2B recommendation]).[a]<br>– For patients with normal endocrine workup (laboratory assessment or MRI scans), repeat laboratory assessments/MRI as clinically indicated.
**Grade 3 or 4**	For Grade 3 or 4 endocrinopathy other than hypothyroidism and Type 1 diabetes mellitus, hold study drug/study regimen dose until endocrinopathy symptom(s) are controlled.<br>Study drug/study regimen can be resumed once event stabilizes and after completion of steroid taper.<br>Patients with endocrinopathies who may require prolonged or continued steroid replacement (e.g., adrenal insufficiency) can be retreated with study drug/study	**For Grade 3 or 4:**<br>– Consult endocrinologist to guide evaluation of endocrine function and, as indicated by suspected endocrinopathy and as clinically indicated, consider pituitary scan. Hospitalization recommended.<br>– For all patients with abnormal endocrine work up, except those with isolated hypothyroidism or Type I DM, and as guided by an endocrinologist, promptly initiate empiric IV methylprednisolone 1 to 2 mg/kg/day or equivalent, as well as relevant hormone replacement (e.g., hydrocortisone, sex hormones).<br>– For adrenal crisis, severe dehydration, hypotension, or

		regimen on the following conditions:<br>1. The event stabilizes and is controlled.<br>2. The patient is clinically stable as per investigator or treating physician's clinical judgement.<br>3. Doses of prednisone are ≤10 mg/day or equivalent.	shock, immediately initiate IV corticosteroids with mineralocorticoid activity.<br>− Isolated hypothyroidism may be treated with replacement therapy, without study drug/study regimen interruption, and without corticosteroids.<br>− Isolated Type 1 diabetes mellitus may be treated with appropriate diabetic therapy, without study drug/study regimen interruption, and without corticosteroids.<br>− Once patients on steroids are improving, gradually taper immunosuppressive steroids (as appropriate and with guidance of endocrinologist) over ≥28 days and consider prophylactic antibiotics, antifungals, and anti-PCP treatment (refer to current NCCN guidelines for treatment of cancer-related infections [Category 2B recommendation]).[a]
**Neurotoxicity**<br>(to include but not be limited to limbic encephalitis and autonomic neuropathy, excluding Myasthenia Gravis and Guillain-Barre)	**Any Grade**<br>(depending on the type of neurotoxicity, refer to NCI CTCAE v5.0 for defining the CTC grade/severity)	**General Guidance**	**For Any Grade:**<br>− Patients should be evaluated to rule out any alternative etiology (e.g., disease progression, infections, metabolic syndromes, or medications).<br>− Monitor patient for general symptoms (headache, nausea, vertigo, behavior change, or weakness).<br>− Consider appropriate diagnostic testing (e.g., electromyogram and nerve conduction investigations).<br>− Perform symptomatic treatment with neurological consult as appropriate.
	**Grade 1**	No dose modifications.	**For Grade 1:**<br>− See "Any Grade" recommendations above.
	**Grade 2**	For acute motor neuropathies or neurotoxicity, hold study drug/study	**For Grade 2:**<br>− Consider, as necessary, discussing with the study physician.<br>− Obtain neurology consult.

		regimen dose until resolution to Grade ≤1.<br>For sensory neuropathy/neuropathic pain, consider holding study drug/study regimen dose until resolution to Grade ≤1.<br>If toxicity worsens, then treat as Grade 3 or 4.<br>Study drug/study regimen can be resumed once event improves to Grade ≤1 and after completion of steroid taper.	− Sensory neuropathy/neuropathic pain may be managed by appropriate medications (e.g., gabapentin or duloxetine).<br>− Promptly start systemic steroids prednisone 1 to 2 mg/kg/day PO or IV equivalent.<br>− If no improvement within 3 to 5 days despite 1 to 2 mg/kg/day prednisone PO or IV equivalent, consider additional workup and promptly treat with additional immunosuppressive therapy (e.g., IV IG).
	**Grade 3 or 4**	**For Grade 3:**<br>Hold study drug/study regimen dose until resolution to Grade ≤1.<br>Permanently discontinue study drug/study regimen if Grade 3 imAE does not resolve to Grade ≤1 within 30 days.<br><br>**For Grade 4:**<br>Permanently discontinue study drug/study regimen.	**For Grade 3 or 4:**<br>− Consider, as necessary, discussing with study physician.<br>− Obtain neurology consult.<br>− Consider hospitalization.<br>− Promptly initiate empiric IV methylprednisolone 1 to 2 mg/kg/day or equivalent.<br>− If no improvement within 3 to 5 days despite IV corticosteroids, consider additional workup and promptly treat with additional immunosuppressants (e.g., IV IG).<br>− Once stable, gradually taper steroids over ≥28 days.
**Peripheral neuromotor syndromes**<br>(such as Guillain-Barre and myasthenia gravis)	**Any Grade**	**General Guidance**	**For Any Grade:**<br>− Permanently discontinue MEDI4736 (durvalumab) following the diagnosis of Guillain Barre or myasthenia gravis for any grade<br>− The prompt diagnosis of immune-mediated peripheral neuromotor syndromes is important, since certain patients may unpredictably experience acute decompensations that can result in substantial morbidity or in the worst case, death. Special care should be taken for certain sentinel symptoms that may predict a more severe outcome, such as prominent dysphagia, rapidly progressive weakness, and signs of

			respiratory insufficiency or autonomic instability.<br>− Patients should be evaluated to rule out any alternative etiology (e.g., disease progression, infections, metabolic syndromes or medications). It should be noted that the diagnosis of immune-mediated peripheral neuromotor syndromes can be particularly challenging in patients with underlying cancer, due to the multiple potential confounding effects of cancer (and its treatments) throughout the neuraxis. Given the importance of prompt and accurate diagnosis, it is essential to have a low threshold to obtain a neurological consult.<br>− Neurophysiologic diagnostic testing (e.g., electromyogram and nerve conduction investigations, and "repetitive stimulation" if myasthenia is suspected) are routinely indicated upon suspicion of such conditions and may be best facilitated by means of a neurology consultation.<br>− It is important to consider that the use of steroids as the primary treatment of Guillain-Barre is not typically considered effective. Patients requiring treatment should be started with IV IG and followed by plasmapheresis if not responsive to IV IG.
	**Grade 1**	No dose modifications. Permanently discontinue MEDI4736 (durvalumab) following the diagnosis of any grade of Guillain Barre or myasthenia gravis	**For Grade 1:**<br>− Consider, as necessary, discussing with the study physician.<br>− Care should be taken to monitor patients for sentinel symptoms of a potential decompensation as described above.<br>− Obtain a neurology consult.
	**Grade 2**	Hold study drug/study regimen dose until resolution to Grade ≤1. Permanently discontinue study	**For Grade 2:**<br>− Consider, as necessary, discussing with the study physician.

	drug/study regimen if it does not resolve to Grade ≤1 within 30 days or if there are signs of respiratory insufficiency or autonomic instability. Permanently discontinue MEDI4736 (durvalumab) following the diagnosis of any grade of Guillain Barre or myasthenia gravis	− Care should be taken to monitor patients for sentinel symptoms of a potential decompensation as described above.<br>− Obtain a neurology consult<br>− Sensory neuropathy/neuropathic pain may be managed by appropriate medications (e.g., gabapentin or duloxetine).<br>*MYASTHENIA GRAVIS:*<br>o Steroids may be successfully used to treat myasthenia gravis. It is important to consider that steroid therapy (especially with high doses) may result in transient worsening of myasthenia and should typically be administered in a monitored setting under supervision of a consulting neurologist.<br>o Patients unable to tolerate steroids may be candidates for treatment with plasmapheresis or IV IG. Such decisions are best made in consultation with a neurologist, taking into account the unique needs of each patient.<br>o If myasthenia gravis-like neurotoxicity is present, consider starting AChE inhibitor therapy in addition to steroids. Such therapy, if successful, can also serve to reinforce the diagnosis.<br>*GUILLAIN-BARRE:*<br>o It is important to consider here that the use of steroids as the primary treatment of Guillain-Barre is not

		typically considered effective.<br>○ Patients requiring treatment should be started with IV IG and followed by plasmapheresis if not responsive to IV IG.
**Grade 3 or 4**	**For Grade 3:**<br>Hold study drug/study regimen dose until resolution to Grade ≤1.<br>Permanently discontinue study drug/study regimen if Grade 3 imAE does not resolve to Grade ≤1 within 30 days or if there are signs of respiratory insufficiency or autonomic instability.<br>Permanently discontinue MEDI4736 (durvalumab) following the diagnosis of any grade of Guillain Barre or myasthenia gravis<br><br>**For Grade 4:**<br>Permanently discontinue study drug/study regimen.	**For Grade 3 or 4 (severe or life-threatening events):**<br>– Consider, as necessary, discussing with study physician.<br>– Recommend hospitalization.<br>– Monitor symptoms and obtain neurological consult.<br>*MYASTHENIA GRAVIS:*<br>○ Steroids may be successfully used to treat myasthenia gravis. They should typically be administered in a monitored setting under supervision of a consulting neurologist.<br>○ Patients unable to tolerate steroids may be candidates for treatment with plasmapheresis or IV IG.<br>○ If myasthenia gravis-like neurotoxicity present, consider starting AChE inhibitor therapy in addition to steroids. Such therapy, if successful, can also serve to reinforce the diagnosis.<br><br>*GUILLAIN-BARRE:*<br>○ It is important to consider here that the use of steroids as the primary treatment of Guillain-Barre is not typically considered effective.<br>○ Patients requiring treatment should be started with IV IG and followed by

			plasmapheresis if not responsive to IV IG.
**Cardiac toxicities (including arrhythmia, conduction disorder heart failure, LV dysfunction, Myocarditis)**	**Any Grade**	**General Guidance**<br>Discontinue drug permanently upon diagnosis of myocarditis, regardless of grade.	**For Any Grade:**<br>− The prompt diagnosis of immune-mediated myocarditis is important, particularly in patients with baseline cardiopulmonary disease and reduced cardiac function.<br>− Consider, as necessary, discussing with the study physician.<br>− Monitor patients for signs and symptoms of myocarditis (new onset or worsening chest pain, arrhythmia, shortness of breath, peripheral edema). As some symptoms can overlap with lung toxicities, simultaneously evaluate for and rule out pulmonary toxicity as well as other causes (e.g., pulmonary embolism, congestive heart failure, malignant pericardial effusion). A Cardiology consultation should be obtained early, with prompt assessment of whether and when to complete a cardiac biopsy, including any other diagnostic procedures.<br>− Initial work-up should include clinical evaluation, BNP, cardiac enzymes, ECG, echocardiogram (ECHO), monitoring of oxygenation via pulse oximetry (resting and exertion), and additional laboratory work-up as indicated. Spiral CT or cardiac MRI can complement ECHO to assess wall motion abnormalities when needed.<br>− Patients should be thoroughly evaluated to rule out any alternative etiology (e.g., disease progression, other medications, or infections)
	**Grade 1**	- No dose modifications required unless clinical suspicion for myocarditis is high, in which case suspected,	**For Grade 1 (no definitive findings):**<br>- Monitor and closely follow up in 2 to 4 days for clinical symptoms, BNP, cardiac enzymes, ECG, ECHO, pulse oximetry (resting and exertion),

		hold durvalumab during work-up.<br>- If myocarditis is excluded, resume after complete resolution to Grade 0.<br>- If myocarditis is diagnosed, permanently discontinue durvalumab.	and laboratory work-up as clinically indicated.<br>- Consider using steroids if clinical suspicion is high.
	**Grade 2, 3 or 4**	- If Grade 2 -- Hold study drug/study regimen. If toxicity rapidly improves to Grade 0 AND myocarditis is excluded, then the decision to reinitiate study drug/study regimen will be based upon treating physician's clinical judgment and after completion of steroid taper.<br>- If toxicity does not rapidly improve, permanently. discontinue study drug/study regimen.<br>- If myocarditis is diagnosed, permanently discontinue durvalumab.<br>- If Grade 3-4, permanently discontinue study drug/study regimen.	**For Grade 2-4:**<br>− Monitor symptoms daily, hospitalize.<br>− Promptly start IV methylprednisolone 2 to 4 mg/kg/day or equivalent after Cardiology consultation has determined whether and when to complete diagnostic procedures including a cardiac biopsy.<br>− Supportive care (e.g., oxygen).<br>− If no improvement within 3 to 5 days despite IV methylprednisolone at 2 to 4 mg/kg/day, promptly start immunosuppressive therapy such as TNF inhibitors (e.g., infliximab at 5 mg/kg every 2 weeks). Caution: It is important to rule out sepsis and refer to infliximab label for general guidance before using infliximab.<br>− Once the patient is improving, gradually taper steroids over ≥28 days and consider prophylactic antibiotics, antifungals, or anti-PJP treatment (refer to current NCCN guidelines for treatment of cancer-related infections [Category 2B recommendation]).[a]
**Myositis/Polymyositis ("Poly/myositis")**	**Any Grade**	**General Guidance**	**For Any Grade:**<br>− Monitor patients for signs and symptoms of poly/myositis. Typically, muscle weakness/pain occurs in proximal muscles including upper arms, thighs, shoulders, hips, neck and back, but rarely affects the extremities including hands and fingers; also difficulty breathing and/or

	trouble swallowing can occur and progress rapidly. Increased general feelings of tiredness and fatigue may occur, and there can be new-onset falling, difficulty getting up from a fall, and trouble climbing stairs, standing up from a seated position, and/or reaching up.<br>– If poly/myositis is suspected, a Neurology consultation should be obtained early, with prompt guidance on diagnostic procedures. Myocarditis may co-occur with poly/myositis; refer to guidance under Myocarditis. Given breathing complications, refer to guidance under Pneumonitis/ILD. Given possibility of an existent (but previously unknown) autoimmune disorder, consider Rheumatology consultation.<br>– Consider, as necessary, discussing with the study physician.<br>– Initial work-up should include clinical evaluation, creatine kinase, aldolase, LDH, BUN/creatinine, erythrocyte sedimentation rate or C-reactive protein level, urine myoglobin, and additional laboratory work-up as indicated, including a number of possible rheumatological/antibody tests (i.e., consider whether a rheumatologist consultation is indicated and could guide need for rheumatoid factor, antinuclear antibody, anti-smooth muscle, antisynthetase [such as anti-Jo-1], and/or signal-recognition particle antibodies). Confirmatory testing may include electromyography, nerve conduction studies, MRI of the muscles, and/or a muscle biopsy. Consider Barium swallow for evaluation of dysphagia or dysphonia.<br>Patients should be thoroughly evaluated

		to rule out any alternative etiology (e.g., disease progression, other medications, or infections).
**Grade 1**	- No dose modifications.	**For Grade 1:**<br>− Monitor and closely follow up in 2 to 4 days for clinical symptoms and initiate evaluation as clinically indicated.<br>− Consider Neurology consult.<br>− Consider, as necessary, discussing with the study physician.
**Grade 2**	Hold study drug/study regimen dose until resolution to Grade ≤1.<br>- Permanently discontinue study drug/study regimen if it does not resolve to Grade ≤1 within 30 days or if there are signs of respiratory insufficiency.	**For Grade 2:**<br>− Monitor symptoms daily and consider hospitalization.<br>− Obtain Neurology consult, and initiate evaluation.<br>− Consider, as necessary, discussing with the study physician.<br>− If clinical course is rapidly progressive (particularly if difficulty breathing and/or trouble swallowing), promptly start IV methylprednisolone 2 to 4 mg/kg/day systemic steroids along with receiving input from Neurology consultant<br>− If clinical course is *not* rapidly progressive, start systemic steroids (e.g., prednisone 1 to 2 mg/kg/day PO or IV equivalent); if no improvement within 3 to 5 days, continue additional work up and start treatment with IV methylprednisolone 2 to 4 mg/kg/day<br>− If after start of IV methylprednisolone at 2 to 4 mg/kg/day there is no improvement within 3 to 5 days, consider start of immunosuppressive therapy such as TNF inhibitors (e.g., infliximab at 5 mg/kg every 2 weeks). Caution: It is important to rule out sepsis and refer to infliximab label for general guidance before using infliximab.<br>− Once the patient is improving, gradually taper steroids over ≥28 days and consider

		prophylactic antibiotics, antifungals, or anti-PJP treatment (refer to current NCCN guidelines for treatment of cancer-related infections [Category 2B recommendation]).[a]
**Grade 3 or 4**	**For Grade 3:**<br>Hold study drug/study regimen dose until resolution to Grade ≤1.<br>Permanently discontinue study drug/study regimen if Grade 3 imAE does not resolve to Grade ≤1 within 30 days or if there are signs of respiratory insufficiency.<br><br>**For Grade 4:**<br>- Permanently discontinue study drug/study regimen.	**For Grade 3 or 4 (severe or life-threatening events):**<br>− Monitor symptoms closely; recommend hospitalization.<br>− Obtain Neurology consult, and complete full evaluation.<br>− Consider, as necessary, discussing with the study physician.<br>− Promptly start IV methylprednisolone 2 to 4 mg/kg/day systemic steroids <u>along with receiving input</u> from Neurology consultant.<br>− If after start of IV methylprednisolone at 2 to 4 mg/kg/day there is no improvement within 3 to 5 days, consider start of immunosuppressive therapy such as TNF inhibitors (e.g., infliximab at 5 mg/kg every 2 weeks). Caution: It is important to rule out sepsis and refer to infliximab label for general guidance before using infliximab.<br>− Consider whether patient may require IV IG, plasmapheresis.<br>− Once the patient is improving, gradually taper steroids over ≥28 days and consider prophylactic antibiotics, antifungals, or anti-PJP treatment (refer to current NCCN guidelines for treatment of cancer-related infections [Category 2B recommendation]).[a]

[a]ASCO Educational Book 2015 “Managing Immune Checkpoint Blocking Antibody Side Effects” by Michael Postow MD.

[b]FDA Liver Guidance Document 2009 Guidance for Industry: Drug Induced Liver Injury – Premarketing Clinical Evaluation.

AChE Acetylcholine esterase; ADL Activities of daily living; AE Adverse event; ALP Alkaline phosphatase test; ALT Alanine aminotransferase; AST Aspartate aminotransferase; BUN Blood urea nitrogen; CT Computed tomography; CTCAE Common Terminology Criteria for Adverse Events; ILD Interstitial lung disease; imAE immune-mediated adverse event; IG Immunoglobulin; IV Intravenous; GI Gastrointestinal; LFT Liver function

tests; LLN Lower limit of normal; MRI Magnetic resonance imaging; NCI National Cancer Institute; NCCN National Comprehensive Cancer Network; PCP ; PO By mouth; T3 Triiodothyronine; T4 Thyroxine; TB Total bilirubin; TNF Tumor necrosis factor; TSH Thyroid-stimulating hormone; ULN Upper limit of normal.

Infusion-Related Reactions

Severity Grade of the Event (NCI CTCAE version 5.0)	Dose Modifications	Toxicity Management
**Any Grade**	General Guidance	**For Any Grade:**<br>– Manage per institutional standard at the discretion of investigator.<br>– Monitor patients for signs and symptoms of infusion-related reactions (e.g., fever and/or shaking chills, flushing and/or itching, alterations in heart rate and blood pressure, dyspnea or chest discomfort, or skin rashes) and anaphylaxis (e.g., generalized urticaria, angioedema, wheezing, hypotension, or tachycardia).
**Grade 1 or 2**	**For Grade 1:**<br>The infusion rate of study drug/study regimen may be decreased by 50% or temporarily interrupted until resolution of the event.<br><br>**For Grade 2:**<br>The infusion rate of study drug/study regimen may be decreased 50% or temporarily interrupted until resolution of the event.<br>Subsequent infusions may be given at 50% of the initial infusion rate.	**For Grade 1 or 2:**<br>– Acetaminophen and/or antihistamines may be administered per institutional standard at the discretion of the investigator.<br>– Consider premedication per institutional standard prior to subsequent doses.<br>– Steroids should not be used for routine premedication of Grade ≤2 infusion reactions.
**Grade 3 or 4**	**For Grade 3 or 4:**<br>Permanently discontinue study drug/study regimen.	**For Grade 3 or 4:**<br>– Manage severe infusion-related reactions per institutional standards (e.g., IM epinephrine, followed by IV diphenhydramine and ranitidine, and IV glucocorticoid).

CTCAE Common Terminology Criteria for Adverse Events; IM intramuscular; IV intravenous; NCI National Cancer Institute.

Non–Immune-Mediated Reactions

Severity Grade of the Event (NCI CTCAE version 5.0)	Dose Modifications	Toxicity Management
**Any Grade**	Note: Dose modifications are not required for AEs not deemed to be related to study treatment (i.e., events due to underlying disease) or for laboratory abnormalities not deemed to be clinically significant.	Treat accordingly, as per institutional standard.
**Grade 1**	No dose modifications.	Treat accordingly, as per institutional standard.
**Grade 2**	Hold study drug/study regimen until resolution to ≤Grade 1 or baseline.	Treat accordingly, as per institutional standard.
**Grade 3**	Hold study drug/study regimen until resolution to ≤Grade 1 or baseline.<br>For AEs that downgrade to ≤Grade 2 within 7 days or resolve to ≤Grade 1 or baseline within 14 days, resume study drug/study regimen administration. Otherwise, discontinue study drug/study regimen.	Treat accordingly, as per institutional standard.
**Grade 4**	Discontinue study drug/study regimen (Note: For Grade 4 labs, decision to discontinue should be based on accompanying clinical signs/symptoms, the Investigator's clinical judgment, and consultation with the Sponsor.).	Treat accordingly, as per institutional standard.

AE Adverse event; CTCAE Common Terminology Criteria for Adverse Events; NCI National Cancer Institute.

6.2 Dose Modification for Cetuximab (15-JAN-2019)

6.2.1 Cetuximab Dose Levels

	**Starting Dose**	**Dose Level –1**	**Dose Level –2**
Cetuximab loading dose	400 mg/m^2	Not applicable	Not applicable
Cetuximab	250 mg/m^2 (weekly)	200 mg/m^2 (weekly)	150 mg/m^2 (weekly)

6.2.2 Cetuximab Dose Modifications for Hematologic Adverse Events

Cetuximab will not be dose reduced or held for hematologic adverse events, such as neutropenia, neutropenic fever, or thrombocytopenia.

6.2.3 Cetuximab Dose Modifications for Non-Hematologic Adverse Events

**Toxicity Grade** (**CTCAE, v5.0**)	**Cetuximab Dose** [a]
**Renal-Calculated**<br>**Creatinine Clearance (Cockroft-Gault Formula, see Section 3.2.3 for formula)**	Maintain dose level
≥ 50 mL/min	Maintain dose level
< 50 mL/min	Maintain dose level
**Nausea/Vomiting**<br>≤ Grade 2 with maximal medical management	Maintain dose level

≥ Grade 3 with maximal medical management	Hold drug until ≤ grade 2, then resume at same dose level
**Other Non-hematologic Adverse Events** [b, c]	
Grade 3- 4, if possibly related to cetuximab, or likely to be exacerbated by continuation of cetuximab, e.g. diarrhea, except for weight loss or mucositis	Hold drug until < grade 3, then resume at 1 dose level reduction
Any grade 1-2	Maintain dose level

[a]Dose levels are relative to the previous dose. Dose reductions of cetuximab below the –2 dose level will not be allowed. If a dose reduction below the -2 dose is mandated by the toxicity grade, cetuximab will be permanently discontinued. In any case of cetuximab treatment delay, there will be no re-loading infusion, and all subsequent treatment will be at the assigned dose level.
[b]With the exception of infusion reaction;
[c]For depressed K or Mg, administer replacement therapy. Chemotherapy should continue at the discretion of the treating physician (see table below for management of hypomagnesemia).

Hypomagnesemia
Electrolyte repletion, principally magnesium, was necessary in some patients treated with cetuximab and in severe cases, intravenous replacement was required. The time to resolution of electrolyte abnormalities is not well known, hence monitoring during and after cetuximab treatment is recommended.

**CTCAE, v5.0 Grade**	**Serum Magnesium**		**Guidelines for management**	**Action**
	**mg/dL**	**mmol/L**		
1	< LLN - 1.2	< LLN - 0.5	Consider replacement with IV magnesium sulfate 2-5 g in normal saline or D5W. Infusion schedule based on institutional guidelines.	Maintain dose and schedule
2	< 1.2 - 0.9	< 0.5 - 0.4	As above for grade 1 and consider prophylactic weekly infusion of magnesium and/or oral magnesium supplementation (e.g. magnesium oxide) if grade 2 of higher hypomagnesemia persists.	Maintain dose and schedule
3	< 0.9 - 0.7	< 0.4 - 0.3	As above for grades 1 and 2	Hold cetuximab until recovery to ≤ grade 2, then resume at same dose level
4	< 0.7	< 0.3	As above for grades 1 and 2	Hold cetuximab until recovery to ≤ grade 2, then reduce by 1 dose level

6.2.4 Cetuximab Infusion Related Reaction Management

**CTCAE, v5.0 Adverse Event Grade**	**Treatment Guidelines** [a]
Grade 1:<br>Mild transient reaction; infusion interruption not indicated; intervention not indicated	For mild infusion reactions manifesting only as delayed drug fever, consider administering prophylactic antihistamine medications for subsequent doses. Maintain the cetuximab dose. Acetaminophen or

	a non-steroidal anti-inflammatory drug (NSAID) may be administered prior to subsequent cetuximab infusions, if not otherwise contraindicated in subjects.
Grade 2:<br>Therapy or infusion interruption indicated but responds promptly to symptomatic treatment (e.g., antihistamines, NSAIDS, narcotics, IV fluids); prophylactic medications indicated for ≤ 24 hrs	For moderate infusion reactions, slow the infusion rate for cetuximab by 50% when the drug is restarted and consider administering antihistamine medications and/or steroidal medications. Maintain the cetuximab dose. Acetaminophen or a non-steroidal anti-inflammatory drug (NSAID) may be administered prior to subsequent cetuximab infusions, if not otherwise contraindicated in subjects.
Grade 3:<br>Prolonged (e.g., not rapidly responsive to symptomatic medication and/or brief interruption of infusion); recurrence of symptoms following initial improvement; hospitalization indicated for clinical sequelae	**NO FURTHER CETUXIMAB .** Severe infusion reactions **require immediate interruption of cetuximab infusion and permanent discontinuation from further treatment with cetuximab.** Appropriate medical therapy including epinephrine, corticosteroids, diphenhydramine bronchodilators, and oxygen should be available for use in the treatment of such reactions. Subjects should be carefully observed until the complete resolution of all signs<br>and symptoms.
Grade 4:<br>Life-threatening consequences; urgent intervention indicated	**NO FURTHER CETUXIMAB.** Life threatening infusion reactions **require immediate interruption of cetuximab infusion and permanent discontinuation from further treatment with cetuximab.** Appropriate medical therapy including epinephrine, corticosteroids, diphenhydramine, bronchodilators, and oxygen should be available for use in the treatment of such reactions. Subjects should be carefully observed until the complete resolution of all signs and symptoms.

[a]Study Therapy Retreatment Following Infusion Reactions: Once a cetuximab infusion rate has been decreased due to an infusion reaction, it will remain decreased for all subsequent infusions. If the subject has a second infusion reaction > grade 2 with the slower infusion rate, the infusion should be stopped, and the subject should receive no further cetuximab treatment. If a subject experiences a Grade 3 or 4 infusion reaction at any time, the subject should receive no further cetuximab treatment. If there is any question as to whether an observed reaction is an infusion reaction of Grades 1-4, the Study Chair or Medical Oncology Co-Chair should be contacted immediately to discuss and grade the reaction.

6.2.5 Cetuximab Special Instructions

Weekly cetuximab will continue if radiation therapy is being held. If cetuximab is omitted for more than four consecutive infusions for adverse events due to cetuximab, or for an intercurrent illness (e.g., infection) requiring interruption of therapy, the subject should be discontinued from further cetuximab therapy. If adverse events prevent the administration of cetuximab, the subject may continue to receive radiation therapy.

If a dose of cetuximab is omitted, it will not be made up or added to the end of treatment. The omitted dose and the reason for the omission should be recorded in the site’s source documentation.

Treatment of Isolated Drug Fever

In the event of isolated drug fever, the investigator must use clinical judgment to determine if the fever is related to the study drug or to an infectious etiology. If a patient experiences isolated drug fever, for the next dose, pre-treat with acetaminophen or non-steroidal anti-inflammatory agent (investigator discretion), repeat antipyretic dose 6 and 12 hours after cetuximab infusion. The infusion rate will remain unchanged for future doses

If a patient experiences recurrent isolated drug fever following pre-medication and post-dosing with an appropriate antipyretic, the infusion rate for subsequent dosing should be 50% of previous rate. If fever recurs following infusion rate change, the investigator should assess the patient's level of discomfort with the event and use clinical judgment to determine if the patient should receive further cetuximab.

Cetuximab-related Rash

Manifestations

Rash associated with EGFR-inhibitors is a relatively new dermatologic condition. It appears to be "acneiform" but it is NOT considered a form of acne; rather, it is a form of folliculitis. Skin changes may be manifested in a number of ways: erythema; follicle based papules, which may ulcerate; pain; itching; cosmetic disturbance; and/or nail disorders. The rash may become infected and transform into cellulitis.

Grading of Cetuximab-induced Rash

According to physician judgment, if a patient experiences ≥ grade 3 rash (according to either the "outside of the radiation field" or the "inside of the radiation field" definitions below), cetuximab treatment adjustments should be made according to the Cetuximab Dose Modification table that follows. In patients with mild and moderate skin adverse events, cetuximab should continue without adjustment.

NOTE: Rash intensity (i.e., the size and number of papules or the level of discomfort and extent of erythema) may be an important consideration. However, the absolute number of lesions, **without associated physical discomfort**, does not necessarily constitute a basis for a dose reduction or delay. Rash considered "intolerable" (because of pain, itching, or appearance) or that has failed to respond to symptomatic management may be considered grade 3 and thus prompt dose reduction or delay of cetuximab. **The clinical judgment of the treating physician is critical to grading and will ultimately dictate dose modification.**

Acute Skin Changes

Rash Occurring **Outside** of the Radiation Field: Should be graded using CTCAE, v5.0 terms; common skin toxicities include the following: pruritus, rash/acneiform, paronychia. A rash complicated by secondary infection or cellulitis should be graded per additional CTCAE, v5.0 terms.

Onset of grade 3 will require modification. See the table below, "Cetuximab Dose Modification Guidelines for Dermatologic Changes".

	**1**	**2**	**3**	**4**
**Pruritus**	Mild or localized; topical intervention indicated	Widespread and intermittent; skin changes from scratching (e.g., edema, papulation, excoriations, lichenification, oozing/crusts); oral intervention indicated; limiting instrumental ADL	Widespread and constant; limiting self care ADL or sleep; systemic corticosteroid or immunosuppressive therapy indicated	-
**Rash/acneiform**	Papules and/or pustules covering <10% BSA, which may or may not be associated with symptoms of pruritus or tenderness	Papules and/or pustules covering 10 - 30% BSA, which may or may not be associated with symptoms of pruritus or tenderness; associated with psychosocial impact;	Papules and/or pustules covering >30% BSA with moderate or severe symptoms; limiting self-care ADL; associated with local superinfection with oral antibiotics	Life-threatening consequences; papules and/or pustules covering any % BSA, which may or may not be associated with symptoms of pruritus or

		limiting instrumental ADL; papules and/or pustules covering > 30% BSA with or without mild symptoms	indicated	tenderness and are associated with extensive superinfection with IV antibiotics indicated
**Paronychia**	Nail fold edema or erythema; disruption of the cuticle	Local intervention indicated; oral intervention indicated (e.g., antibiotic, antifungal, antiviral); nail fold edema or erythema with pain; associated with discharge or nail plate separation; limiting instrumental ADL	Operative intervention indicated; IV antibiotics indicated; limiting self care ADL	

Rash Occurring **Inside** the Radiation Field: Acute radiation dermatitis may be exacerbated by cetuximab or chemotherapy. The severity of such rash should be graded using CTCAE, v5.0 criteria for radiation dermatitis (table below).

	1	2	3	4
Radiation recall reaction (dermatologic); Dermatitis radiation	Faint erythema or dry desquamation	Moderate to brisk erythema; patchy moist desquamation, mostly confined to skin folds and creases; moderate edema	Moist desquamation other than skin folds and creases; bleeding induced by minor trauma or abrasion	Life-threatening consequences; skin necrosis or ulceration of full thickness dermis; spontaneous bleeding from involved site; skin graft indicated

Cetuximab Dose Modification Guidelines for Dermatologic Changes (≥ Grade 3)			
	Cetuximab	Outcome	Cetuximab Dose Modification
1st occurrence	Delay infusion 1 to 2 weeks	Improvement to ≤ Grade 2	Continue at 250 mg/m^2
		No Improvement; remains grade 3	Discontinue cetuximab
2nd occurrence	Delay infusion 1 to 2 weeks	Improvement to ≤ Grade 2	Reduce dose to 200 mg/m^2
		No Improvement; remains grade 3	Discontinue cetuximab
3rd occurrence	Delay infusion 1 to 2 weeks	Improvement to ≤ Grade 2	Reduce dose to 150 mg/m^2
		No Improvement; remains grade 3	Discontinue cetuximab
4th occurrence	Discontinue cetuximab		

Drug-Related Rash Management

Patients developing dermatologic adverse events while receiving cetuximab should be monitored for the development of inflammatory or infectious sequelae, and appropriate treatment of these symptoms initiated. Below are suggestions for managing cetuximab-induced rash*:

- **Antibiotics**: The benefit of routine antibiotics in uncomplicated (uninfected) rash is unclear. Some clinicians have used oral minocycline (Minocin), mupirocin (Bactroban), or topical clindamycin (Cleocin). Rash complicated by cellulitis should be treated with appropriate antibiotics based on clinical judgment or microbial sensitivity analysis.
- **Antihistamines**: Benadryl or Atarax may be helpful to control itching.
- **Topical Steroids**: The benefit of topical steroids is unclear. In general, steroids should not be used to treat a rash in the RT field.
- **Retinoids**: No data to support use. Use is not advised.
- **Benzoyl peroxide**: Should NOT be used—may aggravate rash.
- **Makeup**: Rash can be covered with makeup; this should not make it worse (use a dermatologist-approved cover-up, e.g., Dermablend, or any other type of foundation). Remove makeup with a skin-friendly liquid cleanser, e.g., Neutrogena, Dove, or Ivory
- Skin Cleansing Liqui-Gel.
- **Moisturizers**: Use emollients to prevent and alleviate the skin dryness, e.g., Neutrogena Norwegian Formula Hand Cream or Vaseline Intensive Care Advanced Healing Lotion.
- **Sunlight**: It is recommended that patients wear sunscreen and hats and limit sun exposure while receiving cetuximab as sunlight can exacerbate any skin reactions that may occur.
- **Over-the-counter medications**: Over-the-counter acne vulgaris medications (e.g., benzoyl peroxide) are not advised. This rash is not like acne vulgaris and these treatments could make it worse.

*Adapted from Perez-Soler R, Delord J, Halpern A, et al. HER1/EGFR inhibitor-associated rash: Future directions for management and investigation outcomes from the HER1/EGFR Inhibitor Rash Management Forum. *Oncologist*. 2005;10:345-56.

7. ADVERSE EVENTS REPORTING REQUIREMENTS (15-JAN-2019)

7.1 Protocol Agents

<u>Investigational Agent</u>

The investigational agent administered in NRG-HN004, MEDI4736 (durvalumab), is being made available under an IND sponsored by CTEP. For MEDI4736 (durvalumab) and cetuximab, determination of whether an adverse event meets expedited reporting criteria, see the reporting table in Section 7.5 of the protocol. **For Arm 2, MEDI4736 (durvalumab), report all adverse events regardless of relationship to protocol treatment during treatment and for 100 days from last dose of MEDI4736 (durvalumab). During follow-up, only report adverse events reasonably related to protocol treatment.**

<u>Commercial Agent</u>

The commercial agent in NRG-HN004 is cetuximab.

For Arm 1, cetuximab, report all adverse events regardless of relationship to protocol treatment during treatment and for 30 days from end of treatment. During follow-up, only report adverse events reasonably related to protocol treatment.

7.2 Adverse Events and Serious Adverse Events (15-JAN-2019)

7.2.1 Adverse Event Reporting

Lead-In Phase: The lead-in phase will utilize CTCAE version 5.0 for CTEP-AERS reporting. All study case report forms will continue to use CTCAE version 4.0.

Phase II/III: The randomized phase II/III study will utilize CTCAE v5.0 for both CTEP-AERS reporting and study case report forms. Note: for the Phase II/III study, CTEP-AERS reporting is performed via a deep link in Rave (see Section 7.5).

All appropriate treatment areas should have access to a copy of CTCAE versions 4.0 and 5.0, which can be downloaded from the CTEP web site

(https://ctep.cancer.gov/protocolDevelopment/electronic_applications/ctc.htm).

7.2.2 Definition of an Adverse Event (AE)

Any untoward medical occurrence associated with the use of a drug in humans, whether or not considered drug related. Therefore, an AE can be any unfavorable and unintended sign (including an abnormal laboratory finding), symptom, or disease temporally associated with the use of a medicinal (investigational) product, whether or not considered related to the medicinal (investigational) product (attribution of unrelated, unlikely, possible, probable, or definite). (International Conference on Harmonisation [ICH], E2A, E6).

For multi-modality trials, adverse event reporting encompasses all aspects of protocol treatment including radiation therapy, surgery, device, and drug.

Due to the risk of intrauterine exposure of a fetus to potentially teratogenic agents, the pregnancy of a study participant must be reported via CTEP-AERS in an expedited manner.

Clinician graded CTCAE is the AE (adverse event) safety standard. PRO-CTCAE items are to complement CTCAE reporting. Patients will respond to PRO-CTCAE items but no protocol directed action will be taken. The specific PRO-CTCAE items for this protocol can be found on the forms section of the CTSU protocol webpage and is titled "NRG-HN004 NCI PRO-CTCAE Item Library." PRO-CTCAE is not intended for expedited reporting, real time review or safety reporting. PRO-CTCAE data are exploratory and not currently intended for use in data safety monitoring or adverse event stopping rules.

NOTE: PRO-CTCAE data should not be used for determining dose delays or dose modifications or any other protocol directed action.

7.2.3 Solicited Adverse Events and PRO-CTCAE

PRO-CTCAE

The PRO-CTCAE instrument will be used to assess patient reported toxicity outcomes. The United States National Cancer Institute's Patient-Reported Outcomes version of the Common Terminology Criteria for Adverse Events (PRO-CTCAE) has been validated and tested for reliability and responsiveness in 975 cancer patients undergoing chemotherapy or radiotherapy (Dueck 2015). In the HNC population, a study evaluating CRT compared the NCI-CTCAE to the PRO-CTCAE, and found a high concordance between patient-reported and practitioner-reported symptom severity at baseline, when symptoms were absent, but as toxicity increased during therapy, it was noted that clinician reported toxicity was significantly lower than patient reported toxicity. The PRO-CTCAE is included to address tolerability of the treatment regimen from the patients' perspective. It was developed in recognition that collecting symptom data directly from patients using PRO tools can improve the accuracy and efficiency of symptomatic AE data collection. This was based on findings from multiple studies demonstrating that physicians and nurses underestimate symptom onset, frequency, and severity in comparison with patient ratings (Basch 2009, Litwin 1998, Sprangers and Aaronson 1992, Falchook 2016). These symptoms have been converted to patient terms (eg, CTCAE term "myalgia" converted to "aching muscles"). For several symptoms, like fatigue and pain, additional questions are asked about symptom frequency, severity, and interference with usual activities. The items included in the PRO-CTCAE have undergone extensive qualitative review among experts and patients. These items have been extensively evaluated by patients with cancer to be clear and comprehensible, and to measure the symptom of interest. In this study, we have selected 17 of the 78 items, which are only items that are considered relevant for the trial based on toxicity profiles of cetuximab, MEDI4736 (durvalumab), and radiotherapy for head and neck cancer.

PRO-CTCAE is available in English, French (Canada), and Spanish for this study. Patients participating on the electronic patient-reported outcome (Medidata Patient Cloud ePRO) will only have the option to complete the English and Spanish language PRO-CTCAE. French (Canada) language PRO-CTCAE is not currently available on the Medidata Patient Cloud ePRO. French speaking patients will only have the option to complete the PRO-CTCAE on paper. Collection time points are listed in Section 4.

Solicited Adverse Events: The following adverse events are considered "expected" and their presence/absence should be solicited, and severity graded, at baseline and for each cycle of treatment by CTCAE, PRO-CTCAE (for the phase II/III portion of study only), or both.

Assessments will be collected as specified in the Section 4 assessment tables.

CTCAE	PRO-CTCAE Items with (Attributes)
1. Dysphagia<br>2. Mucositis (oral)<br>3. Anorexia<br>4. Nausea<br>5. Vomiting<br>6. Diarrhea<br>7. Abdominal pain<br>8. Dyspnea<br>9. Cough<br>10. Wheezing<br>11. Edema limbs<br>12. Rash maculopapular<br>13. Rash acneiform<br>14. Pruritus<br>15. Dermatitis radiation<br>16. Myalgia<br>17. Fatigue	Oral:<br>1. Difficulty swallowing (S)<br>2. Mouth/Throat sores (SI)<br>Gastrointestinal:<br>3. Decrease appetite (SI)<br>4. Nausea (FS)<br>5. Vomiting (FS)<br>6. Diarrhea (F)<br>7. Abdominal pain (FSI)<br>Respiratory:<br>8. Shortness of breath (SI)<br>9. Cough (SI)<br>10. Wheezing (S)<br>Cardiovascular<br>11. Arm or leg swelling (FSI)<br>Cutaneous:<br>12. Rash (P)<br>13. Acne (S)<br>14. Itching (S)<br>15. Radiation skin reaction (S)<br>Pain:<br>16. Muscle pain (FSI)<br>Sleep/wake:<br>17. Fatigue (SI)

Attributes:
F: Frequency
S: Severity
I: Interference
P: Presence/Absence/Amount

7.3 Comprehensive Adverse Events and Potential Risks (CAEPR) List for MEDI4736 (Durvalumab, NSC 778709) (21-AUG-2019)

The Comprehensive Adverse Events and Potential Risks list (CAEPR) provides a single list of reported and/or potential adverse events (AE) associated with an agent using a uniform presentation of events by body system. In addition to the comprehensive list, a subset, the Specific Protocol Exceptions to Expedited Reporting (SPEER), appears in a separate column and is identified with bold and italicized text. This subset of AEs (SPEER) is a list of events that are protocol specific exceptions to expedited reporting to NCI (except as noted below). Refer to the 'CTEP, NCI Guidelines: Adverse Event Reporting Requirements' http://ctep.cancer.gov/protocolDevelopment/electronic_applications/docs/aeguidelines.pdf for further clarification. *Frequency is provided based on 2833 patients.* Below is the CAEPR for MEDI4736 (durvalumab).

NOTE: Report AEs on the SPEER **ONLY IF** they exceed the grade noted in parentheses next to the AE in the SPEER. If this CAEPR is part of a combination protocol using multiple investigational agents and has an AE listed on different SPEERs, use the lower of the grades to determine if expedited reporting is required.

Version 2.4, April 17, 2019[1]

Adverse Events with Possible Relationship to MEDI4736 (durvalumab) (CTCAE 5.0 Term) [n= 2833]			Specific Protocol Exceptions to Expedited Reporting (SPEER)
**Likely (>20%)**	**Less Likely (<=20%)**	**Rare but Serious (<3%)**	
BLOOD AND LYMPHATIC SYSTEM DISORDERS			
		Blood and lymphatic system disorders - Other (idiopathic thrombocytopenic purpura)[2]	
		Thrombotic thrombocytopenic purpura[2]	
CARDIAC DISORDERS			
		Myocarditis[2]	
		Pericarditis[2]	
ENDOCRINE DISORDERS			
		Adrenal insufficiency[2]	
		Endocrine disorders - Other (diabetes insipidus)	
		Endocrine disorders - Other (diabetes mellitus type 1)[2]	
	Hyperthyroidism[2]		
		Hypopituitarism[2]	
	Hypothyroidism[2]		
EYE DISORDERS			
		Keratitis[2]	
		Uveitis[2]	
GASTROINTESTINAL DISORDERS			
	Abdominal pain		***Abdominal pain (Gr 2)***
		Colitis[2]	
	Diarrhea		***Diarrhea (Gr 2)***
		Gastrointestinal disorders - Other - (gastrointestinal perforation)[2,3]	
	Nausea		***Nausea (Gr 2)***
		Pancreatitis[2]	
	Vomiting		***Vomiting (Gr 2)***
GENERAL DISORDERS AND ADMINISTRATION SITE CONDITIONS			
	Edema limbs		***Edema limbs (Gr 2)***
	Fatigue		***Fatigue (Gr 2)***
	Fever		***Fever (Gr 2)***
HEPATOBILIARY DISORDERS			
		Hepatobiliary disorders - Other (autoimmune hepatitis)[2]	
IMMUNE SYSTEM DISORDERS			
		Immune system disorders - Other (immune related adverse events)[2]	
		Immune system disorders - Other (sarcoidosis)	
INFECTIONS AND INFESTATIONS			

Adverse Events with Possible Relationship to MEDI4736 (durvalumab) (CTCAE 5.0 Term) [n= 2833]			Specific Protocol Exceptions to Expedited Reporting (SPEER)
**Likely (>20%)**	**Less Likely (<=20%)**	**Rare but Serious (<3%)**	
	Infection[4]		*Infection[4] (Gr 2)*
INJURY, POISONING AND PROCEDURAL COMPLICATIONS			
		Infusion related reaction	
INVESTIGATIONS			
	Alanine aminotransferase increased[2]		*Alanine aminotransferase increased[2] (Gr 2)*
	Aspartate aminotransferase increased[2]		*Aspartate aminotransferase increased[2] (Gr 2)*
	Creatinine increased		*Creatinine increased (Gr 2)*
METABOLISM AND NUTRITION DISORDERS			
	Anorexia		*Anorexia (Gr 2)*
MUSCULOSKELETAL AND CONNECTIVE TISSUE DISORDERS			
	Arthritis[2]		
		Musculoskeletal and connective tissue disorder - Other (polymyositis)[2]	
	Myalgia		*Myalgia (Gr 2)*
		Myositis[2]	
NERVOUS SYSTEM DISORDERS			
		Guillain-Barre syndrome[2,5]	
		Myasthenia gravis[2]	
		Nervous system disorders - Other (aseptic meningitis)[2]	
		Peripheral sensory neuropathy	
RENAL AND URINARY DISORDERS			
	Dysuria		*Dysuria (Gr 2)*
		Renal and urinary disorders - Other (autoimmune nephritis)[2]	
RESPIRATORY, THORACIC AND MEDIASTINAL DISORDERS			
Cough			*Cough (Gr 2)*
	Dyspnea		*Dyspnea (Gr 2)*
	Pneumonitis[2]		
	Respiratory, thoracic and mediastinal disorders - Other (dysphonia)		
SKIN AND SUBCUTANEOUS TISSUE DISORDERS			
	Hyperhidrosis		
	Pruritus		*Pruritus (Gr 2)*
	Rash[2,6]		*Rash[2,6] (Gr 2)*
		Skin and subcutaneous tissue disorders - Other (scleroderma)	
		Skin and subcutaneous tissue disorders - Other (severe dermatitis)[2,7]	
	Skin hypopigmentation		*Skin hypopigmentation (Gr 2)*

NOTE: Cardiomyopathy, and graft versus host disease, while not observed on clinical trials of MEDI4736 (durvalumab) at this time, are known events with this class of agent (PD-L1 antagonist).

[1]This table will be updated as the toxicity profile of the agent is revised. Updates will be distributed to all Principal Investigators at the time of revision. The current version can be obtained by contacting PIO@CTEP.NCI.NIH.GOV. Your name, the name of the investigator, the protocol and the agent should be included in the e-mail.

[2]Immune-mediated adverse reactions (irAEs) have been reported in patients receiving MEDI4736 (durvalumab). irAEs can involve any of the organs or systems in the body. Most irAEs were reversible and managed with interruptions of MEDI4736 (durvalumab), administration of corticosteroids and supportive care, however, these events can be serious and fatal.

[3]Gastrointestinal perforations have been observed only in patients receiving MEDI4736 (durvalumab) in combination with tremelimumab (CP-675,206).

[4]Infections includes infection in the lungs, upper respiratory tract, dental and oral soft tissues and other organs under the INFECTIONS AND INFESTATIONS SOC. Infections generally are mild (Gr 1-2) but severe infections including sepsis, necrotizing fasciitis, and osteomyelitis have been reported.

[5]Guillain-Barre Syndrome has been reported in patients receiving MEDI4736 (durvalumab) in combination with tremelimumab (CP-675,206) but can potentially occur after durvalumab monotherapy.

[6]Rash includes the terms: rash erythematous, rash generalized, rash macular, rash maculopapular, rash papular, rash pruritic, rash pustular, erythema, and eczema.

[7]In rare cases, severe dermatitis has been reported to manifest as Stevens-Johnson syndrome, toxic epidermal necrolysis, or rashes complicated by dermal ulceration or necrotic, bullous, or hemorrhagic manifectations.

Adverse events reported on MEDI4736 (durvalumab) trials, but for which there is insufficient evidence to suggest that there was a reasonable possibility that MEDI4736 (durvalumab) caused the adverse event:

BLOOD AND LYMPHATIC SYSTEM DISORDERS - Anemia; Disseminated intravascular coagulation
CARDIAC DISORDERS - Atrial fibrillation; Atrial flutter; Cardiac disorders - Other (coronary artery disease); Pericardial effusion; Pericardial tamponade; Restrictive cardiomyopathy; Right ventricular dysfunction; Sinus tachycardia
EAR AND LABYRINTH DISORDERS - Hearing impaired
EYE DISORDERS - Eye disorders - Other (choroidal effusion with shut down of ciliary body)
GASTROINTESTINAL DISORDERS - Ascites; Constipation; Dental caries; Gastrointestinal disorders - Other (gastrointestinal hemorrhage); Mucositis oral; Proctitis; Small intestinal obstruction; Upper gastrointestinal hemorrhage
GENERAL DISORDERS AND ADMINISTRATION SITE CONDITIONS - Edema trunk; Non-cardiac chest pain; Pain
HEPATOBILIARY DISORDERS - Hepatic hemorrhage
IMMUNE SYSTEM DISORDERS - Immune system disorders - Other (drug-induced liver injury); Serum sickness
INJURY, POISONING AND PROCEDURAL COMPLICATIONS - Wound complication
INVESTIGATIONS - Blood bilirubin increased; CPK increased; Electrocardiogram T wave abnormal; GGT increased; Lipase increased; Lymphocyte count decreased; Neutrophil count decreased; Platelet count decreased; Serum amylase increased; Weight loss; White blood cell decreased
METABOLISM AND NUTRITION DISORDERS - Dehydration; Hypercalcemia; Hyperglycemia; Hyperkalemia; Hypermagnesemia; Hypoalbuminemia; Hypokalemia; Hypomagnesemia; Hyponatremia
MUSCULOSKELETAL AND CONNECTIVE TISSUE DISORDERS - Arthralgia; Back pain; Rhabdomyolysis
NEOPLASMS BENIGN, MALIGNANT AND UNSPECIFIED (INCL CYSTS AND POLYPS) - Neoplasms benign, malignant and unspecified (incl cysts and polyps) - Other (brain metastasis swelling); Neoplasms benign,

malignant and unspecified (incl cysts and polyps) - Other (lung cyst); Neoplasms benign, malignant and unspecified (incl cysts and polyps) - Other (tumor flare, tumor inflammation); Treatment related secondary malignancy; Tumor hemorrhage; Tumor pain
NERVOUS SYSTEM DISORDERS - Ataxia; Dizziness; Edema cerebral; Headache; Nervous system disorders - Other (axonal neuropathy); Nervous system disorders - Other (hemiparesis); Paresthesia; Seizure
PSYCHIATRIC DISORDERS - Confusion
RENAL AND URINARY DISORDERS - Acute kidney injury
RESPIRATORY, THORACIC AND MEDIASTINAL DISORDERS - Bronchopulmonary hemorrhage; Hypoxia; Pleural effusion; Pneumothorax; Respiratory failure
SKIN AND SUBCUTANEOUS TISSUE DISORDERS - Bullous dermatitis; Dry skin
VASCULAR DISORDERS - Hypertension

Note: MEDI4736 (durvalumab) in combination with other agents could cause an exacerbation of any adverse event currently known to be caused by the other agent, or the combination may result in events never previously associated with either agent.

7.4 Adverse Events for Commercial Study Agents: Cetuximab
Refer to the package insert for detailed pharmacologic and safety information
Common adverse events include**:** infusion reaction; nausea, vomiting, diarrhea; weight; loss; abnormal liver function tests; rash acneiform; radiation dermatitis.

7.5 Expedited Reporting of Adverse Events (21-AUG-2019)
Lead-In Phase: All serious adverse events that meet expedited reporting criteria defined in the reporting table below will be reported via the CTEP Adverse Event Reporting System, CTEP-AERS, accessed via the CTEP web site,
https://eapps-ctep.nci.nih.gov/ctepaers/pages/task?rand=1390853489613

Phase II/III: All adverse events are submitted for expedited reporting protocol-specific rules evaluation using the Medidata Rave data management system. All AEs must first be entered in Rave and will undergo CTEP-AERS rules evaluation to determine whether expedited reporting is recommended based on a set of programmed expedited reporting rules. AEs identified as meeting the programmed expedited reporting requirements can then be submitted in CTEP-AERS. A deep link in Rave will take the user directly to CTEP-AERS where the expedited report may be completed and submitted via CTEP-AERS.

All serious adverse events that meet expedited reporting criteria defined in the reporting table below will be reported via the CTEP Adverse Event Reporting System, CTEP-AERS, accessed via the link in Rave. CTEP-AERS is also accessed via the CTEP web site, but all expedited reports must be initiated in RAVE
https://eapps-ctep.nci.nih.gov/ctepaers/pages/task?rand=1390853489613

In the rare event when Internet connectivity is disrupted, a 24-hour notification must be made to the NRG Oncology by phone at 1-215-574-3191. An electronic report must be submitted immediately upon re-establishment of the Internet connection.

7.5.1 Expedited Reporting Methods

- “Per CTEP NCI Guidelines for Adverse Events Reporting, a CTEP-AERS 24-hour notification must be submitted within 24 hours of learning of the adverse event. CTEP-AERS 24-hour notification must be followed by a complete report within 5 days.
- Supporting source documentation is requested by CTEP or NRG as needed to complete adverse event review. Supporting source documentation should include the protocol number, patient ID number, and CTEP-AERS ticket number on each page. Contact NRG Oncology at 1-215-574-3191 for details to submit source documentation (CTEP fax: 301-897-7404).
- A serious adverse event that meets expedited reporting criteria outlined in the AE Reporting Tables but is assessed by the CTEP-AERS as “an action *not* recommended” must still be reported to fulfill NRG safety reporting obligations. Sites must bypass the “NOT recommended” assessment; the CTEP-AERS allows submission of all reports regardless of the results of the assessment.

7.5.2 CTEP Expedited Reporting Requirements for Adverse Events

For the Lead-In and Arms 1 and 2 of the Phase 2/3: Expedited Reporting Requirements for Adverse Events that Occur within 30 Days of the Last Administration of Cetuximab or RT and within 100 Days of the Last Administration of MEDI4736 (durvalumab)[1, 2]

FDA REPORTING REQUIREMENTS FOR SERIOUS ADVERSE EVENTS (21 CFR Part 312)

NOTE: Investigators **MUST** immediately report to the sponsor (NCI) **ANY** Serious Adverse Events, whether or not they are considered related to the investigational agent(s)/intervention (21 CFR 312.64)

An adverse event is considered serious if it results in **ANY** of the following outcomes:

1) Death
2) A life-threatening adverse event
3) An adverse event that results in inpatient hospitalization or prolongation of existing hospitalization for ≥ 24 hours
4) A persistent or significant incapacity or substantial disruption of the ability to conduct normal life functions
5) A congenital anomaly/birth defect.
6) Important Medical Events (IME) that may not result in death, be life threatening, or require hospitalization may be considered serious when, based upon medical judgment, they may jeopardize the patient or subject and may require medical or surgical intervention to prevent one of the outcomes listed in this definition. (FDA, 21 CFR 312.32; ICH E2A and ICH E6).

ALL SERIOUS adverse events that meet the above criteria **MUST** be immediately reported to the NCI via electronic submission within the timeframes detailed in the table below.

<table>
<tr><th>Hospitalization</th><th>Grade 1 Timeframes</th><th>Grade 2 Timeframes</th><th>Grade 3 Timeframes</th><th>Grade 4 & 5 Timeframes</th></tr>
<tr><td>Resulting in Hospitalization ≥ 24 hrs</td><td colspan="3">10 Calendar Days</td><td rowspan="2">24-Hour 5 Calendar Days</td></tr>
<tr><td>Not resulting in Hospitalization ≥ 24 hrs</td><td colspan="2">Not required</td><td>10 Calendar Days</td></tr>
</table>

NOTE: Protocol specific exceptions to expedited reporting of serious adverse events are found in the Specific Protocol Exceptions to Expedited Reporting (SPEER) portion of the CAEPR

Expedited AE reporting timelines are defined as:

- "24-Hour; 5 Calendar Days" - The AE must initially be submitted electronically within 24 hours of learning of the AE, followed by a complete expedited report within 5 calendar days of the initial 24-hour report.
- "10 Calendar Days" - A complete expedited report on the AE must be submitted electronically within 10 calendar days of learning of the AE.

[1]Serious adverse events that occur more than 30 days after the last administration of protocol treatment (Arm 1) and more than 100 days after the last administration of MEDI4736 (durvalumab) (safety lead-in and Arm 2) and have an attribution of possible, probable, or definite require reporting as follows:

Expedited 24-hour notification followed by complete report within 5 calendar days for:

- All Grade 4, and Grade 5 AEs

Expedited 10 calendar day reports for:

- Grade 2 adverse events resulting in hospitalization or prolongation of hospitalization
- Grade 3 adverse events

[2]For studies using PET or SPECT IND agents, the AE reporting period is limited to 10 radioactive half-lives, rounded UP to the nearest whole day, after the agent/intervention was last administered. Footnote "1" above applies after this reporting period.

Effective Date: May 5, 2011

Additional Protocol-Specific Instructions or Exceptions to Expedited Reporting Requirements

Clinician graded CTCAE is the AE safety standard. PRO-CTCAE items are to complement CTCAE reporting. Patients will respond to PRO-CTCAE items but no protocol directed action will be taken. The

study-specific PRO-CTCAE items for this protocol can be found on the forms section of the CTSU protocol webpage and is titled "NRG-HN004 NCI PRO-CTCAE Item Library"

7.5.3 Reporting to the Site IRB/REB

Investigators will report serious adverse events to the local Institutional Review Board (IRB) or Research Ethics Board (REB) responsible for oversight of the patient according to institutional policy.

7.5.4 Secondary Malignancy

A secondary malignancy is a cancer caused by treatment for a previous malignancy (e.g., treatment with investigational agent/intervention, radiation or chemotherapy). A secondary malignancy is not considered a metastasis of the initial neoplasm.

CTEP requires all secondary malignancies that occur during or subsequent to treatment with an agent under an NCI IND/IDE be reported via CTEP-AERS. In addition, secondary malignancies following radiation therapy must be reported via CTEP-AERS. Three options are available to describe the event:

- Leukemia secondary to oncology chemotherapy (e.g., acute myelocytic leukemia [AML])
- Myelodysplastic syndrome (MDS)
- Treatment-related secondary malignancy

Any malignancy possibly related to cancer treatment (including AML/MDS) should also be reported via the routine reporting mechanisms outlined in each protocol.

Second Malignancy:

A second malignancy is one unrelated to the treatment of a prior malignancy (and is NOT a metastasis from the initial malignancy). Second malignancies require ONLY routine reporting via CDUS unless otherwise specified.

7.6 Routine Reporting Requirements for Adverse Events (15-JAN-2019)

All Adverse Events **must** be reported in routine study data submissions. For the Lead-In, AEs reported expeditiously through CTEP-AERS must also be reported in routine study data submissions. For the Phase II/III, all AEs must first be entered in Rave and will undergo CTEP-AERS rules evaluation to determine whether expedited reporting is recommended based on a set of programmed expedited reporting rules. AEs identified as meeting the programmed expedited reporting requirements can then be submitted in CTEP-AERS via a deep link in Rave.

7.6.1 Reporting PRO-CTCAE

Symptomatic Adverse Events reported by patients through PRO-CTCAE are not safety reporting and should also be clinician graded using the CTCAE v5.0 and reported as routine AE data.

7.7 Pregnancy (15-JAN-2019)

Although not an adverse event in and of itself, pregnancy as well as its outcome must be documented via **CTEP-AERS**. In addition, the ***Pregnancy Information Form*** included within the NCI Guidelines for Adverse Event Reporting Requirements must be completed and submitted to CTEP. Any pregnancy occurring in a patient or patient's partner from the time of consent to 6 months after the last dose of either cetuximab or MEDI4736 (durvalumab) must be reported and then followed for outcome. Newborn infants should be followed until 30 days old. Please see the "NCI Guidelines for Investigators: Adverse Event Reporting Requirements for DCTD (CTEP and CIP) and DCP INDs and IDEs" (at http://ctep.cancer.gov/protocolDevelopment/adverse_effects.htm) for more details on how to report pregnancy and its outcome to CTEP.

8. REGISTRATION AND STUDY ENTRY PROCEDURES

8.1 CTEP Registration Procedures and Access requirements for OPEN, Medidata Rave, and TRIAD (13-JAN-2021)

Food and Drug Administration (FDA) regulations and National Cancer Institute (NCI) policy require all individuals contributing to NCI-sponsored trials to register and renew their registration annually. To

register, all individuals must obtain a Cancer Therapy Evaluation Program (CTEP) Identity and Access Management (IAM) account (https://ctepcore.nci.nih.gov/iam). In addition, persons with a registration type of Investigator (IVR), Non-Physician Investigator (NPIVR), or Associate Plus (AP) must complete their annual registration using CTEP's web-based Registration and Credential Repository (RCR) at https://ctepcore.nci.nih.gov/rcr.

RCR utilizes five person registration types.

- IVR — MD, DO, or international equivalent;
- NPIVR — advanced practice providers (e.g., NP or PA) or graduate level researchers (e.g., PhD);
- AP — clinical site staff (e.g., RN or CRA) with data entry access to CTSU applications such as the Roster Update Management System (RUMS), OPEN, Rave acting as a primary site contact, or with consenting privileges;
- Associate (A) — other clinical site staff involved in the conduct of NCI-sponsored trials; and
- Associate Basic (AB) — individuals (e.g., pharmaceutical company employees) with limited access to NCI-supported systems.

RCR requires the following registration documents:

**Documentation Required**	**IVR**	**NPIVR**	**AP**	**A**	**AB**
FDA Form 1572	✔	✔			
Financial Disclosure Form	✔	✔	✔		
NCI Biosketch (education, training, employment, license, and certification)	✔	✔	✔		
GCP training	✔	✔	✔		
Agent Shipment Form (if applicable)	✔				
CV (optional)	✔	✔	✔		

An active CTEP-IAM user account and appropriate RCR registration is required to access all CTEP and CTSU (Cancer Trials Support Unit) websites and applications. In addition, IVRs and NPIVRs must list all clinical practice sites and IRBs covering their practice sites on the FDA Form 1572 in RCR to allow the following:

- Added to a site roster;
- Assigned the treating, credit, consenting, or drug shipment (IVR only) tasks in OPEN;
- Act as the site-protocol Protocol Investigator (PI) on the IRB approval; and
- Assigned the Clinical Investigator (CI) role on the Delegation of Tasks Log (DTL).

In addition, all investigators acting as the site-protocol PI (investigator listed on the IRB approval), consenting/treating/drug shipment investigator in OPEN, or as the CI on the DTL must be rostered at the enrolling site with a participating organization.

Additional information is located on the CTEP website at https://ctep.cancer.gov/investigatorResources/default.htm. For questions, please contact the ***RCR Help Desk*** by email at RCRHelpDesk@nih.gov.

8.2 CTSU Registration Procedures (09-MAR-2022)

This study is supported by the NCI Cancer Trials Support Unit (CTSU).

8.2.1 IRB Approval

For CTEP and Division of Cancer Prevention (DCP) studies open to the National Clinical Trials Network (NCTN) and NCI Community Oncology Research Program (NCORP) Research Bases after March 1, 2019, all U.S.-based sites must be members of the NCI Central Institutional Review Board (NCI CIRB). In addition, U.S.-based sites must accept the NCI CIRB review to activate new studies at the site after March 1, 2019. Local IRB review will continue to be accepted for studies that are not reviewed by the CIRB, or if the study was previously open at the site under the local IRB. International sites should continue to submit Research Ethics Board (REB) approval to the CTSU Regulatory Office following country-specific regulations.

Sites participating with the NCI CIRB must submit the Study Specific Worksheet (SSW) for Local Context to the CIRB using IRBManager to indicate their intent to open the study locally. The NCI CIRB's approval of the SSW is automatically communicated to the CTSU Regulatory Office, but sites are required to contact the CTSU Regulatory Office at CTSURegPref@ctsu.coccg.org to establish site preferences for applying NCI CIRB approvals across their Signatory Network. Site preferences can be set at the network or protocol level. Questions about establishing site preferences can be addressed to the CTSU Regulatory Office by email or calling 1-888-651-CTSU (2878).

In addition, the site-protocol Principal Investigator (PI) (i.e. the investigator on the IRB/REB approval) must meet the following criteria in order for the processing of the IRB/REB approval record to be completed:

- Holds an active CTEP status;
- Active status at the site(s) on the IRB/REB approval (applies to US and Canadian sites only) on at least one participating organization's roster;
- If using NCI CIRB, active on the NCI CIRB roster under the applicable CIRB Signatory Institutions(s) record;
- Includes the IRB number of the IRB providing approval in the Form FDA 1572 in the RCR profile;
- Lists all sites on the IRB/REB approval as Practice Sites in the Form FDA 1572 in the RCR profile; and
- Holds the appropriate CTEP registration type for the protocol.

Additional Requirements for NRG-HN004 Site Registration

Additional site requirements to obtain an approved site registration status include:

- An active Federal Wide Assurance (FWA) number;
- An active roster affiliation with the Lead Protocol Organization (LPO) or a Participating Organization (PO);
- A valid IRB approval;
- An active roster affiliation with the NCI CIRB roster under at least one CIRB Signatory Institution (US sites only);
- This is a study with a radiation and/or imaging (RTI) component and the enrolling site must be aligned to an RTI provider (see further information below).
- Delegation of Task Log (DTL) – see section 8.2.4 for further instructions.
- IROC Credentialing Status Inquiry (CSI) Form – this form is submitted to IROC to begin the modality credentialing process.
    - Credentialing documentation received from IROC Houston for this trial- See Section 8.3 Table for details.
- Compliance with all protocol specific requirements (PSRs).

<u>RTI Provider</u>

This is a study with a radiation and/or imaging (RTI) component and the enrolling site must be aligned

to an RTI provider. To manage provider associations or to add or remove associated providers, access the Provider Association page from the Regulatory section on the CTSU members' website at https://www.ctsu.org/RSS/RTFProviderAssociation. Sites must be linked to at least one Imaging and Radiation Oncology Core (IROC) provider to participate on trials with an RTI component. Enrolling sites are responsible for ensuring that the appropriate agreements and IRB approvals are in place with their RTI provider. An individual with a primary role on a treating site roster can update the provider associations, though all individuals at a site may view provider associations. To find who holds primary roles at your site, view the Person Roster Browser under the RUMS section on the CTSU website.

To complete protocol-specific credentialing the RTI provider or enrolling site should follow instructions in the section 8.3 of protocol to submit documentation or other materials to the designated IROC Quality Assurance (QA) center. IROC Houston QA Center will notify the site that all desired credentialing requirements have been met. The site will need to upload a PDF of approval email from IROC Houston to the CTSU Regulatory Portal for RSS to be updated.

Upon site registration approval in RSS, the enrolling site may access OPEN to complete enrollments. The enrolling site will select their credentialed provider treating the subject in the OPEN credentialing screen and may need to answer additional questions related to treatment in the eligibility checklist.

8.2.2 Downloading Site Registration Documents:

Download the site registration forms from the NRG-HN004 protocol page located on the CTSU members' website. Permission to view and download this protocol and its supporting documents is restricted to institutions and its associated investigators and staff on a participating roster. To view/download site registration forms:

- Log in to the CTSU members' website (https://www.ctsu.org) using your CTEP-IAM username and password;
- Click on *Protocols* in the upper left of the screen:
  - Enter the protocol number in the search field at the top of the protocol tree; or
  - Click on the By Lead Organization folder to expand, then select *NRG*, and protocol number *NRG-HN004*.
- Click on *Documents*, *Protocol Related Documents*, and use the *Document Type* filter and select *Site Registration* to download and complete the forms provided. (Note: For sites under the CIRB, IRB data will load automatically to the CTSU.)

8.2.3 Submitting Regulatory Documents:

Submit required forms and documents to the CTSU Regulatory Office using the Regulatory Submission Portal on the CTSU members' website.

To access the Regulatory Submission Portal, log in to the CTSU members' website, go to the *Regulatory* section and select *Regulatory Submission*.

Institutions with patients waiting that are unable to use the Portal should alert the CTSU Regulatory Office immediately by phone or email: 1-866-651-CTSU (2878), or CTSURegHelp@coccg.org in order to receive further instruction and support.

8.2.4 Delegation of Task Log (DTL)

Each site must complete a protocol-specific DTL. The Clinical Investigator (CI) is required to review and electronically sign the DTL prior to the site receiving an approved site registration status and enrolling patients to the study. To maintain an approved site registration status the CI must re-sign the DTL at least annually and when a new version of the DTL is released; and to activate new task assignments requiring CI sign-off. Any individual at the enrolling site on a participating roster may initiate the site DTL. Once the DTL is submitted for CI approval, only the designated DTL Administrators or the CI may update the DTL. Instructions on completing the DTL are available in the Help Topics button in the DTL application and include a Master Task List, which describes DTL task assignments, CI signature, and CTEP registration

requirements.

Canadian sites participating under the Canadian Cancer Trials Group (CCTG), should complete the DTL in CCTG's Ripple application when CCTG holds the Clinical Trials Agreement with Health Canada. Ripple is integrated with the CTSU DTL application for this trial.

8.2.5 Checking Site's Registration Status:

Site registration status may be verified on the CTSU members' website.

Click on *Regulatory* at the top of the screen;

Click on *Site Registration*; and

Enter the site's 5-character CTEP Institution Code and click on Go:

- o Additional filters are available to sort by Protocol, Registration Status, Protocol Status, and/or IRB Type.

Note: The status shown only reflects institutional compliance with site registration requirements as outlined within the protocol. It does not reflect compliance with protocol requirements for individuals participating on the protocol or the enrolling investigator's status with NCI or their affiliated networks.

8.3 RT-Specific Pre-Registration Requirements (09-MAR-2022)

For detailed information on the specific technology requirement required for this study, please refer to the table below and utilize the web link provided for detailed instructions. The check marks under the treatment modality columns indicate whether that specific credentialing requirement is required for this study. Specific credentialing components may require you to work with various QA centers; however, the IROC Houston QA Center will notify your institution and NRG Headquarters when all credentialing requirements have been met and the institution is RT credentialed to enter patients onto this study.

**Credentialing Requirements**	**Web Link for Procedures and Instructions: [www.irochouston.meanderson.org**	
	**Treatment Modality**	**Key Information**
	**Photons**	
Facility Questionnaire	✓	The IROC Houston electronic facility questionnaire (FQ) should be completed or updated with the most recent information about your institution. To access this FQ, email http://irochouston.mdanderson.org to receive your FQ link.
Credentialing Status Inquiry Form	✓	To determine if your institution has completed the requirements above, please complete a "Credentialing Status Inquiry Form" found under Credentialing on the IROC Houston QA Center website (http://irochouston.mdanderson.org).
Phantom Irradiation	✓	An IMRT Head and neck phantom irradiation provided by the IROC Houston QA Center must be successfully completed. Instructions for requesting and irradiating the phantom are found on the IROC Houston web site (http://irochouston.mdanderson.org**)**. Note that an institution, depending on its treatment delivery modalities, may be required to irradiate a phantom on different delivery machines such as TomoTherapy.
IGRT Verification Study	✓	Institutions must be credentialed for bony tissue IGRT by IROC Houston. Find details on the IROC Houston QA Center website (http://irochouston.mdanderson.org) Institutions that have previously been approved for IGRT may not need to repeat credentialing.
**Credentialing Issued to:**		
Institution		IROC Houston QA Center will notify the site that all desired credentialing requirements have been met. The site will need to upload a PDF of approval email from IROC Houston to the

		CTSU Regulatory Portal for RSS to be updated.

8.3.1 Digital RT Data Submission to NRG Using TRIAD

Transfer of Images and Data (TRIAD) is the American College of Radiology's (ACR) image exchange application. TRIAD provides sites participating in clinical trials a secure method to transmit images. TRIAD anonymizes and validates the images as they are transferred.

<u>TRIAD Access Requirements</u>:

- A valid Cancer Therapy Evaluation Program Identity and Access Management (CTEP-IAM) account.
- Registration type of: Associate (A), Associate Plus (AP), Non-Physician Investigator (NPIVR), or Investigator (IVR) registration type. Refer to the CTEP Registration Procedures section for instructions on how to request a CTEP-IAM account and complete registration in RCR.
- TRIAD Site User role on an NCTN or ETCTN roster.

All individuals on the Imaging and Radiation Oncology Core provider roster have access to TRIAD and may submit images for credentialing purposes, or for enrollments to which the provider is linked in OPEN.

<u>TRIAD Installations</u>:

To submit images, the individual holding the TRIAD Site User role will need to install the TRIAD application on their workstation. TRIAD installation documentation is available at https://triadinstall.acr.org/triadclient/.

This process can be done in parallel to obtaining your CTEP-IAM account and RCR registration.

For questions, contact TRIAD Technical Support staff via email TRIAD-Support@acr.org or 1-703-390-9858.

8.4 Patient Enrollment (09-MAR-2022)

Patient registration can occur only after evaluation for eligibility is complete, eligibility criteria have been met, and the study site is listed as 'approved' in the CTSU RSS. Patients must have signed and dated all applicable consents and authorization forms.

8.4.1 Oncology Patient Enrollment Network (OPEN)

Patient enrollment for this **2-step (registration and randomization)** trial will be facilitated using the Oncology Patient Enrollment Network (OPEN). OPEN is a web-based registration system available on a 24/7 basis. OPEN is integrated with CTSU regulatory and roster data and with the LPOs registration/randomization systems or the Theradex Interactive Web Response System (IWRS) for retrieval of patient registration/randomization assignment. OPEN will populate the patient enrollment data in NCI's clinical data management system, Medidata Rave.

Requirements for OPEN access:

- A valid CTEP-IAM account;
- To perform enrollments: Must be on an LPO roster, ETCTN corresponding roster, or participating

organization roster with the role of Registrar. Registrars must hold a minimum of an Associate Plus (AP) registration type;

- If a Delegation of Tasks Log (DTL) is required for the study, the registrars must hold the OPEN Registrar task on the DTL for the site; and
- Have an approved site registration for the protocol prior to patient enrollment.

To assign an IVR or NPIVR as the treating, crediting, consenting, drug shipment (IVR only), or investigator receiving a transfer in OPEN, the IVR or NPIVR must list the IRB number used on the site's approval on their Form FDA 1572 in RCR. If a DTL is required for the study, the IVR or NPIVR must be assigned the appropriate OPEN-related tasks on the DTL.

Prior to accessing OPEN site staff should verify the following:

- Patient has met all eligibility criteria within the protocol stated timeframes; and
- All patients have signed an appropriate consent form and Health Insurance Portability and Accountability Act (HIPAA) authorization form (if applicable).

Note: The OPEN system will provide the site with a printable confirmation of registration and treatment information. You may print this confirmation for your records.

Access OPEN at https://open.ctsu.org or from the OPEN link on the CTSU members' website. Further instructional information is in the OPEN section of the CTSU website at https://www.ctsu.org or at https://open.ctsu.org. For any additional questions, contact the CTSU Help Desk at 1-888-823-5923 or ctsucontact@westat.com.

NOTE: Step 2 registration requires a second web registration for all patients.
If a patient is not going on to randomization, Step 2 registration must still be completed via OPEN and the reason why the patient will not go on study must be provided.

8.4.2 Summary of Registration Procedures

This is a 2-step registration study.

- All eligibility criteria (except central p16 review*) must be met prior to Step 1 registration (*central p16 review applicable to Oropharynx and Unknown primary only).
- All patients will be registered to Step 1.
    - For Oropharynx and Unknown primary patients, tissue submission is required for the central p16 review after Step 1 registration has been completed and a case number assigned (refer to section 10 for details on tissue submission).
        - The central review will take approximately 1-2 business days from receipt of samples.
        - NRG Oncology will notify the sites via an e-mail once the p16 results have been received. Sites can complete Step 2 registration at this time.
    - For Oral cavity, Hypopharynx, and Larynx primary patients, sites must complete step II registration the following day. NRG requires the time to calculate the stratification, which is required for randomization.

8.5 Medidata Patient Cloud ePRO Registration (13-JAN-2021)

This study includes the use of Medidata Patient Cloud ePRO (electronic patient-reported outcomes). After the patient is registered to the trial via OPEN, and if the patient is willing to participate in electronic data collection, the site staff will then complete a registration for the patient to the Patient Cloud ePRO through iMedidata. Note: Site staff must have already completed required eLearning for the Patient Cloud ePRO application to register a patient and information about the training is in the ePRO Appendix. The registration to the Patient Cloud ePRO will create a unique patient registration code that the site staff will provide to the patient. The patient (with assistance from the site staff) should be instructed to download the Patient Cloud ePRO app onto his/her own device (IOS, Android, phone or tablet) and use the unique patient registration code to create an account. Once the patient's account is set up, the patient will be able to complete the submission of patient reported outcomes electronically for the trial. There are multiple versions of the app available. The **Patient Cloud** App will be used on this study. Ensure that the patient downloads the correct version of the ePRO app. Note only 1 version of the app is active per protocol.

For sites providing a shared institutional device for use by multiple patients on site:

- The site staff should assist the patient with access and registration to the Patient Cloud ePRO app, and the patient can then complete the electronic data submission independently. Site staff may need to assist patients with logging on to the device at each visit.

8.5.1 CRA Patient Registration Instructions for ePRO

Please visit the CTSU website for reference information.

i. The subject registration process starts in iMedidata. Begin by selecting the Patient Cloud ePRO Registration link for your study

ii. The patient management app will display, select your STUDY and SITE from the drop downs and click Launch.

iii. Now you can register your first patient. Create a subject ID and select a Country / Language from the drop down, (these are the only required data fields). The subject initials are optional, but are helpful in identifying which subject ID maps with which activation code. When finished, click Add.

iv. The subject added and will include the date the patient was added, the subject ID, subject initials, (if included) and a unique auto-generated activation code. The activation code is unique for each patient and linked to the subject ID, it is not interchangeable. In addition, there is a status section, which indicates if the patient has registered. When the patient has registered the status will change from "invited" to "registered".

Reminder- site staff must have already completed the Medidata Patient Cloud training in order to register study participants. Please visit the CTSU website for reference information.

9. DRUG INFORMATION

9.1 Investigational Study Agent: MEDI4736 (Durvalumab) (NSC 778709, IND 134416) (09-MAR-2022)

9.1.1 Agent Ordering and Agent Accountability

NCI-supplied agents may be requested by eligible participating Investigators (or their authorized designee) at each participating institution. The CTEP-assigned protocol number must be used for ordering all CTEP-supplied investigational agents. The eligible participating investigators at each participating institution must be registered with CTEP, DCTD through an annual submission of FDA Form 1572 (Statement of Investigator), NCI Biosketch, Agent Shipment Form, and Financial Disclosure Form (FDF). If there are several participating investigators at one institution, CTEP-supplied investigational agents for the study should be ordered under the name of one lead investigator at that institution. Active CTEP-registered investigators and investigator-designated shipping designees and ordering designees can submit agent requests through the PMB Online Agent Order Processing (OAOP) application. Access to OAOP requires the establishment of a CTEP Identity and Access Management (IAM) account and the maintenance of an “active” account status and a “current” password. For questions about drug orders, transfers, returns, or accountability, call or email PMB any time. Refer to the PMB’s website for specific policies and guidelines related to agent management.

9.1.2 Subjects must be enrolled and assigned to the MEDI4736 (durvalumab) treatment arm prior to submitting the agent request to PMB.

Refer to the Policy and Guidelines for Investigational Agent Ordering **and the contact information below for order processing time and conditions. Normal order processing time is two business days. An express courier account number must be provided for next-day delivery.**

CTEP Forms, Templates, Documents: http://ctep.cancer.gov/forms/
NCI CTEP Investigator Registration: RCRHelpDesk@nih.gov
PMB policies and guidelines: http://ctep.cancer.gov/branches/pmb/agent_management.htm
PMB Online Agent Order Processing (OAOP) application: https://ctepcore.nci.nih.gov/OAOP
CTEP Identity and Access Management (IAM) account: https://ctepcore.nci.nih.gov/iam/
CTEP IAM account help: ctepreghelp@ctep.nci.nih.gov
PMB email: PMBAfterHours@mail.nih.gov
PMB phone and hours of service: (240) 276-6575 Monday through Friday between 8:30 am and 4:30 pm (ET)

The investigator, or a responsible party designated by the investigator, must maintain a careful record of the receipt, dispensing and final disposition of all agents received from the PMB using the appropriate NCI

Investigational Agent (Drug) Accountability Record (DARF) available on the CTEP forms page. Store and maintain separate NCI Investigational Agent Accountability Records for each agent, strength, formulation and ordering investigator on this protocol.

9.1.3 Agent-Specific Information

To supplement the toxicity information contained in this document, investigators must obtain the current version of the investigator brochure (IB), if available, for comprehensive pharmacologic and safety information. The current version of the Investigator Brochure (IB) will be accessible to site investigators and research staff through the PMB Online Agent Order Processing (OAOP) application. Access to OAOP requires the establishment of a CTEP Identity and Access Management (IAM) account and the maintenance of an "active" account status, a "current" password, and active person registration status. Questions about IB access may be directed to the PMB IB Coordinator via email to ibcoordinator@mail.nih.gov or the IB Coordinator may be contacted at 240-276-6575.

Other Names: IMFINZI™

Classification: Anti-PD-L1 MAb

Molecular Weight: ~ 149 kDa

Mode of Action: Durvalumab (MEDI4736) inhibits binding of programmed cell death ligand 1 (PD-L1) to PD-1 and CD80. In-vitro studies demonstrate that durvalumab (MEDI4736) relieves PD-L1-mediated suppression of human T-cell activation. Durvalumab (MEDI4736) does not trigger antibody-dependent cellular cytotoxicity or complement-dependent cytotoxicity in cell-based functional assays.

Description: Durvalumab (MEDI4736) is a human immunoglobulin G1 kappa (IgG1κ) monoclonal antibody.

How Supplied: Durvalumab (MEDI4736) is supplied by AstraZeneca, and distributed by the Pharmaceutical Management Branch, CTEP/DCTD/NCI. Durvalumab (MEDI4736) injection is a clear to slightly opalescent, colorless to slightly yellow solution for intravenous use. Each vial contains 500 mg of durvalumab (MEDI4736) in 10 mL of solution. Each 1 mL of solution contains 50 mg of durvalumab (MEDI4736) and is formulated in: L-histidine (2 mg), L-histidine hydrochloride monohydrate (2.7 mg), α,α-trehalose dihydrate (104 mg), polysorbate 80 (0.2 mg), and Water for Injection, USP.

Preparation: Durvalumab (MEDI4736) solution for infusion must be diluted prior to administration. To prepare the infusion solution add the dose volume of durvalumab (MEDI4736) to an infusion bag containing 0.9% Sodium Chloride Injection or Dextrose 5% in Water Injection, USP and mix by gentle inversion to ensure homogeneity of the dose in the bag. The final concentration must be between **1 mg/mL to 15 mg/mL**.

Infusion bags must be latex-free and can be made of polypropylene, polyethylene, polyolefin copolymers, or polyvinyl chloride.

Storage: Store intact vials between 2-8°C (36-46°F). Do not freeze. Protect from light by storing in the original box.

If a storage temperature excursion is identified, promptly return durvalumab (MEDI4736) to between 2-8°C and quarantine the supplies. Provide a detailed report of the excursion (including documentation of temperature monitoring and duration of the excursion) to PMBAfterHours@mail.nih.gov for determination of suitability.

Stability: Refer to the package label for expiration.

Total in-use storage time from needle puncture of durvalumab (MEDI4736) vial to start of administration should not exceed 8 hours at room temperature or 24 hours at 2-8°C (36-46°F). Prior to the start of the infusion, ensure that the bag contents are at room temperature (approximately 25°C) to avoid an infusion reaction due to the administration of the solution at low temperatures.

Route of Administration: IV infusion

Method of Administration: Infuse over approximately 60 minutes using an infusion set containing a 0.22 or 0.2 µm in-line filter. No incompatibilities between durvalumab (MEDI4736) and polyethylene, polypropylene, polyvinylchloride, or polyolefin copolymers have been observed. Flush the IV line with a volume of IV bag diluent equal to the priming volume of the infusion set used at the completion of infusion. Do not co-administer other drugs through the same infusion line.

Patient Care Implications: Refer to the protocol for information on evaluation and management of potential immune-related adverse events.

9.2 Commercial Agent: Cetuximab NSC# 714692

Sites must refer to the package insert for detailed pharmacologic and safety information.

9.2.1 Adverse Events

A list of the adverse events and potential risks associated with cetuximab administered in this study can be found in Section 7.4.

9.2.2 Availability/Supply

Cetuximab is available via commercial prescription.

9.2.3 Preparation, Storage and Stability

Refer to the current FDA-approved package insert.

9.2.4 Administration

Please see Section 5.1.2 for administration instructions. Administer through a low protein-binding 0.22 micrometer in-line filter.

10. PATHOLOGY/BIOSPECIMEN

10.1 Biospecimen Submissions (04-OCT-2019)

Note: Investigators should check with their pathology department regarding release of biospecimens before approaching patients about participation in the trial.

In this study, submission of H&E stained slides and block (or punch biopsy of paraffin block) to the Biospecimen Bank at UCSF for central review is mandatory for all patients, for the purpose of analyzing tissue for PD-L1 status. In addition, oral cavity, laryngeal, and hypopharyngeal primaries will be tested for p16 at the end of the trial. These submissions must be received at the biobank within 7-10 business days from time of patient registration. Sites must confirm with their pathology labs to make sure they can provide the required material as the bank must be able to retain these samples for the mandatory testing.

Note: Fine needle aspirates (FNA) samples are not acceptable since they do not provide enough material for PD-L1 and p16 testing. A cell block derived from the FNA is allowable if there are sufficient cells present in the block for PD-L1 testing. Dr Jordan will determine this upon receipt. For sites submitting FNA cellblocks for ALL patients, they must do so within 7-10 business days from registering the patient. Sites must confirm with their cytology/pathology labs to make sure they can provide the required material as the bank must be able to retain these samples for the mandatory testing.

Sites can obtain a new biopsy to get sufficient tissue if needed (***prior to Step 1 registration***). An additional biopsy is NOT required, but if there is not enough tissue to verify p16 status from the standard of care pre-registration biopsy, patients with oropharyngeal and unknown primaries will be ineligible for the study and will not receive study treatment (see details below).

For oropharyngeal and unknown primaries, submission of H&E and p16 stained slides (with the required block for PD-L1) to the Biospecimen Bank at UCSF is required ***prior to Step 2 registration***.

It is also highly recommended (but optional for the patient) that serum and peripheral blood specimens be

submitted for banking for future translational research.

10.2 Pre-hoc Central Review of Oropharyngeal and Unknown Primaries (04-OCT-2019)

Pre-hoc central review mandatory for oropharyngeal and unknown primaries to confirm p16 status prior to Step 2 registration. Institutions must test for p16 status by immunohistochemistry (IHC) using a Clinical Laboratory Improvement Amendments (CLIA)-certified laboratory. Note: A rigorous laboratory accreditation process similar to the U.S. CLIA certification, such as the provincial accreditation status offered by the Ontario Laboratory Accreditation (OLA) Program in Canada, the College of American Pathologists (CAP), or an equivalent accreditation in other countries, is acceptable.

Pre-hoc central review will be conducted with rapid turnaround (1-2 business days from receipt of the slides) coordinated by Dr. Richard Jordan at the NRG Oncology Biospecimen Bank–San Francisco. Specifics of type and source of p16 antibody and testing method will be requested (although not required) at the time of specimen submission. Every effort should be made to obtain and submit this information at the time of specimen transmission, in order to ensure the fastest possible resolution should there be any questions about staining technique.

H&E stained slides will be submitted for oropharyngeal and unknown primaries used to confirm presence of tumor in the sample and to aid in assay interpretation. These slides must be submitted with blocks. The primary reviewer will be the Pathology Co-Chair, Richard Jordan, DDS, PhD. Interpretation of each p16 immunostained slide will be performed using the H-score method described by Jordan et al. (2012) that has been validated as a reliable, reproducible, and accurate method to score p16 in squamous cell carcinoma of the head and neck.

The p16 IHC will be scored as evaluable if strong and diffuse positivity was observed in the tissue mounted on each slide. The highest intensity of p16 staining present in the tumor will be scored on an ordinal score of 0-3, relative to the intensity of the positive (score 3) and negative (score 0). The percent of tumor staining at the highest intensity also will be estimated within 5% increments. The H score is derived from the cross product of the intensity score (0 to 3), and the percent of tumor staining at the highest intensity (0-100%). An optimal H-score cut-point of 60 on a scale of 0-300 yields an average sensitivity of 91.6% and specificity of 90.4% for HR-HPV oncogene expression and thus an H-score cut-point of 60 indicates that a tumor with diffuse low-intensity nuclear and cytoplasmic p16 staining in the majority of the tumor is a true positive (Jordan 2012). High agreement on inter-rater interpretation has been reported (Schlecht 2011, Thavaraj 2011), indicating the familiarity of pathologists with interpretation of IHC assays. Similarly, comparable assay performance for p16 to that observed here has also been previously reported (Schlecht 2011, Thavaraj 2011). The most common p16 monoclonal antibody in use is E6H4 (CINtec). Other acceptable p16 antibody types include 16P04 and JC8. If a different p16 antibody is used, discussion with the Pathology Co-Chairs is strongly encouraged.

**Mandatory: Specimen Collection for Central p16 Confirmation of Oropharyngeal and Unknown Primary Tumors**

- H&E and p16 stained slides must be submitted for oropharyngeal and unknown primary tumors.
- For patients with oropharyngeal or unknown primary cases, rapid central review of p16 slides will be performed within 1-2 business days from receipt of samples and paperwork. H&E slide will be used to confirm presence of tumor.
- P16 staining will be performed at UCSF for oral cavity, laryngeal, and hypopharyngeal primaries cases at the end of the trial.
- Required Forms: ST form and pathology reports with study and case number date of procedure, pathology accession number and p16 staining result; any other personal health information (PHI) should be redacted by sites before sending.
- Shipping costs: Submitting site pays cost of shipping FFPE samples and returns.
- Residual Material: p16 slides will be retained unless return is requested by the submitting site. H&E slides and blocks will be retained at the bank.

Ship all biospecimens for central review for this trial by courier (Fed Ex/UPS) to:
NRG Oncology Biospecimen Bank–San Francisco
University of California San Francisco
2340 Sutter Street, Room S341
San Francisco, CA 94115
415-476-7864; FAX 415-476-5271
NRGBB@ucsf.edu

Specimen Type	Collection Time Points	Collection Information and Requirements	Shipping
For oropharyngeal and unknown primary tumors:<br><br>-One H&E slide<br>-One p16 stained IHC slide from primary tumor<br><br>H&E slides can be duplicate cut slides, they do not have to be the diagnostic slide	Pre-Treatment	H&E and p16 slide must be made from the same pathology block. Enrolling institution, using a CLIA certified laboratory, screens patient with p16 testing by IHC. Then Biospecimen Bank does central review prior to randomization.	Slides shipped ambient to NRG Biospecimen Bank San Francisco

10.3 Tissue Blocks for Central Review of PD-L1 and p16 status (13-JAN-2021)
All tissue specimens will be analyzed for PD-L1 status at the end of the trial. Tissue specimens for oral cavity, laryngeal, and hypopharyngeal primaries will also be analyzed for p16 status at the end of the trial.

PD-L1 testing will be performed at QualTek Molecular Laboratories Clinical Laboratories (215-504-7402) at the end of the trial. QualTek has two fully equipped Laboratory locations in Santa Barbara, CA (CAP/CLIA accredited) and Newtown, PA (GLP capable). We will use the Ventana SP263 rabbit polyclonal antibody, with a positive result considered as >25% expressing tumor cells of any intensity.

Tumor p16 expression will be evaluated by immunohistochemical analysis with a mouse monoclonal antibody (MTM Laboratories) visualized with use of an autostainer (Ventana XT, Ventana) and a one-view secondary detection kit (Ventana). Positive p16 expression will be defined as strong and diffuse nuclear and cytoplasmic staining in 70% or more of the tumor cells (Ang 2010).

Mandatory: Specimen Collection for PD-L1 and p16 Expression

• Submission of H&E stained slides and block (or punch biopsy of paraffin block) to the Biospecimen Bank at UCSF for central review is mandatory for all patients.<br>• For oropharyngeal or unknown primary patients, the block must be submitted at the same time as the H&E and p16 slide.<br>• For all other patients, the slides and block must be submitted within 14 days from date patient was enrolled on study.<br>• Required Forms: ST form and pathology reports with date of procedure, pathology accession number; any other personal health information (PHI) should be redacted by sites before sending.<br>• FFPE Punch Kits should be requested from the Biospecimen Bank when requesting Frozen specimen kits.<br>• Shipping costs: Submitting site pays cost of shipping FFPE samples and returns.<br>• PD-L1 testing will be performed on all cases when study closes to accrual by QualTek Molecular Laboratories Clinical Laboratories<br>Results: QualTek Molecular Laboratories Clinical Laboratories will report to NRG Oncology when study closes to accrual.<br>• Residual Material: FFPE specimens will be retained at the Biospecimen Bank unless needed for patient continuing care. One to two 3-mm punches will be taken from the blocks before shipping to sites that have requested the return of their blocks.<br><br>**Ship all biospecimens to**:<br>NRG Oncology Biospecimen Bank–San Francisco<br>University of California San Francisco<br>2340 Sutter Street, Room S341<br>San Francisco, CA 94115<br>415-476-7864; FAX 415-476-5271<br>NRGBB@ucsf.edu

Specimen Type	Collection Time Points	Collection Information and Requirements	Shipping
One H&E slide from primary tumor; H&E slides can be duplicate cut slides, they do not have to be the diagnostic slide	Baseline tumor specimen	H&E stained slide; can be the same as submitted for central review or additional slides can be submitted	Slides shipped ambient to NRG Biospecimen Bank San Francisco
FFPE Block or one to two 3-mm punches taken from the tumor block (embedded*)	Pre-treatment	Corresponding FFPE Block or one to two 3-mm punches from the same block as the H&E slide that is being submitted (embedded*)	Shipped ambient or with cold pack to the NRG Biospecimen Bank San Francisco

*For sites with the capability to do so, the one to two punch biopsies should be embedded into one new paraffin block from which an H&E slide should be obtained. The constructed block containing the punch and the new H&E slide must then be submitted to the NRG Oncology Biospecimen Bank in San Francisco. Alternatively, sites can either 1) send the block to the Biospecimen Bank, and the bank will punch and embed the blocks for the sites before returning them, or 2) send the one to two punches to the bank to be embedded by the Biospecimen Bank.

10.4 **Optional Specimen Submissions** (13-JAN-2021)

Patients must be offered the opportunity to consent to optional specimen collection. If the patient consents to participate, the site is required to submit the patient's specimens as specified per protocol. Sites are not permitted to delete the specimen component from the protocol or from the sample consent.

**Specimen Collection for Biobanking for Potential Future Research**

(to be offered to all patients)

Forms: ST Form – filled out completely with study, case, date of procedure, institution and time points.

Kits: can be requested from the NRGBB-SF at NRGBB@ucsf.edu. Allow 5-10 business days for kits. Sites must have IRB approval before requesting kits.

Shipping: One prepaid return label provided for each case for batch shipping frozen biospecimens only.

Batch ship frozen serum samples Monday-Wednesday (US sites) and Monday-Tuesday (Canadian Sites).

Processing Instructions: Located on the CTSU website protocol specific documents.
NOTE: If sites missed collecting serum samples at baseline, they should not collect serum at future time points.

Ship all serum specimens by overnight courier to:
NRG Oncology Biospecimen Bank – San Francisco
UCSF – Dept of Radiation Oncology
2340 Sutter Street- Room S341
San Francisco, CA 94115

For questions, please contact the San Francisco Bank at:
Email: NRGBB@ucsf.edu
415-476-7864/Fax 415-476-5271

Specimen Type	Collection Time Points	Collection Information and Requirements/Instructions for Site	Shipping
Serum: One 10 mL red top (clot) tube	● Baseline (within 14 days prior to starting systemic therapy)<br>● Within 7 days prior to starting RT (after starting systemic therapy)<br>● 1 month after end of RT (± 7 days)<br>● 1 month (± 14 days) after last dose of systemic therapy (durvalumab arm) OR 4 months (± 14 days) after RT (cetuximab arm)	Serum centrifuged and aliquotted.<br><br>Frozen serum samples containing a minimum of 0.5 mL per aliquot in 1 mL cryovials (up to 5 per tube drawn).<br><br>Samples should be frozen and stored at -80ºC until ready to batch ship.<br><br>If sites missed collecting serum samples at baseline, they should not collect serum at future time points.	Serum sent frozen on dry ice via overnight carrier to the NRG Biospecimen Bank- San Francisco<br><br>Batch shipping of multiple cases/time points in one shipment is encouraged.

Tumor tissue (for patients who consent to optional biobanking)
Residual tissue from mandatory pre-treatment tissue studies will be retained at the NRG Oncology Biospecimen Bank–San Francisco, if the patient consents to optional biobanking.

Analysis of Peripheral Blood Specimens (to be offered to Phase II/III patients only)

Peripheral Blood Specimens
Peripheral blood specimens will be collected at baseline and longitudinally. Specimens will be banked and analyzed at the Moores Cancer Center (MCC) biorepository at University of California San Diego.

NOTE: If sites missed collecting peripheral blood samples at baseline, they should not collect peripheral blood samples at future time points. For baseline samples collected but determined non-viable by the MCC lab, sites will be notified to not collect future time points.

After adequate samples for all time points have been collected for 100 patients, we will contact sites to stop sample collection.

Required Form: Study Specific Specimen Transmittal (ST) form
Biospecimen Kits: Can be requested from the NRG Biobank at NRGBB@ucsf.edu. Allow 5-10 business days for kits. Sites must have IRB approval before requesting kits.
Shipping days: Monday- Wednesday (U.S. sites).

Shipping labels will be provided with kits for peripheral blood.

Ship specimens for this peripheral blood specimen study to:
Attn.: Sharmeela Kaushal
Assistant Director, MCC Biorepository
UCSD Moores Cancer Center Biorepository
3855 Health Sciences Dr, Room 3331 OR Room 3345-G
La Jolla, CA 92093-0819
Email: skaushal@ucsd.edu
Phone: 858-534-7302
Fax: 858-822-5380

NOTE: Samples MUST be overnighted within 12 hours of collection.

NOTE: Do NOT collect and/or ship samples on Friday, Saturday, or Sunday.

NOTE: The coordinator or personnel collecting the sample MUST send a notification by email to Sharmeela Kaushal (skaushal@ucsd.edu) and Mason Kyle (mkyle@ucsd.edu) in advance and provide the tracking information.

NOTE: Do **NOT** ship peripheral blood to NRG Oncology Biospecimen Bank.

For additional questions, contact:
Dr. Mell: 858-246-2174 (lmell@ucsd.edu) or Alfredo Molinolo (amolinolo@ucsd.edu)

**Specimen Type**	**Collection Time Points**	**Collection Information and Requirements**	**Shipping**
Draw blood in five (5) 10 ml green top (heparin) tubes. Collect at least 8 ml per tube.	● Baseline (within 14 days prior to starting systemic therapy)<br>● Within 7 days prior to starting RT (after starting systemic therapy)<br>● 1 month after end of RT (± 7 days)<br>● 1 month (± 14 days) after last dose of systemic therapy	See Appendix II for processing and shipping information.<br><br>If sites missed collecting peripheral blood at baseline, they should not collect future time points.	Ship fresh at room temperature overnight for morning delivery to the UCSD MCC Biorepository

	(durvalumab arm) OR 4 months (± 14 days) after RT (cetuximab arm)		
Peripheral blood (for patients who consent to optional biobanking)<br>Residual blood or derivatives from optional studies will be shipped to the NRG Oncology Biospecimen Bank–San Francisco from the UCSD MCC Biorepository for biobanking, if the patient consents to optional biobanking. Based on the usability of the specimen and at the discretion of the NRG Biospecimen Bank Director (Dr. Richard Jordan) these will be retained or destroyed.			

11. SPECIAL STUDIES (NON-TISSUE)

All participating centers will be required to participate in the quality of life and patient reported outcome assessments.

11.1 Quality of Life Instruments Description (15-JAN-2019)

See Section 4 for time points.

EORTC QLQ-C30 / EORTC QLQ-H&N35

The European Organization for Research and Treatment of Cancer Core Questionnaire (EORTC QLQ-C30 Version 3.0) and the Head-and-Neck module (EORTC QLQ-H&N35 Version 1.0) (Aaronson NK, 2012, Sherman AC, 2000)

The EORTC QLQ-C30 / EORTC QLQ-H&N35 (Version 1) consists of a 30-item self-reporting questionnaire developed to assess overall QOL of patients with cancer and a 35-item head and neck cancer specific module. The EORTC QLQ-C30 is grouped into five functional subscales (role, physical, cognitive, emotional and social functioning), three multi-item symptom scales (fatigue, pain, and nausea and vomiting), individual questions concerning common symptoms in cancer patients, and two questions assessing overall QOL. This copyrighted instrument is validated and can be found on the NRG-HN004 protocol page of the CTSU website. The EORTC QLQ-H&N35 module is specific for multi-modality HNC therapy and contains an additional 35 questions. Both of the scales and single-item measures range in score from 0 to 100; a high scale score represents a higher response/functioning level (better QOL). The time for administration is about 7-10 minutes for each instrument. While social and emotional well-being are important to QOL, they are not as likely to change as quickly or dramatically over time or in response to therapy. Furthermore, in a pooled analysis of RTOG 90-03 and RTOG 91-11, the emotional well-being subscale was not shown to be predictive of survival in cancer patients (Coyne et al., 2007). Therefore we will evaluate the individual subscales of the EORTC QLQ, in particular the role and physical functioning between arms. The minimum important difference (MID), which is defined as the smallest difference in score that a patient perceives as important, that may lead to a change in the patient's management (King, 2011). The clinically meaningful MID of a QOL instrument is often estimated as being at least 5-10% of the instrument range. Cella et al. found that clinical meaningful group score changes in global QOL are often small for QOL improvements from baseline, whereas worsening global QOL is often associated with much larger negative score changes (Cella 2002).

NRG Oncology has obtained permission to use the EORTC QLQ-C30 and H&N35 for this study in English, Spanish, and French. If a site would like access to another language, please contact NRG Oncology to discuss.

MD Anderson Dysphagia Inventory (MDADI)

Swallowing will be evaluated using the validated PRO swallowing instrument is the MD Anderson Dysphagia Inventory (MDADI), a 20-item self-administered patient reported dysphagia-specific instrument consisting of global, emotional, functional, and physical subscales (Chen 2001), and is currently being used on several prospective multicenter randomized NRG oncology clinical trials. Hutcheson et al. evaluated the MD Anderson Dysphagia Inventory (MDADI) questionnaire (scored out of 100) for swallowing-related QOL in 1,136 patients with HNC. Using anchor based methods, a ten-point difference between groups in composite MDADI scores was identified as clinically meaningful (Hutcheson 2016). The difference in the mean MD Anderson Dysphagia Inventory (MDADI) composite score, measured at one year after radiation between arms will be compared. In this study, because it is expected that patients will be older or have more medical comorbidities with contraindication to

cisplatin, and include laryngeal and hypopharyngeal patients, swallowing QOL may be worse in this patient cohort compared patients participating in other ongoing NRG oncology studies. The effects of durvalumab immunotherapy combined with IMRT in HNSCC on swallowing-related QOL are unknown. It will be important to evaluate long term swallowing in this patient cohort particularly if the functional morbidity of dysphagia after multimodality treatment potentially contributes to non-cancer deaths secondary to complications from malnutrition and aspiration, which could potentially offset any survival gains from combined modality therapy (Cooper 2012; Forastiere 2013).

NRG Oncology has obtained permission to use the MDADI for this study in English, Spanish, and French. If a site would like access to another language, please contact NRG Oncology to discuss.

<u>Charlson Comorbidity Index (CCI) and Demographic and Health Status Markers</u>
Multiple studies have shown that in addition to classic prognostic markers such as age, race, performance status, and smoking and alcohol use, many demographic and health status markers, including body mass index, socioeconomic status (and proxy instruments, such as distance traveled), marital status, and comorbidity, have additive prognostic value and explain a considerable proportion of the variation in outcomes for head/neck cancer patients (Mell 2010; Rose 2011). In addition, it is known that models accounting for such factors outperform standard prognostic methods (Carmona 2016) and that failing to account for them can bias or confound results of phase III trials (Zakeri 2013; Mell 2014). The current study affords a unique opportunity to gather detailed baseline and demographic health variables in a clinical trial population to compare and contrast with results from population studies.

In this study, research associates will gather information on Charlson Comorbidity Index (Charlson 1987), body mass index ((mass in kg) / (height in m^2)), patient and treatment center zip code, and marital status. These are not PRO instruments, and the data will be collected only at baseline by extracting information from the patient's history and/or chart. These are simple metrics to collect, posing no significant additional burden to the patient or study team. The metrics are not used to determine eligibility and will be used only to characterize the sample and assess comparability between the treatment arms.

<u>Geriatric-8 Questionnaire (G-8)</u>
Routine serial implementation of the comprehensive geriatric assessment (CGA) is challenging due the comprehensive length of the tool and resources required to complete the full assessment. Hence a screening tool to evaluate QOL and patient vulnerability (defined as impairment of 2 or more domains within the CGA) will be evaluated using the G-8. The G-8 tool has been validated in the elderly vulnerable population (Liuu 2014). The G-8 is a seven-item clinician-administered questionnaire which includes, age, Mini Nutritional Assessment (MNA), Activities Daily Living (ADL), Instrumental ADL, Mini-Mental State Exam, Geriatric Depression Scale, Cumulative Illness Rating Scale-Geriatrics, and Timed Get Up and Go. When considering the presence of at least one questionnaire with an impaired score as an abnormal reference exam, the prevalence of being at risk varied from 60% to 94% per the various definitions of the reference test. When considering the primary reference test, a cut-off value of 14 for the G-8 tool provided a good sensitivity estimate (85%) without deteriorating the specificity excessively (65%)(Bellera et al., 2012). In the head and neck cancer population, the G-8 has been compared to other screening tools including the Vulnerable Elders Survey-13 (VES-13), in combination and correlated with the European Organisation for Research and Treatment of Cancer Quality of Life Questionnaires (EORTC QLQ)-C30 and -H&N35 in 100 HNC patients, aged ≥65 years, undergoing curative radio(chemo)therapy. Pretreatment, the G-8 defined patients as vulnerable in 69.0% compared to 71.3% using the CGA, mainly due to the presence of severe grade co-morbidities, difficulties in community functioning and nutritional problems. At week 4 of radiation, significantly more patients were identified as vulnerable due to nutritional, functional and emotional deterioration. Vulnerable patients reported lower function and higher symptom burden on HR-QOL scores as compared with "fit" patients. A comparable deterioration in HR-QOL was observed in vulnerable and fit patients during treatment. This study demonstrated that the G-8 was the screening tool of choice (Pottel 2014) and superior to the VES-13 in identifying vulnerable patients. G-8 has also shown to be indicative of quality-adjusted survival in older HNC patients (Pottel 2015).
<u>Quality-Adjusted Survival, EuroQol (EQ-5D-5L)</u>
The EQ-5D™ is a trademark of the EuroQol Group, it is a well-accepted instrument to measure general QOL and cost-utility analysis (EuroQol Group 1990) and will be used to assess quality-adjusted survival for this study. It is a 2-part questionnaire that the patient can complete in approximately 5 minutes and has been translated into multiple languages. The first part consists of 5 items covering 5 dimensions, including mobility, self-care, usual activities, pain/discomfort, and anxiety/depression. Each dimension is graded on 5 levels: 1-no problems, 2-slight problems, 3-

moderate problems, 4-severe problems, and 5-unable to perform/extreme problems. There are 243 potential health states. The second part is a visual analogue scale (VAS) valuing current health state, measured on a 20 cm, 10 point-interval scale. Either the index score or the VAS score can be used in the quality-adjusted survival analysis. The benefit of measuring quality-adjusted survival is that the product, quality-adjusted survival, can be compared to the outcomes of other interventions across disease sites and can be used by health policy makers to rank interventions. The EQ5D-5L will be used to evaluate the effect of adding MEDI4736 to chemoradiation on quality-adjusted survival. The EQ-5D-5L is available in over 125 languages.

NRG Oncology has obtained permission to use the EQ-5D-5L for this study in English, Spanish, and French. If a site would like access to another language, please contact NRG Oncology to discuss.

Performance Status Scale for Head and Neck Cancer (PSS-HN)
The Performance Status Scale for Head and Neck Cancer (PSS-HN) and Swallowing Function
The PSS-HN will be a secondary tool to assess swallowing function in addition to physician assessment of nutrition and PEG tube status and has been used on multiple RTOG head and neck trials. The PSS-HN is a clinician-rated instrument consisting of 3 subscales: normalcy of diet, public eating, and understandability of speech. The PSS-HN has been psychometrically validated (List 1997; List 1999) and recommended by the NCCN for measurement of swallowing and speech performance in patients with head and neck cancer undergoing primary radiotherapy based treatment. PSS-HN scores are an important evaluation of normalcy of diet and ability to eat in public. In RTOG 0522, it was found that 38% and 26% of patients at 12 months had clinically worsened normalcy of diet and ability to eat in public, respectively, after chemoradiation using the PSS-HN. Furthermore, baseline PSS-HN- diet and eating scores in the intermediate and high-risk OPC and non-OPC patients were lower compared to the low-risk OPC patient population. In RTOG 0522, longitudinal swallowing function demonstrated acute deterioration in scores for PSS-HN-diet and eating in both treatment arms in the last 2 weeks of treatment to 3 months in all patients receiving CRT. However, p16-negative OPC patients demonstrate a slower recovery compared to p16-positive OPC patients (Truong 2017). It is hypothesized that MEDI4736 (durvalumab) will have less decline in long-term swallowing function with respect to normalcy of diet and ability to eat in public and swallowing-related QOL as measured by the EORTC H&N35 subscale items pertaining to eating and swallowing at 12 months from the end of RT compared to the control arm.

PSS-HN is not a PRO; hence, investigators or research associates can complete it quickly at the scheduled visits without adding to patient burden.

11.1.1 Administration of NRG-HN004 Patient-Completed Questionnaires

Time points for administration are located in Section 4.

11.1.2 Administration Instructions

For patient opting out of ePRO, the PRO Forms should be administered during an office visit if at all possible, preferably while the patient is waiting to be seen. Once the questionnaires are completed by the patient, the staff member should review it to ensure that no items were unintentionally left blank. When absolutely necessary, it may also be administered by mail or phone. The completed forms will be data entered in Medidata Rave.
Patients who never initiate NRG-HN004 study therapy or who experience disease progression should continue participating in the PRO study. If a patient does not come in to clinic, the questionnaires will either be mailed to the patient or the research assistant will call the patient to complete the forms. If the patient does not return the forms within two weeks the patient will be called and either another set will be sent or the patient will complete the questionnaires over the phone with the research assistant.

If a patient declines to complete a scheduled PRO forms or if the questionnaire is not completed for any other reason (and cannot be completed by phone or mail), the QOL coversheet must be completed in Rave. For patients who agree to use ePRO for PRO collection, please refer to Appendix III.

12. MODALITY REVIEWS (09-MAR-2022)

12.1 Radiation Therapy Quality Assurance Reviews

Cases for the RT Quality Assurance Reviews will be selected using a sampling scheme. The Principal

Investigator, Dr. Loren Mell, or NRG Oncology Headquarters approved designee will perform an RT Quality Assurance Review for all patients included in the sample who receive radiation therapy. Reviews will be performed after NRG Headquarters has received complete data for each case enrolled. These reviews will be ongoing and will be facilitated by IROC Philadelphia RT. The goal of the review is to evaluate protocol compliance. The review process is contingent on timely submission of radiotherapy treatment data.

The scoring mechanism is: **Per Protocol, Variation Acceptable, Deviation Unacceptable, and Not Evaluable**.

12.2 Medical Oncology Modality Quality Assurance Reviews (15-JAN-2019)

The Medical Oncology Co-Chair, Dr. Stuart Wong, or NRG Oncology Headquarters approved designee will perform a Systemic Therapy Assurance Review of all patients who receive or are to receive systemic therapy in this trial. The goal of the review is to evaluate protocol compliance. The review process is contingent on timely submission of systemic therapy data as specified in Section 12.1. The scoring mechanism is: **1) Per Protocol, 2) Acceptable Variation, 3) Unacceptable Deviation, and 4) Not Evaluable**.

Dr. Wong/designee will perform a Quality Assurance Review after NRG Oncology Headquarters has received complete data for each case. The reviews will be ongoing.

13. DATA AND RECORDS

13.1 Data Management/Collection (09-MAR-2022)

Medidata Rave is a clinical data management system being used for data collection for this trial/study. Access to the trial in Rave is controlled through the CTEP-IAM system and role assignments.

Requirements to access Rave via iMedidata:

- A valid CTEP-IAM account; and
- Assigned a Rave role on the LPO or PO roster at the enrolling site of: Rave CRA, Rave Read Only, Rave CRA (LabAdmin), Rave SLA, or Rave Investigator.

Rave role requirements:

- Rave CRA or Rave CRA (Lab Admin) role must have a minimum of an Associate Plus (AP) registration type;
- Rave Investigator role must be registered as an Non-Physician Investigator (NPIVR) or Investigator (IVR); and
- Rave Read Only role must have at a minimum an Associates (A) registration type.

Refer to https://ctep.cancer.gov/investigatorResources/default.htm for registration types and documentation required.

This study has a Delegation of Tasks Log (DTL). Therefore, those requiring write access to Rave must also be assigned the appropriate Rave tasks on the DTL.

Upon initial site registration approval for the study in Regulatory Support System (RSS), all persons with Rave roles assigned on the appropriate roster will be sent a study invitation email from iMedidata.

To accept the invitation, site users must either click on the link in the email or log in to iMedidata via the CTSU members' website under *Data Management* > *Rave Home* and click to *accept* the invitation in the *Tasks* pane located in the upper right corner of the iMedidata screen. Site staff will not be able to access the study in Rave until all required Medidata and study specific trainings are completed. Trainings will be in the form of electronic learnings (eLearnings) and can be accessed by clicking on the eLearning link in the *Tasks* pane located in the upper right corner of the iMedidata screen. If an eLearning is required for a study and has not yet been taken, the link to the eLearning will appear under the study name in the *Studies* pane located in the center of the iMedidata screen; once the successful completion of the eLearning has been recorded, access to the study in Rave will be granted, and a Rave EDC link will replace the eLearning link under the study name.

Site staff that have not previously activated their iMedidata/Rave account at the time of initial site registration approval for the study in RSS will receive a separate invitation from iMedidata to activate their account. Account activation instructions are located on the CTSU website in the Data Management section

under the Rave resource materials (Medidata Account Activation and Study Invitation Acceptance). Additional information on iMedidata/Rave is available on the CTSU members' website in the Data Management > Rave section at www.ctsu.org/RAVE/ or by contacting the CTSU Help Desk at 1-888-823-5923 or by email at ctsucontact@westat.com.

13.2 **Data Quality Portal** (09-MAR-2022)

The Data Quality Portal (DQP) provides a central location for site staff to manage unanswered queries and form delinquencies, monitor data quality and timeliness, generate reports, and review metrics.

The DQP is located on the CTSU members' website under Data Management. The Rave Home section displays a table providing summary counts of Total Delinquencies and Total Queries. DQP Queries, DQP Delinquent Forms, DQP Form Status, and the DQP Reports modules are available to access details and reports of unanswered queries, delinquent forms, forms with current status, and timeliness reports. Review the DQP modules on a regular basis to manage specified queries and delinquent forms.

The DQP is accessible by site staff that are rostered to a site and have access to the CTSU website. Staff that have Rave study access can access the Rave study data using a direct link on the DQP.

To learn more about DQP use and access, click on the Help icon displayed on the Rave Home, DQP Queries, DQP Delinquent Forms, DQP Form Status, and DQP Reports modules.

13.3 **Rave-CTEP-AERS integration** (09-MAR-2022)

The Rave Cancer Therapy Evaluation Program Adverse Event Reporting System (CTEP-AERS) Integration enables evaluation of Adverse Events (AE) entered in Rave to determine whether they require expedited reporting and facilitates entry in CTEP-AERS for those AEs requiring expedited reporting. **Sites must initiate all AEs for this study in Medidata Rave.**

Pre-treatment AEs: Solicited baseline adverse events that occur prior to start of treatment are collected in Medidata Rave on the Protocol Specific Adverse Event Baseline form.

Treatment-emergent AEs: All AEs that occur after start of treatment are collected in Medidata Rave using the Adverse Event form, which is available for entry at each treatment course or reporting period and is used to collect AEs that start during the period or persist from the previous reporting period. AEs that occur 30 Days after the Last Administration of the Investigational Agent/Intervention are collected using the Late Adverse Event form.

Prior to sending AEs through the rules evaluation process, site staff should verify the following on the Adverse Event form in Rave:

- The reporting period (course/cycle) is correct; and
- AEs are recorded and complete (no missing fields) and the form is query free.

The CRA reports AEs in Rave at the time the Investigator learns of the event. If the CRA modifies an AE, it must be re-submitted for rules evaluation.

Upon completion of AE entry in Medidata Rave, the CRA submits the AE for rules evaluation by completing the Expedited Reporting Evaluation form. Both NCI and protocol-specific reporting rules evaluate the AEs submitted for expedited reporting. A report is initiated in CTEP-AERS using information entered in Medidata Rave for AEs that meet reporting requirements. The CRA completes the report by accessing CTEP-AERS via a direct link on the Medidata Rave Expedited Reporting Evaluation form. Contact the CTSU Help Desk at 1-888-823-5923 or by email at ctsucontact@westat.com if you have any issues submitting an expedited report in CTEP-AERS.

In the rare occurrence that internet connectivity is lost, a 24-hour notification is to be made to

CTEP by telephone at 301-897-7497. Once internet connectivity is restored, the 24-hour notification that was phoned in must be entered immediately into CTEP-AERS using the direct link from Medidata Rave.

Additional information about the CTEP-AERS integration is available on the CTSU members' website:

- Study specific documents: *Protocols* > *Documents*> *Protocol Related Documents*> *Adverse Event Reporting*; and
- Additional resources: *Resources* > *CTSU Operations Information*> *User Guides & Help Topics*.

NCI requirements for SAE reporting are available on the CTEP website:

- NCI Guidelines for Investigators: Adverse Event Reporting Requirements is available at **https://ctep.cancer.gov/protocolDevelopment/electronic_applications/docs/aeguidelines.pdf**

13.4 Summary of Data Submission (21-AUG-2019)

Adverse event data collection and reporting, which are required as part of every clinical trial, are done to ensure the safety of patients enrolled in the studies as well as those who will enroll in future studies using similar agents. Adverse events are reported in a routine manner at scheduled times during the trial using Medidata Rave®. Additionally, certain adverse events must be reported in an expedited manner for more timely monitoring of patient safety and care. See Section 7 for information about expedited and routine reporting. PRO-CTCAE is not intended for expedited reporting, real time review, or safety reporting. PRO-CTCAE data are exploratory and not currently intended for use in data safety monitoring or adverse event stopping rules.

Summary of Data Submission: Refer to the CTSU website.

Summary of Dosimetry Digital Data Submission (Submit to TRIAD; see Section 8.3.1 for account access and installation instructions.)

<table>
<tr><td rowspan="5">DICOM DIGITAL DATA</td><td>DICOM CT IMAGE SET</td><td rowspan="8">TRIAD submission time point =

RT DIGITAL PLAN

Due within 1 week of the start of RT</td></tr>
<tr><td>DICOM RT STRUCTURE</td></tr>
<tr><td>DICOM RT DOSE</td></tr>
<tr><td>DICOM RT PLAN</td></tr>
<tr><td>*DICOM PET (Required when Applicable)
*DICOM PET/CT (Required when Applicable)
*DICOM MRI (Required when Applicable)</td></tr>
<tr><td colspan="2">*All image data sets used for structure delineation must be submitted with RT data (Section 5.2.3).</td></tr>
<tr><td colspan="2">All required structures MUST be labeled per the tables in Sections 5.2.4 and 5.2.5.</td></tr>
<tr><td colspan="2">Upon submission of the Digital Data via TRIAD, complete an online Digital Data Submission Information Form (DDSI)
https://www.irocqa.org/Resources/TRIAD-for-RT-QA</td></tr>
</table>

NOTE: ALL SIMULATION AND PORTAL FILMS AND OR DIGITAL FILM IMAGES WILL BE KEPT BY THE INSTITUTION AND ONLY SUBMITTED IF REQUESTED.

13.5 Global Reporting/Monitoring

This study will be monitored by the Clinical Data Update System (CDUS) Version 3.0. Cumulative protocol- and patient-specific CDUS data will be submitted electronically to CTEP on a quarterly basis by FTP burst of data. Reports are due January 31, April 30, July 31, and October 31. Instructions for submitting data using the CDUS can be found on the CTEP Web site (http://ctep.cancer.gov/reporting/cdus.html).

14. STATISTICAL CONSIDERATIONS

14.1 Study Design (15-JAN-2019)

Justification of Design

This is a randomized phase II/III trial of radiotherapy with concurrent MEDI4736 (durvalumab) vs. radiotherapy with concurrent cetuximab in patients with stage locoregionally advanced head and neck cancer with a contradiction to cisplatin. There will be a safety lead in to evaluate the dose limiting toxicities for radiotherapy with concurrent MEDI4736 (durvalumab). If proved to be safe, patients will be randomized between the two arms for a comparison of PFS in the phase II component, these patients will be included in the phase III final analysis if there is a statistically significant difference in PFS in the phase II.

Randomization

The stratified permuted block randomization treatment allocation scheme described by Zelen (1974) will be used as it balances patient factors other than the treating institution. The randomization ratio between the two arms will be 2 (RT + MEDI4736):1 (RT + cetuximab).

Stratification

Patients will be stratified by the following factors:

- Stage (T0-3 and N0-2 vs. T4 and/or N3)
- Performance status/comorbidity (PS=0 and modified CCI=0 vs. PS=1-2 and/or modified CCI>0)
- Primary site (p16+ OPX/CUP vs. Other [Larynx, HPX, Oral Cavity, or p16- OPX/CUP])

Total Accrual

Lead-In: 8-30 evaluable patients (depending on required dose levels)
Phase II: 234 randomized patients (projecting 260 enrolled to reach 234 randomized)
Phase III: 444 randomized patients, including the 234 randomized in Phase II (projecting 493 enrolled to reach 444 randomized)

14.2 Study Endpoints (15-JAN-2019)

14.2.1 Primary Endpoints

Lead-In: Dose-limiting toxicity (DLT)
Phase II: Progression-free survival (PFS)
Phase III: Overall survival (OS)

14.2.3 Secondary Endpoints

- Locoregional failure (LRF)
- Distant metastasis (DM)
- Competing mortality
- Response on 4-month FDG-PET/CT, measured by RECIST
- Adverse events measured by CTCAE and PRO-CTCAE
- QOL endpoints including functional domain of the EORTC QLQ and swallowing QOL using total composite MDADI score at 1 year from end of RT change from baseline
- Performance status per PSS-HN
- Translational research, including PD-L1 and p16

14.2.4 Exploratory Endpoints

- Secondary biomarkers
- PRO-CTCAE
- QOL endpoints using other items in EORTC QLQ/HN35, EQ5D and MDADI subscales in the short term (end of RT to 8 months) and long term (12-24 months from end of RT)

14.3 Primary Objectives Study Design (09-MAR-2022)

14.3.1 Primary Hypotheses and Endpoint Definitions

Lead-In

It is hypothesized that MEDI4736 (durvalumab) can be safely delivered, per the DLT definition, when given concurrently with radiation for patients with locoregionally advanced HNC who have a contraindication to cisplatin.

DLT for radiotherapy with concurrent MEDI4736 (durvalumab), as defined below (please see section 5.1 for dose level guidelines).

- DLT Observation Period: The DLT observation period will start at the first dose of MEDI4736 (durvalumab) and extend for 4 weeks after the completion of radiation therapy.

- DLT Definition: DLT is defined as the occurrence of an adverse event (AE) listed below that is definitely or probably related to MEDI4736 (durvalumab) irrespective of relationship to radiation therapy and occurs during the specified observation window. AEs will be graded according to NCI CTCAE version 4.0.

The following criteria will be used to define DLT:

- Any ≥ Grade 3 non-hematologic toxicity **except**:
  - Grade 3 or 4 in-field radiation dermatitis for which RT is held ≤ 1 week (5 fractions);
  - Grade 3 or 4 mucositis for which RT is held ≤ 1 week (5 fractions);
  - Grade 3 or 4 hypomagnesemia, hypokalemia, or hypophosphatemia without life-threatening consequences, which corrects to Grade ≤ 2 with observation or replacement therapy;
  - Grade 3 pain, dysphagia, weight loss, or fatigue which are expected toxicities for chemoradiation of HNSCC and will be managed aggressively by treating investigators per standards of care;
  - Grade 3 endocrine disorder (thyroid, pituitary, and/or adrenal insufficiency) that is managed with or without systemic corticosteroid therapy and/or hormone replacement therapy and the patient is asymptomatic;
  - Grade 3 inflammatory reaction attributed to a local antitumor response (e.g., inflammatory reaction at sites of metastatic disease, lymph nodes, etc.);
  - Grade 3 or 4 concurrent vitiligo or alopecia;
  - Grade 3 infusion-related reaction (first occurrence and in the absence of steroid prophylaxis) that resolves within 6 hours with appropriate clinical management;
  - Isolated Grade 3 electrolyte abnormalities that are not associated with clinical signs or symptoms and are reversed with appropriate maximal medical intervention within 3 days;
  - Grade 3 AST/ALT elevation ≤ 8 x ULN;
  - Grade 3 bilirubin elevation ≤ 5 x ULN.
- Any Grade 3 or 4 immune-related adverse event, excluding colitis or pneumonitis, that does not downgrade to Grade 2 within 3 days despite optimal medical management including systemic corticosteroids or does not downgrade to ≤ Grade 1 or baseline within 14 days;
- Delay in completion of RT > 2 weeks (10 fractions), or inability to complete prescribed RT course, due to immune toxicity definitely or probably related to MEDI4736;
- Grade 3 or 4 neutropenia with fever (oral temperature > 39°C);
- Grade 3 or 4 neutropenia that is not associated with fever or systemic infection that does not improve by at least 1 grade within 3 days;
- Grade 3 or 4 thrombocytopenia with bleeding;
- Grade 3 or 4 thrombocytopenia that is not associated with clinically significant bleeding that requires medical intervention that does not improve by at least 1 grade within 3 days;
- Grade 3 AST/ALT elevation > 8 × ULN;

- Grade 3 bilirubin elevation > 5 × ULN;
- Any Grade 5 toxicity.

Phase II
It is hypothesized that MEDI4736 (durvalumab) given concurrently with radiation for patients with locoregionally advanced HNC who have a contraindication to cisplatin will show a signal for improved PFS, compared to patients treated with concurrent radiation and cetuximab.

Progression-free survival is defined as time from randomization until first evidence of local, regional, or distant disease progression or recurrence, or death from any cause. (Note: PFS factors in effects of therapies on both cancer events and competing mortality events. Both are important to assess the net benefit of therapies in this population. There is reason to believe that MEDI4736 (durvalumab) will have a beneficial effect on both cancer events and competing events relative to cetuximab. Endpoints like local-regional control or time to progression would not be sensitive to a beneficial effect on competing mortality. PFS is thus the best phase II primary endpoint to assess effectiveness).

Phase III
It is hypothesized that MEDI4736 (durvalumab) given concurrently with radiation for patients with locoregionally advanced HNC who have a contraindication to cisplatin will improve OS, compared to patients treated with radiation and cetuximab.

Overall survival is defined as time from randomization until death from any cause.

14.3.2 How Primary Endpoints Will Be Analyzed

Lead-In
All patients who receive at least one dose of MEDI4736 (durvalumab) are considered evaluable for DLT, should a DLT occur. In the absence of DLT, patients must have received at least one dose of MEDI4736 (durvalumab) and one fraction of radiation therapy and have completed the DLT observation period to be evaluable for DLT. Patients considered inevaluable may be replaced to ensure the required number of evaluable patients per arm.

For the first dose level, 8 patients will be evaluated. It is projected that 10 patients will be entered to reach the required 8 evaluable patients. If more than 8 patients are evaluable, data from the first 8 patients will be analyzed for the DLT endpoint. In the event of 0-2 DLT events, the experimental therapy is considered safe and the study will proceed to phase II. Otherwise, the MEDI4736 (durvalumab) dose will be de-escalated. Should dose de-escalation be necessary, then a second (and, if necessary, third) dose level will be evaluated with 8 patients each. It is projected that 10 patients will be entered to reach the required 8 evaluable patients. If more than 8 patients are evaluable, data from the first 8 patients will be analyzed for the DLT endpoint. For a given de-escalated dose, in the event of 0-2 DLT events, the experimental therapy is considered safe and the study will proceed to phase II. The treatment plans for dose de-escalation (if necessary) appear in Tables 1 and 2 in Section 5.

Phase II
The intention-to-treat (ITT) population (all randomized patients) will be used for the primary analysis of PFS. PFS will be estimated using the Kaplan-Meier method (1958). The difference in PFS between the two arms will be tested using a log-rank test. If the PFS endpoint is met in Phase II, then the study will continue.

If the PFS endpoint is not met, the study will be reported, including exploratory analysis adjusting for important factors such as age, site, Zubrod performance status, and clinical stages will be conducted using Cox models, otherwise, multivariable analyses for PFS will be done at the end of the Phase III portion of the study.

Phase III
The intention-to-treat (ITT) population (all randomized patients) will be used for the primary analysis of

OS. OS will be estimated using the Kaplan-Meier method (1958). The difference in OS between the two arms will be tested using a log-rank test. Exploratory analysis adjusting for important factors such as age, site, Zubrod performance status, and clinical stages will be conducted using Cox models.

14.3.3 Sample Size and Power Calculations

Lead-In

The lead-in will test a fixed-dose in a cohort of 8 evaluable patients for a given dose level. In the event of 0-2 DLT events, the experimental therapy is considered safe and the study will proceed to phase II. With a cohort of 8 patients, the probability of the experimental arm being judged to be too toxic when the true toxicity rate is 45% or higher is at least 78%. If the true toxicity rate is 20% or lower, the probability that the therapy will be safe is 80%. Should dose de-escalation be necessary, then a second (and, if necessary, third) dose level will include 8 analyzable patients each (expecting to enroll 10 patients accrued to get 8 analyzable).

Phase II

A sample size of 234 randomized patients is required, assuming an accrual rate of 10 patients per month, total accrual time of 2.2 years, 2:1 distribution of patients in the experimental and control arms, a hazard ratio (HR) of 0.65 for the experimental arm relative to control, a 2-year PFS of 40.3% in the control arm (median PFS 1.53 years for the control arm, 2.35 years for the experimental arm), one-sided alpha of 0.20, and power 0.80. A total of 69 PFS events will be needed to achieve the statistical power. Accounting for 10% non-randomization, we expect to enroll 260 patients in order to obtain the required 234 randomized patients. When we randomize 234 patients, we expect to observe the total number of events needed for the phase II final analysis. If so, there will be no or minimal time needed for the analysis. On the other hand, if we still need time to observe certain number of events before reaching the total required for the final analysis, accrual will be suspended temporarily to observe any remaining events.

Revised Phase II Sample Size: The accrual to the phase II portion stopped with 186 randomized patients following an interim futility analysis and toxicity monitoring results. The phase II primary endpoint analysis will be done once 69 PFS events have been observed in the 186-patient ITT population using the design parameters specified in Section 14.4.2. Phase III target accrual remains unchanged.

Phase III

An additional 210 randomized patients will be required, assuming an accrual rate of 10 patients per month, total additional accrual time of 4.1 years, 2:1 distribution of patients in the experimental and control arms, a hazard ratio (HR) of 0.73 for the experimental arm relative to control, a 2-year OS of 46.6% in the control arm (median OS 1.82 years for the control arm, 2.49 years for the experimental arm), two-sided alpha of 0.05, and power 0.80. A total of 364 OS events will be needed to achieve the statistical power. Accounting for 10% non-randomization, we expect to enroll an additional 233 patients. In total (excluding lead-in), we expect to enroll 493 patients in order to obtain the required 444 randomized patients. The exact timing of the analysis for OS will be based on the required total number of events.

14.4 Study Monitoring of Primary Objectives (09-MAR-2022)

14.4.1 Interim Reports to Monitor the Study Progress

Interim reports with will be prepared twice per year until the initial treatment results have been presented/published. In general, the interim reports will contain the following information:

- patient accrual rate with a projected completion date (while the study is still accruing)
- total patients accrued
- distributions of important pre-treatment and prognostic baseline variables
- the frequencies and severity of adverse events by treatment arm

The interim reports will not contain the results from the primary endpoints (DLTs, PFS, OS) or any secondary endpoints, with the exception of routine reporting of adverse events.

14.4.2 Significance Testing for Early Stopping and/or Reporting

Phase II

In phase II, the primary endpoint PFS will be tested for the interim analysis when there are 35 events. If the

observed hazard ratio is ≥ 1.0 favoring control arm, then early stopping will be considered, with the conclusion being that this regimen would not be a candidate for further evaluation in phase III. The interim results will be reported to the NRG Oncology DMC for its review and recommendation regarding next steps of the trial.

At the phase II final analysis, a log-rank test will be used for the comparison of the two treatment arms; the total required PFS events is 69 at 2.2 years from accrual start. If the hazard ratio is ≤ 0.806, we will reject the null hypothesis and continue to phase III. This analysis will be performed within 6 weeks from the date the required number of events are observed.

Key Sensitivity Analysis to the Phase II Primary Endpoint*:* When phase II accrual was halted, some patients that were still under RT+durvalumab treatment were allowed to switch to off-protocol treatment after the release of the Dear Investigator Letter/Dear Patient Letter. Therefore, a key sensitivity analysis to the protocol-specified phase II primary analysis will be performed to inform the decision-making regarding continuing to the phase III portion using the following approach: *patients randomized to the RT+durvalumab arm eight weeks or less prior to the date of release of the DP/DI letters (8/9/2021) will be censored at the release time. All the remaining patients receiving RT+durvalumab at the release time will be included in the final analysis without this censoring type.*

If at the time of phase II primary endpoint analysis the study statistician recommends reopening the trial based on the protocol-specified design, all relevant outcome and toxicity information supporting the reopening recommendation will be provided to CTEP in a confidential manner to get approval before this recommendation is presented to the NRG DMC.

The NRG DMC will be notified regarding the Go/No Go decision in phase II.

Phase III
There will be two interim analyses on OS when 182 (50%), and 273 (75%) deaths are observed for the two arms combined. The results will be reported to the NRG Oncology DMC with recommendations based on the results. An O'Brien-Fleming boundary will be utilized for efficacy monitoring. The stopping hazard ratio (HR) boundary and cumulative alpha spent are shown in the table below:

# Events	Hazard ratio	One-sided significance level	Cumulative alpha spent
182	0.627	0.002	0.002
273	0.739	0.008	0.010

For futility, the statistical monitoring boundary will be based on the LIB20 method at the same analysis times, as recommended by Freidlin (2010).

If the study is not stopped at an interim analysis, the final analysis will occur when 364 total deaths are observed and tested at a 1-sided significance level of 0.021, for a cumulative alpha level of 0.025.

14.4.3 Data Monitoring Reviews

Lead-In
The study data, especially adverse events, will be closely monitored by the study team including but not limited to the study PI, co-PIs, and statistician, as listed on the protocol cover page. Given the projected monthly accrual and required data submission, a minimum of monthly conference calls will be held with the full study team (as described above) to review the study data, especially SAEs and DLTs. More frequent calls will be held as needed. Information will be disseminated to institutional PIs per standard practice of NRG Oncology. The study chair will follow up any report of DLT with individual investigators.

Phase II/III
The NRG Oncology Data Monitoring Committee (DMC) will review the randomized portion of the study twice a year with respect to patient accrual and morbidity. The DMC also will review the study for protocol-specified primary endpoint interim analyses, as well as on an "as needed" basis for any other

issues.

14.5 Accrual/Study Duration Considerations (09-MAR-2022)

14.5.1 Lead-In

The accrual rate will be 5 patients per month in the lead in phase. During the first three months after study activation, minimal accrual is expected. So the duration from start of accrual to primary endpoint reporting is 8-9 months for the initial dose of the lead-in, including accrual ramp-up time. If de-escalation is required, the duration from start of accrual to primary endpoint reporting for each additional dose level is approximately 8 months. The lead in accrual will be monitored according to the CTEP early phase slow accrual guidelines.

14.5.2 Phase II and Phase III

The monthly accrual will be 10 patients per month for the phase II/III component, with minimal accrual is expected during the first three months after the Phase II begins. The accrual will be monitored according to the CTEP late phase slow accrual guidelines.

<u>Accrual Goal and Duration</u>
Phase II: 234 randomized patients (projecting 260 enrolled to reach 234 randomized) accrued over 2.2 years.

<u>Actual Phase II Accrual (Revised)</u>: 186 randomized patients and 190 enrolled patients.

Phase III: 444 randomized patients, including the 234 randomized in Phase II (projecting 493 enrolled to reach 444 randomized), accrued over 4.1 years (including the Phase II accrual time)

<u>Estimated Duration for Completion of Primary Endpoint</u>

<u>Phase II</u>
It is projected that the 69 PFS events required for the PFS primary endpoint analysis will occur approximately 2.2 years after the Phase II study activation, depending on the length of the safety lead-in portion, and approximately 3 years (assuming one cohort for the lead-in) after the randomized accrual begins.

<u>Phase III</u>
It is projected that the 364 deaths required for the OS primary endpoint analysis will occur approximately 8.1 years after study activation, depending on the length of the safety lead-in portion, and approximately 6.5 years after the randomized accrual begins.

14.6 Secondary Endpoints (15-JAN-2019)

14.6.1 Response

The 4-month response by FDG-PET/CT will be measured by RECIST. Response rates between the two arms will be compared using Fisher's exact test.

14.6.2 Local-Regional Failure and Distant Metastases

The intention-to-treat (ITT) population (all randomized patients) will be used for LRF and DM analyses. LRF is defined as time from randomization until first evidence of local, regional disease progression or recurrence, or death from study cancer or unknown causes. DM is defined as time from randomization until first evidence of distant metastasis. The table below shows the failures and competing risks for each of the three endpoints. LRF and DM will be estimated by cumulative incidence methods and compared using a cause-specific log-rank test. All failure times will be measured from the date of study randomization to the date of failure or last follow up.

**First event**	**Progression-Free Survival**	**Local-Regional Failure**	**Distant Metastasis**
None	Censored	Censored	Censored
Local-regional progression or recurrence	Failure	Failure	Competing risk

Distant metastasis	Failure	Competing risk	Failure
Death due to study cancer or from unknown causes	Failure	Failure	Competing risk
Death due to any other reason	Failure	Competing risk	Competing risk

14.6.3 Toxicity

CTCAE

For toxicity analysis, all treated patients will be included in the analysis. The rates of adverse events will be estimated using a binomial distribution along with their associated 95% confidence intervals and will be compared using Fisher's exact test.

PRO-CTCAE

PRO-CTCAE is not intended for expedited reporting, real time review or safety reporting. PRO-CTCAE data are exploratory and not currently intended for use in data safety monitoring or adverse event stopping rules. Once a patient submits the responses, the data goes directly from the device into the Rave database. There are no documents to audit. The electronic responses are the source documentation.

14.6.4 Competing Mortality

Competing mortality for PFS and OS is defined as deaths due to other causes. This will be estimated by cumulative incidence methods and the effects of other covariates will be assessed using the GCE model.

14.6.5 Quality of Life Analysis

EORTC QLQ-C30/HN35

The focus of the quality of life (QOL) analysis is the EORTC QLQ-C30/H&N35 change in score from baseline to 12 months from the end of RT. The null hypothesis (H_0) states that there is no difference in mean change in QOL physical function domain of EORTC QLQ between the MEDI4736-RT and cetuximab-RT arms. The alternative hypothesis (H_A) states that there is a clinically meaningful difference in this mean change, whereby MEDI4736 will have a less decline in QOL compared to the cetuximab arm at one year from end of RT. A ≥ 10-point difference in mean change in score between arms is considered a minimum important change. A 10-point change on a 0-100 points scale in physical functioning QOL domain of the EORTC QLQ 30 is considered clinically meaningful. The intention-to-treat (ITT) population (all randomized patients) will be used for QOL analyses. Sensitivity analyses limited to patients that started protocol treatment may also be done.

Based on prior trials in head and neck cancers, assuming the attrition rate is 35% at 12 months, it is projected that 289 randomized patients will be evaluable for this analysis. To be conservative in power estimation, a standard deviation of 26.7 for the physical function domain of the EORTC QLQ mean change score is assumed. With a two-sided test at an alpha of 0.05, there will be 80% statistical power to detect a difference between treatment arms in mean change scores of 0.35 standard deviations.

Overall, the mean individual domain scores of the EORTC QLQ/H&N35, overall summary score of the EQ5D and MDADI, and the subscales will be determined. The mean change from baseline at each time point will be summarized using mean and standard deviations for each arm. Mean change from baseline will be compared between the arms using a two sample t test. If data normality assumptions are not met, the Wilcoxon rank sum test will be used to test the hypothesis

In addition to the primary hypothesis, exploratory analyses will evaluate change from baseline to other time points, as well as other domains of the EORTC QLQ/HN35.

MDADI

The MDADI will measure swallowing related QOL. Two summary scores can be obtained from the MDADI: 1) global and 2) composite. The global scale is a single question "my swallowing impacts my day-to-day life". The composite MDADI score summarizes overall performance on remaining 19-items of the MDADI, as a weighted average of the physical, emotional, and functional subscale questions. Summary and subscale MDADI scores are normalized to range from 20 (extremely low functioning) to 100 (high functioning). The composite MDADI score will be chosen as the primary swallowing endpoint as it reflects

overall performance on 19-items (Hutcheson, 2016). The MDADI total composite score for clinically acceptable swallowing is defined as total composite MDADI score ≥ 60. A secondary QOL objective is to compare swallowing related QOL between arms and it is hypothesized that the MEDI4736-RT will have less swallowing QOL decline, based on the change in score from baseline to 12 months from end of RT, compared to the cetuximab arm. The Null Hypothesis states that is that there is no difference in swallowing related QOL decline between arms. The swallowing QOL endpoint will measure the mean individual change in total composite MDADI score at 1 year (from end of RT) from baseline in each arm. One of the arms will be deemed inferior to the other if there is a mean change score of ≥ 10 points from baseline for that arm and there is a ≥ 10 point difference between the 2 arms that is statistically significant at a 2-sided alpha of 0.05. Exploratory analyses will evaluate the percentage of patients in each arm with poor swallowing QOL, defined as an individual patient total composite MDADI score of < 60 at 12 months from end of RT and will also evaluate the subscale scores (global, physical, emotional, functional) of the MDADI inventory, with particular focus on the physical and functional subscales at 12 months from end of RT and the EORTC HN35 swallowing domain.

QOL will be measured during the phase II and III portion of the study. In addition to comparing the change scores at end of RT and at 4, 8, 12, 18, and 24 months from end of RT to baseline, overall trends in these QOL tools, and subscale scores will be modeled using the general linear mixed-effect model. This model will be used to compare the differences of scores over time between the two arms and to compute least squares mean and SEs, while adjusting for clinical variables and treatment by visit interaction terms. The model also allows for adjustments using stratification variables and other covariates of interest. The use of general linear mixed modeling allows flexibility in analyzing data with missing responses. For QOL endpoints, based on results from 175 patients enrolled on RTOG 0522 and Ringash (2004), the mean change score is approximately half of the standard deviation. Also, from the results of Curran (2007), we expect a meaningful between group change from baseline of 10 points on the EORTC QLQ/H&N35.

Based on prior trials in head and neck cancers, assuming the attrition rate is 35% at 12 months, we expect 289 patients will be randomized for the evaluation at 1 year. To be conservative in power estimation, we assume a standard deviation of 26.7 for physical function domain of the EORTC QLQ change score and MDADI at 12 months (from end of RT) from baseline. With a two-sided test of 5%, even if the change from baseline were as small as 0.375 of the standard deviation, the statistical power would be 88%.

PSS-HN

The PSS-HN is a clinician-rated instrument consisting of 3 subscales: normalcy of diet, public eating, and understandability of speech, each of which is rated from 0 to 100, with higher scores indicating better performance. In this study it is hypothesized that MEDI4736 (durvalumab) will have less decline in long-term swallowing function with respect to normalcy of diet and public eating from baseline to 12 months from the end of RT compared to the control arm. The mean score change for each of these subscales between arms will be compared using a two-sample independent t-test with a two-sided significance level of 0.05. A Wilcoxon test will be used if normality assumption does not hold. Temporal trends and differences between arms at other time points will be assessed using mixed models with the following covariates: time, treatment arm and its interaction.

Addressing Missing Data

To handle poor compliance and missing data, efforts will be made to minimize attrition due to avoidable factors. To assess the missing data mechanism, we will compare possible differences between patients who dropped out of the study against those who remained in the study with respect to imbalance factors such as treatment, baseline scores, clinical and demographic data. We will undertake sensitivity analyses to investigate reasons for missingness (e.g. by drop-out), considering various factors as mentioned earlier. A logistic regression model will be used to summarize number of missing data and to test if the dropout process is missing completely at random (MCAR). Analysis of complete cases and cases with multiple imputations for missing observations (before death) will be done to check robustness of the main results. A pattern mixed model or selection model will be used to assess treatment effect to see if it is dropout dependent. Cox proportional hazard models will be used to determine the prognostic effects of baseline QOL on overall survival after adjusting for RT level, MEDI4736 (durvalumab) or cetuximab usage and other potential factors.

14.6.6 Correlative Biomarkers

The primary correlative hypotheses are that PD-L1 and p16 are prognostic and possibly predictive for OS. To test these hypotheses, analyses of interaction between treatment arm and marker status will be done. Additionally toxicity for the two arms by marker status will be compared. Although both analyses are exploratory, we provide power for the prognostic effects on OS as shown in the following table. For dichotomized variables, the statistical power can be calculated by the method of Schoenfeld (1981). The table below shows statistical power to detect hazard ratios of 1.25, 1.50, 1.75, 2.00, 2.25, and 2.50 for prevalence rates of 10%, or 20%, or 30%, etc. Statistical power will be the same if prevalence rate is 1-prevalence. The significance level was set at 0.05. As seen in the table, there will be > 87% power (given prevalence of >10% of the factor of interest in the study population) to detect a hazard ratio of 1.50 or greater for two arms combined.

Overall Survival
Statistical Power to Detect Various Hazard Ratios with 2-Sided 0.05

		Hazard Ratio					
Proportion Marker+	Events	1.25	1.50	1.75	2.00	2.25	2.50
0.90 or 0.10	364	0.24	0.64	0.89	0.97	0.99	0.99
0.80 or 0.20	364	0.39	0.87	0.98	0.99	0.99	0.99
0.70 or 0.30	364	0.49	0.94	0.99	0.99	0.99	0.99
0.60 or 0.40	364	0.55	0.96	0.99	0.99	0.99	0.99
0.50	364	0.56	0.97	0.99	0.99	0.99	0.99

Univariable and multivariable analysis will be performed using the Cox proportional hazards model for OS. Potential covariates evaluated for the multivariate models would be assigned treatment, age, Zubrod performance status, T-stage, N-stage, primary site, smoking history, other risk factors, as well as p16 and/PD-L1.

14.6.7 Gastrostomy Tube Retention Rates

Gastrostomy tube retention rates between arms at 1 year from the end of the RT will be compared using a Chi-square test with a two-sided alpha level of 0.05. Comparison of rates between arms at other time-points will be done if it is deemed necessary.

14.7 Exploratory Analyses (21-AUG-2019)

Translational (Exploratory) Analyses on Peripheral Blood Specimens

The exploratory analyses in this section will be performed conditional on sufficient funding. Samples from the first 100 randomized patients at the different time points will be collected. We will test the hypothesis that MEDI4736 (durvalumab) combined with radiation enhances the adaptive immune response compared to cetuximab plus radiation. We will interrogate this hypothesis using three distinct methods of Immunophenotyping. Blood samples will be collected from patients into sodium heparin coated vacutainers (BD Vacutainer® Green BD Hemogard™) at time points specified under the study protocol: 1) Baseline (within 14 days prior to starting immunotherapy), 2) within 7 days prior to starting RT (and after starting systemic therapy), 3) 1 month after end of RT, 4) 1 month after last dose of systemic therapy (durvalumab arm) or 4 months after RT (cetuximab arm).

We will perform Multiparametric Flow cytometry on peripheral mononuclear cells (PBMC) from patient-derived blood samples to quantify changes in immune cell frequency and activation status before, during and after radiation combined with cetuximab or radiation combined with MEDI4736 (durvalumab) including: T-cell subsets (CD3, CD4, CD8, CD25, FOXP3, CD45RA, CD62L, CD127, CD278, PD-1, IFN-gamma, TNF-alpha), B cells (CD19, CD20), Macrophage I/II and myeloid-derived suppressor cells (MDSC) (CD16, CD68, CD206) and Natural killer cells (CD56, CD16, KIR, CD117, CD94).

1) We will perform Next generation T-cell receptor sequencing (ImmunoSeq), using TCR-Beta CDR3 Kit (Adaptive Biotechnologies). TCR-β CDR3 regions will be amplified and sequenced using the survey ImmunoSeq assay in a multiplexed PCR method with reads obtained using an Illumina HiSeq System.

2) Assessment of plasma IgG anti-tumor antibody responses will be performed using high-content protein microarray. IgGs levels against candidate antigens will validated uses multiplexed antigen-conjugated, spectrally distinguishable, fluorescent proprietary microspheres.
3) For TCR ImmunoSeq Analysis: Shannon entropy will be calculated on the clonal abundance of all productive TCR sequences in the data set. Shannon entropy will be normalized to the range by dividing Shannon entropy by the logarithm of the number of unique productive TCR or BCR sequences in the data set. This normalized entropy value will then inverted (1 − normalized entropy) to produce the clonality metric.

For analytical assessment of three phenotype changes such as plasma IgG anti-tumor antibody responses: changes in plasma IgG levels will be reported relative to pretreatment. To assess the statistical significance of pre- to post-treatment changes in levels of IgGs as well as other markers, normalized signal intensities (log2) will be tested using a paired t test within an arm and two sample t test between the two arms. If the distribution assumption is violated for the t tests, nonparametric tests, such as the Wilcoxon signed-rank test will be considered. IgG response will also be defined as ≥2-fold increase in signal intensity at 3 months post-treatment compared with pre-treatment and compared by a chi-square test. The Benjamini and Hochberg procedure will be used to perform multiple testing adjustment of P values and obtain estimated false discovery rates (FDR).

Lastly, the association between PFS and OS and post-treatment changes in frequency and activation state of T-cells, clonality and diversity of TCR, and IgG levels will be evaluated using a two-sided Wald test at 0.05 level on the basis of Cox models.

From this analysis we will determine:
1) Whether there is any statistically significant difference in the frequency and activation state of T-cells, memory T-cell precursors, B-cells, Macrophages, and NK cells in peripheral blood during or after treatment with radiation combined with cetuximab versus radiation combined with MEDI4736 (durvalumab);
2) Whether there is a statistically significant changes in clonality and diversity of T-cell receptors in the peripheral blood during or after treatment with radiation combined with cetuximab versus radiation combined with MEDI4736 (durvalumab). Additionally the ImmunoSeq analysis will allow us to determine whether there is any statistically significant difference in the magnitude and diversity of adaptive immune responses after the combined treatment.
3) From the IgG analysis, whether there is any statistically significant difference in development of humoral B-cell mediated antibody responses in patients during or after radiation combined with cetuximab versus radiation combined with MEDI4736 (durvalumab).

Patient-Reported Fatigue

Patient reported fatigue is measured using the fatigue items 10, 12, and 18 in the EORTC QLQ-C30. The range of the PR fatigue scores is 0-100, where a high score represents a high level of fatigue. The change in EORTC QLQ fatigue scores from baseline to 4 months from the end of RT between the MEDI4736-RT and cetuximab-RT arms will be compared using a two-sample t-test with a two-sided alpha level of 0.05. If the normality assumption does not hold, a Wilcoxon test will be used. Data analyses of change from baseline to the other collected time points will also be analyzed. A dichotomized version of the EORTC QLQ-C30 raw fatigue scores may be considered. A comparison of severity and interference (attributes a and b in Item 53) of PRO-CTCAE fatigue from baseline to 4 months from the end of RT between arms will be done using a chi-squared test with an alpha level of 0.05. For each patient a new outcome with three categories is defined: improved, stable (no change) and worsened. A patient is labeled as 'improved' if the PRO-CTCAE fatigue score at the baseline is greater than that at 4 months from the end of RT. Conversely, a patient is labeled as 'worsened' if the PRO-CTCAE fatigue score at the baseline is smaller than that at 4 months from the end of RT. Otherwise, the patient is being classified as 'stable (no change)'. The distribution of the new outcome will be compared between the two arms using the chi-square statistics.

Clinician and PRO Toxicity

Grades 1 to 5 in the CTCAE refers to the severity of the AEs, while the AE scores in PRO-CTCAE use a 5-point Likert scale for each attribute of an AE (frequency, severity, amount, interference and presence/absence). PRO-CTCAE scores with an attribute of severity (none, mild, moderate, severe or very severe with scores from 0 to 4, respectively) will be compared to AE scores graded by based on the CTCAE (none, mild, moderate, severe, life threating and death with scores from 0 to 5). The agreement between clinician and PRO toxicity will be assessed using the Pearson correlation between scores and the independence between scores will the tested using the Mantel-Haenszel chi-square statistic with a one-sided alpha level of 0.025 (Agresti, 2002).

Health Utilities Using the EQ5D-5L

Quality adjusted survival or quality adjusted life years (QAYL) will be compared between “fit” and “vulnerable” patients as classified by the G-8 and EQ5D (Pottel 2015). Quality adjusted survival is calculated as the weighted sum of different time in different health states added up to a total quality adjusted survival time (Glasziou, 1990).

The visual analog scale (VAS) and index scores from the EQ-5D-5L will be calculated at each time point (baseline, end of RT, and at 4, 8, 12, 18 and 24 months after RT ends) and compared between treatment arms using a t-test with a 2-sided significance level of 0.05.

14.8 Gender/Ethnicity/Race Distribution (15-JAN-2019)

Lead-In

**Racial Categories**	**DOMESTIC PLANNED ENROLLMENT REPORT**				
	**Ethnic Categories**				
	Not Hispanic or Latino		Hispanic or Latino		Total
	Female	Male	Female	Male	
American Indian/Alaska Native	0	0	0	0	0
Asian	0	0	0	0	0
Native Hawaiian or Other Pacific	0	0	0	0	0

Islander					
Black or African American	1	4	0	0	5
White	4	14	1	2	21
More Than One Race	0	0	0	0	0
Total	5	18	1	2	26

**Racial Categories**	**INTERNATIONAL (including Canadian participants) PLANNED ENROLLMENT REPORT**				
	**Ethnic Categories**				
	Not Hispanic or Latino		Hispanic or Latino		Total
	Female	Male	Female	Male	
American Indian/Alaska Native	0	0	0	0	0
Asian	0	0	0	0	0
Native Hawaiian or Other Pacific Islander	0	0	0	0	0
Black or African American	0	0	0	0	0
White	1	3	0	0	4
More Than One Race	0	0	0	0	0
Total	1	3	0	0	4

Phase II/III

**Racial Categories**	**DOMESTIC PLANNED ENROLLMENT REPORT**				
	**Ethnic Categories**				
	Not Hispanic or Latino		Hispanic or Latino		Total
	Female	Male	Female	Male	
American Indian/Alaska Native	1	3	0	0	4
Asian	1	3	0	0	4
Native Hawaiian or Other Pacific Islander	0	0	0	0	0
Black or African American	14	58	1	5	78
White	57	228	4	17	306
More Than One Race	0	0	0	0	0
Total	73	292	5	22	392

**Racial Categories**	**INTERNATIONAL (including Canadian participants) PLANNED ENROLLMENT REPORT**				
	**Ethnic Categories**				
	Not Hispanic or Latino		Hispanic or Latino		Total
	Female	Male	Female	Male	
American Indian/Alaska Native	0	1	0	0	1

Asian	0	1	0	0	1
Native Hawaiian or Other Pacific Islander	0	0	0	0	0
Black or African American	2	7	0	1	10
White	7	30	1	2	40
More Than One Race	0	0	0	0	0
Total	9	39	1	3	52

REFERENCES (21-AUG-2019)

Adelstein DJ, Lavertu P, Saxton JP, et al. Mature results of a phase III randomized trial comparing concurrent chemoradiotherapy with radiation therapy alone in patients with stage III and IV squamous cell carcinoma of the head and neck. *Cancer*. 2000;88:876-83. PMID: 10679658.

Agresti, A. Categorical Data Analysis. Wiley Series in Probability and Statistics, 2002.

Ahn MJ, D'Cruz A, Vermorken JB, et al. Clinical recommendations for defining platinum unsuitable head and neck cancer patient populations on chemoradiotherapy: A literature review. *Oral Oncol.* 2016 Feb;53:10-6.

Alongi F, Bignardi M, Garassino I, et al. Prospective phase II trial of cetuximab plus VMAT-SIB in locally advanced head and neck squamous cell carcinoma. Feasibility and tolerability in elderly and chemotherapy-ineligible patients. *Strahlenther Onkol.* 2012;188(1):49-55.

Alsidawi S, Westin G, Chintakuntlawar AV, Okuno SH, Price K. The impact of HPV infection on survival of patients with non-oropharyngeal head and neck cancer (abstr.) Proc Amer Soc Clin Oncol; *J Clin Oncol.* 2017;35(suppl; abstr 6048).

Ang KK, Chen A, Curran WJ Jr, et al. Head and neck carcinoma in the United States: First comprehensive report of the Longitudinal Oncology Registry of Head and Neck Carcinoma (LORHAN). *Cancer*. 2012;118(23):5783-92.

Ang KK, Harris J, Wheeler R, et al. Human papillomavirus and survival of patients with oropharyngeal cancer. *N Engl J Med*. 2010;363(1):24-35. PMID: 20530316.

Ang KK, Zhang Q, Rosenthal DI, et al. Randomized phase III trial of concurrent accelerated radiation plus cisplatin with or without cetuximab for stage III to IV head and neck carcinoma: RTOG 0522. *J Clin Oncol*. 2014;32(27):2940-50. PMID: 25154822.

Baxi SS, O'Neill C, Sherman EJ, Atoria CL, Lee NY, Pfister DG, Elkin EB. Trends in chemoradiation use in elderly patients with head and neck cancer: Changing treatment patterns with cetuximab. *Head Neck*. 2016 Apr;38(suppl 1): E165-71. PMID: 25535104.

Bellera CA, Rainfray M, Mathoulin-Pelissier S, et al. Screening older cancer patients: first evaluation of the G-8 geriatric screening tool. *Ann Oncol*. 2012;23(8):2166-72.

Bernard DL, Loeffler DR, Rothrock NE, et al. 2017. The challenge of measuring intra-individual change in fatigue during cancer treatment. *Qual Life Res.* 2017;26(2):259-271.

Bernier J, Domenge C, Ozsahin M, et al. Postoperative irradiation with or without concomitant chemotherapy for locally advanced head and neck cancer. *N Engl J Med*. 2004;350:1945-52. PMID: 15128894.

Bhide, S. A, Gulliford, S, Kazi, R, et al. Correlation between dose to the pharyngeal constrictors and patient quality of life and late dysphagia following chemo-IMRT for head and neck cancer. *Radiother Oncol.* 2009;93(3):539-44.

Bonner JA, Harari PM, Giralt J, et al. Radiotherapy plus cetuximab for locoregionally advanced head and neck cancer: 5-year survival data from a phase 3 randomised trial, and relation between cetuximab-induced rash and survival. *Lancet Oncol*. 2010;11(1):21-8. PMID: 19897418.

Bonner JA, Harari PM, Giralt J, et al. Radiotherapy plus cetuximab for squamous-cell carcinoma of the head and neck. *N Engl J Med*. 2006;354:567-78. PMID: 16467544.

Brizel DM, Albers ME, Fisher SR, et al. Hyperfractionated irradiation with or without concurrent chemotherapy for

locally advanced head and neck cancer. *N Engl J Med*. 1998;338:1798-804. PMID: 9632446.
Carmona R, Zakeri K, Green G, et al. Improved method to stratify elderly cancer patients at risk for competing events. *J Clin Oncol*. 2016;34(11):1270-7.
Cella D, Hahn EA, Dineen, K. Meaningful change in cancer-specific quality of life scores: differences between improvement and worsening. *Qual Life Res.* 2002;11(3):207-21.
Charlson ME, Pompei P, Ales KL, MacKenzie CR. A new method of classifying prognostic comorbidity in longitudinal studies: development and validation. *J Chronic Dis.* 1987;40(5):373-383.
Chen AY, Frankowski R, Bishop-Leone J, et al. The development and validation of a dysphagia-specific quality-of-life questionnaire for patients with head and neck cancer: The M. D. Anderson Dysphagia Inventory. *Arch Otolaryngol Head Neck Surg.* 2001;127(7):870-76.
Chen BJ, Chapuy B, Ouyang J, et al. PD-L1 expression is characteristic of a subset of aggressive B-cell lymphomas and virus-associated malignancies. *Clin Cancer Res*. 2013;19:3462-73.
Chera BS, Eisbruch A, Murphy BA, et al. Recommended patient-reported core set of symptoms to measure in head and neck cancer treatment trials. *J Natl Cancer Inst.* 2014;106(7). PMID: 25006189.
Chung CH, Zhang Q, Kong CS, et al. p16 protein expression and human papillomavirus status as prognostic biomarkers of nonoropharyngeal head and neck squamous cell carcinoma. *J Clin Oncol* 2014;32:3930-8.
Cooper JS, Pajak TF, Forastiere AA, et al. Postoperative concurrent radiotherapy and chemotherapy for high-risk squamous-cell carcinoma of the head and neck. *N Engl J Med*. 2004;350:1937-44. PMID: 15128893.
Cooper JS, Zhang Q, Pajak TF, et al. Long-term follow-up of the RTOG 9501/intergroup phase III trial: postoperative concurrent radiation therapy and chemotherapy in high-risk squamous cell carcinoma of the head and neck. *Int J Radiat Oncol Biol Phys.* 2012;84(5):1198-1205.
Coyne JC, Pajak, T. F, Harris, J, Konski, A, Movsas, B, Ang, K, Watkins Bruner, D. Emotional well-being does not predict survival in head and neck cancer patients: A Radiation Therapy Oncology Group study. *Cancer.* 2007;110(11):2568-75.
Dansky Ullmann C, Harlan LC, Shavers VL, Stevens JL. A population-based study of therapy and survival for patients with head and neck cancer treated in the community. *Cancer.* 2012;118(18):4452-61.
Eisbruch A, Kim HM, Feng FY, et al. Chemo-IMRT of oropharyngeal cancer aiming to reduce dysphagia: swallowing organs late complication probabilities and dosimetric correlates. *Int J Radiat Oncol Biol Phys.* 2011;81(3):e93-9.
Fairman D, Narwal R, Liang M, et al. Pharmacokinetics of MEDI4736, a fully human anti-PDL1 monoclonal antibody, in patients with advanced solid tumors. *J Clin Oncol.* 2014;32(15): 2602.
Fakhry C, Westra WH, Wang SJ, et al. The prognostic role of sex, race, and human papillomavirus in oropharyngeal and nonoropharyngeal head and neck squamous cell cancer. *Cancer*. 2017;123(9):1566-75.
Falchook AD, Green R, Knowles ME, et al. Comparison of patient- and practitioner-reported toxic effects associated with chemoradiotherapy for head and neck cancer. *JAMA Otolaryngol Head Neck Surg.* 2016;142(6):517-23.
Feng FY, Kim HM, Lyden, TH, et al. Intensity-modulated radiotherapy of head and neck cancer aiming to reduce dysphagia: early dose-effect relationships for the swallowing structures. *Int J Radiat Oncol Biol Phys.* 2007;68(5):1289-98.
Ferris RL, Blumenschein GR, Fayette J, et al. Further evaluations of nivolumab (nivo) versus investigator's choice (IC) chemotherapy for recurrent or metastatic (R/M) squamous cell carcinoma of the head and neck (SCCHN): CheckMate 141 (abstr.) *J Clin Oncol* 2016;34(suppl; abstr 6009).
Firat S, Byhardt RW, Gore E. Comorbidity and Karnofksy performance score are independent prognostic factors in stage III non-small-cell lung cancer: an institutional analysis of patients treated on four RTOG studies. Radiation Therapy Oncology Group. *Int J Radiat Oncol Biol Phys* 2002;54(2):357-64.
Forastiere AA, Zhang Q, Weber RS, et al. Long-term results of RTOG 91-11: a comparison of three nonsurgical treatment strategies to preserve the larynx in patients with locally advanced larynx cancer. *J Clin Oncol.* 2013;31(7): 845-52.
Freidlin B., Korn E.L., Gray R., (2010), A general inefficacy interim monitoring rule for randomized clinical trials. Clinical Trials 7, 197-208.
Fu KK, Pajak TF, Trotti A, et al. A Radiation Therapy Oncology Group (RTOG) phase III randomized study to compare hyperfractionation and two variants of accelerated fractionation to standard fractionation radiotherapy for head and neck squamous cell carcinomas: first report of RTOG 9003. *Int J Radiat Oncol Biol Phys.* 2000;48(1):7-16.
Glasziou, P. P., Simes, R. J., Gelber, R. D. Quality adjusted survival analysis. *Stat Med.* 1990;9(11):1259-1276.
Gottesman SR, Walford RL, Thorbecke GJ. Proliferative and cytotoxic immune functions in aging mice. II. Decreased generation of specific suppressor cells in alloreactive cultures. *J Immunol.* 1984;133(4):1782-7.

Grégoire V, Ang K, Budach W, et al. Delineation of the neck node levels for head and neck tumors: a 2013 update. DAHANCA, EORTC, HKNPCSG, NCIC CTG, NCRI, RTOG, TROG consensus guidelines. *Radiother Oncol.* 2014;110(1):172–81.
Gulliford SL, Miah AB, Brennan S, et al. Dosimetric explanations of fatigue in head and neck radiotherapy: an analysis from the PARSPORT Phase III trial. *Radiother Oncol.* 2012;104(2):205-12.
Haynes L, Maue AC. Effects of aging on T cell function. *Curr Opin Immunol*. 2009;21(4):414-7. PMID: 19500967.
Haynes L, Eaton, SM. The effect of age on the cognate function of CD4+ T cells. *Immunol Rev* 2005;205:220-8; PMID: 15882356.
Hurria A, Cirrincione, CT, Muss, HB, et al. Implementing a geriatric assessment in cooperative group clinical cancer trials: CALGB 360401 *J Clin Oncol*. 2011;29(10):1290–96.
Hurria A, Gupta S, Zauderer M, et al. Developing a cancer-specific geriatric assessment: A feasibility study. *Cancer*. 2005;104(9): 1998-2005. PMID: 16206252.
Hutcheson KA, Barrow MP, Lisec A, Barringer DA, Gries K, Lewin JS. What is a clinically relevant difference in MDADI scores between groups of head and neck cancer patients? *Laryngoscope*. 2016;126(5):1108-13.
Jemal A, Siegel R, Xu J, Ward E. Cancer statistics. *CA Cancer J Clin*. 2010;60(5):277-300.
Jensen AD, Bergmann ZP, Garcia-Huttenlocher H, et al. Cetuximab and radiation for primary and recurrent squamous cell carcinoma of the head and neck (SCCHN) in the elderly and multi-morbid patient: a single-centre experience. *Head Neck Oncol.* 2010;2:34.
Kallogjeri D, Gaynor SM, Piccirillo ML, Jean RA, Spitznagel EL Jr, Piccirillo JF. Comparison of comorbidity collection methods. *J Am Coll Surg.* 2014;219(2):245-55.
Kaplan EL, Meier P. Nonparametric estimation from incomplete observations. *J Amer Stat Assoc*. 1958;53:457-81.
King MT. 2011. A point of minimal important difference (MID): A critique of terminology and methods. *Expert Rev Pharmacoecon Outcomes Res.* 2011;11 (2):171-184.
Kirilovsky A, Marliot F, El Sissy C, Haicheur N, Galon J, Pagès F. Rational bases for the use of the Immunoscore in routine clinical settings as a prognostic and predictive biomarker in cancer patients. *Int Immunol*. 2016;28(8):373-82.
Kish JA, Zhang Q, Langer CJ, et al. The effect of age on outcome in prospective, phase III NRG Oncology/RTOG trials of radiotherapy (XRT) +/- chemotherapy in locally advanced (LA) head and neck cancer (HNC) [abstract]. *J Clin Oncol*. 2015;33 (suppl; abstr 6003).
Lacas B, Bourhis J, Overgaard J, Zhang Q, Grégoire V, Nankivell M, Zackrisson B, Szutkowski Z, Suwiński R, Poulsen M, O'Sullivan B, Corvò R, Laskar SG, Fallai C, Yamazaki H, Dobrowsky W, Cho KH, Beadle B, Langendijk JA, Viegas CMP, Hay J, Lotayef M, Parmar MKB, Aupérin A, van Herpen C, Maingon P, Trotti AM, Grau C, Pignon JP, Blanchard P; MARCH Collaborative Group. Role of radiotherapy fractionation in head and neck cancers (MARCH): an updated meta-analysis. Lancet Oncol. 2017 Sep;18(9):1221-1237.
Levendag PC, Teguh DN, Voet P, et al. Dysphagia disorders in patients with cancer of the oropharynx are significantly affected by the radiation therapy dose to the superior and middle constrictor muscle: A dose-effect relationship. *Radiother Oncol.* 2007;85(1):64-73.
Liang H, Deng L, Burnette B, Weichselbaum RR, Fu YX. Radiation-induced tumor dormancy reflects an equilibrium between the proliferation and T lymphocyte-mediated death of malignant cells. *Oncoimmunology*. 2013;2(9): e25668. PMID: 24319637.
List MA, Mumby, P, Haraf D, et al. Performance and quality of life outcome in patients completing concomitant chemoradiotherapy protocols for head and neck cancer. *Qual Life Res*. 1997;6(3): 274-284.
List MA, Siston A, Haraf D, et al. Quality of life and performance in advanced head and neck cancer patients on concomitant chemoradiotherapy: A prospective examination. *J Clin Oncol.* 1999;17(3):1020-28.
Liuu E, Canoui-Poitrine F, Tournigand C, et al. Accuracy of the G-8 geriatric-oncology screening tool for identifying vulnerable elderly patients with cancer according to tumour site: the ELCAPA-02 study. *J Geriatr Oncol.* 2014;5(1):11-19.
Lustgarten J, Dominguez AL, Thoman M. Aged mice develop protective antitumor immune responses with appropriate costimulation. *J Immunol.* 2004;173:4510-5.
Lyford-Pike S, Peng S, Young GD, et al. Evidence for a role of the PD-1: PD-L1 pathway in immune resistance of HPV-associated head and neck squamous cell carcinoma. *Cancer Res*. 2013;73(6):1733-41. PMID: 23288508.
MacFarlane AW, Jillab M, Plimack ER, et al. PD-1 expression on peripheral blood cells increases with stage in renal cell carcinoma patients and is rapidly reduced after surgical tumor resection. *Cancer Immunol Res*. 2014;2:320-31.
Magrini SM, Buglione M, Corvò R, et al. Cetuximab and radiotherapy versus cisplatin and radiotherapy for locally advanced head and neck cancer: A randomized phase II trial. *J Clin Oncol.* 2016;34(5):427-35.

Mell LK, Dignam JJ, Salama JK, et al. Predictors of competing mortality in advanced head and neck cancer. *J Clin Oncol*. 2010;28(1):15-20. PMID: 19933920.
Mell LK, Zakeri K, Rose BS. On lumping, splitting, and the nosology of clinical trial populations and end points. *J Clin Oncol*. 2014;32(10):1089-90. PMID: 24550413.
Mell LK, Shen H, Nguyen-Tan PF, et al. Nomogram to predict the benefit of intensive treatment for locoregionally advanced head and neck cancer. *Clinical Cancer Research* 2019; in press.
Michiels S, Le Maître A, Buyse M, et al. Surrogate endpoints for overall survival in locally advanced head and neck cancer: meta-analyses of individual patient data. *Lancet Oncol.* 2009;10(4):341-50.
Miller MD, Paradis CF, Houck PR, et al. Rating chronic medical illness burden in geropsychiatric practice and research: application of the Cumulative Illness Rating Scale. *Psychiatry Res.* 1992;41:237-48.
Mosley RL, Koker MM, Miller RA. Idiosyncratic alterations of TCR size distributions affecting both CD4 and CD8 T cell subsets in aging mice. *Cell Immunol.* 1998;189(1):10-8.
Movsas B, Hunt D, Watkins-Bruner D, et al. Can electronic web-based technology improve quality of life data collection? Analysis of Radiation Therapy Oncology Group 0828. *Pract Radiat Oncol.* 2014;4(3):187-91.
Nishijima TF, Muss HB, Shachar SS, Moschos SJ. Comparison of efficacy of immune checkpoint inhibitors (ICIs) between younger and older patients: A systematic review and meta-analysis. *Cancer Treat Rev*. 2016;45:30-7.
Niska JR, Halyard, MY, Tan AD., Atherton PJ, Patel SH, & Sloan JA. Electronic patient-reported outcomes and toxicities during radiotherapy for head-and-neck cancer. *Qual Life Res.* 2017, Feb in press.
Perez-Soler R, Delord J, Halpern A, et al. HER1/EGFR inhibitor-associated
rash: Future directions for management and investigation outcomes from the HER1/EGFR
Inhibitor Rash Management Forum. *Oncologist*. 2005;10:345-56,.
Pfister DG, Spencer S, Brizel DM, et al. Head and neck cancers, Version 1. *J Natl Compr Canc Netw*. 2015;13(7):847-55.
Pignon JP, le Maître A, Maillard E, Bourhis J; MACH-NC Collaborative Group. Meta-analysis of chemotherapy in head and neck cancer (MACH-NC): An update on 93 randomised trials and 17,346 patients. *Radiother Oncol*. 2009;92(1):4-14.
Pottel L, Lycke M, Boterberg T, et al. G-8 indicates overall and quality-adjusted survival in older head and neck cancer patients treated with curative radiochemotherapy. *BMC Cancer.* 2015;15:875.
Pottel L, Lycke M, Boterberg T, et al. Serial comprehensive geriatric assessment in elderly head and neck cancer patients undergoing curative radiotherapy identifies evolution of multidimensional health problems and is indicative of quality of life. *Eur J Cancer Care* 2014;23(3):401-412.
Powell C, Schick U, Morden JP, et al. 2014. Fatigue during chemoradiotherapy for nasopharyngeal cancer and its relationship to radiation dose distribution in the brain. *Radiother Oncol.* 2014;110(3) 416-421.
Powles T, Eder JP, Fine GD, et al. MPDL3280A (anti-PD-L1) treatment leads to clinical activity in metastatic bladder cancer. *Nature*. 2014;515(7528): 558-62. PMID: 25428503
Quinten C, Maringwa J, Gotay CC, et al. Patient self-reports of symptoms and clinician ratings as predictors of overall cancer survival. *J Natl Cancer Inst.* 2011;103(24):1851-1858.
Rades D, Seidl D, Janssen S, Bajrovic A, Karner K, Strojan P, Schild SE. Comparison of weekly administration of cisplatin versus three courses of cisplatin 100 mg/m(2) for definitive radiochemotherapy of locally advanced head-and-neck cancers. *BMC Cancer.* 2016;16:437.
Ringash, J., O'Sullivan, B., Bezjak, A., Redelmeier, D. A. Interpreting clinically significant changes in patient-reported outcomes. *Cancer*. 2007;110(1):196-202.
Rose BS, Jeong JH, Nath SK, Lu SM, Mell LK. Population-based study of competing mortality in head and neck cancer. *J Clin Oncol*. 2011;29(26): 3503-9. PMID: 21844503
Rubinstein L, Crowley J, Ivy P, Leblanc M, Sargent D. Randomized phase II designs. *Clin Cancer Res*. 2009;15(6):1883-90. PMID:1927627
Sarris EG, Harrington KJ, Saif MW, Syrigos KN. Multimodal treatment strategies for elderly patients with head and neck cancer. *Cancer Treat Rev*. 2014;40(3):465-75. PMID: 24238923
Segal NH, Ou SH, Balmanoukian AS, et al. Safety and efficacy of MEDI4736, an anti-PD-L1 antibody, in patients from a squamous cell carcinoma of the head and neck (SCCHN) expansion cohort (Abstr.) *J Clin Oncol*. 2015;33:15 (suppl; abstr 3011).
Seiwert TY, Burtness B, Weiss J, et al. A phase Ib study of MK-3475 in patients with human papillomavirus (HPV)-associated and non-HPV–associated head and neck (H/N) cancer. *J Clin Oncol*. 2014;32:5s (suppl; abstr 6011).
Seiwert TY, Weiss J, Baxi SS, et al. A phase 3, randomized, open-label study of first-line durvalumab (MEDI4736) ± tremelimumab versus standard of care (SoC; EXTREME regimen) in recurrent/metastatic (R/M) SCCHN: KESTREL (Abstr.) *J Clin Oncol*. 2016;34(15), (suppl; abstr TPS6101).

Sharabi AB, Nirschl CJ, Kochel CM, et al. Stereotactic radiation therapy augments antigen-specific PD-1 mediated anti-tumor immune responses via cross-presentation of tumor antigen. *Cancer Immunol Res*. 2015;3(4):345-55. PMID: 25527358
Sharabi AB, Lim M, DeWeese TL, Drake CG. Radiation and checkpoint blockade immunotherapy: Radiosensitisation and potential mechanisms of synergy. *Lancet Oncol.* 2015;16(13):e498-509.
Siddiqui F, Gwede CK. Head and neck cancer in the elderly population. *Semin Radiat Oncol*. 2012;22(4):321-33. PMID: 22985815
Sprecher E, Becker Y, Kraal G, Hall E, Harrison D, Shultz LD. Effect of aging on epidermal dendritic cell populations in C57BL/6J mice. *J Invest Dermatol.* 1990:94;247-53. PMID 2299200
Strom TJ, Trotti AM, Kish J, Russell JS, Rao NG, McCaffrey J, Padhya TA, Otto KJ, Caudell JJ. Comparison of every 3 week cisplatin or weekly cetuximab with concurrent radiotherapy for locally advanced head and neck cancer. *Oral Oncol.* 2015;51:704-8.
Thoman ML, Ernst DN, Hobbs MV, Weigle WO. T cell differentiation and functional maturation in aging mice. *Adv Exp Med Biol.* 1993;330:93-106.
Truong MT, Zhang Q, Rosenthal DI, et al. 2017. Quality of life and performance status from a substudy conducted within a prospective phase 3 randomized trial of concurrent accelerated radiation plus cisplatin with or without cetuximab for locally advanced head and neck carcinoma: NRG Oncology Radiation Therapy Oncology Group 0522. *Int J Radiat Oncol Biol Phys.* 2016;97(4):687-99.
Twyman-Saint V, Rech AJ, Maity A, et al. Radiation and dual checkpoint blockade activate non-redundant immune mechanisms in cancer. *Nature.* 2015;520(7547):373-7.
Vainshtein JM, Moon DH, Feng FY, Chepeha DB, Eisbruch A, Stenmark MH. 2015. Long-term quality of life after swallowing and salivary-sparing chemo-intensity modulated radiation therapy in survivors of human papillomavirus-related oropharyngeal cancer. *Int J Radiat Oncol Biol Phys.* 2015;91(5): 925-933.
VanderWalde NA, Meyer AM, Deal AM, et al. Effectiveness of chemoradiation for head and neck cancer in an older patient population. *Int J Radiat Oncol Biol Phys.* 2014;89:30-7.
Wildiers H, Mauer M, Pallis A, et al. End points and trial design in geriatric oncology research: A joint European organisation for research and treatment of cancer—Alliance for Clinical Trials in Oncology–International Society of Geriatric Oncology position article. *J Clin Oncol.* 2013;31(29): 3711-8. PMID: 24019549
WHO GLOBOCAN database: <http://www-dep.iarc.fr> accessed January 31, 2015.
Wong SJ, Harari PM, Garden AS, et al. Longitudinal Oncology Registry of Head and Neck Carcinoma (LORHAN): Analysis of chemoradiation treatment approaches in the United States. *Cancer*. 2011;117(8): 1679-86.
Wookey V, Appiah AK, Kallam A, Ernani V, Smith L, Ganti AK. HPV status and survival in non-oropharyngeal squamous cell carcinoma of the head and neck (abstr.) Proc Amer Soc Clin Oncol; *J Clin Oncol* 35, 2017 (suppl; abstr 6046).
Xiao, C., Hanlon, A., Zhang, Q, et al. 2013. Symptom clusters in patients with head and neck cancer receiving concurrent chemoradiotherapy. *Oral Oncol*. 49(4): 360-66.
Yost KJ, Eton DT, Garcia, SF, Cella D. 2011. Minimally important differences were estimated for six Patient-Reported Outcomes Measurement Information System-Cancer scales in advanced-stage cancer patients. *J Clin Epidemiol.* 64(5): 507-16.
Zakeri K, Rotolo F, Lacas B, Vitzthum LK, Le QT, Gregoire V, Overgaard J, Tobias J, Zackrisson B, Parmar MK, Burtness BA, Ghi MG, Sanguineti G, O'Sullivan B, Fortpied C, Bourhis J, Shen H, Harris J, Pignon J, Mell LK. Predictor of Effectiveness of Treatment Intensification on Overall Survival in Head and Neck Cancer (HNC). *Ann Oncol* (abstr.) 2018; 29 (suppl 8): viii372-viii399.
Zandberg DP, Strome SE. The role of the PD-L1:PD-1 pathway in squamous cell carcinoma of the head and neck. *Oral Oncol.* 2014 Jul;50(7):627-32.
Zavala WD, Cavicchia JC. Deterioration of the Langerhans cell network of the human gingival epithelium with aging. *Arch Oral Biol.* 2006;51:1150-5.
Zelen M. The randomization and stratification of patients to clinical trials. *J Chron Dis*. 1974;27: 365-75.
Zeng J, See AP, Phallen J, et al. Anti-PD-1 blockade and stereotactic radiation produce long-term survival in mice with intracranial gliomas. *Int J Radiat Oncol Biol Phys*. 2013;86(2): 343-9.
Zhang Q, Freidlin B, Korn E, Halabi S, Mandrekar S, Dignam JJ. Comparison of futility monitoring guidelines using completed phase III oncology trials, *Clinical Trials*. 2017;14(1):48-58.
Zumsteg ZS, Cook-Wiens G, Yoshida E, et al. Incidence of oropharyngeal cancer among elderly patients in the United States. *JAMA Oncol.* 2016;2(12):1617-23.

APPENDIX I: CTEP COLLABORATIVE AGREEMENTS LANGUAGE

The agent supplied by CTEP, DCTD, NCI used in this protocol is provided to the NCI under a Collaborative Agreement (CRADA, CTA, CSA) between the Pharmaceutical Company(ies) (hereinafter referred to as "Collaborator(s)") and the NCI Division of Cancer Treatment and Diagnosis. Therefore, the following obligations/guidelines, in addition to the provisions in the "Intellectual Property Option to Collaborator" (http://ctep.cancer.gov/industryCollaborations2/intellectual_property.htm) contained within the terms of award, apply to the use of the Agent(s) in this study:

1. Agent(s) may not be used for any purpose outside the scope of this protocol, nor can Agent(s) be transferred or licensed to any party not participating in the clinical study. Collaborator(s) data for Agent(s) are confidential and proprietary to Collaborator(s) and shall be maintained as such by the investigators. The protocol documents for studies utilizing Agents contain confidential information and should not be shared or distributed without the permission of the NCI. If a copy of this protocol is requested by a patient or patient's family member participating on the study, the individual should sign a confidentiality agreement. A suitable model agreement can be downloaded from: http://ctep.cancer.gov.

2. For a clinical protocol where there is an investigational Agent used in combination with (an)other Agent(s), each the subject of different Collaborative Agreements, the access to and use of data by each Collaborator shall be as follows (data pertaining to such combination use shall hereinafter be referred to as "Multi-Party Data"):

   a. NCI will provide all Collaborators with prior written notice regarding the existence and nature of any agreements governing their collaboration with NCI, the design of the proposed combination protocol, and the existence of any obligations that would tend to restrict NCI's participation in the proposed combination protocol.

   b. Each Collaborator shall agree to permit use of the Multi-Party Data from the clinical trial by any other Collaborator solely to the extent necessary to allow said other Collaborator to develop, obtain regulatory approval or commercialize its own Agent.

   c. Any Collaborator having the right to use the Multi-Party Data from these trials must agree in writing prior to the commencement of the trials that it will use the Multi-Party Data solely for development, regulatory approval, and commercialization of its own Agent.

3. Clinical Trial Data and Results and Raw Data developed under a Collaborative Agreement will be made available to Collaborator(s), the NCI, and the FDA, as appropriate and unless additional disclosure is required by law or court order as described in the IP Option to Collaborator (http://ctep.cancer.gov/industryCollaborations2/intellectual_property.htm).-Additionally, all Clinical Data and Results and Raw Data will be collected, used and disclosed consistent with all applicable federal statutes and regulations for the protection of human subjects, including, if applicable, the *Standards for Privacy of Individually Identifiable Health Information* set forth in 45 C.F.R. Part 164.

4. When a Collaborator wishes to initiate a data request, the request should first be sent to the NCI, who will then notify the appropriate investigators (Group Chair for Cooperative Group studies, or PI for other studies) of Collaborator's wish to contact them.

5. Any data provided to Collaborator(s) for Phase 3 studies must be in accordance with the guidelines and policies of the responsible Data Monitoring Committee (DMC), if there is a DMC for this clinical trial.

6. Any manuscripts reporting the results of this clinical trial must be provided to CTEP by the Group office for Cooperative Group studies or by the principal investigator for non-Cooperative Group studies for immediate delivery to Collaborator(s) for advisory review and comment prior to submission for publication. Collaborator(s) will have 30 days from the date of receipt for review. Collaborator shall have the right to request that publication be delayed for up to an additional 30 days in order to ensure

that Collaborator's confidential and proprietary data, in addition to Collaborator(s)'s intellectual property rights, are protected. Copies of abstracts must be provided to CTEP for forwarding to Collaborator(s) for courtesy review as soon as possible and preferably at least three (3) days prior to submission, but in any case, prior to presentation at the meeting or publication in the proceedings. Press releases and other media presentations must also be forwarded to CTEP prior to release. Copies of any manuscript, abstract and/or press release/ media presentation should be sent to:

E-mail: ncicteppubs@mail.nih.gov

The Regulatory Affairs Branch will then distribute them to Collaborator(s). No publication, manuscript or other form of public disclosure shall contain any of Collaborator's confidential/ proprietary information.

APPENDIX II: PREPARATION, PROCESSING, AND SHIPPING OF PERIPHERAL BLOOD (21-AUG-2019)

To be offered to Phase II/III Patients only

The Biospecimen Kit (described below) is available from the UCSF Biobank (Contact NRGBB@ucsf.edu).

Kit Contents (per time point):

- Five (5) 10 ml Heparin Blood (green top) draw tubes
- Styrofoam tube holder
- Outer box for tube holder with plastic bag
- Study-specific specimen Transmittal (ST) Form
- Fed Ex Clinical Pak with label

Processing:

1. Sites should only draw blood samples on Monday-Wednesday (U.S. Sites).
2. Draw blood in five (5) 10 ml green top (heparin) tubes. Collect at least 8 ml per tube.
3. Mix gently.
4. Place tubes in tube holder and outer box provided with Kit.
5. Seal up tube holder box and place in Fed Ex Clinical Pak provided.
6. Ship the blood ambient for overnight morning delivery to the Mell Laboratory. The shipping label is provided with the Clinical Pak.
7. During cold weather months, protect green top tubes from freezing, which will cause hemolysis and render the tubes unusable. A room-temperature gel pack is advised.
8. If sites draw the blood sample late in the day, the site should maintain the sample at room temperature until it is shipped and note this on the ST form.

NOTE: Samples MUST be overnighted within 12 hours of collection.

NOTE: Do NOT collect and/or ship samples on Friday, Saturday, or Sunday.

NOTE: The coordinator or personnel collecting the sample MUST send a notification by email to Sharmeela Kaushal (skaushal@ucsd.edu) and Mason Kyle (mkyle@ucsd.edu) in advance and provide the tracking information.

NOTE: Do **NOT** ship peripheral blood to NRG Oncology Biospecimen Bank.

Ship specimens for this peripheral blood specimen study to:
Attn.: Sharmeela Kaushal
Assistant Director, MCC Biorepository
UCSD Moores Cancer Center Biorepository
3855 Health Sciences Dr, Room 3331 OR Room 3345-G
La Jolla, CA 92093-0819
Email: skaushal@ucsd.edu
Phone: 858-534-7302
Fax: 858-822-5380

For questions, contact:

- Sharmeela Kaushal, skaushal@ucsd.edu
- Mason Kyle (mkyle@ucsd.edu)
- Loren Mell, lmell@ucsd.edu
- Alfredo Molinolo, amolinolo@ucsd.edu

APPENDIX III (13-JAN-2021)
Medidata Patient Cloud Operational Instructions

Introduction
Electronic collection of patient-reported outcomes (ePRO) through Medidata Patient Cloud is preferred but not mandatory. Traditional paper submission is the other option. Patients who will be submitting PRO data via Patient Cloud ePRO must be registered to Patient Cloud ePRO by an authorized site user after the patient has been registered to the study. Patients may use their own device or one provisioned by the site.

Sites can use a site-specific tablet for multiple study participants. If a site-specific tablet is used, CRAs need to setup the tablet for multiple users. Multi-user mode lets multiple study participants log in to Patient Cloud with their passwords or their PIN codes on the same device.

ePRO Application Download
Note that there are multiple versions of the Medidata Patient Cloud ePRO Application. Patients should be instructed to download the version chosen by the study team for the protocol. The patient will receive an error if the wrong version is downloaded.

Patient Cloud ePRO

The following Patient Cloud App is used on this study:
Patient Cloud

CRA Site Users
Site users of Patient Cloud require the same access as Rave. Access to the trial in the Patient Cloud is granted through the iMedidata. Site users will receive an invitation to Patient Cloud and the site user must accept the invitation to begin patient registration. Users who have not previously activated their iMedidata/Rave account at the time of initial approval of site registration will also receive a separate invitation from iMedidata to activate their account. Account activation instructions are located on the CTSU website, Rave tab under the Rave resource materials (Medidata Account Activation and Study Invitation Acceptance). Please note, site users will not be able to access the study in the Patient Cloud until all required Rave and study specific trainings are completed.

Additional information on iMedidata/Rave is available on the CTSU members' website under the Rave tab at www.ctsu.org/RAVE/ or by contacting the CTSU Help Desk at 1-888-823-5923 or by e-mail at ctsucontact@westat.com.

CRA Instructions for Setting the Patient Cloud App to Multi-User Mode
Sites conducting studies entirely on-premise, where participants travel to the sites to fill out questionnaires, can use multi-user mode. Multi-user mode lets multiple study participants log in to Patient Cloud with their passwords or their PIN codes on the same device. . If patients will be using devices supplied by the institution, site staff will need to help the patient to access the device if the device is locked.

The study provider will download the Patient Cloud app to the device and set the Patient Cloud ePRO App to multi-user mode if applicable.
To switch from personal mode (default setting) to multi-user mode:

1. Tap **About** at the bottom of the log in screen.
2. Scroll to the bottom and tap **Advanced User**.
3. Tap **Mode**, then select **Multi-User**.
4. Tap **Yes** to confirm.
5. Tap the back arrows to return to the log in screen.

Note: If enabling multi-user mode on a device, it is highly recommended that completion reminders are turned off on that device.

For a video demonstration, see Show Me How to Switch to Multi-User Mode.

Patient Users

To use the Patient Cloud, patients will need to use their own device (IOS, Android phone or tablet). Short term data will only appear on the patient's device until responses are completed and submitted. The patient data will import directly into the database once the patient selects the "Submit" button and will no longer be visible on the patient's device.

Sites can provide a site-specific tablet for multiple study participant use on site. If a site-specific tablet is used, study staff need to setup the tablet for multiple users. Multi-user mode lets multiple study participants log into Patient Cloud with their passwords or their PIN codes on the same device. Refer to the paragraph above on Setting the Patient Cloud App to Multi-User Mode.

Patient Instructions for Accessing the Patient Cloud Using Your Personal Device

Downloading the Patient Cloud App

If you are using your personal device, and you do not have the Patient Cloud app, use the following instructions. When downloading the app, you must use the Apple ID or Google account associated with the device. If the Patient Cloud app is already on the device, or if you are using a provider's device, you can skip this section.

You will need an email address that you agree to use for this purpose. The e-mail address is needed to identify you on the Patient Cloud Application and for you to receive notifications to let you know when forms are due. Your e-mail address will only be used for this survey study, and will not be used for mail or marketing purposes.

If you decide to use the electronic method to complete the questionnaires, and do not have an e-mail address, you may sign up for one at no charge at many different websites. A few sites that are commonly used and will allow you to create an email address very easily are Yahoo, Gmail, and Outlook.

For iOS:

1. An Apple ID is required for downloading the Patient Cloud app.
2. Tap the *App Store* icon.
3. Search for *Medidata Patient Cloud* and follow the installation instructions.

Note: Patient Cloud is listed as an iPhone App in the App store. When using an iPad, please view the search results under iPhone apps.

For Android:

1. A Google account is required for downloading the Patient Cloud app
2. Tap the *Play Store* icon.
3. Search for *Medidata Patient Cloud* and follow the installation instructions.

Registering

You must register in order to complete and submit your study forms. When you register, you will create a username, which is your email address, and a password that allows you to log in to the Patient Cloud app.

Note**:** You must have an activation code to begin this process. If you do not have an activation code, please contact your provider.

There are two possible ways to register. Your provider may have sent you a link to a web address where you may register from any web browser, including the one on your device. The other way to register is on the Patient Cloud app.

1. If registering from the Patient Cloud app, tap Register on the bottom of the log in page. If registering on the web, open the URL shield.imedidata.com on a web browser.
2. Enter your activation code and tap Activate.
3. On the next page, read the instructions and tap Next.
4. Read the privacy notice and tap I agree. Then tap OK to confirm.
5. Enter and confirm your email address. Tap Next.
6. Enter and confirm your password. Tap Next.

7. Choose a security question by scrolling through the dropdown menu to display the question of your choice.
8. Enter your security question response.
9. Tap Create my account to complete your registration.

If you registered on the Patient Cloud app, it automatically logs you out. If you registered on the web, you are presented with the option to download the Patient Cloud app. You can then proceed to log in with the credentials you created.

Logging in to the App

1. Enter your Email and Password that you created during the registration process. (If you previously set a PIN code, just enter your four-digit PIN.)
2. Tap Log in.

Note: If you do not remember your password, tap **Forgot Password**, and follow the instructions provided.

Setting a PIN Code

The first time you log in to the Patient Cloud app, you are given the option to create a PIN code. A PIN code allows you to bypass the step of entering your email and password every time you need to log in to the Patient Cloud app. Instead, you can enter a four-digit PIN.

1. If you wish to set a PIN code the first time you log in, tap Yes when prompted.
2. Note: You can also set your PIN at a later time by tapping the options menu on the top left of most pages and selecting Set PIN.
3. Enter a four-digit PIN.
4. Re-enter the four-digit PIN to confirm.

If you forget your PIN code, tap **Forgot PIN** and you can access the app using your email and password. You may reset your PIN by tapping the options menu on the top left of most pages and selecting Set PIN.

Resetting Your Password

You can reset your password by using the options menu at the top left of most pages.

1. Tap the options menu icon.
2. Tap Reset Password.
3. Follow the instructions to reset your password.

Completing and Submitting Forms

Once logged in, forms related to your study display on the Tasks page. If you are enrolled in multiple studies, select the appropriate study first, and then select a form. New forms can appear on the Tasks page at any time, depending on how the study is designed.

There are two types of forms displayed on the Task List page:

- *Scheduled Forms* (with a icon): These forms have a "Due Date" indicator in them so you are aware of the last day by which you will need to complete the form. If the form is due in less than one day, you will see the due time in hours.
- *Anytime Forms* (with a icon): These forms have "Last Completed Time" indicator on them which tells the most recent date or time when you completed the form. If you start a form, but do not complete it, you will see an 'Incomplete" status beneath the form name, along with a half-moon icon.

1. Select the appropriate form.
2. Follow the on-screen instructions until you reach the end of the form where you are given the opportunity to review and change your responses prior to submitting.
3. Review your responses by scrolling down the list.
4. If you need to change an answer, tap the question to go back and change the answer.
5. When you are ready to submit, tap Submit Your Data.

Note: Once a form is submitted, you will be unable to edit any of your responses. In some cases, you may be asked to acknowledge your submission by entering your password.

Patient Compliance
The patient data imports directly from a device into the Rave database. There are no documents to audit. The patient-submitted electronic responses are the source documentation.
Security
All data is encrypted on the device (256 bit encryption and Hyper Text Transfer Protocol Secure [https]) and the app requires each user to have a unique username and password for access. If the user is idle for too long (5 minutes inactivity time), the app will time out and the user will need to log in again.

The data will only reside on the device for a short period of time. Once the user clicks "Submit," the data is securely transferred over HTTPS between the device and internal relay to the Rave database. Except for the patient's email address, no identifying information is stored in iMedidata. The email address is stored for what purpose? The patient's email links the device (used) and (ePRO) account to where the data is stored. The patient's email is not visible to anyone in the system.

The Patient information (email/password) does not reside in Medidata Rave EDC and the patient accounts are hidden in iMedidata from sites and LPOs.

The Patient Cloud application is 21 CFR Part 11 compliant and acts as a gateway between the device and Medidata Clinical Cloud (MCC).

Messages and information communicated to and from the Patient Cloud are encrypted and therefore this information cannot be read if intercepted while in transit.
Site checklist for activities prior to consenting a patient
- Site staff must have already completed required eLearning for the Patient Cloud application. See last bullet with hyperlink to training video library. Contact the LPO to request appropriate Rave access to register patients in Patient Cloud
- Accept study invitation at iMedidata.com
  - Note: you must be rostered in RSS and have received an invitation to Patient Cloud
- Verify the IOS or Android operating system is using the most current version
- Verify Patient Cloud app is using the most current version
- If using institutional shared devices, first patient only: Verify Patient Cloud app is in Multi-User mode
- Refer to Review Quick Reference Guides for videos and other procedural information

Patient withdraws study consent or withdraws consent from participating on ePRO
CRA must instruct the patients that are participating on ePRO who decide to withdraw consent to delete the App from their smart phones. This will prevent QOL reminders from being sent to the patient.
